# Screening of benzenesulfonamide in combination with chemically diverse fragments against carbonic anhydrase by differential scanning fluorimetry

**DOI:** 10.1080/14756366.2019.1698562

**Published:** 2019-12-04

**Authors:** Mikhail Krasavin, Stanislav Kalinin, Sergey Zozulya, Anastasiia Gryniukova, Petro Borysko, Andrea Angeli, Claudiu T. Supuran

**Affiliations:** a Institute of Chemistry, Saint Petersburg State University, Saint Petersburg, Russian Federation; b Enamine Ltd, Kyiv, Ukraine; c Taras Shevchenko National University, Kyiv, Ukraine; d Neurofarba Department, Universita degli Studi di Firenze, Florence, Italy

**Keywords:** Differential scanning fluorimetry, thermal shift assay, protein affinity, carbonic anhydrase II, fragment-based drug discovery, primary sulphonamide, zinc binding group

## Abstract

The differential scanning fluorimetry (DSF) screening of 5.692 fragments in combination with benzenesulfonamide (BSA) against bovine carbonic anhydrase (*b*CA) delivered >100 hits that either caused, on their own, a significant thermal shift (Δ*T_m_*, °C) in the protein melting temperature or significantly influenced the thermal shift observed for BSA alone. Three hits based on 1,2,3-triazole moiety represent the periphery of the recently reported potent inhibitors of *h*CA II, IX and XII which were efficacious *in vivo*. Such a re-discovery of suitable BSA periphery essentially validates the new fragment-based approach to the discovery of future CAIs. Structures of other validated fragment hits are reported.

## Graphical Abstract

**Figure:**
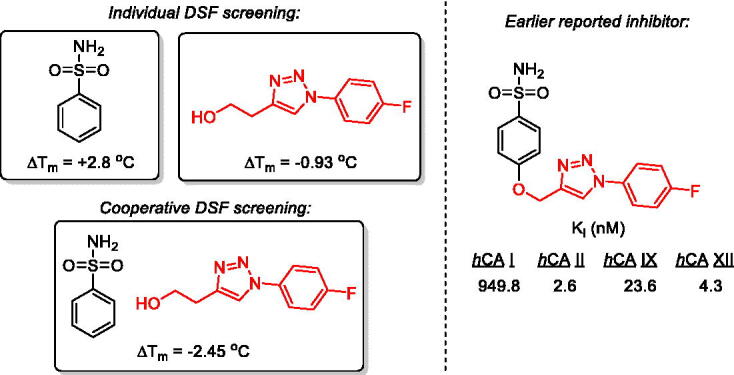

## Introduction

Differential scanning fluorimetry (DSF), also termed thermal shift assay (TSA), is an efficient technique for direct determination of a small molecule’s affinity to a protein target^1^. The underlying principle of the method is the ability of a small molecule binding to the protein to stabilise or destabilise the tertiary structure of the macromolecule and thus increase or decrease its melting temperature (*T_m_*), respectively.

Carbonic anhydrases (CAs) catalyse the fundamental biochemical process of carbon dioxide hydration (a reversible reaction producing a bicarbonate anion and a proton) and are, therefore, one of the principal regulators of cellular pH homeostasis^2^. The potential of this enzyme family as an important class of biological targets for chemotherapeutic intervention was recognised several decades ago^3^. This has led to the development of several effective drugs in areas as diverse as ophthalmology (glaucoma), metabolic disease (diabetes) and gastroenterology (gastric and duodenal ulcers)^4^.

**Figure 1.**
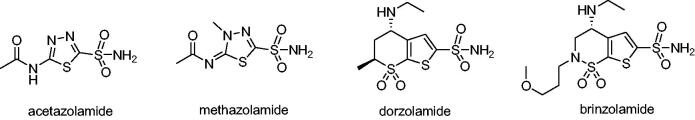
Examples of clinically used carbonic anhydrase inhibitors.

The earlier CA inhibitors (CAIs) (examples of which are shown in Figure 1) are almost exclusively non-selective, pan-inhibitors of all human CAs (of which there are currently 16 isoforms known). More recent research efforts were directed towards the discovery of isoform-selective CAIs and understanding the guiding structural principles that can help achieve the desired selectivity^5^.

**Figure 2.**
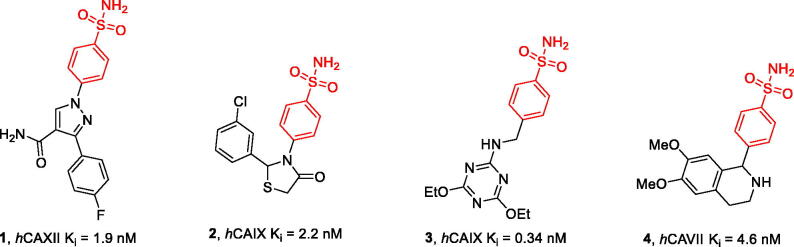
Examples of potent and isoform-selective CAIs – derivatives of benzenesulfonamide (BSA).

Primary sulphonamides are the central and most prominent class of CAIs^6^. The sulphonamide functionality in these compounds is responsible for coordination to the enzyme’s prosthetic metal ion (which is almost exclusively Zn^2+^ across the known CAs). It is, therefore, denoted as a zinc-binding group (ZBG). It is, however, the CAI molecule’s periphery that determines the potency and selectivity. This is illustrated by the evolution of the weak and non-selective CA inhibitor benzenesulfonamide (BSA)^7^ into highly potent isoform-selective sulphonamides **1–4** (Figure 2)^8–11^.

DSF is a versatile biophysical platform for fragment-based drug discovery^12^. Fragments are molecular tools of low (<300 Da) molecular weight, low lipophilicity (cLogP <3.0) and high solubility^13^ which are typically screened at high concentrations to identify those that bind to the protein target with weak (*K_d_* 0.1…1.0 mM) affinity. Considering the low molecular weight of fragments, the ligand efficiency^14^ of such fragment hits are still considerable and they are subsequently evolved (e.g. by judiciously growing their molecular periphery) into more tightly binding druglike compounds with preserved ligand efficiency^15^.

**Figure 3.**
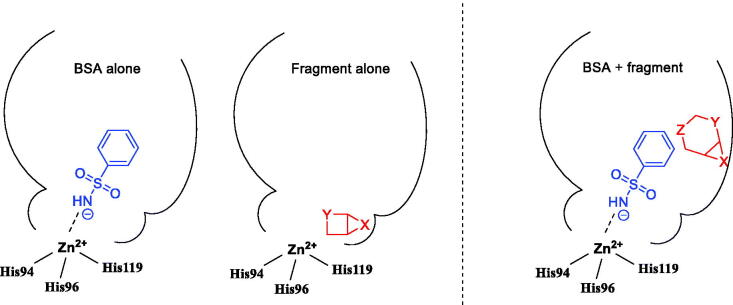
Weak binding of BSA and of a given fragment alone (A) in contrast to cooperative binding (B) associated with altered thermal shift for B *vs.* A.

We reasoned that if the low-affinity BSA is screened in combinations with various fragments, the combinations that cause greater thermal shift than BSA or fragment in question alone could signify suitable BSA periphery for the design of novel CAs. In other words, this could provide a basis for the discovery of fragments that bind to CA in cooperative fashion with BSA and thus can serve as candidates for subsequent chemical linking (random or crystallography-guided) to the BSA motif eventually leading to potent CAIs (Figure 3).

We tested this strategy using a set of 5692 diverse fragments from the Enamine, Ltd. Screening Collection^16^ screened against bovine carbonic anhydrase (*b*CA) as a model CA enzyme^17^. This led to the discovery of 110 fragments that substantially altered the thermal shift (Δ*T_m_*) of BSA and thus could be considered cooperative binders while some of the fragments thus discovered represent hitherto undescribed periphery for BSA-based CAs and will be pursued in further studies, one set of fragments corresponded to the recently described series of potent BSA-based CAIs. We consider such a rediscovery an important fact that validates the fragment-based drug discovery approach proposed above. Herein, we present this significant result in detail.

## Materials and methods

### Chemical compounds

**Figure 4.**
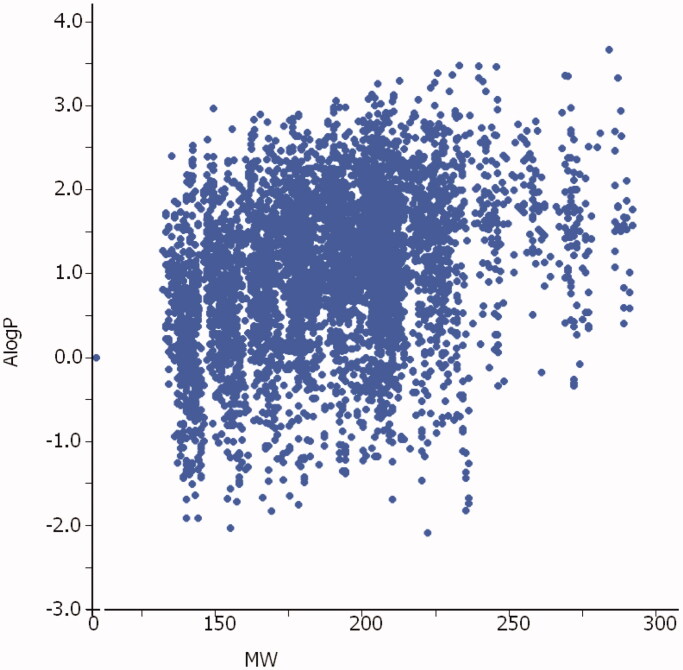
Molecular parameters (MW and ALogP^18^) of the 5692 fragments screened in this study.

The 5692 fragment compounds (for the full list, see Supplemental Material) for the DSF screening were selected by substructure search and obtained directly from the Enamine screening collection^16^. Their identity and purity was confirmed by ^1^H NMR spectroscopy prior to biochemical testing in CA inhibition assay. The majority of these compounds (3344) had molecular weight lower than 200 and were characterised by low polarity (Figure 4).

### Differential scanning fluorimetry (thermal shift) assay

Thermal shift assay was carried out using ViiA™ 7 Real-Time PCR System equipped with 384-well block (Applied Biosystems, USA). The TSA procedure was adopted from the literature^19–21^ and was modified in order to allow measuring the *b*CA melting temperature on interaction with various compounds, including the known CA inhibitor azetazolamide (AZ)^22^, which was used in this study as a reference *b*CA binder at 20 µM concentration.

For the fragment screening, the test reactions were set up in the following buffer: 10 mM NaH_2_PO_4_/Na_2_HPO_4_, pH 7.0, 10 mM NaCl. The total volume of the reaction mixture per well was 10 µL. Carbonic anhydrase (Sigma Aldrich Cat# C3934) in 300 µg/mL concentration was pre-mixed with environment-sensitive SYPRO orange dye (Invitrogen, Cat# S6650) at final concentration in the reaction of 10x, with regard to the stock concentration stated by the vendor. The mixtures were pre-incubated for 1 h at 4 °C with 20 µM concentrations of the compounds (and 1% final concentration of DMSO), placed into MicroAmp^®^ Optical 384-Well Reaction Plate (ThermoFisher, Cat# 4309849). The reaction mixture was kept at room temperature for 15 min to ensure full protein-compound interactions. The temperature was raised at 1.6 °C/s rate to 40 °C without signal reading. Starting from 40 °C up to 85 °C the heating rate was set to 0.05 °C/s with constant fluorescence reading, using 470/623 nm filter set. The raw data of dye fluorescence intensity change upon melting of the protein were obtained from the instrument ViiA 7 RUO software. Further data processing and visualisation was performed by custom-made Microsoft Excel scripts. The peak of the first derivative for the fluorescence curve was used to define melting temperature *T_m_*. *T_m_* for DMSO control wells, having only protein, dye and 1% DMSO was used as a *T_o_* to determine melting temperature shifts (Δ*T_m_*). All measurements were performed in quadruplicates.

Each fragment was screened either alone (50 µM) or, in the same concentration, in the presence of BSA (50 µM). Compounds that displayed significant (>0.5 °C) change in the thermal shift produced by BSA were subsequently tested, in quadruplicates, at three different concentrations (25, 50 and 100 µM).

## Results and discussion

**Figure 5.**
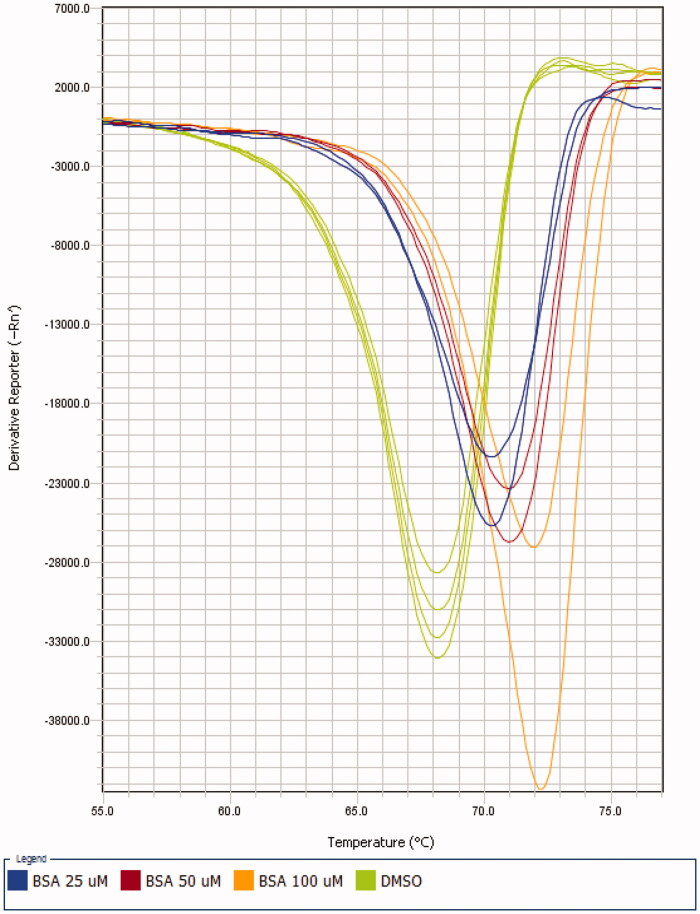
Melting curves of BSA obtained in various DSF experiments.

Benzenesulfonamide (BSA) alone produced a noticeable positive thermal shift of *b*CA melting temperature (Δ*T_m_*) which was dose dependent (Figure 5). This observation speaks for the stabilisation of *b*CA tertiary structure on BSA binding.

As expected, the Δ*T_m_* value of BSA was significantly lower than the one observed for AZ, a potent CA inhibitor, at 20 µM concentration (>5.0 °C)^22^. For the screening of fragments in combination with BSA, 50 mM concentration of the latter was chosen considering the sizeable (2.8 °C) value of thermal shift (Table 1).

**Table 1.** Values of Δ*T_m_* observed in DSF experiments of *b*CA at various concentrations of BSA.

Compound added	Observed *T_m_*, °C	*SD*	Δ*T_m_*, °C
None	68.2	±0.1	0.0
BSA (100 µM)	72.3	±0.2	4.1
BSA (50 µM)	71.0	±0.1	2.8
BSA (25 µM)	70.1	±0.1	2.0

**Figure 6.**
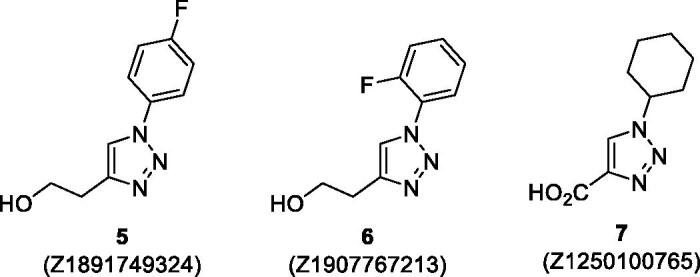
Three 1,2,3-triazole fragment hits discovered in this study (Enamine Ltd. Z-numbers are shown to aid in identifying these compounds in the Supplementary Material).

Screening of the 5692 fragments selected in this study yielded 108 hits which produced >0.5 °C effect on the thermal shift caused by 50 µM of BSA (see Supplementary Material). The vast majority of these hits will be employed in subsequent studies either as the basis for their evolution into potent CA inhibitors on their own (as some of them caused significant thermal shift). However, in terms of the cooperative effect with BSA, three fragments in particular (**5**–**7**) attracted our attention as they all contain a common 1,2,3-triazole core (Figure 6).

**Figure 7.**
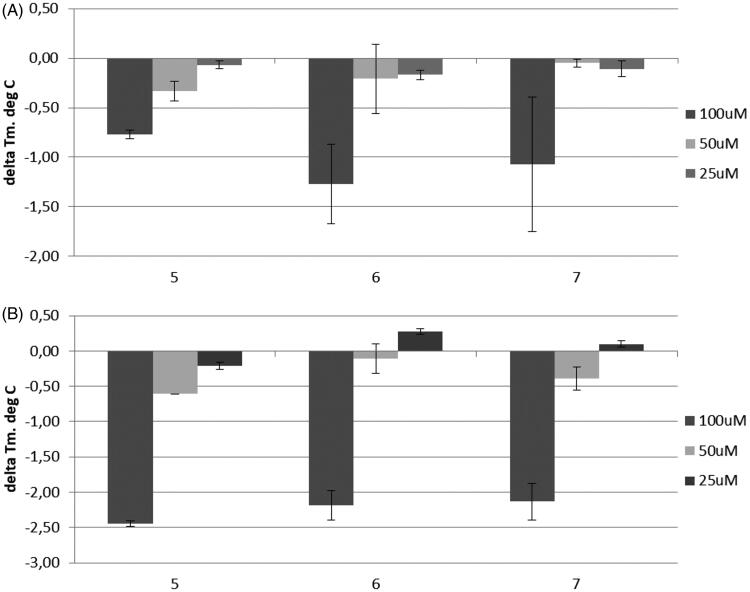
(A) Thermal shift (Δ*T_m_*, °C) observed at different concentrations of fragments **5**–**7** applied against *b*CA; (B) Δ*T_m_*, °C values observed when fragments **5**–**7** were tested in combination with BSA (50 μM).

These three hits (**5**–**7**), when tested as such against *b*CA by DSF, turned out to be negative thermal shifters, i.e. they decreased the protein melting temperature (*T_m_*, °C). This is indicative of their binding being a destabilising factor to the tertiary structure of the protein (Figure 7(A))^23^. However, the cooperative effect from these fragments tested on top of BSA was a pronounced potentiation of the negative shift, despite the fact that BSA alone displayed a substantial positive thermal shift, *vide supra* (Figure 7(B)). This can be interpreted as the most significant effect of added fragments on the thermal shift of BSA observed in this study and, therefore, 1,2,3-triazoles **5**–**7** likely represent suitable prototypes for the development of BSA into potent CA inhibitors via addition of periphery groups to the relevant positions of the benzene ring.

**Figure 8.**
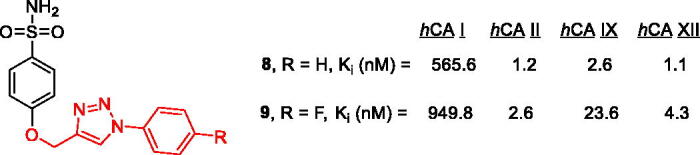
Earlier reported potent benzenesulfonamide-based CAIs incorporating flexible triazole moieties.

The discovery of the three 1,2,3-triazole fragments **5**–**7** is significant in light of the recently reported^24^ new series of potent inhibitors **8**–**9** of cytosolic *h*CA II (glaucoma target) as well as membrane-bound *h*CA IX and XII (cancer targets) that are based on benzenesulfonamide decorated with a 1,2,3-triazole moiety attached *via* a flexible linker (Figure 8). The periphery of **8**–**9** is essentially analogous to the fragments **5**–**7** and was not only justified by X-ray crystallographic studies but it was also shown that compound **8** was efficacious in lowering intraocular pressure in glaucoma animal model^24^.

## Conclusion

The differential scanning fluorimetry screening of 5.692 fragments in combination with benzenesulfonamide (BSA) against bovine carbonic anhydrase (*b*CA) delivered >100 hits that either caused, on their own, a significant thermal shift (Δ*T_m_*, °C) in the protein melting temperature or significantly influenced the thermal shift observed for BSA alone. Three such hits were of particular interest as they most significantly altered the thermal shift of BSA and are structurally related to each other and to the periphery of the recently reported series of potent *h*CA inhibitors which were efficacious *in vivo*. The findings reported in this Communication essentially validate the novel fragment approach to the discovery of new inhibitors of carbonic anhydrase. This approach is expected to eventually alleviate the need to screen larger libraries of compounds to identify potent hits. Our focus is currently on extending this approach to other fragments containing a primary sulphonamide moiety. The results of these studies will be reported in due course.

## Supplementary Material

Supplemental Material
