# Supplementary material for: Screening of benzenesulfonamide in combination with chemically diverse fragments against carbonic anhydrase by differential scanning fluorimetry
Source: J Enzyme Inhib Med Chem. 2019 Dec 4;35(1):306–10. doi: 10.1080/14756366.2019.1698562 (PMC6896451; doi:10.1080/14756366.2019.1698562)
Supplement: Supplemental Material [file IENZ_A_1698562_SM2417.pdf]

Supplemental Material for

Screening of benzenesulfonamide in combination with chemically diverse fragments against carbonic anhydrase by differential scanning fluorimetry

Mikhail Krasavin,[*] Stanislav Kalinin, Sergey Zozulya, Anastasia Grinuikova. Petro Borysko, Andrea Angeli, Claudiu T. Supuran[*]

Contents:

List of 108 validated fragment hits 2-37

Total list of 5,692 fragments screened 38-122

Enamine ID	MW	Formula	Structure
Z1259339771	144,17	C7H12O3	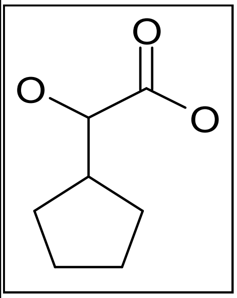
Z1891749324	207,21	C10H10FN3O	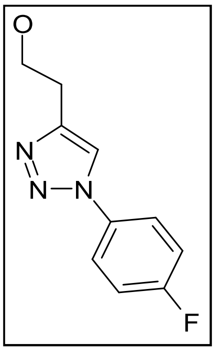
Z1907767213	207,21	C10H10FN3O	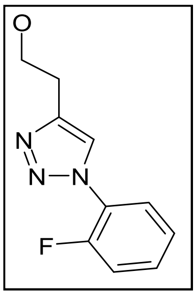

Z1250100765	195,22	C9H13N3O2	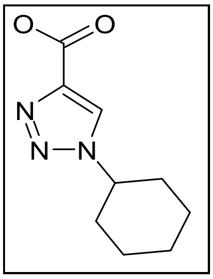
Z1741967601	233,04	C8H6BrFO2	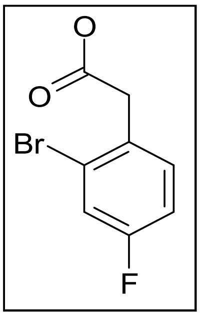
Z1639162606	203,23	C8H10FNO2S	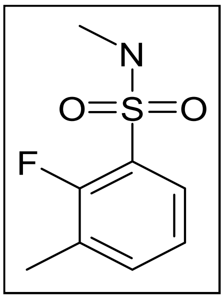

Z973151768	208,24	C11H13FN2O	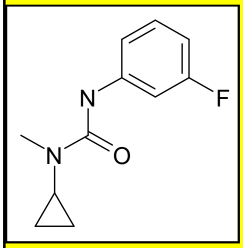
Z1699557311	203,25	C10H13N5	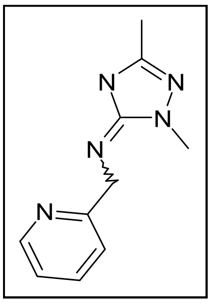
Z2068674771	221,25	C10H17F2NO2	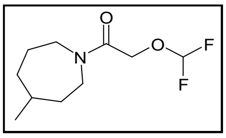

Z1517673547	193,23	C10H12FN3	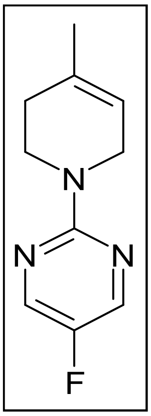
Z2327040050	136,15	C8H8O2	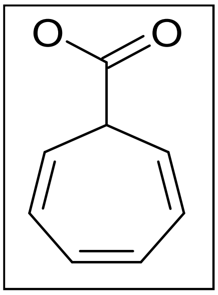
Z2235810219	178,24	C10H14N2O	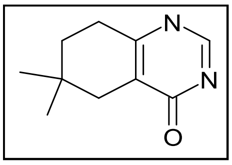

Z30802725	211,21	C11H11F2NO	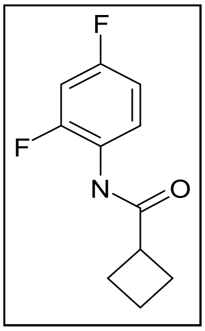
Z1262246089	203,20	C11H9NO3	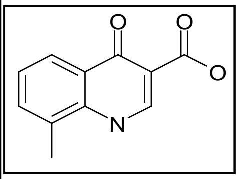
Z1436105147	165,15	C7H7N3O2	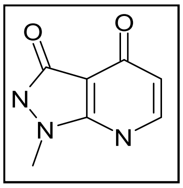

Z274553586	169,18	$C7H11N3O2$	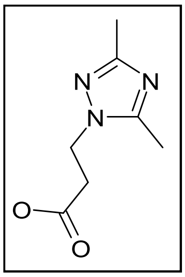
Z2583036115	144,17	$C7H12O3$	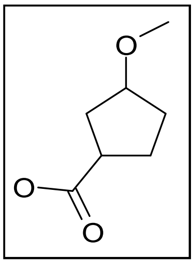
Z2168541836	161,20	$C10H11NO$	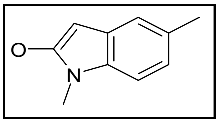

Z1267885765	164,25	C10H16N2	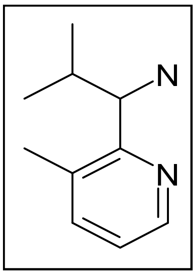
Z1262237384	196,31	C10H16N2S	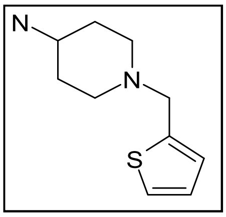
Z1258578217	171,26	C8H13NOS	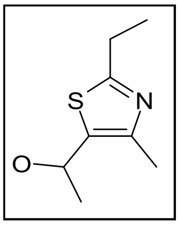

Z2472860046	206,20	C10H10N2O3	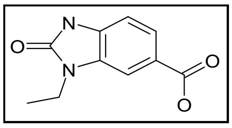
Z56347071	179,24	C9H9NOS	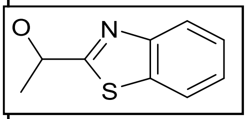
Z1269702208	147,22	C10H13N	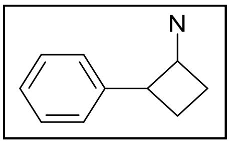

Z1741965991	181,19	$C_9H_{11}NO_3$	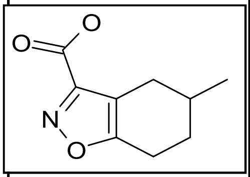
Z1889997414	230,06	$C_8H_8BrNO_2$	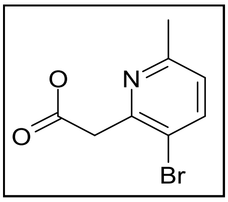
Z2465620163	168,20	$C_7H_{12}N_4O$	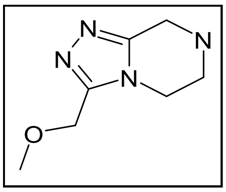

Z31791472	218,27	C11H10N2OS	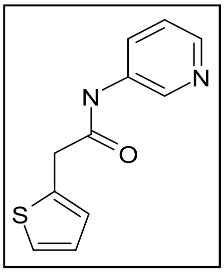
Z1878408220	242,12	C10H12BrNO	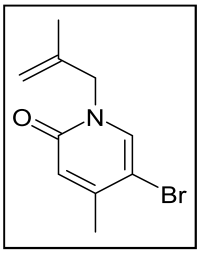
Z228574062	198,69	C10H15ClN2	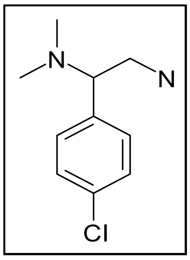

Z1603606287	225,26	C10H11NO3S	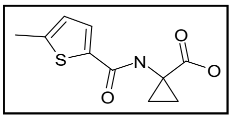
Z2285175561	229,27	C10H12FNO2S	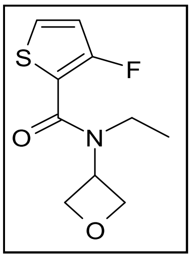
Z1983897532	223,27	C10H10FN3S	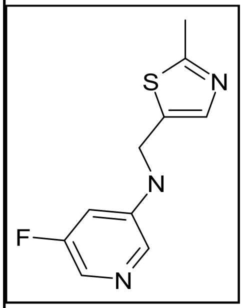

Z1779277596	213,23	C11H13F2NO	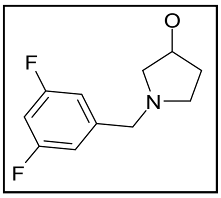
Z2301499695	166,22	C9H14N2O	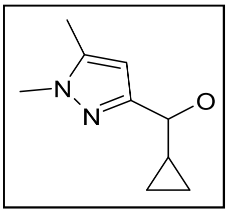
Z1103241017	205,31	C12H19N3	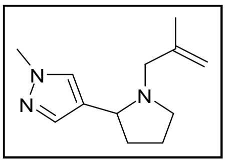

Z111834298	231,05	C8H7BrO3	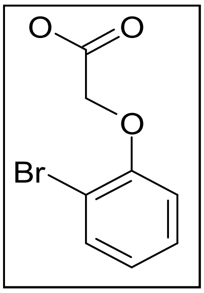
Z1365534328	209,13	C6H6F3N3O2	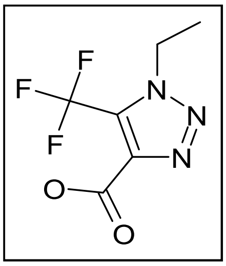
Z1838500084	209,25	C10H15N3O2	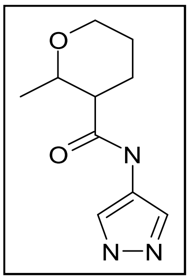

Z2510258100	152,15	C7H8N2O2	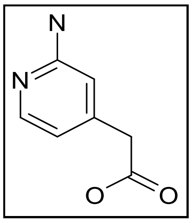
Z1460224226	228,65	C10H10ClFN2O	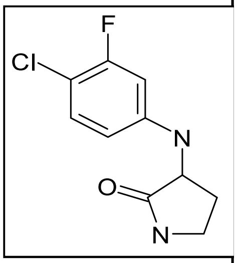
Z1267773594	165,24	C10H15NO	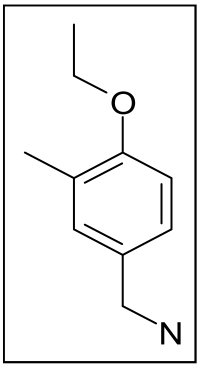

Z219186376	202,22	C10H10N4O	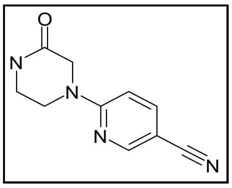
Z1238835893	229,66	C10H12ClNO3	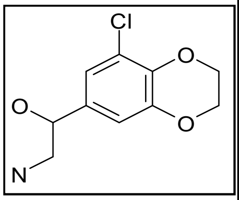
Z1796224493	212,27	C11H17FN2O	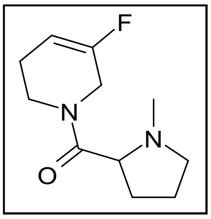

Z56347545	204,19	C10H8N2O3	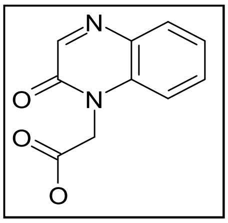
Z56347236	204,27	C12H16N2O	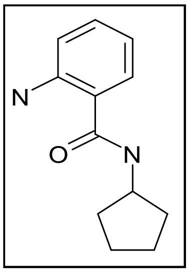
Z364575442	210,23	C10H14N2O3	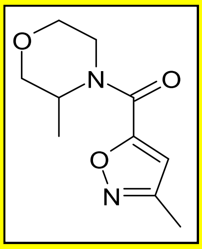

Z1649677579	237,65	C10H11ClF3N	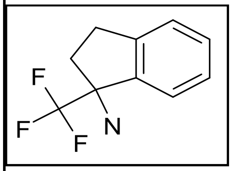
Z1474242573	208,27	C10H16N4O	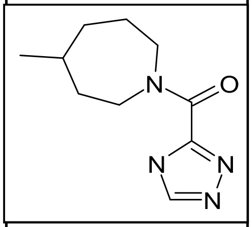
Z19629920	204,19	C10H8N2O3	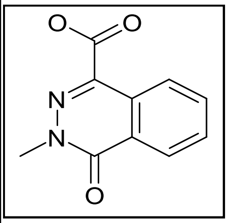

Z1272739430	191,27	C12H17NO	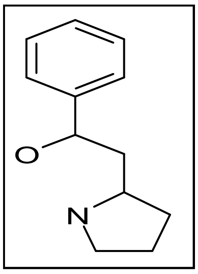
Z1874203205	226,71	C10H15ClN4	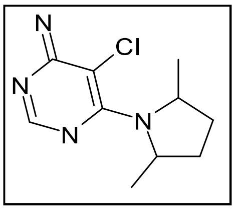
Z1267881725	200,24	C12H12N2O	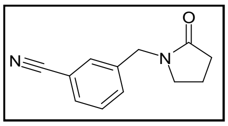

Z1262245653	219,68	C8H10ClNO2S	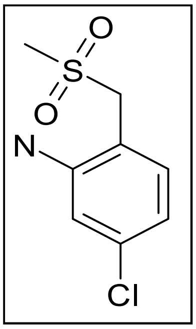
Z2054890075	212,29	C11H20N2O2	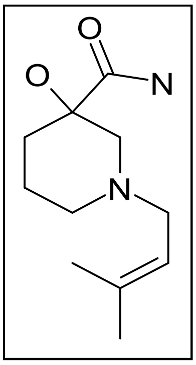
Z2327531717	202,26	C12H14N2O	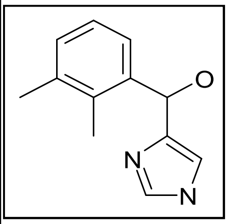

Z1250132763	168,17	C9H9FO2	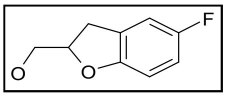
Z2182115458	157,19	C6H7NO2S	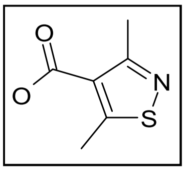
Z57127001	151,17	C8H9NO2	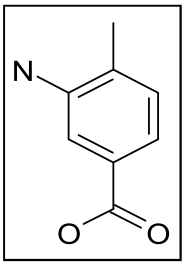

Z2218585849	154,22	C7H14N4	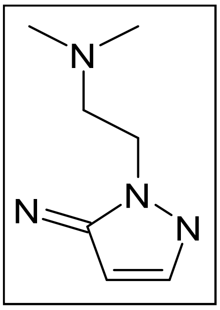
Z57980675	193,27	C9H11N3S	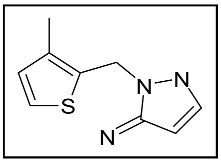
Z57047005	198,19	C10H11FO3	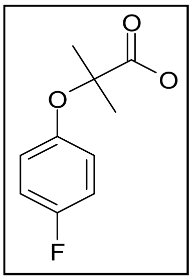

Z1262252948	167,21	C8H13N3O	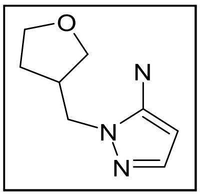
Z1271843038	141,21	C8H15NO	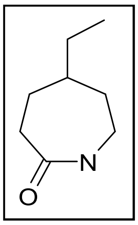
Z2037273251	168,19	C9H12O3	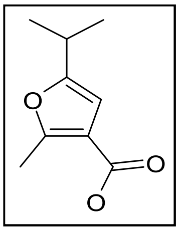

Z373775848	221,28	$C_{10}H_{11}N_3OS$	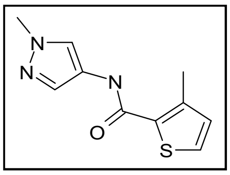
Z31504642	165,19	$C_9H_{11}NO_2$	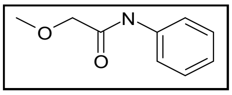
Z1262237439	184,28	$C_{10}H_{20}N_2O$	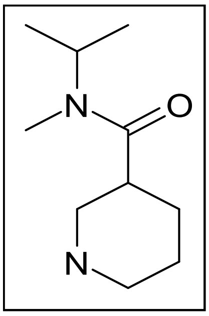

Z1573313752	223,27	C10H10FN3S	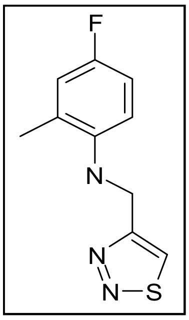
Z1337784682	197,21	C10H12FNO2	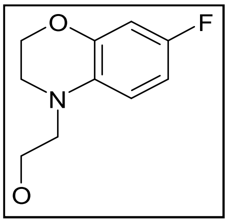
Z1082510666	208,22	C10H12N2O3	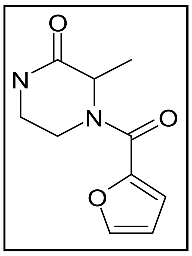

Z2235387992	196,29	C11H20N2O	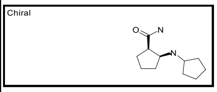
Z2574918328	179,27	C10H17N3	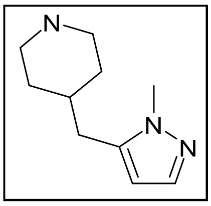
Z2034364269	138,17	C7H10N2O	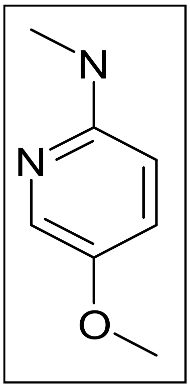

Z2510259511	164,18	C5H8O4S	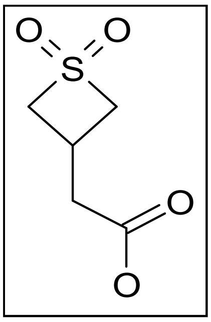
Z2510259303	167,17	C7H9N3O2	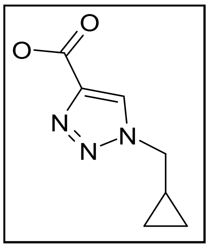
Z2327531729	167,21	C9H13NO2	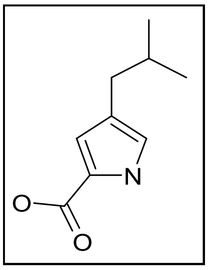

Z2168390419	167,16	C8H9NO3	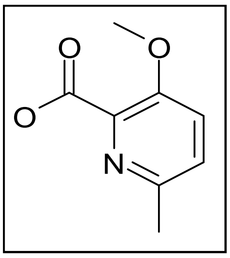
Z1262237455	168,26	C8H12N2S	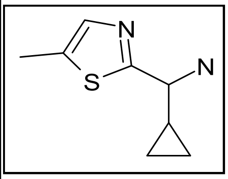
Z234897683	201,27	C12H15N3	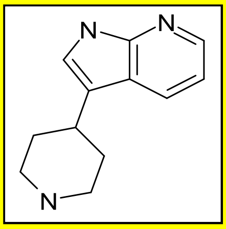

Z929052328	292,15	C9H10BrNO3S	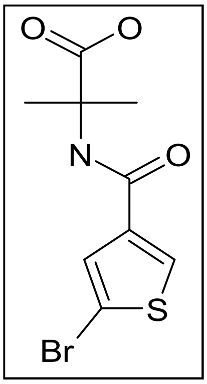
Z1259273099	188,13	C8H6F2O3	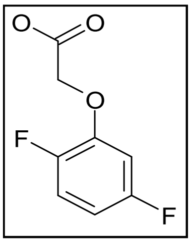
Z1259162080	168,24	C10H16O2	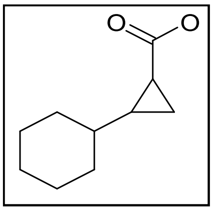

Z1796028750	193,22	$C_{11}H_{12}FNO$	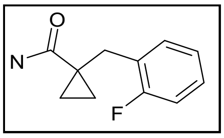
Z281802048	153,19	$C_7H_{11}N_3O$	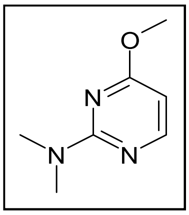
Z2327884032	158,25	$C_8H_{18}N_2O$	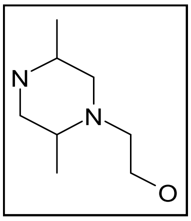

Z422368058	226,29	C10H14N2O2S	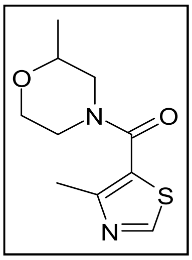
Z2027049516	254,09	C9H8BrN3O	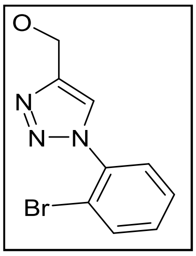
Z1820273488	225,28	C8H7N3OS2	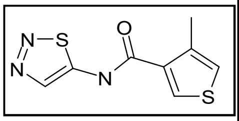

Z1272739859	230,06	C8H8BrNO2	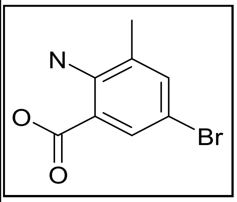
Z1276496838	201,27	C12H15N3	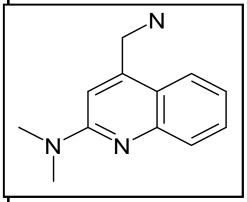
Z2177031006	177,25	C11H15NO	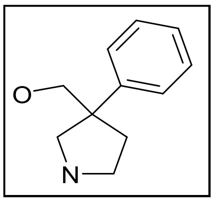

Z57040473	163,18	C8H9N3O	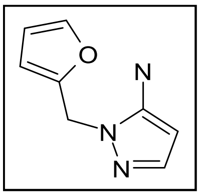
Z1232688349	200,25	C11H12N4	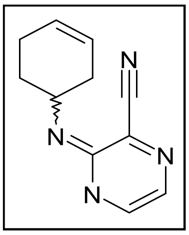
Z2240988377	185,32	C10H23N3	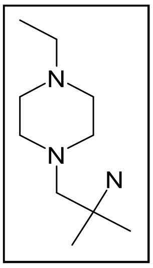

Z1741815708	142,25	C8H18N2	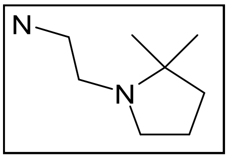
Z234893819	148,17	C8H8N2O	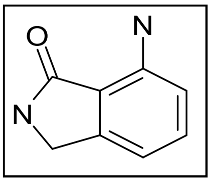
Z85917566	147,18	C9H9NO	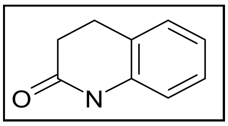

Z228474642	187,25	C11H13N3	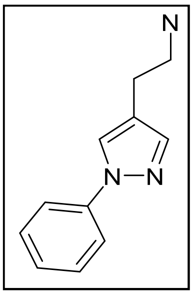
Z1271843050	184,20	C11H8N2O	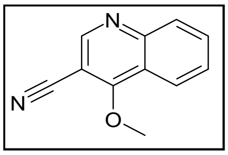
Z1791179790	223,25	C9H9N3O2S	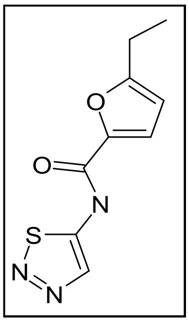

Z1268152406	191,28	C11H17N3	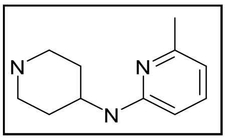
Z1267886876	192,22	C10H12N2O2	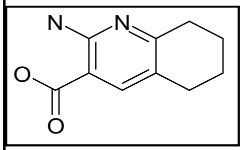
Z1267882601	187,25	C11H13N3	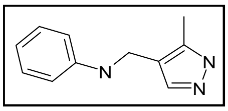

Z1891772658	187,20	C8H13NO4	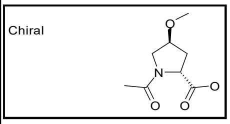
Z2467541171	140,23	C9H16O	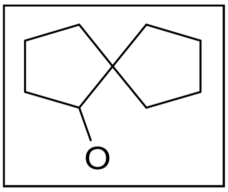
Z2168499582	226,10	C8H3F5O2	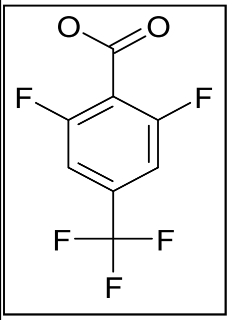

Enamine ID	SMILES	MW	Molecular Formula
Z1259339771	OC(C1CCCC1)C(O)=O	144,17	C7H12O3
Z1891749324	OCCC1=CN(N=N1)C1=CC=C(F)C=C1	207,21	C10H10FN3O
Z1907767213	OCCC1=CN(N=N1)C1=C(F)C=CC=C1	207,21	C10H10FN3O
Z1250100765	OC(=O)C1=CN(N=N1)C1CCCCC1	195,22	C9H13N3O2
Z1868316789	CCOC1=C(C(O)=O)C(C)=CC=N1	181,19	C9H11NO3
Z2188171616	CC1=C(C=CC(N)=C1)C1=CC=NC=C1	184,24	C12H12N2
Z2009840048	NC1CC(=O)N2CCCCC12	154,21	C8H14N2O
Z1262237414	CC(CN)CN1CCCC1=O	156,23	C8H16N2O
Z1823335958	CC1=C(C=CO1)S(=O)CC1=NC(C)=CS1	241,32	C10H11NO2S2
Z1350579526	CC(N)C1=CC=C(C=C1)N1C=CN=C1C	201,27	C12H15N3
Z2194169420	CNCC1CCCOC1C1=CC=CC=C1	205,30	C13H19NO
Z1267773501	NCCC1=NN(C=C1)C1=CC=C(F)C=C1	205,24	C11H12FN3
Z951367728	CC(CN)C1=NC2=C(S1)C=CC=C2	192,28	C10H12N2S
Z267168630	CC1=NOC(CNC2=C(C)C=CC=C2)=C1	202,26	C12H14N2O
Z1272670424	CC1(CCC2=C(C1)C=CC=C2)C(O)=O	190,24	C12H14O2
Z1270207977	CC1CNC2=C(C=CC=C2)N(C1)C(C)=O	204,27	C12H16N2O
Z212851096	CC1CN(CCO1)C(=O)C1=NC=CN=C1	207,23	C10H13N3O2
Z1238477809	CCC(O)CN1CCNCC1	158,25	C8H18N2O
Z101186134	NC(=O)CC1=NOC2=C1C=CC=C2	176,18	C9H8N2O2
Z56871715	COC1=C(C=C(Cl)C=C1)C#N	167,59	C8H6ClNO
Z2216303152	NC1=CC2=C(OC(CO)=C2)C=C1	163,18	C9H9NO2
Z2211231539	COC1=CC2=C(NC(=O)C(C)N2)C=C1	192,22	C10H12N2O2
Z2255626880	CC1CNCC(O1)C1CC1	141,21	C8H15NO
Z108566670	CNC1=C(C(O)=O)C(C)=NS1	172,20	C6H8N2O2S
Z224184044	CCC(=O)NC1=C(C=CC=C1C)C(O)=O	207,23	C11H13NO3
Z14775230	NC(=O)COC1=C(Cl)C=CC(Cl)=C1	220,05	C8H7Cl2NO2
Z16115067	CC1=CC(OCC(N)=O)=C(Cl)C=C1	199,63	C9H10ClNO2
Z212063058	CC(=O)NC1=CC=C(S1)C(O)=O	185,20	C7H7NO3S
Z55410632	CN1N=CC(C(N)=O)=C1N	140,15	C5H8N4O
Z212062948	CC1=C(SC(NC(=O)C2CC2)=C1)C(O)=O	225,26	C10H11NO3S
Z219997506	OC(=O)CC1=C(Cl)C=C(Cl)C=C1	205,03	C8H6Cl2O2
Z45202427	CC(NC1=C(C)C(C)=CC=C1)C(N)=O	192,26	C11H16N2O
Z207209754	CN1C(=O)C(C)=NC2=C1C=CC=C2	174,20	C10H10N2O
Z1863617838	NC1=C2CNCCC2=CC=N1	149,20	C8H11N3
Z1863617794	CC(C)NC1=NC=CN1C	139,20	C7H13N3
Z1667555483	CN1N=C(C)N=C1NC(=O)C1=CC=CO1	206,21	C9H10N4O2
Z1729204367	COC1=C(Cl)C=C(CN2C=CN=N2)C=C1	223,66	C10H10ClN3O
Z1743510401	CC1=CC(NCC2(C)CCOCC2)=NO1	210,28	C11H18N2O2
Z1860991558	CC1=CC(=O)C2=C(N1)C=CC(N)=C2	174,20	C10H10N2O
Z169675004	CN1C=CC(NC(=O)CN2C=CC=N2)=N1	205,22	C9H11N5O
Z1833679631	CCN(C)C(=O)NC1=CN=NS1	186,23	C6H10N4OS
Z1889902494	CC1=C(C=CO1)C1=CC(N)=NO1	164,16	C8H8N2O2
Z1881599143	OCC1CCC(CO)CC1	144,21	C8H16O2
Z1657810189	CN1C(C)=NN=C1NC(=O)CC1CCC1	208,27	C10H16N4O
Z1020680176	NCC(O)C1=CC=C(Cl)C=C1	171,62	C8H10ClNO
Z228042642	OC(=O)CN1CC(=O)NC2=C1C=CC=C2	206,20	C10H10N2O3
Z1575143888	NCC1CCCN(C1)C1=NC=CC=N1	192,27	C10H16N4
Z1575143894	COC1=CC(N)=C(F)C=C1	141,15	C7H8FNO
Z1575143702	NCC1=NOC2=C1C=CC=C2	148,17	C8H8N2O
Z2216711752	CC(C)C1(CO)CCCNC1	157,26	C9H19NO
Z234893683	NCCC1=NC=C(C=C1)C(F)(F)F	190,17	C8H9F3N2
Z1251207602	CC1=NN=C(S1)N1CCCCC1	183,27	C8H13N3S
Z1255637874	CCC1=CSC(=N1)C1=C(N)C=CC=C1	204,29	C11H12N2S
Z324556956	CNC(=O)C1CCCCN1S(C)(=O)=O	220,29	C8H16N2O3S
Z1171979277	OC(=O)C1=CNN=C1C1CCCCC1	194,23	C10H14N2O2
Z993967108	CCC(C(O)=O)C1=C(C)ON=C1C	183,21	C9H13NO3
Z1267773535	NCCC1=C(F)C=C(F)C=C1	157,16	C8H9F2N
Z1258578241	CC(C)C(O)C1=NC=CC=C1	151,21	C9H13NO
Z1020680358	CC(O)(CN)C1CCCC1	143,23	C8H17NO
Z1230795906	CN1C=CN=C1C(=O)NCC=C	165,20	C8H11N3O
Z53038390	CNS(=O)(=O)C1=CC(C(O)=O)=C(C)C=C1	229,25	C9H11NO4S
Z2492774597	NC1=CC2=C(CS(=O)(=O)C2)C=C1	183,23	C8H9NO2S
Z2216710432	CCNS(=O)(=O)C1=CC(C)=C(Cl)C=C1	233,71	C9H12ClNO2S
Z1416200983	OC(=O)C1=CN2C(C=C1)=NN=C2C(F)F	213,14	C8H5F2N3O2
Z1741980409	C1CNC2=C(C1)C=CC=N2	134,18	C8H10N2
Z1891772663	CC(=O)NC(C1CCOCC1)C(O)=O	201,22	C9H15NO4

Z2509351220	BrC1=CC2=C(OCCNC2=O)C=C1	242,07	C9H8BrNO2
Z1982493933	CN1C(=O)COC2=C1C=CC(N)=C2	178,19	C9H10N2O2
Z344843836	CCN(C)C(=O)CC1=C(Cl)C=CC=C1Cl	246,13	C11H13Cl2NO
Z1973465555	CC1=C(C=C(O1)C#N)C(O)=O	151,12	C7H5NO3
Z1966484358	CC1=C(C(C)=NO1)C(C)(C)C(O)=O	183,21	C9H13NO3
Z1891775602	CC(=O)NC1=C(N)C=CC=N1	151,17	C7H9N3O
Z57261333	CC1=C(Cl)C=C(C=C1)C(=O)NCCO	213,66	C10H12ClNO2
Z1945984210	CN(C)C(=O)NC1=C(C)C(N)=CC=C1	193,25	C10H15N3O
Z272156568	CCOC1=C(C=CC=N1)C(N)=O	166,18	C8H10N2O2
Z1741978641	OC(=O)C1=CN2CCCCC2=N1	166,18	C8H10N2O2
Z229615464	CN1N=C(C)C2=C1SC(=C2)C(N)=O	195,24	C8H9N3OS
Z241898600	CN1N=C(C)C2=C1N=C(C)C=C2C(N)=O	204,23	C10H12N4O
Z239136710	CC(=O)NC1=CC(C)=NN1	139,16	C6H9N3O
Z2692095185	CN1N=CC=C1C(N)C1=CC=NN1C	191,24	C9H13N5
Z2692095209	NC1=NC(=NS1)C1CCOCC1	185,25	C7H11N3OS
Z1255634340	CC(C)C1=NC(=CN1)S(=O)(=O)N(C)C	217,29	C8H15N3O2S
Z2692093625	NC(CCO)C1=CC(F)=C(Cl)C=C1	203,64	C9H11ClFNO
Z1821397907	CC(C)CC1(CO)CCCN1	157,26	C9H19NO
Z1259155925	CC1(O)CCC2=C(C1)C=CC=C2	162,23	C11H14O
Z1259155865	COC1=C(C=CC=C1)C1(CC1)C(O)=O	192,21	C11H12O3
Z1741967601	OC(=O)CC1=C(Br)C=C(F)C=C1	233,04	C8H6BrFO2
Z1849008050	CC1=CSC(C(O)=O)=C1Br	221,07	C6H5BrO2S
Z1849010842	CN(C)S(=O)(=O)C1=CN=C(N)C=C1	201,24	C7H11N3O2S
Z1348386878	O=S1CCN(CC1)C1=CN=C(C=C1)C#N	221,28	C10H11N3OS
Z1839475324	OC(=O)C1=CC=CN1CC=C	151,17	C8H9NO2
Z1127744289	NC(=O)C1=CC=C(CN2C=CC=C2)C=C1	200,24	C12H12N2O
Z1837073463	NC1C(CO)CCC2=C1C=CC=C2	177,25	C11H15NO
Z57899965	COC1=CC(=CC(OC)=C1)C#N	163,18	C9H9NO2
Z1183649742	CCC1=CC2=C(C=C1)C(Cl)=C(S2)C(N)=O	239,72	C11H10ClNOS
Z1834278447	CN1OC(=CC1=O)C(O)=O	143,10	C5H5NO4
Z1696930122	CC1=CSC=C1C(=O)NC1=NC=CN=C1	219,26	C10H9N3OS
Z1827897963	O=C1CN(CCCN1)C1CC1	154,21	C8H14N2O
Z1682035348	CC1CN(CC2=CSN=N2)C(C)(C)CO1	227,33	C10H17N3OS
Z29692196	CN1C=CC=C1C(=O)NCC1=CC=CS1	220,29	C11H12N2OS
Z1824511099	CC1=CC(=O)C2=C(N1)C=CS2	165,21	C8H7NOS
Z1258992459	OCC1=CC(=CC=C1)C#N	133,15	C8H7NO
Z1258992442	CC(O)C1=NC2=C(N1)C=CC(Cl)=C2	196,63	C9H9ClN2O
Z1173707511	OC1(CNC2=NC=C(Br)C=N2)CCC1	258,12	C9H12BrN3O
Z1258854546	CCNC(=O)N1CCC2=C1C=C(N)C=C2	205,26	C11H15N3O
Z1258578989	COC1=C(C=CC=C1)C1(C)CC1C(O)=O	206,24	C12H14O3
Z1258578330	CC(C)C1=NC=NC(O)=C1	138,17	C7H10N2O
Z1258578306	CC(O)C1=CC2=C(NC(=O)C2)C=C1	177,20	C10H11NO2
Z1251222566	NCC1=CC2=C(O1)C=CC=C2	147,18	C9H9NO
Z751811134	CN(C)S(=O)(=O)C1=CN=CC=C1	186,23	C7H10N2O2S
Z1251171287	CC1=CC(CCN)=CC(C)=C1	149,24	C10H15N
Z1259335848	CCN1N=CC2=C1C=C(N)C=C2	161,21	C9H11N3
Z1259341108	CC1=CC(C)=C(C=C1)C1=NN=C(N)S1	205,28	C10H11N3S
Z1259341095	CCOC1=NC=CC(=C1)C(O)=O	167,16	C8H9NO3
Z1259341091	CCC1=C(C=NC(C)=N1)C(O)=O	166,18	C8H10N2O2
Z1259339962	CN1C=C(CCC(O)=O)C(C)=N1	168,20	C8H12N2O2
Z1259162172	NC(C(O)=O)C1=C(C=CC=C1)C(F)(F)F	219,16	C9H8F3NO2
Z1259162070	CC(CC1=C(C)NN=C1C)C(O)=O	182,22	C9H14N2O2
Z1623889816	FC1=CCCN(C1)C1=NC=C(C=C1)C#N	203,22	C11H10FN3
Z1583292118	CC1=NN=C(CNC2=C(F)C=CC=C2)S1	223,27	C10H10FN3S
Z1354370680	CN(CCO)C1=C(F)C=C(Cl)C=N1	204,63	C8H10ClFN2O
Z1980875175	CC(NC1=NC(C)=NS1)C1=NNC=C1	209,27	C8H11N5S
Z262247854	COCC(=O)NC1(C)CCS(=O)(=O)C1	221,27	C8H15NO4S
Z1262327459	CC(C)C(=O)NCC1CCCNC1	184,28	C10H20N2O
Z2012523861	CC(=O)NC1=C(F)C=C(OC(F)F)C=C1	219,16	C9H8F3NO2
Z2687203946	NC1(COC2=C(C1)C=CC=C2)C(O)=O	193,20	C10H11NO3
Z1497330165	CNC(=O)C1=C(F)C=C(C=C1)N(C)C	196,23	C10H13FN2O
Z1639162606	CNS(=O)(=O)C1=C(F)C(C)=CC=C1	203,23	C8H10FNO2S
Z2684295076	OCCC1CCCNC1=O	143,19	C7H13NO2
Z2680629390	NC1=C(C=CC=C1)N1CCNC(=O)C1	191,23	C10H13N3O
Z1171979278	CC(C)CC1=NC(C)=C(S1)C(O)=O	199,27	C9H13NO2S
Z1171978788	CN(CC(O)=O)C(=O)C1=NC=CC=C1	194,19	C9H10N2O3
Z1171978806	CC(C(O)=O)C1=C(C)NN=C1C	168,20	C8H12N2O2

Z333523646	CCC(=O)NC1CCCC2=C1C=CO2	193,25	C11H15NO2
Z434911440	CN(CC(N)=O)C(=O)C1=C(Br)C=CS1	277,14	C8H9BrN2O2S
Z31111899	C(NC1=NC=CC=C1)C1=CC=CS1	190,26	C10H10N2S
Z287484230	CN1N=C(C)C(CC(N)=O)=C1C	167,21	C8H13N3O
Z1770193310	CC1=COC(=C1C(O)=O)C1=CC=CC=C1	202,21	C12H10O3
Z1768428973	COC(C)C1=NC=C(S1)C(O)=O	187,21	C7H9NO3S
Z1666810169	FC1=C(NC(=O)C2CC=CC2)N=CC=C1	206,22	C11H11FN2O
Z1762658235	CC1=C(N)C(C(O)=O)=C(Br)C=C1	230,06	C8H8BrNO2
Z1616719042	CN(C)C(=O)N(C)C1(CCCC1)C(N)=O	213,28	C10H19N3O2
Z1593013689	CN(C)C1CN(C1)C1=NC=C(Cl)C=N1	212,68	C9H13ClN4
Z1603606863	OC(=O)C1(CC1)NC(=O)C1=C(Br)C=CS1	290,13	C9H8BrNO3S
Z1742055008	CC1=COC2=C1C=C(N)C=C2	147,18	C9H9NO
Z1741880725	CC1CCCCC1(N)C(O)=O	157,21	C8H15NO2
Z1718361780	NC1=C(C=NC(=N1)C1CC1)C#N	160,18	C8H8N4
Z1272500940	C1C2CC=CCC2CN1C1=NC=CN=C1	201,27	C12H15N3
Z1407004019	CCOC1=NC=C(N)C=C1	138,17	C7H10N2O
Z1681200613	OC1(CC2=NC(=NO2)C2CC2)CCCC1	208,26	C11H16N2O2
Z1289535961	CC1CS(=O)(=O)CCN1CC1CCC1	217,33	C10H19NO2S
Z90517838	C(NC1CC1)C1=CC=CO1	137,18	C8H11NO
Z1271660837	CCNCC1=CN(C)N=N1	140,19	C6H12N4
Z2335495746	CCOC1=CC(F)=C(NC(C)=O)C=C1	197,21	C10H12FNO2
Z2106611472	CC1=CN(CC(O)=O)N=C1C(F)(F)F	208,14	C7H7F3N2O2
Z2689172057	COC1=CC(=CC=C1)C1=C(N)NN=C1C	203,25	C11H13N3O
Z2146515783	CN1C(C)=NC2=C1N=CC(Br)=C2	226,08	C8H8BrN3
Z2146513621	CNS(=O)(=O)C(C)C1=CC=CC=C1	199,27	C9H13NO2S
Z2146514261	CC(O)C1=NC=C(C=C1)N(C)C	166,22	C9H14N2O
Z2092369803	COC1=C(OC)C(C)=C(C=C1)C(O)=O	196,20	C10H12O4
Z2092368585	CC1(C)CNC(=O)C(C)(C)N1	156,23	C8H16N2O
Z1265814003	CNC(=O)C1=C(C)C=CC(Br)=C1	228,09	C9H10BrNO
Z1787644587	CC(O)C(=O)NCC1=C(C)C=CC(Br)=C1	272,14	C11H14BrNO2
Z2160914711	COC1=CC2=C(C=C1)C(=O)NN2	164,16	C8H8N2O2
Z1262237375	CNC1=CN=C(OC)C=C1	138,17	C7H10N2O
Z45534888	O=S(=O)(NC1CC1)C1=CC=CS1	203,27	C7H9NO2S2
Z1688198928	OC(=O)C1CC11CCOC1	142,15	C7H10O3
Z1302595798	CN(C)C(=O)C1=CC(=NO1)C1CCCC1	208,26	C11H16N2O2
Z1139507648	CCN1CCCC1C1=C(C)N(C)N=C1C	207,32	C12H21N3
Z1741794221	OC(=O)CC1CCCC=C1	140,18	C8H12O2
Z1741794235	CC1=NC2=C(C=CC=C2)N=C1	144,18	C9H8N2
Z234895091	NCC1CC2=C1C=CC=C2	133,19	C9H11N
Z1696871223	CC(C)C1=NC(=NC=C1)N1CCNCC1	206,29	C11H18N4
Z56040660	CCC(C)NC(=O)C1=C(N)C=CC=C1	192,26	C11H16N2O
Z105197244	NCC1=NC(=NO1)C1=CC=CS1	181,21	C7H7N3OS
Z910841232	CC(=O)NC1=C(C=C(C)C=C1C)C(O)=O	207,23	C11H13NO3
Z85956709	CC(N)C1=CC=C(C=C1)N1C=NC=N1	188,23	C10H12N4
Z57841909	CC(N1C=NC2=C1C=CC=C2)C(O)=O	190,20	C10H10N2O2
Z55692910	C1CC1C1=NC2=C(N1)C=CC=C2	158,20	C10H10N2
Z71501992	CNC(=O)C1=C(C=CS1)C1=CC=CC=C1	217,29	C12H11NOS
Z111781340	CC1=NC2=C(C=NN2C(C)=C1)C(O)=O	191,19	C9H9N3O2
Z228590120	COC1=CC=C(C=C1)C1=NNC(N)=C1C	203,25	C11H13N3O
Z992422568	NCC1=C(C=C(Br)C=C1)C(F)(F)F	254,05	C8H7BrF3N
Z969560582	CNC(=O)C1=CN=C(C)S1	156,20	C6H8N2OS
Z1416282800	NC[C@@H](O)CN1CCOCC1	160,22	C7H16N2O2
Z973151768	CN(C1CC1)C(=O)NC1=CC(F)=CC=C1	208,24	C11H13FN2O
Z280818240	CC(C1CC1)N(C)C(=O)C1=C(C)C=CO1	207,27	C12H17NO2
Z265564928	CC1CCCCN1CC1=NC(C)=NO1	195,27	C10H17N3O
Z382767716	CCC1CCCN(CC2=NC(C)=NO2)C1	209,29	C11H19N3O
Z103891156	OC1CCN(CC2=CC=C(Cl)S2)CC1	231,74	C10H14ClNOS
Z416341642	CC(C)N(C)S(=O)(=O)N1CCOC(C)C1	236,33	C9H20N2O3S
Z1241573367	CC(CC1=NC(C)=CS1)N1C=C(Cl)C=N1	241,74	C10H12ClN3S
Z217102784	CC(N)C1=NC2=C(C=CC=C2)N1C(F)F	211,22	C10H11F2N3
Z2327402244	COC1=CC=C(C=C1)N1C(=O)NC=C1C	204,23	C11H12N2O2
Z2327226168	CN1C=C(C(O)=O)C(=N1)C1CCC1	180,21	C9H12N2O2
Z2327226126	CC1=C(Br)C(F)=CC(N)=C1	204,04	C7H7BrFN
Z274767396	CCN1N=C(C)C(N)=C1C	139,20	C7H13N3
Z1218147280	CC(N1CC(C)C2=C1C=CC=C2)C(O)=O	205,26	C12H15NO2
Z2309759405	CNC1=CC=C(C=C1)C1=NC=CS1	190,26	C10H10N2S
Z2306626038	CN1CC2=C(N)C=CC=C2C1=O	162,19	C9H10N2O

Z2305986981	CCN1C(C)=CC2=C(C(N)=NN2)C1=O	192,22	C9H12N4O
Z274799154	OCC1CN2CCC1CC2	141,21	C8H15NO
Z2293608485	OC(=O)C1CN(C1)C1CCCCC1	183,25	C10H17NO2
Z2293606411	CC1=CC(Br)=C(S1)C(O)=O	221,07	C6H5BrO2S
Z2284390049	BrC1=CC(=CC=C1)N1CCCNC1=O	255,12	C10H11BrN2O
Z2284399991		172,22	C9H16O3
Z1891776116	NCC1=CC(NC2CC2)=NC=C1	163,22	C9H13N3
Z1267773690	CC1=C(Br)C=CC(C(O)=O)=C1N	230,06	C8H8BrNO2
Z1266854952	CN(C)C(=O)CN1CCC(N)CC1	185,27	C9H19N3O
Z1266854930	CNS(=O)(=O)C1CCNCC1	178,25	C6H14N2O2S
Z1266823218	CCC(C1=NN=C(N)S1)C1=CC=CC=C1	219,31	C11H13N3S
Z1263824428	CCOC1=C(N)C=C(OC)C=C1	167,21	C9H13NO2
Z1263820490	NC(=O)C1=C(CO)C=CC=C1	151,17	C8H9NO2
Z1263820443	COC1=CC(=NC=C1)N1CCNCC1	193,25	C10H15N3O
Z1263820309	CNS(=O)(=O)C1=C(CN)C=CC=C1	200,26	C8H12N2O2S
Z1263714062	NC(CO)CC1=CNC2=C1C=CC=C2	190,25	C11H14N2O
Z1263602405	CCOC1=C(N)C=C(Br)C=C1	216,08	C8H10BrNO
Z1245735276	OC1(CCCC1)C1=CC=CC=C1	162,23	C11H14O
Z254586068	CCC1=C(SN=N1)C(=O)N(C)C	185,25	C7H11N3OS
Z1891701433	NCC1=NC(=CS1)C1=C(F)C=CC=C1	208,25	C10H9FN2S
Z2273353833	CCC1(CC1)NC1=NC(C)=NS1	183,27	C8H13N3S
Z1575304397	NCCNC1=NN=CC=C1	138,17	C6H10N4
Z1983132756		155,24	C9H17NO
Z1695921554	CN1C=C(C=N1)C(O)C(O)=O	156,14	C6H8N2O3
Z1381271509	CC1=CC(N2CCCC2)=C(CN)C=C1	190,29	C12H18N2
Z1699557311	CN1N=C(C)N=C1NCC1=NC=CC=C1	203,25	C10H13N5
Z1575311949	CN1C=C(N)C(C)=CC1=O	138,17	C7H10N2O
Z2091496785	CN(CC1=NC=CS1)C1=NC=C(F)C=C1	223,27	C10H10FN3S
Z1615132073	CN(CC(C)=C)C1=NC=C(F)C=N1	181,21	C9H12FN3
Z2256514859	CN(CC1=C(F)C=NC=C1)C1CCOC1	210,25	C11H15FN2O
Z1537215768	COC1=C(F)C(=CC=C1)C(=O)N(C)C	197,21	C10H12FNO2
Z2272040615	CC1(O)CC(C1)C(=O)N1CCC=C(F)C1	213,25	C11H16FNO2
Z1587272989	CC1=NOC(CN2CCC=C(F)C2)=N1	197,21	C9H12FN3O
Z1749360781	CC1=C(C)N(CC2=CC(F)=CN=C2)C=N1	205,24	C11H12FN3
Z2272040604		213,25	C11H16FNO2
Z2068674771	CC1CCCN(CC1)C(=O)COC(F)F	221,25	C10H17F2NO2
Z1517673547	CC1=CCN(CC1)C1=NC=C(F)C=N1	193,23	C10H12FN3
Z1973748497	CC1=CCCN(C1)C1=NC=C(F)C=N1	193,23	C10H12FN3
Z56347489	CN(C)C(CN)C1=CC(F)=CC=C1	182,24	C10H15FN2
Z435369470	CN(CC1=CC(Br)=CS1)C(=O)C1CCC1	288,20	C11H14BrNOS
Z1486705591	CS(=O)(=O)N1CCOCC11CCC1	205,27	C8H15NO3S
Z1188325591	CCN(C)C(=O)NCC1=CC(C)=CC=C1	206,29	C12H18N2O
Z940634048	CCS(=O)(=O)NC1COC2=C1C=CC=C2	227,28	C10H13NO3S
Z1506050651	CN(C(=O)[C@H]1CC1(C)C)C1=CN=CC=C1	204,27	C12H16N2O
Z2327040050	OC(=O)C1C=CC=CC=C1	136,15	C8H8O2
Z2293642517	CC1(CCCCO1)C(O)=O	144,17	C7H12O3
Z1263529842	FC(F)OC1=C(C=CC=C1)C#N	169,13	C8H5F2NO
Z89264959	CC1=NC2=C(C=C(N)C=C2)C(N)=C1	173,22	C10H11N3
Z85923113	CC1=C2CCCCC2=NC(N)=N1	163,22	C9H13N3
Z57899732	COC1=CC(=CC(OC)=C1OC)C#N	193,20	C10H11NO3
Z85923173	COC1=C(OC(F)F)C=CC(=C1)C#N	199,16	C9H7F2NO2
Z85932361	OC(=O)C1=CC2=C(OCCO2)C(Cl)=C1	214,60	C9H7ClO4
Z57984664	NC1=CC=NN1CC1=CC(Br)=CS1	258,14	C8H8BrN3S
Z26148273	NC(=O)CN1N=NC2=C(C=CC=C2)C1=O	204,19	C9H8N4O2
Z14772440	CNC(=O)COC1=C(Cl)C=CC(Cl)=C1	234,08	C9H9Cl2NO2
Z56347458	OC(=O)CN1C(=O)COC2=C1C=CC=C2	207,19	C10H9NO4
Z57029692	C[C@H](NC(=O)C1=CC(C)=CC=C1)C(O)=O	207,23	C11H13NO3
Z401537598	CCC1=NC(CC(O)=O)=CS1	171,21	C7H9NO2S
Z33547100	NC(=O)C1=CC2=C(OCCO2)C=C1	179,18	C9H9NO3
Z31407956	CC1=CC=C(S1)C(=O)NCC1=CC=CO1	221,27	C11H11NO2S
Z263785508	COCC(=O)NC1=NN=C(C)S1	187,22	C6H9N3O2S
Z752370760	CC(C)OC1=C(Cl)C=C(C=N1)C(O)=O	215,63	C9H10ClNO3
Z228589452	OC(=O)CC1=CSC(=N1)C1=CC=CO1	209,22	C9H7NO3S
Z321209398	CC(C)NC(=O)C1=NNC(=C1)C1CC1	193,25	C10H15N3O
Z235449228	COC1=CC(=CC=C1)C1=NC(CO)=CS1	221,27	C11H11NO2S
Z33546492	COC1=CC=C(C=C1)C(N)=O	151,17	C8H9NO2
Z30173060	O=C(NCC1=CN=CC=C1)C1=CC=CS1	218,27	C11H10N2OS

Z345089918	CC1=C(CNC(=O)C2=CC=CO2)SC=C1	221,27	C11H11NO2S
Z50147461	CC1=NN(C(=O)C1)C1=C(C)C=CC(C)=C1	202,26	C12H14N2O
Z235342005	NCC1(CCCCC1)NC1=CC=CC=C1	204,32	C13H20N2
Z235338709	O=C1N(N=NC2=C1C=CC=C2)C1CC1	187,20	C10H9N3O
Z30166515	O=C(NCC1=NC=CC=C1)C1=CC=CS1	218,27	C11H10N2OS
Z332659786	CNC(=O)C1=NNC(=C1)C1=CC=CO1	191,19	C9H9N3O2
Z2469606504	CN1C=NC2=C1N=CNC2=O	150,14	C6H6N4O
Z1551700540	CC(C)C1=CC(NC(=O)CO)=CC=C1	193,25	C11H15NO2
Z2442033754	NC1=C(F)C=CC(=C1)N1CCNC1=O	195,20	C9H10FN3O
Z2429425678	CC1CCN2N=CC(C(O)=O)=C2C1	180,21	C9H12N2O2
Z2429425504	CC1=CC=C(C=C1)N1C=CNC1=O	174,20	C10H10N2O
Z2429425500	CC1=NC(CCO)=CN=C1	138,17	C7H10N2O
Z2429425528	OCC1CNC2=C(C1)C=CC=C2F	181,21	C10H12FNO
Z2418194206	OC1CCCN2C=CN=C12	138,17	C7H10N2O
Z2412196557	CC1=C(C)N=C(C)C(=O)N1	138,17	C7H10N2O
Z137992830	CCN1CCCCC1CO	143,23	C8H17NO
Z1263529734	C(C1CCCCN1)N1CCOCC1	184,28	C10H20N2O
Z1443588951	CC(C(O)=O)C1=CCCC1	140,18	C8H12O2
Z1374788709	CN(C)C1=C(CN)C=CC(C)=C1	164,25	C10H16N2
Z1250132777	CC(N)CC1=C(C)ON=C1C	154,21	C8H14N2O
Z1272672332	CC(CN)C1=C(F)C=C(F)C=C1	171,19	C9H11F2N
Z1272728880	CC(C)C(N)CC1=CC=C(F)C=C1	181,25	C11H16FN
Z1263811754	COC1=C(CC(C)(C)N)C=CC=C1	179,26	C11H17NO
Z1362754425	CC(O)CN1C=CN=C1C	140,19	C7H12N2O
Z1362754408	NC1CCCN2N=CN=C12	138,17	C6H10N4
Z1381287341	NC1CCN(C1)C1=C(F)C=CC=C1F	198,22	C10H12F2N2
Z1350579567	COC1CCC(CN)CC1	143,23	C8H17NO
Z1346370676	CCC1=CN=C(S1)C(C)N	156,25	C7H12N2S
Z1407659697	CCC1CCC(O1)C(O)=O	144,17	C7H12O3
Z1176666054	CCN1CCC(CC1)C(C)O	157,26	C9H19NO
Z89271885	CCS(=O)(=O)C1=CC2=C(OC(N)=N2)C=C1	226,25	C9H10N2O3S
Z106805790	CCC1N(CC(N)=O)CCC2=C1C=CS2	224,32	C11H16N2OS
Z168883434	CNS(=O)(=O)C1=CN(C)C(=C1)C(O)=O	218,23	C7H10N2O4S
Z223688278	CNC(C(=O)NC)C1=CC=C(Cl)C=C1	212,68	C10H13ClN2O
Z228474590	CN1C(=O)N(CC(O)=O)C2=C1C=CC=C2	206,20	C10H10N2O3
Z119993908	CC1=C(CC(O)=O)C(C)=NC(=O)N1	182,18	C8H10N2O3
Z104341874	CC1=CC2=C(C=CC=C2)N1CC(O)=O	189,21	C11H11NO2
Z56824601	OC(=O)COC1=C(C=CC=C1)C#N	177,16	C9H7NO3
Z1250083006	OC(=O)C1=C2OCCOC2=CC=C1	180,16	C9H8O4
Z1250080927	CS(=O)(=O)C1(CN)CCOCC1	193,26	C7H15NO3S
Z1238477790	CC(NC(=O)C1CCCC1)C(O)=O	185,22	C9H15NO3
Z2469657345	CS(=O)(=O)C1=CC2=C(NC=C2)C=C1	195,24	C9H9NO2S
Z1222423406	CC1=CC=C(O1)C1=NC(N)=NN=C1	176,18	C8H8N4O
Z1429750050	COC1=C2C=CC=NC2=CC=C1	159,19	C10H9NO
Z1262395976	CC1=C(C=C(Cl)C=C1)N1CCC(CN)C1	224,73	C12H17ClN2
Z1267885772	CC1CN(CCN1)C1=NC(C)=CC=C1	191,28	C11H17N3
Z2235791789	CC1CNCCC1CCC(O)=O	171,24	C9H17NO2
Z2240999272	COC1=CC2=C(N=CC=C2)C(OC)=C1	189,21	C11H11NO2
Z1741964335	C1CNC(CN1)C1=CC=CC=C1	162,24	C10H14N2
Z2238934986	COC1=NC=C(N=C1)C#N	135,13	C6H5N3O
Z1720655573	CC(C)C1=C(C=CC(C)=N1)C(O)=O	179,22	C10H13NO2
Z1198183883	NC1=NC2=C(CCC2)C=C1	134,18	C8H10N2
Z2179938347	CN1N=CC(CCC(O)=O)=N1	155,16	C6H9N3O2
Z2235810122	CC1=CN=C2C=C(C=CN12)C(O)=O	176,18	C9H8N2O2
Z2235810219	CC1(C)CCC2=C(C1)C(O)=NC=N2	178,24	C10H14N2O
Z1263529593		177,25	C11H15NO
Z1983132695	BrC1=C2OCCNC2=CC=C1	214,06	C8H8BrNO
Z1103034534	CC1=CC=CN(CC2=CC=C(Cl)S2)C1=O	239,72	C11H10ClNOS
Z1250132460	CC(C)N1C=CC(CN)=N1	139,20	C7H13N3
Z606312288	CN(C)C(=O)CN1C=C(Br)C=N1	232,08	C7H10BrN3O
Z1245646665	OC(C1=CC=C(Cl)C=C1)C1=CN=CC=C1	219,67	C12H10ClNO
Z1245633392	CC(O)C1=CC2=C(OCC2)C=C1	164,20	C10H12O2
Z56822177	OC(=O)C1CC2=C(CN1)C=CC=C2	177,20	C10H11NO2
Z1230795916	CC(C)CNC(=O)C1=NC=CN1C	181,24	C9H15N3O
Z1983132706	C1CC2(CO1)COCCN2	143,19	C7H13NO2
Z32803522	CCC(=O)NC1=CC(CO)=CC=C1	179,22	C10H13NO2
Z1729207294	ClC1=C(CCN2C=CN=N2)C=CC=C1	207,66	C10H10ClN3

Z1269702508	CC(N)C1=C(C)C(C)=CC=C1	149,24	C10H15N
Z105197348	CC(N)C1=NC2=C(S1)C=CC=C2	178,25	C9H10N2S
Z803153754	NCC1=CC(=NC=C1)N1CCOCC1	193,25	C10H15N3O
Z1895554285	CC(CCO)C1=CNC2=C1C=CC=C2	189,26	C12H15NO
Z1655103965	CN(C)C1=C(N)N=CC=C1	137,19	C7H11N3
Z1696822333	OC(=O)C1CCCN1C1CCNC1	184,24	C9H16N2O2
Z1998612646	C1CNC(CN1)C1=CN=CC=C1	163,22	C9H13N3
Z1230248368	CN(CCO)C(=O)NC1=C(Br)C=CC=C1	273,13	C10H13BrN2O2
Z1993522703	CN1C(=O)CC2=C1C=CC(Cl)=C2	181,62	C9H8ClNO
Z383202616	CC(=O)NC1=CC2=NNC=C2C=C1	175,19	C9H9N3O
Z2028214360	OC(=O)C[C@@H]1C[C@H]1C1=CC=CC=C1	176,22	C11H12O2
Z2027915114	CC(C)C1=NNC(=N1)C(F)F	161,16	C6H9F2N3
Z2027915113	CC1=NN(C(F)F)C(C)=C1CO	176,17	C7H10F2N2O
Z1896598013	CCC1=CC(=O)N2N=C(NC)SC2=N1	210,26	C8H10N4OS
Z1899648075	O=C1N(CC2CC2)C=CC2=C1C=NC=N2	201,23	C11H11N3O
Z1623872052	CC(C)C(O)C1(CN)CC1	143,23	C8H17NO
Z103698666	CC(N1CCCCC1)C(N)=O	156,23	C8H16N2O
Z1582262983	CCC1=CSC(=N1)N1C=C(C=N1)C(O)=O	223,25	C9H9N3O2S
Z1575311614	NC1CCS(=O)(=O)C2=C1C=C(Cl)C=C2	231,69	C9H10ClNO2S
Z1575304427	CC1=NN(CC(O)=O)C2=C1C=CC=N2	191,19	C9H9N3O2
Z1569709055	CCC1=C(C=C(C=C1)C(O)=O)S(C)(=O)=O	228,26	C10H12O4S
Z1569709199	CC1=NNC2=C1C=CC(N)=C2	147,18	C8H9N3
Z1551777829	CCS(=O)(=O)N1CCN(CC1)C(=O)CO	236,29	C8H16N2O4S
Z1642389710	CC1=C(F)C=CC(C(O)=O)=C1N	169,16	C8H8FNO2
Z1642389640	NC1=CC2=C(CCCC2)N=C1	148,21	C9H12N2
Z1642389487	CN(C)C1=C(C=CN=C1)C#N	147,18	C8H9N3
Z755044716	CCNC(=O)C1=CNN=N1	140,15	C5H8N4O
Z1119758873	OCC1CCN(CC2=NC=CC=C2)CC1	206,29	C12H18N2O
Z1262396026	CC(N)C1=CC(Br)=CC=C1	200,08	C8H10BrN
Z1981623627	CN(C(=O)C1CCC1)C1=NC=CN=C1	191,23	C10H13N3O
Z1702887672	CC1CN(C1)C(=O)NC1=NC=CC=C1	191,23	C10H13N3O
Z1463254676	NCC1=C2OCCCOC2=CC=C1	179,22	C10H13NO2
Z1575308472	CC1=C(CCN)C=NC=C1	136,20	C8H12N2
Z1695867452	CC1=NC(C)=NC(=C1)N1CCC(N)CC1	206,29	C11H18N4
Z1689341639	CC1=C(Br)C=C(C(O)=O)C(=O)N1	232,03	C7H6BrNO3
Z2054764431	C[C@H]1OCCN(CC=C(C)C)[C@@H]1C(N)=O	212,29	C11H20N2O2
Z2235790650	COC1=C(C=C(Cl)C=C1)C(O)CN	201,65	C9H12ClNO2
Z2235681415		176,22	C11H12O2
Z2235681413	CC1(C)CNC2=C(O1)C=CC=N2	164,21	C9H12N2O
Z1939263633	NC(=O)COC1=C2CCCNC2=CC=C1	206,25	C11H14N2O2
Z2235409161	NC1=NC=CC(CCO)=C1	138,17	C7H10N2O
Z2235409319	COC1=NC=NC(=C1)C#N	135,13	C6H5N3O
Z2235330321	NCC1CS(=O)(=O)C2=C1C=CC=C2	197,25	C9H11NO2S
Z85879488	OC(=O)C1=C(NC(=O)C2CC2)C=CC=C1	205,21	C11H11NO3
Z2217033053	CN1N=CC=C1[C@H]1[C@H](N)CCC(=O)N1C	208,27	C10H16N4O
Z2106594228	OC(=O)C1=C(Br)C=NN1C1CCCC1	259,10	C9H11BrN2O2
Z1562205518	NC(=O)C1=CN(CCO)N=C1	155,16	C6H9N3O2
Z1516516959	CC(C)(O)CN1C=C(Br)C=N1	219,08	C7H11BrN2O
Z57952782	CC1CC2=C(O1)C=CC(=C2)C(O)=O	178,19	C10H10O3
Z2568758085	CN1C=C(C=N1)N1CCC(CO)C1	181,24	C9H15N3O
Z2568758079	CC1=CC2=C(C=C1)C(N)=NC=C2	158,20	C10H10N2
Z2522940202	CC1=CCC(C)(CC1)C(O)=O	154,21	C9H14O2
Z2517219346	CN1CCC2=C(C=CC(N)=C2)C1=O	176,22	C10H12N2O
Z1508967947		208,21	C11H12O4
Z1993611196	Cl.CNCC(=O)NC1=NC=CC(C)=C1	215,68	C9H14ClN3O
Z2515207540	COC1=CC=C(C=C1)C1=NC(N)=NO1	191,19	C9H9N3O2
Z345556840	CC(=O)NCC1=C(Cl)C=CC=C1Cl	218,08	C9H9Cl2NO
Z228585484	OCC(=O)NC1=CC=C(Cl)C=C1	185,61	C8H8ClNO2
Z1741967387	NC(CC(O)=O)C1=CC=C(Br)C=C1	244,09	C9H10BrNO2
Z54745174	CNC1=NSC2=C1C=CC=C2	164,23	C8H8N2S
Z2513337538	OCCC1=C2C=CC=CC2=NC=C1	173,22	C11H11NO
Z1889909750	CC(C)C1OCCCC1C(O)=O	172,22	C9H16O3
Z166605304	CC1=NC2=NN=C(CN)N2C(C)=C1	177,21	C8H11N5
Z57983005	OC(=O)CC1OC2=C(NC1=O)C=CC=C2	207,19	C10H9NO4
Z85917495	OC(=O)CC1=CSC(=N1)C1=NC=CC=C1	220,25	C10H8N2O2S
Z56945486	OC(=O)CNC(=O)C1=C(Br)C=CC=C1	258,07	C9H8BrNO3
Z56939169	CC1=C(C(O)=O)C(C)=NO1	141,13	C6H7NO3

Z56886259	OC(=O)CNC(=O)C1=CC=CS1	185,20	C7H7NO3S
Z56921163	NC(CC(O)=O)C1=CC=C(F)C=C1	183,18	C9H10FNO2
Z56914816	CCOC1=CC=C(C=C1)C1=NN=C(N)O1	205,22	C10H11N3O2
Z56862696	CC(=O)NC1=CC(OC(F)F)=C(Br)C=C1	280,07	C9H8BrF2NO2
Z30802725	FC1=CC(F)=C(NC(=O)C2CCC2)C=C1	211,21	C11H11F2NO
Z56827184	CCOC1=C(Br)C=C(NC(C)=O)C=C1	258,12	C10H12BrNO2
Z56827160	OC(=O)CNC(=O)C1=C(Cl)C=CC=C1	213,62	C9H8ClNO3
Z56795887	CC1=CC(=O)C2=NN=CN2N1	150,14	C6H6N4O
Z234969411	CC1=NN(CCO)C(C)=C1C(O)=O	184,20	C8H12N2O3
Z56757135	NC(=O)C12CCC(CC1)C2	139,20	C8H13NO
Z1431905456	CCC(O)(C(N)=O)C1=CC=C(Br)C=C1	258,12	C10H12BrNO2
Z1428159818	OC(=O)C1CCNC(=O)C1	143,14	C6H9NO3
Z1416282423	CC1=C(CS(C)(=O)=O)C=C(S1)C(O)=O	234,28	C8H10O4S2
Z1316699693	CC1=CC(C)=NC(NCC2(O)CCC2)=N1	207,28	C11H17N3O
Z1416200910	COC1=C(OC)C=C(C(O)=O)C(F)=C1	200,17	C9H9FO4
Z1416200891	COC1=CC(N)=C(C)C=C1	137,18	C8H11NO
Z1316700062	CC1=NC(C)=NC(NCC2(O)CCC2)=C1	207,28	C11H17N3O
Z1270081818	CC1(CO)CCCC1NC1=NC=CC=N1	207,28	C11H17N3O
Z166605426	CN1C(C)=CC=C1C(O)=O	139,15	C7H9NO2
Z1374884282	COC1=NC2=C(O1)C=CC(N)=C2	164,16	C8H8N2O2
Z1210869358	NC1CCC(CC1)C1=CC=CC=C1	175,28	C12H17N
Z140561750	CNC(C)C1=C(OC)C=CC=C1	165,24	C10H15NO
Z1259161793	CC1=CC=C(O1)C(C)(O)CN	155,20	C8H13NO2
Z927412230	CC1CCCCN1C(=O)CCN	170,26	C9H18N2O
Z1162910733	NCC1CCCN1CC1CCCCC1	196,34	C12H24N2
Z1318357717	CCOC1=NC(N)=CC=C1	138,17	C7H10N2O
Z234897649	NCC1=CC(=NC=C1)N1CCCCCC1	205,31	C12H19N3
Z369045030	CCC1=NC(CN2CCOCC2C)=CS1	226,34	C11H18N2OS
Z55002275	N#CC1=C(CN2CCCC2)C=CC=C1	186,26	C12H14N2
Z1343332730	CN(CC1CCOC1)C1=NC=C(Br)C=N1	272,15	C10H14BrN3O
Z2234631223	CC1=C(C=C(C=C1)C(O)=O)C(O)=O	180,16	C9H8O4
Z1222285084	COC1CCN(CC1)C1CCCCC1N	212,34	C12H24N2O
Z2130657289	CC(C)OC1=C(CO)C=NC=C1	167,21	C9H13NO2
Z1899842917	CC1=NN(CC2=C(C)ON=C2C)C(=O)S1	225,27	C9H11N3O2S
Z229424542	C1CC2=C(CN1C1=NC=CC=N1)C=CS2	217,29	C11H11N3S
Z2037257299	OCC1CCCS(=O)(=O)C1	164,22	C6H12O3S
Z1841067347	OC1CCCC1NCC1=CC=C(Br)O1	260,13	C10H14BrNO2
Z1787761777	COC1CN(CC2=CN=CS2)CCC1C	226,34	C11H18N2OS
Z1899855407	CCC1=NC(CN2N=C(C)SC2=O)=CS1	241,33	C9H11N3OS2
Z2028236283	CN(C)CC(N)C1=CC=C(Cl)C=C1	198,69	C10H15ClN2
Z2010052046	COCC1(CCC1)C(O)=O	144,17	C7H12O3
Z33546677	NC(=O)CC1=CC=C(Cl)C=C1	169,61	C8H8ClNO
Z370745502	CNC(=O)C1=CC=C(O1)S(=O)(=O)N(C)C	232,25	C8H12N2O4S
Z242435796	FC1=CC=CC(F)=C1C(=O)NCC=C	197,19	C10H9F2NO
Z1101435050	CN1C=C(CC(O)=O)C=N1	140,14	C6H8N2O2
Z54128519	CCC(C)(C)NC(=O)C1CC1	155,24	C9H17NO
Z441756998	CCC(C)(C)NC(=O)C1=NNC=C1	181,24	C9H15N3O
Z409510704	CC1CN(CCO1)C(=O)NC1=NN=CS1	228,27	C8H12N4O2S
Z285675388	CNC(=O)C1=CN=C(C=C1)N1CCCC1	205,26	C11H15N3O
Z332751146	CN(C)C(=O)C1=NNC(=C1)C1CC1	179,22	C9H13N3O
Z45462907	O=C1CN(CCN1)S(=O)(=O)C1=CC=CS1	246,30	C8H10N2O3S2
Z740611958	CC(C)C(=O)NC(C)C1=C(C)N=C(C)S1	226,34	C11H18N2OS
Z853055300	CC1(C)COCCN1C(=O)C1=NSN=C1	227,28	C9H13N3O2S
Z1272949026	CCOC1=C(C=CC=C1F)C(O)=O	184,17	C9H9FO3
Z1271843096	CC(C)C1CCNC(=O)CC1	155,24	C9H17NO
Z734736324	CCC(C)(C)NC(=O)C1=CC(C)=NN1C	209,29	C11H19N3O
Z1318356930	CC1=CC(CC(O)=O)=C(C)S1	170,23	C8H10O2S
Z1318356922	CC(C1=CC=CC=C1)C(C)(O)C(O)=O	194,23	C11H14O3
Z1310750949	NC1(CCOCC1)C(O)=O	145,16	C6H11NO3
Z274554320	CC(CN1N=C(C)C=C1C)C(O)=O	182,22	C9H14N2O2
Z274554370	CC(CC(O)=O)N1N=C(C)C(Br)=C1C	261,12	C9H13BrN2O2
Z274554330	CC(CN1C=NC=N1)C(O)=O	155,16	C6H9N3O2
Z1281658025	OC(=O)CCC1=NC2=C(S1)C=CC(Br)=C2	286,14	C10H8BrNO2S
Z56324724	CN(C)C(=O)NC1=CC(Cl)=C(C)C=C1	212,68	C10H13ClN2O
Z1921389504	CC(=C)CN1CC(O)CC2=C1C=CC=C2	203,29	C13H17NO
Z1481101458	FC1=CN=C(NC2CC2)N=C1	153,16	C7H8FN3
Z2219513504	CC1CN(CC2=C(C)N(C)N=C2C)C1C	207,32	C12H21N3

Z1250132461	CCC(N)C1=C(F)C=C(F)C=C1	171,19	C9H11F2N
Z1222285079	COC1=CC(=CC=C1)N1CCC(CN)C1	206,29	C12H18N2O
Z234897221	NCC1=CN=C(OC2CCCCC2)C=C1	206,29	C12H18N2O
Z1272480091	COCC1CN(CCO1)C1=NC=CC=C1	208,26	C11H16N2O2
Z1262395911	COC1=NC(CN)=CC=C1	138,17	C7H10N2O
Z1333043394	CNCC1=NC2=C(CCCC2)S1	182,29	C9H14N2S
Z1224460119	OCCNC1CCCC2=C1C=CC=C2	191,27	C12H17NO
Z1614721070	OC(=O)C1(CC1)C(=O)NC1CCCC=C1	209,25	C11H15NO3
Z1918366445	FC1=C(Cl)C=CC(NCC2=NNC=C2)=C1	225,65	C10H9ClFN3
Z2065464240	CC1=NOC(CN2N=NC(N)=C2C)=C1	193,21	C8H11N5O
Z32367965	FC1=C(CC(=O)NC2CC2)C(Cl)=CC=C1	227,66	C11H11ClFNO
Z1848951744	FC1=C(NCC2=CN=CS2)C=CN=C1	209,24	C9H8FN3S
Z1983199026	COC(C)(C)C(=O)NC1=NC(C)=CC=N1	209,25	C10H15N3O2
Z1796281450	CN(C)CC(=O)NC1=C(F)C=CC=C1C	210,25	C11H15FN2O
Z1791179060	CC1=C(OC=C1)C(=O)NC1=CN=NS1	209,22	C8H7N3O2S
Z1270403514	OC(=O)C1CCS(=O)(=O)CC1	178,20	C6H10O4S
Z414834498	OC(=O)[C@H]1C[C@H](C1)C(O)=O	144,13	C6H8O4
Z57157168	CC1=CCC(C(C1)C(O)=O)C(O)=O	184,19	C9H12O4
Z85887020	OC(=O)CNC(=O)C1=CC2=C(CCC2)S1	225,26	C10H11NO3S
Z959151608	O[C@@H]1CCOC2=C1C=CC=C2F	168,17	C9H9FO2
Z1020680168	NCC1(O)CCCCCC1	143,23	C8H17NO
Z1268152304	O=S1CCCNC2=C1C=CC=C2	181,25	C9H11NOS
Z29672824	CC1=NN=C(NC(=O)C2=CC=CO2)S1	209,22	C8H7N3O2S
Z927306976	NCCNC(=O)C1=NC=CN=C1	166,18	C7H10N4O
Z1262246158	CC(C)N1N=CC2=C1C=C(N)C=C2	175,24	C10H13N3
Z1262246089	CC1=C2NC=C(C(O)=O)C(=O)C2=CC=C1	203,20	C11H9NO3
Z220383506	NC(=O)CC1=C(Cl)C=C(C=N1)C(F)(F)F	238,59	C8H6ClF3N2O
Z2658865866	COC1=CC2=C(C(=O)C=CN2)C(OC)=C1	205,21	C11H11NO3
Z846981274	CC1=CC(CS(C)(=O)=O)=C(C)S1	204,30	C8H12O2S2
Z2106591704	OC(=O)C1=CC=C(CN2C=CC=N2)S1	208,24	C9H8N2O2S
Z2106593035	OC(=O)CN1C=CC(=N1)C1CC1	166,18	C8H10N2O2
Z2060348232	CN(CC(O)=O)C1=C(F)C=C(C=C1)C#N	208,19	C10H9FN2O2
Z2106601348	CC(C)N1C=C(C(O)=O)C(C)=N1	168,20	C8H12N2O2
Z2510258132	COC1=CC2=C(CCC2C(O)=O)C=C1	192,21	C11H12O3
Z2412196567	CCOC1=NC(N)=CN=C1	139,16	C6H9N3O
Z1624945782	CC(=CC1=CC=C(Cl)S1)C1=NC=CN=C1	236,72	C11H9ClN2S
Z1217960891	CN1CCC(OC2=CC(F)=CC=C2)C1=O	209,22	C11H12FNO2
Z2379802745		191,23	C11H13NO2
Z2379802683	CN1N=C(N)C2=C1C=C(C)N(C)C2=O	192,22	C9H12N4O
Z1413929716	OCCNC1=CC(F)=C(F)C=C1	173,16	C8H9F2NO
Z2379087651	OC(=O)CC1=CC=C(C=C1)N1C=NN=C1	203,20	C10H9N3O2
Z275169862	CN1N=C(C(O)=O)C(Cl)=C1C	174,58	C6H7ClN2O2
Z2346081588	CC(C)(O)C1=C(N)C=C2C=CC=CC2=N1	202,26	C12H14N2O
Z2327883945	O=S1(=O)CCCN1C1CCNCC1	204,29	C8H16N2O2S
Z2327883755	CCN1C(=O)C(O)=NC2=C1C=CC=C2	190,20	C10H10N2O2
Z2327883839	CC1=CSC2=C1N=CN=C2	150,20	C7H6N2S
Z111808714	CC1=NC2=C(C=CC=C2N)C=C1	158,20	C10H10N2
Z227823010	CCNC1=NC=CC(=C1)C(O)=O	166,18	C8H10N2O2
Z1262398568	CC(=O)N1CCCNC2=C1C=CC=C2	190,25	C11H14N2O
Z28134680	CC(=O)NC1=CC=C(C)C=C1	149,19	C9H11NO
Z1697079442	CC1=NC=NC(=C1)N1CCC=C(F)C1	193,23	C10H12FN3
Z1262327470	CNS(=O)(=O)C1=C(C)C=C(N)C=C1	200,26	C8H12N2O2S
Z1262327418	CC1CNC(=O)N1C1=CC(N)=CC=C1	191,23	C10H13N3O
Z1262254339	OC(C1CCCCN1)C1=CC=CC=C1	191,27	C12H17NO
Z1259339084	CN1N=CC=C1C1CCNCC1	165,24	C9H15N3
Z1262252941	NCC1=C(F)C=C(F)C=C1	143,14	C7H7F2N
Z1272267454	CC1=NSC(=N1)N1CCC[C@@H]1CO	199,27	C8H13N3OS
Z1836338670	CC1NCCN2C=NN=C12	138,17	C6H10N4
Z1827898544	CCC1=C(N)C=NN1C1CCCC1	179,27	C10H17N3
Z1250132437	OC(=O)CCC1CCOC1	144,17	C7H12O3
Z1823262976	CCNC1=CC2=C(NC=C2)C=C1	160,22	C10H12N2
Z1815149220	CCC(C)N1N=CC(N)=C1C1CC1	179,27	C10H17N3
Z1516316257	CCC1=NOC(C)=C1CN1C=CC(C)=N1	205,26	C11H15N3O
Z1741963038	CC1=C(CN)C=C(Cl)C=C1	155,63	C8H10ClN
Z1801361876	C1COC(C1)C1CCNCC1	155,24	C9H17NO
Z1820007381	CC(C)N1N=CC(C(O)=O)=C1N	169,18	C7H11N3O2
Z1891775929	OC1CCCNC2=C1C=C(F)C=C2	181,21	C10H12FNO

Z2242946524	NC1=C(Br)C=C2CNCC2=C1	213,08	C8H9BrN2
Z1893446212	CC1=C(N)ON=C1C1=C(F)C=CC(C)=C1	206,22	C11H11FN2O
Z1889917194	OC(=O)C1CCCN1C1=CC=CC=C1	191,23	C11H13NO2
Z1837073492	CC1=C(CO)C=CC(=C1)N1C=CN=C1	188,23	C11H12N2O
Z1723633687	CC1=C(C=C(F)C=C1)C1=NN=C(N)O1	193,18	C9H8FN3O
Z2226312235	CC1=NOC=C1CN1CCC=C(F)C1	196,23	C10H13FN2O
Z2329283105	OC1(CCN2CCC=C(F)C2)CCC1	199,27	C11H18FNO
Z1619984629	CN1CCC(N2CCC=C(F)C2)C1=O	198,24	C10H15FN2O
Z2034487295	CN(CC1CCOC1)C1=NC=C(F)C=C1	210,25	C11H15FN2O
Z1929757385	CC1(C)CN(CC1O)C1=C(F)C=CC=N1	210,25	C11H15FN2O
Z1748646799	C[C@H](NC(=O)CO)C1=C(Cl)C=CC(Cl)=C1	248,10	C10H11Cl2NO2
Z1675358685	CN(CC1(O)CCC1)C1=C(Cl)C=NC=C1	226,70	C11H15ClN2O
Z1985112155	CCN(C(=O)C1CC1)C1=C(C)C=CC=N1	204,27	C12H16N2O
Z1463350841	CC(C)CN1C=CN=C1N	139,20	C7H13N3
Z2087745225		205,30	C13H19NO
Z2092579816	CC(N)CN(C)C1CCN(C)CC1	185,32	C10H23N3
Z2442044577	OCC(F)=C1CCCNC1	145,18	C7H12FNO
Z1348559502	CNC1(CCCC1)C(N)=O	142,20	C7H14N2O
Z1526503471	CC1=C(C)N=C(C(O)=O)C(=O)N1	168,15	C7H8N2O3
Z1501469698	CC(C)C1=C(N)N=CC=C1	136,20	C8H12N2
Z1333043520	CN1C(=O)CNC2=C1C=CC=C2	162,19	C9H10N2O
Z1492781233	CC(C)(C1=NOC(N)=C1)S(C)(=O)=O	204,24	C7H12N2O3S
Z1480642142	COC1(CN)CCOCC1	145,20	C7H15NO2
Z1459946056	COCC1=NC=NC(O)=C1	140,14	C6H8N2O2
Z57990849	OC(=O)CCC1=NC2=C(O1)C=CC=C2	191,19	C10H9NO3
Z1442018040	CC1=C(C(O)=O)C(N)=C(Cl)C=C1	185,61	C8H8ClNO2
Z1436472922	COCC1=NC=NC(N)=C1	139,16	C6H9N3O
Z1436105147	CN1NC(=O)C2=C1NC=CC2=O	165,15	C7H7N3O2
Z274779140	CC(CN)OC1=CC=C(F)C=C1	169,20	C9H12FNO
Z1428019338	CCC1CN(CCC1N)S(C)(=O)=O	206,30	C8H18N2O2S
Z1266823391	COCCN1CCNCC1C	158,25	C8H18N2O
Z1342884092	CC(C1CCCC1)C(O)=O	142,20	C8H14O2
Z1222285092	COC1=C(C=CC=C1)N1CCC(N)CC1	206,29	C12H18N2O
Z1238477839	CC(N)CN1CCCCC1	142,25	C8H18N2
Z1263529706	COC1=C2OC(CN)=CC2=CC=C1	177,20	C10H11NO2
Z1262237458	NC(C1CC1)C1=NC=CS1	154,23	C7H10N2S
Z454097862	CC(C)NC(=O)C1=NNC(=C1)C(C)C	195,27	C10H17N3O
Z1455009926	CC1=C(Cl)C=CC(=C1)C(=O)NCC(O)=O	227,64	C10H10ClNO3
Z647156496	CC(O)CNC(=O)C1=C(C)C=C(Br)C=C1	272,14	C11H14BrNO2
Z1741975280	CC1=NC(N)=C(CO)C=N1	139,16	C6H9N3O
Z1945695955	OC(=O)CCC1=CC2=C(C=CC=C2)N=C1	201,23	C12H11NO2
Z1273312303	CCNC(=O)C1=CC(Br)=CN1C(C)C	259,15	C10H15BrN2O
Z1934400485	NCC1=NN=C(S1)C1=C(F)C=NC=C1	210,23	C8H7FN4S
Z1699521115	CN1N=CN=C1NCC1=C(C)N=C(C)S1	223,30	C9H13N5S
Z1896597109	CNC1=NC=C(N=C1)C#N	134,14	C6H6N4
Z1901071385	CN(C)C1=NC=C(N=C1)C#N	148,17	C7H8N4
Z1783879420	N#CC1=CSC(CNC2=NC=CC=N2)=C1	216,26	C10H8N4S
Z1895582742		202,65	C8H7ClO2S
Z744593250	OC(=O)C1=C2NCCCN2N=C1	167,17	C7H9N3O2
Z56763896	OC(CN1C=NC=N1)C1=CC=C(Cl)C=C1	223,66	C10H10ClN3O
Z373775846	CN1C=C(NC(=O)C2=CC=C(C)S2)C=N1	221,28	C10H11N3OS
Z372503386	CCC1=CC=C(O1)C(=O)NC1=CNN=C1	205,22	C10H11N3O2
Z145545076	BrC1=CNC(=C1)C(=O)N1CCNC(=O)C1	272,10	C9H10BrN3O2
Z57074586	CC(NC(=O)C1=CC=C(Br)O1)C(O)=O	262,06	C8H8BrNO4
Z744754540	CN1CC(=O)NC2=C1C=CC(=C2)C(O)=O	206,20	C10H10N2O3
Z228586716	O=C(NCC1=CC=CS1)C1CCNCC1	224,32	C11H16N2OS
Z1259339138	CNC1CCN(CC2=CC=NC=C2)CC1	205,31	C12H19N3
Z1258620051	CC1=C2NC=C(CCN)C2=CC=C1	174,25	C11H14N2
Z228581550	CC1=NN(C=C1C(O)=O)C1=CC=CC=C1	202,21	C11H10N2O2
Z1170039578	CC1CCC2=C(C1)C=C(C#N)C(=O)N2	188,23	C11H12N2O
Z955526726	CNC(=O)C1CC1C1=CC(Cl)=CC=C1	209,67	C11H12ClNO
Z228584312	OCC1=NC2=C(S1)C=CC(=C2)C(F)(F)F	233,21	C9H6F3NOS
Z1171978821	OC(=O)CN1N=CC2=C1C=CC=C2	176,18	C9H8N2O2
Z432066794	NC1=NN=C(O1)C1CCNCC1	168,20	C7H12N4O
Z224841742	NC1=C(N=CC=N1)C(=O)NC1CCCC1	206,25	C10H14N4O
Z274553586	CC1=NN(CCC(O)=O)C(C)=N1	169,18	C7H11N3O2
Z2588063830	CCOC1=CC(CO)=CN=C1	153,18	C8H11NO2

Z751928604	CC1=NNC=C1CNC1=CC(F)=CC=C1	205,24	C11H12FN3
Z1925944096	CC(C)CC1=C(N)ON=C1C(F)(F)F	208,18	C8H11F3N2O
Z1324812698	CC(=O)NCC1=NOC=C1	140,14	C6H8N2O2
Z2583036115	COC1CCC(C1)C(O)=O	144,17	C7H12O3
Z2568771122	C=CCOC1=CN=CC=C1	135,17	C8H9NO
Z2188253056	C[C@@H](O)CN(C)C(=O)C1=NC(C)=CC=C1	208,26	C11H16N2O2
Z228586320	CCC(O)C1=CC=C(OC)C=C1	166,22	C10H14O2
Z2216303091	OC(=O)COC1=CC(F)=C(Cl)C=C1	204,58	C8H6ClFO3
Z2211231664	COC1=C2C(CCC2=CC=C1)C(O)=O	192,21	C11H12O3
Z2001336017	CC1(C)CNC(=O)CN1S(=O)(=O)C1CC1	232,30	C9H16N2O3S
Z2205958778	CC1=C(SC2=C1C=C(C=C2)C#N)C(O)=O	217,24	C11H7NO2S
Z2189283988	OC(=O)CCC1=CN=CN=C1	152,15	C7H8N2O2
Z2188172118	OC(=O)CC1=CCOCC1	142,15	C7H10O3
Z1952438221	CC(NC(=O)C1=C(Br)C=CC=N1)C=C	255,12	C10H11BrN2O
Z2168541814	CC(N1CC(C)C1)C(O)=O	143,19	C7H13NO2
Z2168541836	CN1C(=O)CC2=C1C=CC(C)=C2	161,20	C10H11NO
Z2168499534	CS(=O)(=O)[C@@H]1CCCC[C@H]1O	178,25	C7H14O3S
Z2168282749	OC(=O)C1CCN(C1)C1=C(F)C=CC=C1	209,22	C11H12FNO2
Z183465912	CC(C)CNC(=O)C1=C(O)N=CC=C1	194,23	C10H14N2O2
Z108544480	CN1C=CC(NC(=O)C2=CC=C(C)S2)=N1	221,28	C10H11N3OS
Z28139094	COC1=CC=C(NC(=O)C2CC2C)C=C1	205,26	C12H15NO2
Z30612266	CCC(=O)NC1=CC=C(C=C1)C#N	174,20	C10H10N2O
Z324552980	Cl.CC1=CC(CN2CCNCC2)=NO1	217,70	C9H16ClN3O
Z104586966	CC(NC1=CC=C(Br)C=C1)C(N)=O	243,10	C9H11BrN2O
Z317046464	NC1=C(C=CC=C1)S(=O)(=O)N1CCCC1	226,29	C10H14N2O2S
Z228589506	CC1=CN2C(C=C1)=NC=C(C(O)=O)C2=O	204,19	C10H8N2O3
Z295451518	CS(=O)(=O)C1=C(NC(=O)CO)C=CC=C1	229,25	C9H11NO4S
Z89324450	COC1=C(C=C(C)C=C1)S(=O)(=O)N(C)C	229,29	C10H15NO3S
Z101185962	COC1=C2OCCOC2=CC(=C1)C(N)=O	209,20	C10H11NO4
Z235345619	COC1=C(OC)C=C(C=C1)C(O)=O	182,18	C9H10O4
Z30612298	CC(C)CC(=O)NC1=CC=C(C=C1)C#N	202,26	C12H14N2O
Z234897551	NC1=C(C=C(C=C1)N1CCCC1)C(O)=O	206,25	C11H14N2O2
Z1169949823	C1CC(C1)C1=NC(=NO1)C1=CN=CC=C1	201,23	C11H11N3O
Z133743156	CC(NS(C)(=O)=O)C1=CC(F)=CC=C1	217,26	C9H12FNO2S
Z1335657450	CCN1N=CC2=C1CCCC2N	165,24	C9H15N3
Z228585382	OCC(=O)N1CCCCCC1	157,21	C8H15NO2
Z1276751957	CC1CCNC(C1)C1=CC=CO1	165,24	C10H15NO
Z1255523342	CC1=CC=C(O1)C(O)CN	141,17	C7H11NO2
Z1333043516	COC1=CC2=C(C=C1)C(O)CO2	166,18	C9H10O3
Z1333717727	OC(=O)CCC1CCCOC1	158,20	C8H14O3
Z1250132472	CC1NCCS(=O)(=O)C1C	163,24	C6H13NO2S
Z1267885765	CC(C)C(N)C1=C(C)C=CC=N1	164,25	C10H16N2
Z1267882052	C=CCOC1CCNCC1	141,21	C8H15NO
Z1262237384	NC1CCN(CC2=CC=CS2)CC1	196,31	C10H16N2S
Z149387406	C=CCN1N=NN(C1=O)C1=CC=CC=C1	202,22	C10H10N4O
Z1258578217	CCC1=NC(C)=C(S1)C(C)O	171,26	C8H13NOS
Z1354416067	COC1=NC(=O)C(F)=CN1	144,11	C5H5FN2O2
Z1347206020	CNS(=O)(=O)C1=C(C)C=C(Cl)C(C)=C1	233,71	C9H12ClNO2S
Z1342879029	COC1=CC=C(CN2C=NC(N)=N2)C=C1	204,23	C10H12N4O
Z1342879027	CNS(=O)(=O)CC1CCCN1	178,25	C6H14N2O2S
Z1342208417	FC1=C2CN=C3CCCN3C2=CC=C1	190,22	C11H11FN2
Z1335657678	CCC1=NC(=CN1)C(O)=O	140,14	C6H8N2O2
Z1333761880	CC(C(O)=O)C1=CC2=C(O1)C=CC=C2	190,20	C11H10O3
Z27805821	CC(=O)NC1=CC=C(F)C=C1	153,16	C8H8FNO
Z1281718596	C(NC1=NC=CN2C=NN=C12)C1CCC1	203,25	C10H13N5
Z1357774299	NC(C(O)=O)C1=CC=CS1	157,19	C6H7NO2S
Z1357774297	CC1(O)CCC2=C1C=CC=C2	148,21	C10H12O
Z1357774199	OC(CC1CCCN1)C1=CC=CS1	197,30	C10H15NOS
Z1816233640	NC1=CN(CC(O)=O)N=C1	141,13	C5H7N3O2
Z1378430661	CN(C)S(=O)(=O)C1=CC2=C(O1)C=CC=C2	225,26	C10H11NO3S
Z276754694	CC(C)N(C(C)C)C(=O)C1=CC=CC=C1	205,30	C13H19NO
Z1801307734	CC(C)(O)C1=NN=C(N)O1	143,15	C5H9N3O2
Z1801307814	O=C1NCC2=C1C=CC=N2	134,14	C7H6N2O
Z1800620533	CC(O)(CCN)C1=CC=CC=C1	165,24	C10H15NO
Z228585444	OCC(=O)NC1=CC=CC=C1	151,17	C8H9NO2
Z1778350582	OC(=O)C1(CC2=C(Cl)C=CC=C2)CC1	210,66	C11H11ClO2
Z1601556832	CN(C)C(=O)CNC1=C(F)C=CC=C1C	210,25	C11H15FN2O

Z94597856	CN(C)S(=O)(=O)C1=C(C)NN=C1C	203,26	C7H13N3O2S
Z449369024	CC1=NC(N)=C(C=N1)C#N	134,14	C6H6N4
Z449369112	CCC1=C(C=NC=N1)C(O)=O	152,15	C7H8N2O2
Z444349766	OC(=O)C1CCC(=O)NC1	143,14	C6H9NO3
Z367858598	CCC1=CC=C(O1)C(=O)NC1=NN=CS1	223,25	C9H9N3O2S
Z363121080	CC1=C(C=CC(=N1)C(=O)NC1CC1)C#N	201,23	C11H11N3O
Z432102204	CC1=C(CCC(O)=O)C(C)=NC(=O)N1	196,21	C9H12N2O3
Z244345748	CNC(=O)C1=C(C=CS1)N1C=CC=C1	206,26	C10H10N2OS
Z425389620	OC(=O)C1=CC=C(C=C1)N1CCNC1=O	206,20	C10H10N2O3
Z415636688	CC1=CC2=NC=C(C(O)=O)C(=O)N2C=C1	204,19	C10H8N2O3
Z406680082	OC(=O)C1=CC(=CC=C1)N1CCNC1=O	206,20	C10H10N2O3
Z398557072	CS(=O)(=O)NCC1CCCNC1	192,28	C7H16N2O2S
Z234894111	NC1=NN=C(O1)C1=CC2=C(CCCC2)S1	221,28	C10H11N3OS
Z398513148	CC(C)C1=C(C=NN1)C(O)=O	154,17	C7H10N2O2
Z398513314	CC(C)C1=NC(C)=C(S1)C(O)=O	185,24	C8H11NO2S
Z242124466	CNC(=O)C1=NOC(=C1)C1=CC=CS1	208,24	C9H8N2O2S
Z108544622	CN1C=CC(NC(=O)C2=CC=CS2)=N1	207,25	C9H9N3OS
Z57068946	NC(C(O)=O)C12CC3CC(CC(C3)C1)C2	209,29	C12H19NO2
Z383822280	NC1=C(N=CC=N1)C(=O)NCC1CCC1	206,25	C10H14N4O
Z27167166	O=C(NC1=NN=C(S1)C1CC1)C1CC1	209,27	C9H11N3OS
Z935140740	Cl.CC1=CC(C)=C(CN)C(C)=C1	185,70	C10H16ClN
Z916919184	CC(=O)NC1=C(C=CC=C1Cl)C(O)=O	213,62	C9H8ClNO3
Z1741963648	OC(=O)C1CC2=C(C1)C=CC=C2	162,19	C10H10O2
Z1250080932	OC(=O)C1(O)CC2=C(C1)C=CC=C2	178,19	C10H10O3
Z1742054869	OC(=O)CN1N=CN2C=CC=C2C1=O	193,16	C8H7N3O3
Z1171978864	CC(C)C(N1CCCC1=O)C(O)=O	185,22	C9H15NO3
Z812517016	OC(=O)CN1C(=O)NC2=C1C=CC=C2	192,17	C9H8N2O3
Z2327039956	CC(C)N1C(=O)NC2=C1C=C(F)C=C2	194,21	C10H11FN2O
Z1258578180	NCC1=CC2=C(C=CO2)C=C1	147,18	C9H9NO
Z1273048546	CC1=CC2=C(C=C1)N=C(CCN)O2	176,22	C10H12N2O
Z2234631222	OC(=O)C1CCCC1C1=CC=CC=C1	190,24	C12H14O2
Z2087745156	NCC1CCC2=NC(=CN2C1)C(F)(F)F	219,21	C9H12F3N3
Z255150112	CC1=NC2=C(CC(N)CC2)C=N1	163,22	C9H13N3
Z1827889368	NCC1CCOC2(CCOC2)C1	171,24	C9H17NO2
Z1262395967	NCCC1=NN(C=C1)C1=CC(F)=CC=C1	205,24	C11H12FN3
Z1335657813	CC(O)CC1=NC2=C(S1)C=CC=C2	193,26	C10H11NOS
Z1335657668	OC(=O)C1CC11CCC2=C1C=CC=C2	188,23	C12H12O2
Z1276751393	CC(N)C1=CC=C(C=C1)N1C=CC=N1	187,25	C11H13N3
Z1272684691	O=C1CC2(CN1)CCOCC2	155,20	C8H13NO2
Z1207823996	CN(CC(C)(C)O)C1=NC=C(Br)C=N1	260,14	C9H14BrN3O
Z1263602401	CN1C2=C(C=CC=C2)C(=O)NCC1=O	190,20	C10H10N2O2
Z449369118	CC(C)C1=C(C=NC=N1)C(O)=O	166,18	C8H10N2O2
Z1868430499	CN1N=C(CC(O)=O)CC1=O	156,14	C6H8N2O3
Z1945708568	CC(C)C1=NC(C(F)(F)F)=NN1	193,17	C7H10F3N3
Z1820077409	CNC(CN)C1=CC=C(F)C=C1	168,22	C9H13FN2
Z1820078920	CC(C)CN1N=CC(N)=C1C(C)C	181,28	C10H19N3
Z2255334838	OC(=O)C12CCCC1CNC2	155,20	C8H13NO2
Z1820078487	NCC1=NC2=C(C=CC=C2)N1C1CC1	187,25	C11H13N3
Z33448386	COCC(=O)NC1=CC2=C(CCC2)C=C1	205,26	C12H15NO2
Z29693768	O=C(NCC1=CC=CS1)C1=CC=CC=C1	217,29	C12H11NOS
Z30624434	CCC(=O)N1CCC2=C1C=CC=C2	175,23	C11H13NO
Z139720892	COC1=NC=C(C=C1)C(O)=O	153,14	C7H7NO3
Z131022006	CC1=C(C)C(C#N)=C(N)O1	136,15	C7H8N2O
Z45528360	CCNS(=O)(=O)C1=C(C)C=CC(C)=C1	213,30	C10H15NO2S
Z57228584	CC(NC(=O)C1=CC=CS1)C(O)=O	199,22	C8H9NO3S
Z217102738	CN1N=C(C)C(C(O)=O)=C1C	154,17	C7H10N2O2
Z30280214	CCNC(=O)C1=C(Br)C=CC(OC)=C1	258,12	C10H12BrNO2
Z1849012892	CC1=CC(=O)C2=C(N1)C=CC=C2	159,19	C10H9NO
Z1889861359	CCC1=CC=C(C=C1)N1CCNC1=O	190,25	C11H14N2O
Z1889706353	OC(=O)C1=C2OCCOC2=C(Cl)C=C1	214,60	C9H7ClO4
Z1881599258	OCC1(CCOCC1)C1=CC=CC=C1	192,26	C12H16O2
Z1881599850	OC1CCN(C1=O)C1=CC(Br)=CC=C1	256,10	C10H10BrNO2
Z1874937335	CN(C)C(=O)C1(CC1)C(O)=O	157,17	C7H11NO3
Z1260735513	CNC(=O)C1=CN=C(S1)C1=CC=NC=C1	219,26	C10H9N3OS
Z1259273134	OC(=O)C1(O)CCOCC1	146,14	C6H10O4
Z1176890038	CC1CC2=C(C=CC=C2)C1NS(C)(=O)=O	225,31	C11H15NO2S
Z1188322569	CCN(C)C(=O)NC1=CC(Cl)=C(F)C=C1	230,67	C10H12ClFN2O

Z1222283885	NCCNC1=C(Cl)C=C(Cl)C=N1	206,07	C7H9Cl2N3
Z1222283884	NC(=O)C1=CC2=C(NCCC(=O)N2)C=C1	205,22	C10H11N3O2
Z1222278648	CN1C=C(C(O)=O)C(=N1)C1CCCCC1	208,26	C11H16N2O2
Z1217170566	CN1NC(=O)C2=C1N=CC=C2	149,15	C7H7N3O
Z1127124543	O[C@@H]1CCN(C1)C1=NC2=C(S1)C=CC=C2	220,29	C11H12N2OS
Z755015062	O=C(NC1CCCCCC1)C1=CNN=N1	208,27	C10H16N4O
Z335675800	OCCOC1=CC(Cl)=CC(Cl)=C1	207,05	C8H8Cl2O2
Z1245633199	CC(C)C(N)C1=NC=CN1C	153,23	C8H15N3
Z1575143878	CC(N)CCN1C=NC=N1	140,19	C6H12N4
Z1575082122	C1CNC(C1)C1=NNC=C1	137,19	C7H11N3
Z737615774	CN(CC1CC1)C(=O)C1=C(C)SC(C)=C1	223,33	C12H17NOS
Z422952578	CN(CC(N)=O)C(=O)C1=CC(Br)=CN=C1	272,10	C9H10BrN3O2
Z422344882	CC1=C(SC=N1)C(=O)N1CCNC(=O)C1	225,27	C9H11N3O2S
Z1172115917	CN1CCC(CC1=O)C(O)=O	157,17	C7H11NO3
Z1172068250	CC(C(O)=O)C1=CC2=C(OCCO2)C=C1	208,21	C11H12O4
Z1172068150	OC(=O)CN1CCCNC1=O	158,16	C6H10N2O3
Z1171979242	CCN1C=C(CC(O)=O)C2=C1C=CC=C2	203,24	C12H13NO2
Z1232874903	CCNS(=O)(=O)N1CCN2CCCC2C1	233,33	C9H19N3O2S
Z1526453862	CC(C)(CO)C1CCCC1	142,24	C9H18O
Z1268153220	CC(CN)OC1=CC(Br)=CC=C1	230,11	C9H12BrNO
Z1407654393	CC1CC(N)CN1CC1=CC=CC=C1	190,29	C12H18N2
Z2509351132	CCC1=C(N)N=C(C)C=C1	136,20	C8H12N2
Z2492774465		211,26	C11H17NO3
Z2489418727	CC1=C(OC(=O)C2=C1C=CC=C2)C(O)=O	204,18	C11H8O4
Z2472860046	CCN1C(=O)NC2=C1C=C(C=C2)C(O)=O	206,20	C10H10N2O3
Z2471547774	OC(=O)C1=C2NC(=O)COC2=CC=C1	193,16	C9H7NO4
Z1982493929	CN1N=CC2=C1CC(CC2)C(O)=O	180,21	C9H12N2O2
Z2509351166	CCN(C(C)=O)C1=NC=C(N)C=C1	179,22	C9H13N3O
Z1973466038	OC(=O)C1=C(OC=C1)C1CC1	152,15	C8H8O3
Z1455010668	OC(=O)CNC(=O)C1=C(Br)C=CC=C1F	276,06	C9H7BrFNO3
Z353237440	CC(CO)NC(=O)C1=C(C)C=CC(F)=C1	211,24	C11H14FNO2
Z1946684511	OC1CCCOC2=C1C=CC=C2F	182,19	C10H11FO2
Z29692416	CC1=CC=C(O1)C(=O)NCC1=CC=CS1	221,27	C11H11NO2S
Z1891775701	CN1CCC(O)(CN)CC1	144,22	C7H16N2O
Z1992316417	NC1=CC(CN2CCOCC2)=C(Cl)C=C1	226,70	C11H15ClN2O
Z1992316403	CN1C=C(C(O)=O)C(=O)C=C1C	167,16	C8H9NO3
Z1983100835	OC(=O)CC1CCCCC(=O)N1	171,20	C8H13NO3
Z1983080176	OC(=O)CC1CCCCNC1=O	171,20	C8H13NO3
Z1983079259	OC(=O)C1=CC(=NO1)C1CC1	153,14	C7H7NO3
Z993967154	CNC(C(O)=O)C1=CC(Cl)=C(Cl)C=C1	234,08	C9H9Cl2NO2
Z993967218	CC1=CC=C(S1)C1=NC(N)=NN=C1	192,24	C8H8N4S
Z19739650	CC(OC1=CC=C(C=C1)C#N)C(N)=O	190,20	C10H10N2O2
Z2453029853	CC1=CC(C2=NNC(N)=C2)=C(F)C=C1	191,21	C10H10FN3
Z2692095159	CCC1(CCNCC1)N1CCOCC1	198,31	C11H22N2O
Z2692095103	CC1(COC2=C(O1)C=CC=C2)C(O)=O	194,19	C10H10O4
Z2692095025	OC(=O)C1=C(NC(=C1)C1CC1)C1CC1	191,23	C11H13NO2
Z2692093988	COC1=NC(N)=C2C=CC=CC2=C1	174,20	C10H10N2O
Z2273464199	FC1=C(N[C@H]2CCCNC2=O)N=CC=C1	209,22	C10H12FN3O
Z1259155996	CC1CN(CC(C)O1)C1=NC=C(N)C=C1	207,28	C11H17N3O
Z1259155942	CC1=C(CO)OC2=C1C=CC=C2	162,19	C10H10O2
Z254580486	CN(C)C(=O)CN1C=CC=N1	153,19	C7H11N3O
Z1846840792	C(NC1CCCCC1)C1=CNN=N1	180,26	C9H16N4
Z1667560467	CN1N=C(C)N=C1NC(=O)C1CC=CC1	206,25	C10H14N4O
Z1837073496	O=C1CCC(CC2=CC=CC=C2)N1	175,23	C11H13NO
Z1837073270	C(NC1=CC=CC=C1)C1=CNN=N1	174,21	C9H10N4
Z1836338761	CN1CCCCC1CC(O)=O	157,21	C8H15NO2
Z1741962792	CC1=CSC(C(O)=O)=C1Cl	176,61	C6H5ClO2S
Z1836338742	COC1(CC(O)=O)CCC1	144,17	C7H12O3
Z1836181359	CC(C)N1C=C(NC(=O)C2(N)CC2)C=N1	208,27	C10H16N4O
Z1834278543	CC1=C2C=CC=C(Br)C2=NN=C1	223,07	C9H7BrN2
Z1827897920	CC1C(CCN1C1=CC=CC=C1)C(O)=O	205,26	C12H15NO2
Z1258992713	CC1CCCC(C)C(=O)N1	141,21	C8H15NO
Z135897268	NC(=O)C1C2CC3CC(C2)CC1C3	179,26	C11H17NO
Z1255523379	CC1=NC2=C(C=CC=C2C(O)=O)C=C1	187,20	C11H9NO2
Z1250132718	OC(=O)C1=CC(=CC=C1)N1CC=CC1	189,21	C11H11NO2
Z1250132694	CC(CC1=C(C)ON=C1C)C(O)=O	183,21	C9H13NO3
Z410097646	CN(C)C(=O)NC1=CC(=CC=C1)C#N	189,22	C10H11N3O

Z1250132381	OCC1CN(C(=O)C1)C1=CC=CC=C1	191,23	C11H13NO2
Z1610998441	CC1=NSC(NCC2=CN=CS2)=N1	212,29	C7H8N4S2
Z1629024437	C[C@H](NC(=O)C1=NC=C(Br)C=C1)C(N)=O	272,10	C9H10BrN3O2
Z1259341037	CC(C)NC1=CN=CC=C1	136,20	C8H12N2
Z1259340994	CC1=C(C)C(=O)N=C(N1)C1=CC=NC=C1	201,23	C11H11N3O
Z1259335789	COC1=C(C(O)=O)C(C)=NN1C	170,17	C7H10N2O3
Z1259335778	CC1=CC(=CC=C1)C(N)C(O)=O	165,19	C9H11NO2
Z1259335770	CN1C=C(C=N1)C1=NC=C(S1)C(O)=O	209,22	C8H7N3O2S
Z1259273402	OC(=O)COC1CCOCC1	160,17	C7H12O4
Z1259161763	CC(N)C1=CN(C)N=C1C	139,20	C7H13N3
Z1460222813	FC1=C(NC2CCNC2=O)C=CC(Cl)=C1	228,65	C10H10ClFN2O
Z1267773633	CCC1=NC=C(CNC)S1	156,25	C7H12N2S
Z1268168726	CC(=O)NCC1=CNC=N1	139,16	C6H9N3O
Z2169086459	CN1C=C(Br)C=C(F)C1=O	206,01	C6H5BrFNO
Z2037267084	CC1=NC2=C(N1)C=CC=C2C(O)=O	176,18	C9H8N2O2
Z2218249029	CNS(=O)(=O)C1=C(F)C=CS1	195,23	C5H6FNO2S2
Z2684295940	CN1CCC(CC(N)C(O)=O)CC1	186,26	C9H18N2O2
Z734660320	CNC(=O)C1=CC2=C(C=CC=C2)N1C	188,23	C11H12N2O
Z2681277212	CN1C=C(N)C(=N1)C1=CC=CO1	163,18	C8H9N3O
Z1741980128	COC1=C2CNC(=O)C2=CC=C1	163,18	C9H9NO2
Z135504726	CN(C)C(=O)C1=CC=CN1	138,17	C7H10N2O
Z1171979256	CC1=NC=CN1C1=CC(=NC=C1)C(O)=O	203,20	C10H9N3O2
Z1171979243	CCN1C=C(Br)C=C1C(O)=O	218,05	C7H8BrNO2
Z1171978890	CC1=NC2=C(C=NN2C=C1)C(O)=O	177,16	C8H7N3O2
Z85895893	CC(=O)N1CCCCC1C(O)=O	171,20	C8H13NO3
Z1094758722	OC(=O)C1=C2OCC(=O)NC2=CC=C1	193,16	C9H7NO4
Z1171978880	CC1=NC(=CS1)C1=CC(=CO1)C(O)=O	209,22	C9H7NO3S
Z1171978866	COC1=CC=C(C=C1)C1(CCC1)C(O)=O	206,24	C12H14O3
Z54082288	CN1C=CC=C1C(=O)NCC1=CC=CO1	204,23	C11H12N2O2
Z85525679	CC(NC(C)=O)C(=O)NC1=NC=CC=C1	207,23	C10H13N3O2
Z354838940	CCNC(=O)C1CCN(CC1)C(C)C	198,31	C11H22N2O
Z954532756	CCNC(=O)C1=CNC2=C1C=CC(Cl)=C2	222,67	C11H11ClN2O
Z55928890	NC1=CC=NN1C1CCCCC1	165,24	C9H15N3
Z1162910732	OC(C1=CC=CS1)C1=NC=CC=C1	191,25	C10H9NOS
Z1160899501	COC1=C(N)C=C(CN2CCCC2)C=C1	206,29	C12H18N2O
Z1770192727	CC1(C)CC(O)C(C)(C)O1	144,21	C8H16O2
Z1768160555	CN1C(=O)CCC2=C1C=CC(=C2)C(O)=O	205,21	C11H11NO3
Z1768160552	OC(=O)C1CCCN2C(=O)NN=C12	183,17	C7H9N3O3
Z1767916692	CC(=O)NC1C(=O)NC2=C(C)C=CC=C12	204,23	C11H12N2O2
Z1766245361	CCC1=CC2=C(C=C1)N=C(O)N2	162,19	C9H10N2O
Z1762997280	C[C@](N)(C1CCCCC1)C(O)=O	171,24	C9H17NO2
Z1753033011	CC(C)OC1=NN=C(N)C=C1	153,19	C7H11N3O
Z1610969627	N#CC1=CSC(CNC2=NN=CS2)=C1	222,28	C8H6N4S2
Z275170072	CCN1N=CC=C1C(O)=O	140,14	C6H8N2O2
Z85888094	CC(=O)NC1(CCCCC1)C(O)=O	185,22	C9H15NO3
Z1295559816	CCC(N)C1=NC(=CS1)C(F)(F)F	210,22	C7H9F3N2S
Z2211246487	COC1=C2CCCNC2=CC=C1	163,22	C10H13NO
Z1558209772	CCN1C=CC(=O)C(N)=C1	138,17	C7H10N2O
Z1335657438	C1CNC(C1)C1=CC=CO1	137,18	C8H11NO
Z1262237470	C1CNCC2(C1)CCOCC2	155,24	C9H17NO
Z1367012439	COC1=NC(CO)=CC=C1	139,15	C7H9NO2
Z1900627279	FC1=CCCN(C1)C1=CN=C(C=C1)C#N	203,22	C11H10FN3
Z2689172043	CN1C(=O)COC2=C1C=C(C=C2)C(O)=O	207,19	C10H9NO4
Z2130642054	COC1=C(CO)C=NC=C1	139,15	C7H9NO2
Z2130642358	CC1=C(C=CC(N)=N1)C1=CC=CC=C1	184,24	C12H12N2
Z2130643237	CCNC1=C(N)C=NC=C1	137,19	C7H11N3
Z2130641782	CC1=C2OC(N)=NC2=CC=C1	148,17	C8H8N2O
Z2120061007	OCC1=C(N=CC=C1)N1CCCC1	178,24	C10H14N2O
Z2092370541	ClC1=CC=C(C=C1)N1CCNC1=O	196,63	C9H9ClN2O
Z2658867442	OC(=O)C1(CCNC1=O)C1CCCC1	197,23	C10H15NO3
Z1603606725	OC(=O)C1(CC1)NC(=O)C1=CC=CC=C1	205,21	C11H11NO3
Z2034719242	C[C@H](NS(C)(=O)=O)C1=NC=C(Cl)C=C1	234,70	C8H11ClN2O2S
Z1492804399	CC1CCN(CC2=NC(N)=CC=N2)C1	192,27	C10H16N4
Z2160914592	C(C1=CNC=N1)C1=CC=CC=C1	158,20	C10H10N2
Z1741957818	CC1=C(Cl)C(=NN1)C(F)(F)F	184,55	C5H4ClF3N2
Z159528828	CN(CCO)C1CCCCC1	157,26	C9H19NO
Z1547844612	CC(C)(O)C1CCCN1CC1CCOC1	213,32	C12H23NO2

Z1473963139	C1COC2CCNCC2C1	141,21	C8H15NO
Z1428018528	ClC1=C2OCCNC2=CC=C1	169,61	C8H8ClNO
Z1436472689	CN1C=C(C)C=C(N)C1=O	138,17	C7H10N2O
Z56347292	NC1=CC=NN1C1CCCC1	151,21	C8H13N3
Z1742056046	CN(C)C1=NN(C=N1)C1=CC(N)=CC=C1	203,25	C10H13N5
Z1741794237	CC1CNCCN1CCO	144,22	C7H16N2O
Z1266823341	CC(C)(N)CNC1=CC=CC=C1	164,25	C10H16N2
Z1527148054	CN(CCO)C(=O)C1=C(C)C=C(F)C=C1	211,24	C11H14FNO2
Z1704693923	CC(C)C1=NC=C(C=C1)C#N	146,19	C9H10N2
Z1546887028	CC(CO)N(C)C1=NC=C(Cl)C=N1	201,65	C8H12ClN3O
Z165137254	CC(=O)NC1=NN=C(S1)C1=CC=CO1	209,22	C8H7N3O2S
Z234896383	CNC1CCCCNC1=O	142,20	C7H14N2O
Z237578634	CC(C)(O)C1=NC2=C(N1)C=CC=C2	176,22	C10H12N2O
Z19725438	CNC(=O)COC1=CC=C(Br)C=C1	244,09	C9H10BrNO2
Z234853694	CC(C)CC1=NNC(=C1)C(O)=O	168,20	C8H12N2O2
Z57834657	CN1C(=O)N(C)C2=C1C=CC(N)=C2	177,21	C9H11N3O
Z119081056	CC1=C(OC=C1)C(=O)NCC1=CC=CS1	221,27	C11H11NO2S
Z363071686	CN(CC(=O)N1CCCCC1)S(C)(=O)=O	234,31	C9H18N2O3S
Z314448400	CCN(C1CC1)C(=O)C1CCCO1	183,25	C10H17NO2
Z1456185598	CC(N)C1CN(C)CCO1	144,22	C7H16N2O
Z1267773576	NC1CCN(C1)C1=C(Br)C=CC=C1	241,13	C10H13BrN2
Z513575288	CCNC(=O)N1CCC(O)CC1	172,23	C8H16N2O2
Z1172231059	CC1CCC(C)N1CC(O)=O	157,21	C8H15NO2
Z280813686	CC(C1CC1)N(C)C(=O)C1CCC=CC1	207,32	C13H21NO
Z314446644	CCN(C1CC1)C(=O)C1CCC1	167,25	C10H17NO
Z314790120	CN1C=CC=C1C(=O)NCCN	167,21	C8H13N3O
Z752989208	CC1=C(N=C(CN)S1)C1=CC=CC=C1	204,29	C11H12N2S
Z270730164	NC1=C(C=CC=C1)N1CCCCCC1	190,29	C12H18N2
Z2327226178	CC1=C(F)C(F)=CC=C1N	143,14	C7H7F2N
Z2327226118	CN1N=NC=C1CC(O)=O	141,13	C5H7N3O2
Z407486572	COC1=CC2=C(NC=C2CC(N)=O)C=C1	204,23	C11H12N2O2
Z2327039924	CC1=NC=C2C=CC=C(N)C2=C1	158,20	C10H10N2
Z2326990062	CC1=CC=C(CN2CCCNC2=O)C=C1	204,27	C12H16N2O
Z2309759496	COC1=C(C=NN1C)C(O)=O	156,14	C6H8N2O3
Z2301499788	CCN1CCC(=O)NC(C)C1=O	170,21	C8H14N2O2
Z2293608802	OC(=O)C1=C(C=CN1)C1CC1	151,17	C8H9NO2
Z2293606507	COC1=CC(N)=CC(OC)=C1Cl	187,62	C8H10ClNO2
Z1945709775	CC1=C(C=NO1)C1=CSC(C)=N1	180,23	C8H8N2OS
Z2285376686	CN1C(=O)OC2=C1C=C(F)C(N)=C2	182,15	C8H7FN2O2
Z126933712	CCNC1=CC=C(C=C1)C#N	146,19	C9H10N2
Z927632696	CCNC(=O)C1=C(N)C=CC(Cl)=C1	198,65	C9H11ClN2O
Z1266854868	CC(C)C1=NC(N)=NC(C)=N1	152,20	C7H12N4
Z1266854826	COC1=CC2=C(OC(C)C(=O)N2)C=C1	193,20	C10H11NO3
Z1266823232	CNC(C)C1=C(C)N=C(C)S1	170,27	C8H14N2S
Z1262253450	O=C(N1CCNCC1)C1=CNC(=O)N1	196,21	C8H12N4O2
Z1266823151	CCC(C1=NN=C(N)O1)C1=CC=CC=C1	203,25	C11H13N3O
Z87615031	CC(=O)NC1=CC=C(CC(N)=O)C=C1	192,22	C10H12N2O2
Z1263714154	COC1=C(C=C(Cl)C=C1)C1=NN=C(N)O1	225,63	C9H8ClN3O2
Z1208171827	CC1=CC=C(S1)C1CCCN1S(C)(=O)=O	245,36	C10H15NO2S2
Z1741815643	NCCN1CCC2=C(C1)C=CC=C2Cl	210,71	C11H15ClN2
Z57102476	COC1=C(CN)C=CC=C1	137,18	C8H11NO
Z414477974	CS(=O)(=O)NC1CCCCNC1=O	206,26	C7H14N2O3S
Z1436472855	COC1=NN=C2CCNCC2=C1	165,20	C8H11N3O
Z1782591374	C=CCNC1CCCOC2=C1C=CC=C2	203,29	C13H17NO
Z2001754599	COCCC(=O)N1CC(=O)NCC1(C)C	214,27	C10H18N2O3
Z1263529750	NC(CN1CCCCC1)C1=CC=CC=C1	204,32	C13H20N2
Z2157438002	BrC1=CN=C(N=C1)N1CCCCN1	243,11	C8H11BrN4
Z1446874472	CC(N)CC1=CC(C)=NN1	139,20	C7H13N3
Z1717424532	FC1=CCCN(CC2=CSN=N2)C1	199,25	C8H10FN3S
Z1900628669	CC1=NC(=CN=C1)N1CCC=C(F)C1	193,23	C10H12FN3
Z1613884074	CC1=C(OC=C1)C(=O)N1CCC=C(F)C1	209,22	C11H12FNO2
Z1899838847	CC1=NN(CC2=NC=C(F)C=C2)C(=O)S1	225,24	C9H8FN3OS
Z1587273510	CN1C=CN=C1CN1CCC=C(F)C1	195,24	C10H14FN3
Z1551815330	OCC(=O)NC1(CC1)C1=C(F)C=CC=C1	209,22	C11H12FNO2
Z1974574407	CC(=O)N[C@@H]1C[C@H]1C1=C(Cl)C=CC=C1F	227,66	C11H11ClFNO
Z1357827227	CNC1=C(N=CC=C1)N(C)C	151,21	C8H13N3
Z1333761736	C1CN(CCN1)C1CCOCC1	170,26	C9H18N2O

Z1168422657	C1CC1C1=CSC(=N1)C1=CC=NC=C1	202,28	C11H10N2S
Z1231609616	ClC1=CN=C(C=C1)C(=O)NC1CC1	196,63	C9H9ClN2O
Z1891774432	CC(CN)N1CCC2=C1C=CC=C2	176,26	C11H16N2
Z2106606815	NC1=CC2=C(CCCC2)C=C1	147,22	C10H13N
Z2234185604	OC(=O)CC1=CC2=C(S1)C=CC=C2	192,23	C10H8O2S
Z2213893012	NC1CCCC2=C1C=C(Cl)S2	187,69	C8H10ClNS
Z2234631175	CCC1CCCC1C(O)=O	142,20	C8H14O2
Z1717761806	CC(C)(C1CCC1)C(O)=O	142,20	C8H14O2
Z1860991693	CCC1OCCCC1C(O)=O	158,20	C8H14O3
Z2235330356	CCOC1=CC(N)=C(Cl)C=C1	171,62	C8H10ClNO
Z2327531741	CN(C)C1=C(C)N=C(C)C(C)=N1	165,24	C9H15N3
Z1575245647	CN1C=C(CCO)C(C)=N1	140,19	C7H12N2O
Z104377982	Cl.COC1=C2CNCC(O)C2=C(OC)C=C1	245,70	C11H16ClNO3
Z57728665	OC(=O)CCN1C(=O)OC2=C1C=CC=C2	207,19	C10H9NO4
Z57066685	CC1=CC2=C(OC(=CC2=O)C(O)=O)C=C1	204,18	C11H8O4
Z57985380	CCN1CCN(CC1)C1=C(N)C=CC=C1	205,31	C12H19N3
Z90122368	CC(=O)N1CCN(CC1)C(N)=O	171,20	C7H13N3O2
Z85932257	CC(O)C1=NC2=C(C=CC=C2)N1C(F)F	212,20	C10H10F2N2O
Z57683752	CC1=C(CC(O)=O)C(C)=NN1	154,17	C7H10N2O2
Z57625083	CN1CCN(CC1)C1=C(N)C=CC=C1	191,28	C11H17N3
Z26743827	CCC(=O)NC1=CC(Cl)=C(OC)C=C1	213,66	C10H12ClNO2
Z56347071	CC(O)C1=NC2=C(S1)C=CC=C2	179,24	C9H9NOS
Z45527657	CNS(=O)(=O)C1=CC=C(OC)C=C1	201,24	C8H11NO3S
Z240299438	CN1C=CC(NC(=O)C2=CC(Br)=CN2)=N1	269,10	C9H9BrN4O
Z818727398	CC(N)COC1=C(C)C=CC=C1C	179,26	C11H17NO
Z812516948	CCC1=C(SC=C1)C(O)=O	156,20	C7H8O2S
Z422426600	CC(C)C(C)NC(=O)C1=C(C)N=CS1	212,31	C10H16N2OS
Z295814046	CNC(=O)C1=C(C)SC(=N1)C1=CC=CO1	222,26	C10H10N2O2S
Z102345362	CC(NC(=O)C1=NNC(=O)C=C1)C1CC1	207,23	C10H13N3O2
Z803145246	CC(C)C(N1CCN(C)CC1)C(O)=O	200,28	C10H20N2O2
Z422952260	CN(CC(N)=O)C(=O)C1=CC=C(Br)S1	277,14	C8H9BrN2O2S
Z383001878	CNS(=O)(=O)C1=CC=C(O1)C(=O)N(C)C	232,25	C8H12N2O4S
Z226408766	CN1N=C(OC1=O)C1=CC=C(F)C=C1	194,17	C9H7FN2O2
Z397955404	NC(=O)CN1C(=O)OC2=C1C=CC(Br)=C2	271,07	C9H7BrN2O3
Z71179597	COC1=C(C=CC=N1)C(O)=O	153,14	C7H7NO3
Z26968795	COC1=CC(Cl)=C(OC)C=C1NC(C)=O	229,66	C10H12ClNO3
Z212277696	CC(=O)NC1=CC=C(S1)C(=O)NC1CC1	224,28	C10H12N2O2S
Z394693554	NCC(O)C1=CC(Cl)=CC=C1	171,62	C8H10ClNO
Z33546734	CC(CC(N)=O)C1=CC=CC=C1	163,22	C10H13NO
Z108547236	CCC(=O)NC1=CC(=CC=C1)S(C)(=O)=O	227,28	C10H13NO3S
Z381785690	OC(=O)C1=CC=C(CN2C=CC=N2)O1	192,17	C9H8N2O3
Z2025666292	NC(=O)N1CCOC2=C(C1)C=C(Br)C=C2	271,11	C10H11BrN2O2
Z235352209	NC1=CC(=CC=C1)C1=NN=C2CCCN12	200,25	C11H12N4
Z235352197	CC1=NC=C(O1)C1=CC=C(N)C=C1	174,20	C10H10N2O
Z235346973	NC1=NC=C(C=C1)N1CCOCC1	179,22	C9H13N3O
Z235345305	CC1=C(CC(O)=O)N=C(O1)C1=CC=CS1	223,25	C10H9NO3S
Z235341203	CNC1=NC2=C(O1)C=CC=C2	148,17	C8H8N2O
Z56886301	CC1=C(Cl)C(N)=NC=C1Cl	177,03	C6H6Cl2N2
Z32367959	O=C(NC1CC1)C1=CC=C(C=C1)C#N	186,21	C11H10N2O
Z57966275	CC1=NN2CCC(=O)NC2=C1	151,17	C7H9N3O
Z332770958	CC1=CC(NC(=O)C2=COC(Br)=C2)=NO1	271,07	C9H7BrN2O3
Z367451004	O=C1NCC2=C1C=CC=C2	133,15	C8H7NO
Z2470210350	COC1=CNC2=C1C=C(F)C=C2	165,17	C9H8FNO
Z2469680276	COC1=CC2=C(NC(C)=C2)C=C1	161,20	C10H11NO
Z2469606526	O=C1NCC(=C1)C1=CC=CC=C1	159,19	C10H9NO
Z2469606458	OC(=O)C1=C(F)C=C(C=C1)C(F)F	190,12	C8H5F3O2
Z2469606506	CCC(C1CNC(=O)C1)C1=CC=CC=C1	203,29	C13H17NO
Z2467454623	FC1=C(F)C=C2OCCNC2=C1	171,15	C8H7F2NO
Z2467454621	COCC1=CC(N)=NC=C1	138,17	C7H10N2O
Z1954800557	N#CC1=NC2=C(NCCC2)C=C1	159,19	C9H9N3
Z2418194168	CC1=CC=C(C=C1)C1(CCC1)C(O)=O	190,24	C12H14O2
Z2418193694	OC1CCCNC1CCC1=CC=CC=C1	205,30	C13H19NO
Z2412196538	CC1(C)C2CC1NC(=O)CC2	153,23	C9H15NO
Z2412196506	CC1=C(C(O)=O)C(N)=CC=C1F	169,16	C8H8FNO2
Z212230762	CC1CN(CCO1)C(=O)CN1CCCC1	212,29	C11H20N2O2
Z1252468609	COCCN1N=C(C)C=C1C	154,21	C8H14N2O
Z1262252989	NCCC1=C(Br)C=CC=C1	200,08	C8H10BrN

Z426201150	OCCOC1=CC(Br)=CC=C1	217,06	C8H9BrO2
Z1374788746	NC1(CC1)C1=CC(F)=C(F)C=C1	169,18	C9H9F2N
Z1269702393	CCC(N)C1=C(C)N(C)N=C1C	167,26	C9H17N3
Z235336557	OC(C1CCNCC1)C1=CC=CC=C1	191,27	C12H17NO
Z235333131	CC1=NN2C(=C1)N=CC(C(O)=O)=C2C	191,19	C9H9N3O2
Z235333081	OC(=O)C1=CN(CC2=CC=CC=C2)N=N1	203,20	C10H9N3O2
Z168883346	CC1=C(C(O)=O)C(C)=CC(=O)O1	168,15	C8H8O4
Z89268045	CC1=CC=C(CN2N=CC=C2N)S1	193,27	C9H11N3S
Z1159152696	NC(=O)C1CCCCN1C1CCCC=C1	208,31	C12H20N2O
Z1238477760	CN(C)C(=O)C1=C(N)C=C(Cl)C=C1	198,65	C9H11ClN2O
Z195562120	CNCC1=NC2=C(C=CC=C2)C(N)=N1	188,23	C10H12N4
Z1725496598	CC(=O)NC1=C2NC=CC2=CC=C1	174,20	C10H10N2O
Z1145207891	CS(=O)(=O)NC1CCC2=C1C=C(F)C=C2	229,27	C10H12FNO2S
Z1222331444	CC(C)OC1=NC(C(O)=O)=C(Cl)C=C1	215,63	C9H10ClNO3
Z1222331409	OC(=O)C1CCC2=NN=C(C3CC3)N2C1	207,23	C10H13N3O2
Z2255112112	CC1(CCC(=O)N1)C(O)=O	143,14	C6H9NO3
Z1437171658	CNC(C)C1=CN(C)N=C1	139,20	C7H13N3
Z1245633213	CC(N)C1=CC(OC(F)F)=CC=C1	187,19	C9H11F2NO
Z1251171288	CC1=CC(NC2CCNCC2)=CC=C1	190,29	C12H18N2
Z31192919	N#CC1=C(N=CC=C1)N1CCCCC1	187,25	C11H13N3
Z1250132548	OC1CCCC1C1=CC=C(F)C=C1	180,22	C11H13FO
Z1267882056	NCCC1=CSC(=N1)N1CCCC1	197,30	C9H15N3S
Z1267773558	CN1CCC(CC1)N1CCC(N)C1	183,30	C10H21N3
Z1259161657	CNC1CCCCC1S(C)(=O)=O	191,29	C8H17NO2S
Z1350579651	CC(O)C(C)C1=NC2=C(S1)C=CC=C2	207,29	C11H13NOS
Z1262252970	CCC1=CC(=CC=C1)N1CCC(N)CC1	204,32	C13H20N2
Z422471910	CC(C1CC1)N(C)C(=O)C1=C(C)N=CS1	224,32	C11H16N2OS
Z1268153229	CC(C)CN1C=C(N)C=N1	139,20	C7H13N3
Z2242946408	CC(O)C1NCC2=C1C=CC=C2	163,22	C10H13NO
Z1741969705	CC1=NC2=C(O1)C=CC=C2N	148,17	C8H8N2O
Z2235831713	CN1C=C(N)C(=N1)C(=O)NCC1CC1	194,24	C9H14N4O
Z1262564339	C(C1=NN=CN1)C1=CC=CC=C1	159,19	C9H9N3
Z2238934532	BrC1=CC(=CC=C1)C1CCNC1=O	240,10	C10H10BrNO
Z1980894300	CC1=NC(CO)=NC(=C1)N1CCCCC1	207,28	C11H17N3O
Z1266823371	CN(C)CC1CNCCO1	144,22	C7H16N2O
Z1437173851	CNC1CCCC2=C1C=NN2C	165,24	C9H15N3
Z2255110160	CC1=C2SC(=O)NC2=CC=C1	165,21	C8H7NOS
Z90517827	C(NC1CC1)C1=CN=CC=C1	148,21	C9H12N2
Z1983132442	CC(O)C1=NC=C(C=C1)C(F)(F)F	191,15	C8H8F3NO
Z372962240	CN(C)C1=C(C=CC=C1)C#N	146,19	C9H10N2
Z745184320	CN(C)C(=O)CCN1C=C(Br)C=N1	246,11	C8H12BrN3O
Z137588472	NC(=O)CNC1=C(Br)C=CC=C1	229,08	C8H9BrN2O
Z1992316316	CC(C)CC1(CC1)C(O)=O	142,20	C8H14O2
Z1347251965	CC(C)(O)CCN1CCCC1	157,26	C9H19NO
Z1953500072	CC(N)C1CN(C)CCN1C	157,26	C8H19N3
Z803153238	CC(C)C1=NC(CCN)=CS1	170,27	C8H14N2S
Z1945984181	COC1(CN)CCC(C)CC1	157,26	C9H19NO
Z1945984192	CC1=NC(CCN)=CC=C1	136,20	C8H12N2
Z1949075885	CC1=C(Br)C(N)=C(F)C=C1	204,04	C7H7BrFN
Z183344018	CC(C)NC(=O)N(C)CC1=CN(C)N=C1	210,28	C10H18N4O
Z1480642078	CC(N)C(C)N1C=CC=N1	139,20	C7H13N3
Z1913645786	OCC12CCCN1CCC2	141,21	C8H15NO
Z1723418925	CC1(C)CCCC1C(O)=O	142,20	C8H14O2
Z1545196403	FC1=C(NCC2=NNC=C2)C=CC=C1	191,21	C10H10FN3
Z1664348558	CC1=NC(C)=C(N)C(N)=C1	137,19	C7H11N3
Z1148165337	CN(C)C(=O)CN1N=CC2=C1C=CC=C2	203,25	C11H13N3O
Z1696860270	CC1=NC(=CS1)C1=C(C)OC(=C1)C(O)=O	223,25	C10H9NO3S
Z1696844798	CC1=NC2=CC(=NN2C(C)=C1)C(O)=O	191,19	C9H9N3O2
Z360056164	C[C@@H](O)C1=CC=C(C=C1)N1C=NC=N1	189,22	C10H11N3O
Z1696091879	OC(=O)[C@@H]1C[C@@]1(C(O)=O)C1=CC=CC=C1	206,20	C11H10O4
Z1695921492	CC(C)(OC1=CC=C(C=C1)C#N)C(O)=O	205,21	C11H11NO3
Z2004547759	BrC1=C2NCCCN2N=C1	202,06	C6H8BrN3
Z1631503812	CC(=O)NC1=NC=CC(=C1)C(F)(F)F	204,15	C8H7F3N2O
Z932997760	CNC(=O)CNC(=O)C1=CC(N)=CC=C1	207,23	C10H13N3O2
Z354263462	CN(C)C1=NC(N)=NC(C)=C1	152,20	C7H12N4
Z1993522718		192,21	C11H12O3
Z1702887683	CC1CN(C1)C(=O)NC1=CC=CC=C1	190,25	C11H14N2O

Z1439138555	BrC1=C(NC2CC2)N=CN=C1	214,07	C7H8BrN3
Z2028214407	OC(=O)C1CCS(=O)(=O)C2=C1C=CC=C2	226,25	C10H10O4S
Z2028214362	CC1=C(OC(CO)=C1)C(O)=O	156,14	C7H8O4
Z2028214426	FC(F)(F)C1=CC2=C(C=C1)C(=O)NC2	201,15	C9H6F3NO
Z1741967141	COC1=C(Cl)C=C(C=N1)C(O)=O	187,58	C7H6ClNO3
Z2027049490	OC(=O)C1=C(NC=C1)C1CC1	151,17	C8H9NO2
Z2027049525	NCCC1=CC2=NNC=C2C=C1	161,21	C9H11N3
Z1682064802	COC1=C(OC)C(Br)=CC(=C1)C(O)=O	261,07	C9H9BrO4
Z208334426	CN(C1CCS(=O)(=O)C1)C(=O)NC1CC1	232,30	C9H16N2O3S
Z1642061413	CN1C=NC(=N1)C1=CC=C(C=C1)C(O)=O	203,20	C10H9N3O2
Z1623872209	CNCC1CCCCC1O	143,23	C8H17NO
Z1623864185	CCC1=NC2=C(C=C(N)C=C2)C(N)=C1	187,25	C11H13N3
Z57370050	CC(O)C1=NC2=C(N1)C=CC(C)=C2	176,22	C10H12N2O
Z198264916	C=CCNC(=O)C1=CC2=C(NN=N2)C=C1	202,22	C10H10N4O
Z1575311809	CN1C=CC(=N1)N1CCC(N)C1=O	180,21	C8H12N4O
Z1569709170	OC(=O)C1=NC(=NC=C1)C1CCCC1	192,22	C10H12N2O2
Z929183718	CC(C)(NC(=O)C1=CC=CC=C1)C(O)=O	207,23	C11H13NO3
Z1230797900	CC(C1CC1)N(C)C(=O)C1=NC=CN1C	207,28	C11H17N3O
Z103698412	N#CC1=C(CN2CCCCC2)C=CC=C1	200,29	C13H16N2
Z1834223522	CC1(C)CC2=C(O1)C(N)=CC=C2	163,22	C10H13NO
Z1820225788	CCNC1=C2C=CNC2=CC=C1	160,22	C10H12N2
Z992422556	CC(N)CCC1CCCO1	143,23	C8H17NO
Z1416200831	CC(N)C1=C(C=CC=C1)N1C=CC=N1	187,25	C11H13N3
Z32064550	C=CCNC(=O)C1=CC=NC=C1	162,19	C9H10N2O
Z1269702208	NC1CCC1C1=CC=CC=C1	147,22	C10H13N
Z90122603	CC(N)C1=CC=C(F)C=C1	139,17	C8H10FN
Z1695709587	OC(=O)C1=C(NN=C1)C1CC1	152,15	C7H8N2O2
Z1683067954	N#CC1=NC(=CC=C1)N1CCNCC1	188,23	C10H12N4
Z1265813904	CN(C)C(=O)C1=CC2=C(O1)C(F)=CC=C2	207,20	C11H10FNO2
Z1665033540	CC1CC(C)(C)CNC1=O	141,21	C8H15NO
Z2158786427	O=C(NC1=CC=NC=C1)N1CCCCN1	206,25	C10H14N4O
Z2235681471	OC(=O)C1=CN=C(C=C1)N1CCC1	178,19	C9H10N2O2
Z2235330314	BrC1=CC2=C(OCC2)N=C1	200,04	C7H6BrNO
Z2061490609	C[C@@H]1CN(C[C@H]1C(N)=O)C(=O)C1(C)CC1	210,28	C11H18N2O2
Z275127490	CC(CC(O)=O)C1=CNC2=C1C=CC=C2	203,24	C12H13NO2
Z2218556484	OCC1CS(=O)(=O)C2=C1C=CC=C2	198,24	C9H10O3S
Z2218555969	CC1=C2OCCCN2N=C1C(O)=O	182,18	C8H10N2O3
Z2217033030	NC1=C(N=NN1)C(=O)N1CCCC1	181,20	C7H11N5O
Z2216711607	NCCC(=O)NC1=CC(Cl)=CC(Cl)=C1	233,09	C9H10Cl2N2O
Z2568751802	COCC1=CSC(=C1)C(O)=O	172,20	C7H8O3S
Z2568751702	BrC1=CC2=C(OCCO2)N=C1	216,03	C7H6BrNO2
Z2515207600	NC(=O)C1=NC2=C(CCCN2)C=C1	177,21	C9H11N3O
Z2515207630	CN1N=CC(C2CCC2)=C1C(O)=O	180,21	C9H12N2O2
Z2515207554	OC(=O)C1=C2COCCC2=CC=C1	178,19	C10H10O3
Z2513333903	NC1=CC2=C(CCCCC2O)C=C1	177,25	C11H15NO
Z2512451323	CC1=CC2=C(N=CN2C=C1)C(O)=O	176,18	C9H8N2O2
Z99600018	CC1=CSC2=C(C#N)C(C)=CC(=O)N12	204,25	C10H8N2OS
Z99598988	CC1=CC(C)=C(C#N)C(=O)N1	148,17	C8H8N2O
Z198194122	CN(CC1=CC=CS1)C(N)=O	170,23	C7H10N2OS
Z46190592	CNCC(=O)NC1=NOC(C)=C1	169,18	C7H11N3O2
Z96091967	CC1=CC=NN1C1=CC=C(C=C1)C(O)=O	202,21	C11H10N2O2
Z55723242	CC1=C(C)N=C2C=C(C=CC2=N1)C(O)=O	202,21	C11H10N2O2
Z90664470	OC(=O)CC1=C(Br)C=C2OCCOC2=C1	273,08	C10H9BrO4
Z55993084	CC(N)C1=CC=C(C=C1)N1C=CN=C1	187,25	C11H13N3
Z208155046	CC1=CC(C)=C(CCC(O)=O)C=C1	178,23	C11H14O2
Z29177127	CC(C)C(=O)NC1=CC(=CC=C1)C(N)=O	206,25	C11H14N2O2
Z28296645	CCC(=O)NC1=CC2=C(C=C1)N=C(C)S2	220,29	C11H12N2OS
Z56754337	OC(=O)C12CCCC(CCC1)(C2)C(O)=O	212,25	C11H16O4
Z56964368	NCCC1=CC2=C(OCCO2)C=C1	179,22	C10H13NO2
Z44507989	Cl.CNCC1=NC2=C(C=CC=C2)C(=O)N1	225,68	C10H12ClN3O
Z56912006	OC(=O)CNC(=O)C1=CC=C(Br)O1	248,03	C7H6BrNO4
Z56913114	CN1C(O)=CC(C)=C(C#N)C1=O	164,16	C8H8N2O2
Z28292377	CC(=O)NC1=C(C)C(Cl)=CC=C1	183,64	C9H10ClNO
Z31480423	O=C(N1CCCC1)C1=CC=C(C=C1)C#N	200,24	C12H12N2O
Z31480727	FC1=CC(Cl)=C(C=C1)C(=O)N1CCCC1	227,66	C11H11ClFNO
Z56839652	OC(=O)C1COC2=C(O1)C=CC=C2	180,16	C9H8O4
Z56753990	CC1=C(C)CC(C(C1)C(O)=O)C(O)=O	198,22	C10H14O4

Z1428158834	OC(=O)C1=C(C=C(C=C1)C(F)(F)F)C#N	215,13	C9H4F3NO2
Z1428018535	NC1=CC=NN1CC1CC1	137,19	C7H11N3
Z1424669442	CCN1C=C(N)C(=N1)C(O)=O	155,16	C6H9N3O2
Z1421528826	OC(=O)C1=CC2=C(CCS(=O)(=O)C2)S1	232,27	C8H8O4S2
Z1416282601	OC(=O)CN1C=C(N=N1)C1CC1	167,17	C7H9N3O2
Z1143584382	CCC1N(CCNC1=O)C(=O)NC(C)C	213,28	C10H19N3O2
Z1328968520	CS(=O)(=O)C1=NC=CN1CC1=CSC=N1	243,30	C8H9N3O2S2
Z1407659631	CC(C)CC1(CN)CCC1	141,26	C9H19N
Z1407007505	O[C@H](C[C@@H]1CCCN1)C1=CC=CC=C1	191,27	C12H17NO
Z1217526242	CN1C=CC=C1C(=O)NC1=NNC=C1C	204,23	C10H12N4O
Z1398461994	CN1N=NC(C(O)=O)=C1C(F)(F)F	195,10	C5H4F3N3O2
Z1381260620	OC(=O)C1=C(Cl)C(=NN1)C1CC1	186,60	C7H7ClN2O2
Z959151860	O[C@@H]1CCC2=C1C=CC(F)=C2	152,17	C9H9FO
Z234897043	CC1=C(C)C=C(C=C1)C1CC1N	161,25	C11H15N
Z818727446	CC(CN)N1CCOCC1	144,22	C7H16N2O
Z401097872	CCC1CCCN(CC2=NN=C(C)O2)C1	209,29	C11H19N3O
Z803153704	NCCC1=C(C=CC=C1)C(F)(F)F	189,18	C9H10F3N
Z1217910516	CC(C)C(CN)C1=CC=CC=C1	163,26	C11H17N
Z1219738993	NC1CCN(C1)C1=CC=CC=C1	162,24	C10H14N2
Z1262237381	NCCC1=C(F)C=CC(Br)=C1	218,07	C8H9BrFN
Z763367910	CC(N)COC1=C(F)C=C(F)C=C1	187,19	C9H11F2NO
Z375692826	CC(C)C(=O)N(C)CC1=NOC(C)=C1	196,25	C10H16N2O2
Z223327076	CC(C)C(=O)NCC1=NN=C2CCCN12	208,27	C10H16N4O
Z104924088	CC(C)N1CCN(CC1)C1=NC=CC=N1	206,29	C11H18N4
Z1348289436	CC(=C)CN1C=CC(=O)C2=C1C=CS2	205,28	C11H11NOS
Z1267773545	CC(C)C1=C(C)SC(CN)=N1	170,27	C8H14N2S
Z2234631245	CNC1=NC(=CO1)C(F)(F)F	166,10	C5H5F3N2O
Z2065788535	NC1C2CCC1CC1=C(C2)C=CC=C1	187,29	C13H17N
Z90504169	C(NC1CCCC1)C1CCCO1	169,27	C10H19NO
Z1900548518	CC1=CN=C(CNC2=CC(C)=NC=C2)O1	203,25	C11H13N3O
Z2065464393	CCC1OCCC1C(O)=O	144,17	C7H12O3
Z1373414370	OCC1CCCN(C1)C1=C(F)C=CC=N1	210,25	C11H15FN2O
Z2050724987	CCC(O)C1=NC=C(F)C=C1	155,17	C8H10FNO
Z2044949019	CC(O)C1=NC=C(F)C=C1	141,15	C7H8FNO
Z2033459081	COC1=C(C=CC=C1)C1CCC(C)CN1	205,30	C13H19NO
Z1896858260	ClC1=CC(CCN2C=NC=N2)=CC(Cl)=C1	242,10	C10H9Cl2N3
Z145545120	ClC1=CNC(=C1)C(=O)N1CCNC(=O)C1	227,65	C9H10ClN3O2
Z1101435401	CC(C)C(=O)NC1CCCNC1	170,26	C9H18N2O
Z422952944	CN(CC(N)=O)C(=O)C1=C(Cl)SC(Cl)=C1	267,12	C8H8Cl2N2O2S
Z54226006	FC1=CC=CC(F)=C1C(=O)N1CCCC1	211,21	C11H11F2NO
Z247484750	CC1=C(OC=C1)C(=O)NC1=NN=CS1	209,22	C8H7N3O2S
Z54953371	CCOC1=C(C=CC=C1)C(N)=O	165,19	C9H11NO2
Z31432009	CC(NC(C)=O)C(=O)N1CCCCCC1	212,29	C11H20N2O2
Z324505866	CCS(=O)(=O)N1CCC(CC1)NC(C)=O	234,31	C9H18N2O3S
Z328695024	CC(C)N(C)C1=C2C=NN(C)C2=NC=N1	205,27	C10H15N5
Z285641686	C=CCNC(=O)C1=C2N=CC=CN2N=C1	202,22	C10H10N4O
Z1273089263	CCC(N)(C(O)=O)C1=CC=CC=C1	179,22	C10H13NO2
Z1310763003	CC1(C)CCN1C(=O)NC1=NN=CS1	212,27	C8H12N4OS
Z1318356921	COC1=CC=C(C=C1)C1=CC=C(CN)S1	219,30	C12H13NOS
Z1318268950	COC1=CC=C(C=C1)C1=CSC(CN)=C1	219,30	C12H13NOS
Z1247413608	CS(=O)(=O)N1CCC(O)CC1	179,23	C6H13NO3S
Z1278581710	CNC(C(O)=O)C1=CC=C(F)C=C1	183,18	C9H10FNO2
Z1037287770	CCN(C)C(=O)NC1=NN=C(S1)C1CC1	226,30	C9H14N4OS
Z803153714	CC(N1CCC(N)C1)C1=CC=CC=C1	190,29	C12H18N2
Z1373438018	CN(C1CCOC1)C1=C(F)C=CC=N1	196,23	C10H13FN2O
Z2313104779	COC1CN(CC2=C(F)C=NC=C2)C1	196,23	C10H13FN2O
Z2219019614	CC1=NOC=C1CNC1CCCCC1	194,28	C11H18N2O
Z1921387755	CC#CCN1CC(O)CC2=C1C=CC=C2	201,27	C13H15NO
Z287256168	CC(NC(=O)C1=NC(C)=CC=C1)C1CC1	204,27	C12H16N2O
Z228587528	O=C(C1CCCC1)N1CCCNCC1	196,29	C11H20N2O
Z1318357730	COCC(N)C1=CC=CO1	141,17	C7H11NO2
Z1259087088	CC1=CC=C(O1)C1CCCCN1	165,24	C10H15NO
Z434621834	COC(C(=O)N(C)C)C1=CC=CC=C1	193,25	C11H15NO2
Z280806616	CC(C1CC1)N(C)C(=O)C1CC1	167,25	C10H17NO
Z1575325610	NCC1CCC2=C1C=CC=C2	147,22	C10H13N
Z31190928	CC(C)NC1=NC=CC=N1	137,19	C7H11N3
Z1258578171	CC1=CC(=C(C)O1)C(C)(O)CN	169,22	C9H15NO2

Z1753051259	CCC1(O)CN(C1)C1=C(C=CC=N1)C#N	203,25	C11H13N3O
Z1918453451	CCC1(O)CN(C1)C(=O)C1=NN=C(C)S1	227,28	C9H13N3O2S
Z2065788225	CNC(=O)C1=C(Br)C(N)=CC=C1	229,08	C8H9BrN2O
Z1918542164	CCC1(O)CN(C1)C1=NC=C(Cl)C=N1	213,67	C9H12ClN3O
Z2045006645	CC1=NC2=C(N1)C=CC=C2C#N	157,18	C9H7N3
Z2044769537	CN1C=NC2=C1C=CN=C2	133,15	C7H7N3
Z2044770199	C1CCC(NC1)C1=NNC=C1	151,21	C8H13N3
Z1780230281	BrC1=CC=C(CNC(=O)C2=CC=CO2)O1	270,08	C10H8BrNO3
Z1220054876	NC(=O)CCN1N=C2C=CC=CN2C1=O	206,21	C9H10N4O2
Z274783176	COC1=C(N)C=C(C=C1)C(O)=O	167,16	C8H9NO3
Z420276166	CC1=C(SC=N1)C(=O)NCC1CCCC1	224,32	C11H16N2OS
Z1270387259	CC(C)(OC1=CC=CC=C1)C(O)=O	180,20	C10H12O3
Z1270384392		198,17	C10H8F2O2
Z1270358401	OC(=O)CC1CCC2=C1C=CC=C2	176,22	C11H12O2
Z1270358383	CN1C=C(C=N1)C1=NC(C)=C(S1)C(O)=O	223,25	C9H9N3O2S
Z1270207964	CC1=C(SC(=N1)C1CCCC1)C(O)=O	211,28	C10H13NO2S
Z1269638425	OC1CCCC2=CNN=C12	138,17	C7H10N2O
Z1268703748	CC1=C(C=CC(=C1)C(O)=O)N1C=NC=N1	203,20	C10H9N3O2
Z1267881762	COC1=C(SC(C)=C1Br)C(O)=O	251,09	C7H7BrO3S
Z2658867260	CC1(C)CCCC(N)(C1)C(O)=O	171,24	C9H17NO2
Z461898648	CN(C)C(=O)C1=CNN=C1	139,16	C6H9N3O
Z1127744301	NC(=O)C1CCC2=C1C=CC=C2	161,20	C10H11NO
Z2588063700	OC(=O)C1C2CCCCCCC12	168,24	C10H16O2
Z2588063726	CC1=NC2=C(O1)N=CC(Br)=C2	213,03	C7H5BrN2O
Z2587654857	OC1(CCNCC1)C1CC1	141,21	C8H15NO
Z271138028	OC(=O)CCC1=C(C=CC=C1)C#N	175,19	C10H9NO2
Z2582143599	CCC1=CNC(=O)C(=C1)C(O)=O	167,16	C8H9NO3
Z992753752	C1CC2=C(CN1)SC(=N2)C1=CC=CC=C1	216,30	C12H12N2S
Z1741974486	NC1=NC=NC(=C1)C(F)(F)F	163,10	C5H4F3N3
Z275177252	CC(C)NC(=O)C1=NN(C)C(=C1)C(O)=O	211,22	C9H13N3O3
Z2106601619	CN1N=C(C)C2=C1NC(=O)C=C2C(O)=O	207,19	C9H9N3O3
Z1741965991	CC1CCC2=C(C1)C(=NO2)C(O)=O	181,19	C9H11NO3
Z1813130067	CC1=C(C=CN1C1CC1)C(O)=O	165,19	C9H11NO2
Z2510258226	COC1=CC2=C(C=C1)C(=NS2)C(O)=O	209,22	C9H7NO3S
Z2510258278	COC1=CC2=C(C=C1)C(CCC2)C(O)=O	206,24	C12H14O3
Z2510258276	CC1=CC=C(S1)C1=C(C=NO1)C(O)=O	209,22	C9H7NO3S
Z2510258156	OC(=O)CC1=CC2=C(C=C1)N=CS2	193,22	C9H7NO2S
Z2510258214	CC(C)CC1=NC(C)=C(O1)C(O)=O	183,21	C9H13NO3
Z2380439254	CC1=C(C)CC(CC1)C(O)=O	154,21	C9H14O2
Z2379802699	CN1N=CC=C1C1NCCC2=C1N=CN2	203,25	C10H13N5
Z1269145581	COC1=C(C(O)=O)C(C)=CC=C1Br	245,07	C9H9BrO3
Z2379087683	COC1=NSC(=C1)C(O)=O	159,16	C5H5NO3S
Z1561884809	NC(=O)C1CCN(CC2CCC=CC2)C1	208,31	C12H20N2O
Z2350907435	CCC1C(=O)NC2=C1C=CC=C2	161,20	C10H11NO
Z2335632234	CN1C(C)=C(Br)C=C(C(O)=O)C1=O	246,06	C8H8BrNO3
Z1289592972	CN(CC(O)=O)C1CCC1	143,19	C7H13NO2
Z2327883869	CC1=C(C2CCCC(=O)N2)C(C)=NN1	193,25	C10H15N3O
Z2327883861	CC1(C)CCCC(C)(O)C1	142,24	C9H18O
Z2327883833		206,24	C12H14O3
Z1263529610	CNC1=NC2=C(C=C1)C=C(N)C=C2	173,22	C10H11N3
Z1213669627	CC1=CC2=C(C=C1)C(=O)C=C(O2)C(O)=O	204,18	C11H8O4
Z1188325566	CCN(C)C(=O)NCC1=CC(F)=CC=C1	210,25	C11H15FN2O
Z1173925785	CNC(=O)C1CC1C1=C(Cl)C(Cl)=CC=C1	244,12	C11H11Cl2NO
Z1263529549	CNC(=O)C1=CN=C(C)NC1=O	167,17	C7H9N3O2
Z1262691682	CC1=NC(O)=C(C)C(C)=N1	138,17	C7H10N2O
Z1262398434	CNC(=O)C1=C(N)C=CC=C1C	164,21	C9H12N2O
Z1741965065	CS(=O)(=O)C1=CC2=C(C=CN2)C=C1	195,24	C9H9NO2S
Z1262327477	CC(C)C1=NOC(=N1)C1=CC=C(N)C=C1	203,25	C11H13N3O
Z1889909664	COCC(O)C1=NC=CS1	159,20	C6H9NO2S
Z1889709962	CC(N1CCNCC1)C1=NC=C(C)S1	211,33	C10H17N3S
Z1271660882	CC1CCC(C1)NCCO	143,23	C8H17NO
Z1823334918	CC1=CC(CS(=O)C2=C(C)OC=C2)=NO1	225,26	C10H11NO3S
Z1527154441	CN(CCO)C(=O)C1=CC(C)=C(Cl)C=C1	227,69	C11H14ClNO2
Z1815149173	COC1=C(C=CC=C1)C(N)CN	166,22	C9H14N2O
Z1815149192	NCC(N)C1=CSC2=C1C=CC=C2	192,28	C10H12N2S
Z1778561006	CNCCC1CCOCC1	143,23	C8H17NO
Z1738061139	O=C1SC=NN1CC1=NOC=C1	183,19	C6H5N3O2S

Z1824511473	CC(=O)N1C[C@H](O)C[C@@H]1C(O)=O	173,17	C7H11NO4
Z2379203156	NC1=NCC2=C1C=CC=C2	132,17	C8H8N2
Z2070537303	CC1=C(N=C(O1)C1CC1)C(O)=O	167,16	C8H9NO3
Z1993522705	O=C1C=CNC(=N1)C1CCOCC1	180,21	C9H12N2O2
Z1891776137	C1CC(CN1)N1N=CC2=C1C=CC=C2	187,25	C11H13N3
Z198262914	CNC(=O)C1=CC2=C(NN=N2)C=C1	176,18	C8H8N4O
Z1945984127	OC(=O)CC1=CC2=C(NN=C2)C=C1	176,18	C9H8N2O2
Z1918018808	CC1=C(N=NN1CC1CCCC1)C(O)=O	209,25	C10H15N3O2
Z1918018307	CN1N=C(C)C2=C1NC=CC2=O	163,18	C8H9N3O
Z1918018438	CN1N=C(C=C1N)C1=CC=C(Cl)S1	213,68	C8H8ClN3S
Z1913660931	CN1N=C(C=C1N)C1=C(F)C=C(F)C=C1	209,20	C10H9F2N3
Z1839480564	ClC1=C(CC2CCCNC2=O)C=CC=C1	223,70	C12H14ClNO
Z1839481042	O=C1NCCCC1CC1=CC=CC=C1	189,26	C12H15NO
Z1262327505	C(NC1=NC=CC=C1)C1CCNC1	177,25	C10H15N3
Z1572855477	FC1=CC=C(NCC2=CSN=N2)C=C1	209,24	C9H8FN3S
Z1460223342	FC1=C(F)C=C(NC2CCNC2=O)C=C1	212,20	C10H10F2N2O
Z729726738	CC1=NOC(NC(=O)C2CCCCC2)=N1	209,25	C10H15N3O2
Z1139696203	CC(C)N1CCCC(CN2C=NC=N2)C1	208,31	C11H20N4
Z2429435052	CC(C)(O)CN1CC[C@H](N)C1	158,25	C8H18N2O
Z1755532218	CC(C)C1=NC2=C(CC(CC2)C(N)=O)N1	207,28	C11H17N3O
Z2582962531	OC(=O)C1CC(C1)OC(F)F	166,12	C6H8F2O3
Z1552027015	CC1=CC=C(OCC2=NC(N)=NO2)C=C1	205,22	C10H11N3O2
Z1378382208	ClC1=C(C=CN=C1)N1CCS(=O)(=O)CC1	246,71	C9H11ClN2O2S
Z1526503435	CC1=C(C)C(=CC(=C1)S(C)(=O)=O)C(O)=O	228,26	C10H12O4S
Z1516394948	NC1=CN=C(OCCO)C=C1	154,17	C7H10N2O2
Z1511494807	OC(=O)C1CCCC1C(O)=O	158,15	C7H10O4
Z1495385259	Cl.CC1=CC(C)=C(CCN)C(C)=C1	199,72	C11H18ClN
Z85956704	CNCC1=CC(F)=C(OC)C=C1	169,20	C9H12FNO
Z1741966332	CCN1C=C(C(C)N)C(C)=N1	153,23	C8H15N3
Z1492781275	CC1=C(C=CC(=C1)C#N)C(O)=O	161,16	C9H7NO2
Z31112041	C(NC1=NC=CN=C1)C1=CC=CS1	191,25	C9H9N3S
Z1446854759	CC1(C)C(=O)NC2=C1C=C(C=C2)C(O)=O	205,21	C11H11NO3
Z57683743	CC1=NNC(=O)C1CCC(O)=O	170,17	C7H10N2O3
Z85923411	C(NC1=CN=CC=C1)C1=CC=CO1	174,20	C10H10N2O
Z1250132399	CN(C)CCN1C=CC2=C1C=C(N)C=C2	203,29	C12H17N3
Z636406392	FC1=CC(NCC2=CC=CO2)=CC=C1	191,21	C11H10FNO
Z32014822	CN(C)C(=O)C1=CC(C)=C(C)C=C1	177,25	C11H15NO
Z1650040241	CCC(=O)NC1CCNC1	142,20	C7H14N2O
Z1263529632	CN(C)C(=O)CN1C=C(N)C=N1	168,20	C7H12N4O
Z1945700460	NC1=NOC(=N1)C1=CC2=C(CCC2)C=C1	201,23	C11H11N3O
Z1891774786	CC1=C(C)N2CCNCC2=N1	151,21	C8H13N3
Z1945708091	CN1C(=O)CC2=C1N=CC(Br)=C2	227,06	C8H7BrN2O
Z352704742	CC(O)CNC(=O)C1=C(F)C=C(Br)C=C1	276,11	C10H11BrFNO2
Z1916655429	CNC1=NN=C(C=C1)C1=CC(F)=CN=C1	204,21	C10H9FN4
Z1907760164	OC(=O)C1=C(C(F)F)N2N=CC=C2N=C1	213,14	C8H5F2N3O2
Z1907760167	CC1=NN=C(O1)C1=C(N)C=CC(F)=C1	193,18	C9H8FN3O
Z1895741677	CC1=NC2=C(CCCC2C(O)=O)S1	197,25	C9H11NO2S
Z1889997414	CC1=NC(CC(O)=O)=C(Br)C=C1	230,06	C8H8BrNO2
Z1889996343	OCC1=NOC(=C1)C1=CC(F)=CC=C1	193,18	C10H8FNO2
Z1889917206	OC(=O)CCN1CCCS1(=O)=O	193,22	C6H11NO4S
Z1889902457	CC(C)(C1CCC(O)CC1)C(O)=O	186,25	C10H18O3
Z594970960	CC1CCC(CC1)NC(=O)C1=NSN=C1	225,31	C10H15N3OS
Z28880966	CC(NC(=O)C1=CC=CS1)C1=CC=CS1	237,34	C11H11NOS2
Z600428394	NC1=NN=C(O1)C1=CC2=C(CCC2)C=C1	201,23	C11H11N3O
Z600428384	CC1=NN2C(N=C(C)C=C2C)=C1C(O)=O	205,22	C10H11N3O2
Z600428386	CC1=NN2C=CC=NC2=C1C(O)=O	177,16	C8H7N3O2
Z133804144	CC(NS(C)(=O)=O)C1=CN=CC=C1	200,26	C8H12N2O2S
Z85887069	OC(=O)CNC(=O)C1=CC=C(Br)S1	264,09	C7H6BrNO3S
Z64780602	CC1=NN(CCO)C(C)=C1	140,19	C7H12N2O
Z240137278	O=S(=O)(N1CCCC1)N1CCOCC1	220,29	C8H16N2O3S
Z372499004	CC1=CC=C(O1)C(=O)NC1=CNN=C1	191,19	C9H9N3O2
Z228589198	CN(C)C1=NC2=C(S1)C=C(S2)C(O)=O	228,28	C8H8N2O2S2
Z29672803	CC1=NN=C(NC(=O)C2=NC=CC=C2)S1	220,25	C9H8N4OS
Z1234028446	CNS(=O)(=O)C1=CNC=N1	161,18	C4H7N3O2S
Z410633222	CC1=NN=C(NC(=O)N2CCOCC2)S1	228,27	C8H12N4O2S
Z1258578315	CC1=NC(N2CCCC2)=C(C=C1)C#N	187,25	C11H13N3
Z1162470566	CC1CCCC2=C1NC(=O)C(=C2)C#N	188,23	C11H12N2O

Z990231828	CC(=C)CN1CCC(CC1)C1=CNN=C1	205,31	C12H19N3
Z295892696	CC(C)NC(=O)C1=C2N=CC=CN2N=C1	204,23	C10H12N4O
Z875024178	CC1=CC(=O)NC(=N1)C1=CC=C(N)C=C1	201,23	C11H11N3O
Z217038356	CC1CN(CCO1)C(=O)CN1C=CC=N1	209,25	C10H15N3O2
Z466628048	CC1=CSC(CNC(=O)C2=NNC=C2)=N1	222,27	C9H10N4OS
Z32015800	CNC(=O)C1=CC(=CC=C1)S(C)(=O)=O	213,25	C9H11NO3S
Z2072621991	FC(F)OCC(=O)N1CC2CCCC2C1	219,23	C10H15F2NO2
Z2587654879	CC(O)C1=C(C)N=CC=C1	137,18	C8H11NO
Z1040397258	CC1(C)COCCN1CC1=CN=CC=C1	206,29	C12H18N2O
Z2379564057	CC1=NSC(=N1)N1CC=CC1	167,23	C7H9N3S
Z2492802465	COCC(N)C1CCOCC1	159,23	C8H17NO2
Z2465620163	COCC1=NN=C2CNCCN12	168,20	C7H12N4O
Z2442103067	COCCN1CCNC(C)(C)C1	172,27	C9H20N2O
Z31791472	O=C(CC1=CC=CS1)NC1=CN=CC=C1	218,27	C11H10N2OS
Z1233341893	CC(C)C(C)C(=O)N(C)C1=NC=CC=C1	206,29	C12H18N2O
Z2574915591	NC1CCCC1CC1=NC=CC=C1	176,26	C11H16N2
Z2574915593	CN1N=C(C)C=C1CC1CCCC1N	193,29	C11H19N3
Z1444214858	CC(C)(NC(=O)C1=CC=NC=C1)C(O)=O	208,22	C10H12N2O3
Z1172068241	OC(=O)CCC1CCCO1	144,17	C7H12O3
Z2213893336	CC1=CC(=O)NC(=C1)C(O)=O	153,14	C7H7NO3
Z2213893369	CN1C=NC2=C1C(=CC(=O)N2)C(O)=O	193,16	C8H7N3O3
Z2213893376	CC1CNC(=O)CN(C1)C1CC1	168,24	C9H16N2O
Z2205958606		206,25	C11H14N2O2
Z2186882035	CC1=CN2C(S1)=NC(C)=C2C#N	177,23	C8H7N3S
Z2186882188	CC1=C(N)C2=NC=NN2C=C1	148,17	C7H8N4
Z118333670	O=C1CCCCN1C1=CC=CC=C1	175,23	C11H13NO
Z2182115414	CC1COC2=C1C=C(C=C2)C(O)=O	178,19	C10H10O3
Z1878408220	CC(=C)CN1C=C(Br)C(C)=CC1=O	242,12	C10H12BrNO
Z32014367	CN(C)C(=O)C1=CC=CO1	139,15	C7H9NO2
Z274762944	OC(=O)CN1C(=O)OC2=C1C=C(Cl)C=C2	227,60	C9H6ClNO4
Z29659913	COC1=C(OC)C=C(NC(C)=O)C(C)=C1	209,25	C11H15NO3
Z285139200	CC(C)C(=O)NC1=CC(CN)=CC=C1	192,26	C11H16N2O
Z278101766	CC1=C(F)C=C(C=C1)C#N	135,14	C8H6FN
Z256708488	CCOC1=C(C=CC=N1)C(O)=O	167,16	C8H9NO3
Z1828858406	COC(C)(C)C1=NC(=NO1)C1=CC=CS1	224,28	C10H12N2O2S
Z363104186	CNC(=O)C1CCCN(C1)C(=O)C(C)C	212,29	C11H20N2O2
Z1816725432	NCC1(O)CCN(CC2=CC=CC=C2)C1	206,29	C12H18N2O
Z1259339838	CC(N)C1=C(C)N=C(C)S1	156,25	C7H12N2S
Z995389448	CCN1C(=O)ON=C1C1=C(C)C=CC=C1	204,23	C11H12N2O2
Z1259339743	COC1=C(C=CC=C1)C(C)(C)O	166,22	C10H14O2
Z1250132810	CN1CCC(CN)C1C1=CC=CS1	196,31	C10H16N2S
Z228574062	CN(C)C(CN)C1=CC=C(Cl)C=C1	198,69	C10H15ClN2
Z1354416068	CC(=O)N1CC2=C(C1)C=C(N)C=C2	176,22	C10H12N2O
Z1342208350	C1CNC2=C(N1)C=CC=N2	135,17	C7H9N3
Z1336457497	CNC1=C(N=CC=C1)N1CCCC1	177,25	C10H15N3
Z1816233707	CNC(=O)C1=NC(=CC=C1)C(O)=O	180,16	C8H8N2O3
Z1816233763	CC1=C(SC=N1)C1=CNC=C1C(O)=O	208,24	C9H8N2O2S
Z1815156932	CN1C=CC(CC(O)=O)=N1	140,14	C6H8N2O2
Z2897268424		159,14	C6H9NO4
Z1815155424	NC(=O)C1(CCOCC1)N1CCCC1	198,27	C10H18N2O2
Z1675346324	CN(C1CC(O)C1)C1=NC(C)=NC(C)=C1	207,28	C11H17N3O
Z1247413461	CS(=O)(=O)N1CCCC(CO)C1	193,26	C7H15NO3S
Z364368134	CC(=O)NC1=C(C)C(=CC=C1)C(O)=O	193,20	C10H11NO3
Z1331708376	O=C(NCC1=CNN=N1)C1CCC=CC1	206,25	C10H14N4O
Z449369146	OC(=O)CCC1=CC=C(Cl)S1	190,64	C7H7ClO2S
Z445226250	CC(N)(C(O)=O)C1=CC=C(Cl)C=C1	199,63	C9H10ClNO2
Z328695000	CC(C)N(C)C1=CC(C)=NC2=NC=NN12	205,27	C10H15N5
Z220564202	C1CC(CCN1)C1=CNC2=C1C=CC=C2	200,29	C13H16N2
Z432066788	CC1=C(F)C=C(C=C1N)C(N)=O	168,17	C8H9FN2O
Z29672805	CC(C)C(=O)NC1=NN=C(C)S1	185,25	C7H11N3OS
Z415636820	CC1=C(N=C(O1)C1=CC=CS1)C(O)=O	209,22	C9H7NO3S
Z71176766	OC(=O)C1=C(N=CC=C1)N1CCCCC1	206,25	C11H14N2O2
Z397585784	OC(=O)C1=CSC(CC2=CC=CC=C2)=N1	219,26	C11H9NO2S
Z442033408	O=C(NCC1=CC=CS1)C1=NNC=C1	207,25	C9H9N3OS
Z240297184	CN1C=CC(NC(=O)C2=C(C)OC=C2)=N1	205,22	C10H11N3O2
Z992716870	CN1N=CC(C2=NC(=NO2)C2CC2)=C1N	205,22	C9H11N5O
Z119654718	CN1C=CC(NC(=O)C2=C(C)C=CO2)=N1	205,22	C10H11N3O2

Z951367936	CC1=C(N)C(=CC(F)=C1)C(O)=O	169,16	C8H8FNO2
Z364411884	CCC(=O)NC1=CC(Cl)=C(C=C1)C(N)=O	226,66	C10H11ClN2O2
Z220784950	CC1=C(OC=C1)C(=O)N1CCNC(=O)C1	208,22	C10H12N2O3
Z916919192	CN(C(C)=O)C1=C(C=CC=C1F)C(O)=O	211,19	C10H10FNO3
Z1868430567	OC(C1CCCCC1)C(O)=O	158,20	C8H14O3
Z1868316830	OC(=O)C1CC=CCC1C(=O)NC1CC1	209,25	C11H15NO3
Z1695906701	CC1=NN(C=C1C(O)=O)C1CCOC1	196,21	C9H12N2O3
Z2574910420	OC(=O)CN1CCN(C2CC2)C1=O	184,20	C8H12N2O3
Z1224459567	CN(C)C(=O)CC1(CCCC1)C(O)=O	199,25	C10H17NO3
Z815264062	CN1C=C(C(O)=O)C(=N1)C1=CC=NC=C1	203,20	C10H9N3O2
Z2327226200	O=C1COCC(CC2=CC=CC=C2)N1	191,23	C11H13NO2
Z1446243816	NC1CCOC2(CCC2)C1	141,21	C8H15NO
Z401964204	CC1CCCCC1NCC1=CNN=C1C	207,32	C12H21N3
Z2087745281	COC1=C(C=CC=C1)[C@H]1CCC[C@@H](C)N1	205,30	C13H19NO
Z2205998814	CN1CCC2OCCNC2C1	156,23	C8H16N2O
Z3244905268	Cl.Cl.Cl.NC1CCN(CCN2CCOCC2)C1	308,67	C10H24Cl3N3O
Z927323342	O=C(C1CC1)N1CCCNCC1	168,24	C9H16N2O
Z57148445	COC1=CC=C(C=C1)C1=NN=C(N)S1	207,25	C9H9N3OS
Z1263602345	COC1=NC2=C(C=CC=C2)C(CN)=C1	188,23	C11H12N2O
Z1415893830	NC1=NN(CC2=CC=NC=C2)C=N1	175,20	C8H9N5
Z1350606444	OC(=O)CCC1=CSC2=C1C=CC=C2	206,26	C11H10O2S
Z285782452	CNC1=C(C=CC=C1)S(C)(=O)=O	185,24	C8H11NO2S
Z1318516260	CC(=O)N1CCC(C)(C1)C(O)=O	171,20	C8H13NO3
Z2216303104	OCC1=CC2=C(ON=C2)C=C1	149,15	C8H7NO2
Z2189170996	OC(=O)C1=C(CC2=CC=CC=C2)SC=N1	219,26	C11H9NO2S
Z2690260832	N#CC1=C2OCCOC2=CS1	167,18	C7H5NO2S
Z26317437	NC(=O)CN1C=C(Br)C=CC1=O	231,05	C7H7BrN2O2
Z53825414	CCS(=O)(=O)N1CCCC1C(O)=O	207,24	C7H13NO4S
Z85890240	CC(NC(=O)C1=CC=CO1)C(O)=O	183,16	C8H9NO4
Z147579704	OC(=O)C1=CC2=C(OC1)C=CC(Cl)=C2	210,61	C10H7ClO3
Z85886788	OC(=O)CNC(=O)C1CCCC1	171,20	C8H13NO3
Z234898187	CC1=C(C=CC=C1N)C(O)=O	151,17	C8H9NO2
Z90122509	NC(=O)N1CCC(CC1)C(O)=O	172,18	C7H12N2O3
Z25080630	CNC(=O)COC1=C(Br)C=C(F)C=C1	262,08	C9H9BrFNO2
Z384361454	CC1=NOC(=C1)C(=O)NCC1CCOC1	210,23	C10H14N2O3
Z44585799	CC1=CC(NC(=O)N2CCCC2)=CC=C1	204,27	C12H16N2O
Z44585777	O=C(NC1=CC=CC=C1)N1CCCC1	190,25	C11H14N2O
Z220616840	NC1=CN=C(C=C1)N1CCOCC1	179,22	C9H13N3O
Z26548083	CC(C)C(=O)NC1=CC(=CC=C1)C#N	188,23	C11H12N2O
Z30841068	BrC1=CC(=CC=C1)C(=O)NC1CC1	240,10	C10H10BrNO
Z359419748	CC1=C(SC2=C1C(C)=CC=N2)C(O)=O	207,25	C10H9NO2S
Z1863617804	COCC1=CC(=O)NC(=N1)N1CCCC1	209,25	C10H15N3O2
Z1530301542	CCC1=CSC(=N1)C1=CNC=N1	179,24	C8H9N3S
Z1180112116	NC1=C(C=CC=C1)C1CCNCC1	176,26	C11H16N2
Z1874937386	OC1=CC(=O)NC(=N1)C(F)(F)F	180,09	C5H3F3N2O2
Z1869801232	CC1(CCCNC1=O)C(O)=O	157,17	C7H11NO3
Z1222331407	CC(C)(CO)NC1=NC=C(Br)C=C1	245,12	C9H13BrN2O
Z1127695826	CC(C)NC(=O)CC1=C(Br)C=CC=C1	256,14	C11H14BrNO
Z165304624	CC1=NC(COC2=C(F)C=CC=C2)=NO1	208,19	C10H9FN2O2
Z320990506	OC(CN1C=CN=C1)C1=CC(Br)=CC=C1	267,13	C11H11BrN2O
Z103739202	CN1CCN(CCO)CC1	144,22	C7H16N2O
Z1098687825	CC1=CC(=NO1)C(=O)N1CCCC1(C)C	208,26	C11H16N2O2
Z241200732	CC(C)C(C)NC(=O)C1=CC=C(C)N1C	208,31	C12H20N2O
Z351222172	OC(=O)C(N1CCCC1)C1=CC=CC=C1	205,26	C12H15NO2
Z1172115933	CC1=C(C=CC(=N1)C1=CC=CO1)C(O)=O	203,20	C11H9NO3
Z649583112	CNCC1=NN=C(C)N1C	140,19	C6H12N4
Z1385328783	CNC(=O)N(C(C)C1=CC=CO1)C1CC1	208,26	C11H16N2O2
Z1526453856	CN1C=CN=C1C1CCNC1	151,21	C8H13N3
Z1262395923	COC1=C(C=CC=C1)C(C)CN	165,24	C10H15NO
Z1270087714	CC1CN(CC(C)O1)C1=NC(C)=NS1	213,30	C9H15N3OS
Z1250132637	CCC(C)(N)C1=NC=CS1	156,25	C7H12N2S
Z1166181228	CC1CN(C)CCN1C(=O)C1=NSN=C1	226,30	C9H14N4OS
Z340498698	CC(C)N(C1CC1)C(=O)C1=C(C)OC=C1	207,27	C12H17NO2
Z324712988	CC(C)N(C1CC1)C(=O)C1=CC=CO1	193,25	C11H15NO2
Z2507919901	C1CC(CO1)C1=NC=CN1	138,17	C7H10N2O
Z1259273141	OC(=O)C1(CCC1)C(=O)NC1CC1	183,21	C9H13NO3
Z2492800383	COCC1=C(N)N=CC=C1	138,17	C7H10N2O

Z2471547875	CN1C(=O)C=CC2=C1CCCC2N	178,24	C10H14N2O
Z295228412	CN(C)C(=O)C1=CN=C(C=C1)C(O)=O	194,19	C9H10N2O3
Z1982493923	CC1(CCC(=O)NC1)C(O)=O	157,17	C7H11NO3
Z1973465514	O=C1NN=C(C=C1)C1CC1	136,15	C7H8N2O
Z1971703350	CN1N=C(CN2CCCC2)C=C1C(O)=O	209,25	C10H15N3O2
Z90122431	CC1=CC(C)=C(C(O)=O)C(=O)N1	167,16	C8H9NO3
Z1962222453	CN1N=CC(C(O)=O)=C1O	142,11	C5H6N2O3
Z32014557	CN(C)C(=O)CC1=C(Cl)C=CC=C1F	215,65	C10H11ClFNO
Z32014670	CN(C)C(=O)CC1=C(Cl)C=CC=C1Cl	232,10	C10H11Cl2NO
Z274740244	CC1=C(N2C=C(Br)C=CC2=N1)C(O)=O	255,07	C9H7BrN2O2
Z1945698150	CN1C(C)=NC2=C1C=C(C=C2)C(O)=O	190,20	C10H10N2O2
Z1983079687	FC(F)(F)C1=CC(=O)NC2=C1C=NN2	203,12	C7H4F3N3O
Z381357590	C1CNC(C1)C1=CC=CN1	136,20	C8H12N2
Z380844598	NCC(O)C1=CC=C(F)C=C1	155,17	C8H10FNO
Z741016936	CC1=CC(NC(=O)C2=CSN=N2)=CC=C1	219,26	C10H9N3OS
Z234855772	CC(C)N1N=CC2=C1NC(=O)C=C2C	191,23	C10H13N3O
Z56767623	C1CN(CCO1)C1=NC2=C(S1)C=CC=C2	220,29	C11H12N2OS
Z227830218	CN(C)C1=NC=C(N)C=C1	137,19	C7H11N3
Z56761814	CC(=O)NC1=C2SC(C)=NC2=CC=C1	206,26	C10H10N2OS
Z56760165	CC1(C)CC2=C(C1)C(=NS2)C(N)=O	196,27	C9H12N2OS
Z55293719	CN(C)C(=O)NC1=CC=C(Cl)C=C1	198,65	C9H11ClN2O
Z235352019	OC(=O)CN1N=C2CCCCCN2C1=O	211,22	C9H13N3O3
Z2692093998	CN1N=C(O)C2=C1CNCC2	153,19	C7H11N3O
Z1741979488	OC(=O)CCC1=CC(F)=C(Cl)C=C1	202,61	C9H8ClFO2
Z2692093605	NC(C1CCCO1)C(O)=O	145,16	C6H11NO3
Z2365130785	C[C@@]1(O)C[C@@H](C1)NC1=C(F)C=CC=N1	196,23	C10H13FN2O
Z2273972081	CC1CN(CC(=O)N1)C1=C(F)C=CC=N1	209,22	C10H12FN3O
Z1373445602	CN1CCN(CC1=O)C1=C(F)C=CC=N1	209,22	C10H12FN3O
Z234897483	OC(=O)C1=C(CN2CCOCC2)C=CO1	211,22	C10H13NO4
Z1821397701	CC1=C(SC(CC2CC2)=N1)C(O)=O	197,25	C9H11NO2S
Z409511958	OC1CCN(CC1)C(=O)NC1=NN=CS1	228,27	C8H12N4O2S
Z1816507673	CN(C)C(C(O)=O)C1=C(Cl)C=CC=C1	213,66	C10H12ClNO2
Z1699518303	CN1N=CN=C1NCC1=NC=C(C)C=C1	203,25	C10H13N5
Z1836338757	CN1C=C(C(O)=O)C(=N1)C1=CC=C(Cl)S1	242,68	C9H7ClN2O2S
Z1836338785	OC(CC1CCCCC1)C(O)=O	172,22	C9H16O3
Z1741957000	OC(=O)C1=C(C=CC=C1F)C(F)(F)F	208,11	C8H4F4O2
Z1729240147	CC1=CC(OCC2=NOC=C2)=CC(C)=N1	204,23	C11H12N2O2
Z1259041087	CC1(C)CCNC(=O)CC1	141,21	C8H15NO
Z1258992517	O=C1NN=C2CCCCN12	139,16	C6H9N3O
Z56823545	OC(=O)CNC(=O)C1=C(Cl)C=C(Cl)C=C1	248,06	C9H7Cl2NO3
Z1258578430	CC1=C(C#N)N2C=CSC2=N1	163,20	C7H5N3S
Z56801375	O=C1NCCC2=C1C=CC=C2	147,18	C9H9NO
Z1137726171	CNCC1=C2NC=CC2=CC=C1	160,22	C10H12N2
Z1259341112	N#CC1=CC2=C(OCCO2)C=C1	161,16	C9H7NO2
Z1259340040	CC1=CC(C(O)=O)=C(C=C1)N1CCCC1	205,26	C12H15NO2
Z1259339939	CC1=CC(CC(O)=O)=NO1	141,13	C6H7NO3
Z1259273386	CN1N=C(C=C1C1CC1)C(O)=O	166,18	C8H10N2O2
Z1259273264	CN1C=C(C=N1)C1=CC(=CN=C1)C(O)=O	203,20	C10H9N3O2
Z1259162160	CC(=O)NC(C)(C1CC1)C(O)=O	171,20	C8H13NO3
Z1601639621	FC1=C(NC2CCCOCC2)N=CC=C1	210,25	C11H15FN2O
Z1262254278	CC(C)NC(=O)CN1CCCNCC1	199,30	C10H21N3O
Z1262252889	CN1CCC(CCN)CC1	142,25	C8H18N2
Z1262253026	NC1CCCC2=C1C=CO2	137,18	C8H11NO
Z339881488	CC(C)N1CCN(CC1)C(=O)N(C)C	199,30	C10H21N3O
Z2687190967	CC1(C)CC(=O)NCCN1	142,20	C7H14N2O
Z1730522163	CC1CN(C(C)=O)C2=C(O1)C(F)=CC=C2	209,22	C11H12FNO2
Z2681277106	CC1=C(OC(=C1)C(F)F)C(O)=O	176,12	C7H6F2O3
Z1171979267	CC(=O)NCC1=CC=C(O1)C(O)=O	183,16	C8H9NO4
Z1171978818	COC1=C(SC=C1)C(O)=O	158,17	C6H6O3S
Z844852064	CN(C1CC1)C(=O)C1=CC=C(C)NC1=O	206,25	C11H14N2O2
Z25637733	CC1=C(C)C(OCC(N)=O)=C(C)C=C1	193,25	C11H15NO2
Z1770193507	OC(=O)C1(CC2=CC=C(F)C=C2)CC1	194,21	C11H11FO2
Z1769859569	CC1=CC(=NN1C1=NC=CC=C1)C(O)=O	203,20	C10H9N3O2
Z1768428971	CN1C(C(CC1=O)C(O)=O)C1=CC=CS1	225,26	C10H11NO3S
Z1677760062	CC(C)N(C)C(=O)NC1=C(F)C=NC=C1	211,24	C10H14FN3O
Z1762997287	COC1=CC(Br)=C(C(O)=O)C(N)=C1	246,06	C8H8BrNO3
Z1753013037	CC(N)CS(=O)(=O)C1=CC=C(C)C=C1	213,30	C10H15NO2S

Z1741785925	CC1CCNC(C1)C(N)=O	142,20	C7H14N2O
Z319891284	CC1=CC(=NO1)C1CCCN1S(C)(=O)=O	230,28	C9H14N2O3S
Z1723549274	CCC1=C(CC)N=C(N)N=N1	152,20	C7H12N4
Z1603606287	CC1=CC=C(S1)C(=O)NC1(CC1)C(O)=O	225,26	C10H11NO3S
Z1444213486	CN1N=CC=C1C(=O)NC(C)(C)C(O)=O	211,22	C9H13N3O3
Z295847540	C(N1CCCCC1)C1=NC(=NO1)C1CC1	207,28	C11H17N3O
Z1217910521	CC(CN)CN1N=C(C)C=C1C	167,26	C9H17N3
Z1809263534	COCCN1CCCNCC1=O	172,23	C8H16N2O2
Z1899615150	CC(C)CN1C=NC2=C(C=CN2)C1=O	191,23	C10H13N3O
Z1333717721	CC1=NN2CCNCC2=N1	138,17	C6H10N4
Z2467540742	OCC(O)CN1C=CC=C1	141,17	C7H11NO2
Z1335657421	O=C1CNCCN1C1CC1	140,19	C7H12N2O
Z1245580422	CC1CCCN(CCN)C1	142,25	C8H18N2
Z1998104358	CN(C)C(=O)N1CCOCC11CCOC1	214,27	C10H18N2O3
Z1238540099	ClC1=C(NC2CCNCC2)C=CC=C1	210,71	C11H15ClN2
Z2235330350	CC1=C(CCO)N=CC=C1	137,18	C8H11NO
Z2689946992	OC(=O)C1=CC(=CN=C1)N1CCCC1=O	206,20	C10H10N2O3
Z2158481117	COC1=C(C=CC(=C1)N(C)C)C(O)=O	195,22	C10H13NO3
Z2111525940	N#CC1=CN(CC2=CC=CC=C2)N=N1	184,20	C10H8N4
Z2096087693	OC1CCC2=C1N=CC(Br)=C2	214,06	C8H8BrNO
Z217102788	C1CNC2=C(C1)N=CC=C2	134,18	C8H10N2
Z2168277541	O=C1NCCC1CC1=CC=CC=C1	175,23	C11H13NO
Z2160887406	COC1=C(N)C=C(F)C(F)=C1	159,14	C7H7F2NO
Z1415893980	CCC1(CCCC1)C(O)=O	142,20	C8H14O2
Z1245580461	CC1CCN(CCN)CC1	142,25	C8H18N2
Z1360063124	CN(C)C(=O)C1=C(Br)C=CO1	218,05	C7H8BrNO2
Z57036273	CN(C)C1=C(N)C=C(C=C1)C(F)(F)F	204,20	C9H11F3N2
Z1162778919	CCC1=NC(CN2C=CC=CC2=O)=NO1	205,22	C10H11N3O2
Z818732104	CC(N)CN1CCOCC1	144,22	C7H16N2O
Z1696870012	NC1CCCCC1NC1=NC=CN=C1	192,27	C10H16N4
Z1891776227	NC1CCOC2(CCOC2)C1	157,21	C8H15NO2
Z2510258210	COCC1=C(SC=N1)C(O)=O	173,19	C6H7NO3S
Z373773422	CN1C=C(NC(=O)CN2C=CC=N2)C=N1	205,22	C9H11N5O
Z910841006	CCC(=O)NC1=C(C=C(C)C=C1)C(O)=O	207,23	C11H13NO3
Z234897203	NC1=C(CN2CCC(O)CC2)C=CC=C1	206,29	C12H18N2O
Z234896705	NCC1=CC(F)=C(Br)C=C1	204,04	C7H7BrFN
Z234853700	CC1=NC(=O)N(CC(O)=O)C(C)=C1	182,18	C8H10N2O3
Z31190999	CC(C)NC1=C2C=CSC2=NC=N1	193,27	C9H11N3S
Z111781394	OC(=O)C1=CC(=NN1)C1CC1	152,15	C7H8N2O2
Z30271924	CNC(=O)C(C)OC1=CC=C(C=C1)C#N	204,23	C11H12N2O2
Z240297434	CN1C=CC(NC(=O)C2=CC(C)=NO2)=N1	206,21	C9H10N4O2
Z969560526	CC1=NC2=C(N1)N=CC=C2	133,15	C7H7N3
Z969559596	NC(C(O)=O)C1=CC(Br)=CC=C1	230,06	C8H8BrNO2
Z314449926	CCN(C1CC1)C(=O)C1CCC=CC1	193,29	C12H19NO
Z382738034	CC1=NOC(CN2CCOC(C)(C)C2)=C1	210,28	C11H18N2O2
Z228574258	CN(C)C(CN)C1=C(F)C=CC=C1F	200,23	C10H14F2N2
Z137705484	CC1CCCN(CC2=C(Cl)SN=N2)C1	231,74	C9H14ClN3S
Z90507765	C(NC1CCCCCC1)C1=CN=CC=C1	204,32	C13H20N2
Z241827180	CN1CCN(CC1)C(=O)C1=NOC(C)=C1	209,25	C10H15N3O2
Z992717376	C1CN(CCN1)C1=NN=CC=C1	164,21	C8H12N4
Z31490283	CC1=C(SC=C1)C(=O)NCC1CCCO1	225,31	C11H15NO2S
Z744843394	CN1CCC(CN)C2=C1C=CC=C2	176,26	C11H16N2
Z818727322	NCC1CCCN(CC2=CC=CC=C2)C1	204,32	C13H20N2
Z227830236	NC1CCN(CC1)C1CC1	140,23	C8H16N2
Z1268961924	CCC1CN2CCCC2CN1S(C)(=O)=O	232,34	C10H20N2O2S
Z2327226238	CC1(C)CC(C(O)=O)C2=C(O1)C=CC=C2	206,24	C12H14O3
Z2327224817	C1CC(CO1)C1=NC2=C(N1)C=CC=C2	188,23	C11H12N2O
Z2305986609	CN1C(C)=CC2=C(C(N)=NN2)C1=O	178,20	C8H10N4O
Z1988459453	CN1N=C(C=C1C)C(O)C1=CN=CC=C1	203,25	C11H13N3O
Z2301499848	OC(=O)CC1=CC(=CC=C1)C1CC1	176,22	C11H12O2
Z31191155	CC(C)NC1=C(C=CC=N1)C#N	161,21	C9H11N3
Z54505513	NC(=O)CN1CCC(O)CC1	158,20	C7H14N2O2
Z1267773753	NC1=C(Cl)C=CC(=C1)N1CCNC1=O	211,65	C9H10ClN3O
Z1267773689	CC(C)C1CCCCC1O	142,24	C9H18O
Z1267773677	NC1=C(C=C2CCCC2=N1)C#N	159,19	C9H9N3
Z1266933830	COC1=C(OC)C=C2N=C(N)NC2=C1	193,21	C9H11N3O2
Z1263714141	CCC(C)(N)C1=NC(=O)C2=C(CCC2)N1	207,28	C11H17N3O

Z33546774	NC(=O)CC1=C(Cl)C=CC=C1Cl	204,05	C8H7Cl2NO
Z18083670	NC(=O)COC1=CC(=CC=C1)C#N	176,18	C9H8N2O2
Z2379767231	CC(C)C1=CSC(=N1)C1=NN=CN1	194,26	C8H10N4S
Z355729118	CC1CN(CC2=CC(C)=NO2)C(C)CO1	210,28	C11H18N2O2
Z2234866028	CCN(C)C(=O)NC1=NSN=C1	186,23	C6H10N4OS
Z286761296	CCN1CCCC1C(=O)N(C)C	170,26	C9H18N2O
Z645688832	CC1=CC=C(CNC2=NC=CN=C2)S1	205,28	C10H11N3S
Z1101640411	CC1CN(C(C)CO1)C(=O)C1=CSC=N1	226,29	C10H14N2O2S
Z1029460366	COC1=CC(CC2CCCN2)=CC=C1	191,27	C12H17NO
Z216242078	CC1CN(CC2=CC(C)=NO2)CCO1	196,25	C10H16N2O2
Z2010010168	CN1N=CC=C1C1CC2CCC(C1)N2	191,28	C11H17N3
Z1250132610	NC1=CC(=CC=C1)N1CCCCCC1	190,29	C12H18N2
Z1401333862	CN1C=CC(NCC2=CC=CC=C2)=N1	187,25	C11H13N3
Z275730348	CC(N1CCC(O)CC1)C1=NC(C)=NO1	211,27	C10H17N3O2
Z2352608837		213,28	C11H19NO3
Z2434504713	CC1CC(CO1)NC1=C(F)C=CC=N1	196,23	C10H13FN2O
Z1102068247	CC(C)(C(N)=O)C1=CC=C(F)C=C1	181,21	C10H12FNO
Z2093301849	C[C@@H](CO)N(C)C1=NC=C(F)C=C1	184,21	C9H13FN2O
Z2285175561	CCN(C1COC1)C(=O)C1=C(F)C=CS1	229,27	C10H12FNO2S
Z1983897532	CC1=NC=C(CNC2=CC(F)=CN=C2)S1	223,27	C10H10FN3S
Z1855969199	FC1=CCCN(C1)S(=O)(=O)C1COC1	221,25	C8H12FNO3S
Z1779277596	OC1CCN(CC2=CC(F)=CC(F)=C2)C1	213,23	C11H13F2NO
Z2379564100	CC(=O)N1CC(C1)NC1=NC(C)=NS1	212,27	C8H12N4OS
Z279623012	CN(C)S(=O)(=O)N1CCCC1	178,25	C6H14N2O2S
Z2234185613	CC1=CC2=C(C=C1)C(=O)C(=CN2)C(O)=O	203,20	C11H9NO3
Z2010655200	CC(C)CCN1C=C(C=N1)C#N	163,22	C9H13N3
Z2055186690	CC(C)CC(=O)N1C[C@@H](C)[C@@H](C1)C(N)=O	212,29	C11H20N2O2
Z1642373092	COC1=C2NC=CC2=CC=C1	147,18	C9H9NO
Z2301499695	CN1N=C(C=C1C)C(O)C1CC1	166,22	C9H14N2O
Z1103241017	CN1C=C(C=N1)C1CCCN1CC(C)=C	205,31	C12H19N3
Z2240988426	CC(N)(CC1=CC=CC=C1)C1CC1	175,28	C12H17N
Z103740620	CC(C)N1CCCC(CO)C1	157,26	C9H19NO
Z57033899	COC1=CC=C(C=C1)C1CCCN1	177,25	C11H15NO
Z57987855	NC1=CC=NN1CC1=CC=C(Br)S1	258,14	C8H8BrN3S
Z19735578	CC(OC1=CC=CC=C1)C(N)=O	165,19	C9H11NO2
Z803145422	CC(C)C1=C(SC=C1)C(O)=O	170,23	C8H10O2S
Z215640462	CCN1N=NN(C1=O)C1=CC=CS1	196,23	C7H8N4OS
Z228464414	O=C(CN1C=NC(=N1)C#N)N1CCCC1	205,22	C9H11N5O
Z763030026	NCC1=C(CO)C=CC=C1	137,18	C8H11NO
Z752989144	NC(=O)CN1C=CC(N)=N1	140,15	C5H8N4O
Z111834298	OC(=O)COC1=C(Br)C=CC=C1	231,05	C8H7BrO3
Z90423275	OC1CCN(CC1)C(=O)CCC(O)=O	201,22	C9H15NO4
Z235354749	Cl.CN1N=C(C)C2=C1N=CC(=C2)C(O)=O	227,65	C9H10ClN3O2
Z235354601	CN1C=CC(=C1)S(=O)(=O)N1CCNCC1	229,30	C9H15N3O2S
Z235354543	CN1N=CC(C(O)=O)=C1N1C=CC=C1	191,19	C9H9N3O2
Z85895203	CN(CC(O)=O)C(=O)C1=CC=CO1	183,16	C8H9NO4
Z56792417	NC1=CC(=CC=C1)S(=O)(=O)N1CCCC1	226,29	C10H14N2O2S
Z57455032	OC(=O)COC1=C(Cl)C=CC(Cl)=C1	221,03	C8H6Cl2O3
Z32013668	CN(C)C(=O)C1=CC=C(C=C1)S(C)(=O)=O	227,28	C10H13NO3S
Z332685442	CNC(=O)CNC(=O)C1=COC(Br)=C1	261,08	C8H9BrN2O3
Z212182648	CC(C)NC(=O)C1=CC=C(NC(C)=O)S1	226,29	C10H14N2O2S
Z57472940	OC(=O)C1CN(C(=O)C1)C1=CC=CC=C1	205,21	C11H11NO3
Z367450878	CC1=CC(C(O)=O)=C(C)N1	139,15	C7H9NO2
Z30533876	CC(=O)NC1=CC=C(OC(F)(F)F)C=C1	219,16	C9H8F3NO2
Z31408124	O=C(NCC1=CC=CO1)C1=NC=CN=C1	203,20	C10H9N3O2
Z1747218443	CNC1CCC(CC1)NC(C)=O	170,26	C9H18N2O
Z2470036257	CC1(C)CCC2=C(O1)C=CC(N)=C2	177,25	C11H15NO
Z1429867185	COC1=C2C=CNC2=CC=C1	147,18	C9H9NO
Z2467454495	CC(C)(CO)N1C=CN=C1	140,19	C7H12N2O
Z2467454603	CN1N=CC=C1C1COCCN1	167,21	C8H13N3O
Z2466617812	FC1=CC2=C(OCCN2)C(F)=C1	171,15	C8H7F2NO
Z2465620036	CS(=O)(=O)C1=C2C=CN=CC2=C(N)C=C1	222,26	C10H10N2O2S
Z2465619816	CCC1=CC=C(C=C1)C1=CNC(N)=N1	187,25	C11H13N3
Z2442102917	OC(=O)CN1N=C2CCCCN2C1=O	197,19	C8H11N3O3
Z228588628	OC(=O)C1(CCC1)C1=CC(F)=CC=C1	194,21	C11H11FO2
Z2418615685	NC(=O)CN1C=C(N)C=CC1=O	167,17	C7H9N3O2
Z2418193688	O=C1NC=NC2=C1COCC2	152,15	C7H8N2O2

Z2418193668	CC1=CC2=C(C=C1)N=NNC2=O	161,16	C8H7N3O
Z1263811695	CC1COCCN1CCN	144,22	C7H16N2O
Z1359429532	NCC(N1C=CC=N1)C1=CC=C(Cl)C=C1	221,69	C11H12ClN3
Z1263811762	NCC1=C(F)C=CC(F)=C1	143,14	C7H7F2N
Z1267882038	NCC1=CC(OC2CCOCC2)=NC=C1	208,26	C11H16N2O2
Z1245664663	COCC1=CC(=CC=C1)C#N	147,18	C9H9NO
Z360056084	C[C@@H](O)C1=CC2=C(CCC2)C=C1	162,23	C11H14O
Z1263529837	CC(N)C1=CC(C)=C(F)C(C)=C1	167,23	C10H14FN
Z1350618224	CN(C)C1CCCC(N)C1	142,25	C8H18N2
Z1416200823	CNC1CCC(CO)CC1	143,23	C8H17NO
Z1318268746	NCCC1=C(F)C=CC(F)=C1	157,16	C8H9F2N
Z235338177	OC(=O)CC1=CN(N=C1)C1=CC=CC=C1	202,21	C11H10N2O2
Z56899122	NC(CC(O)=O)C1=CC=C(Cl)C=C1	199,63	C9H10ClNO2
Z56895681	OC(=O)CN1C=NC2=C(C=CC=C2)C1=O	204,19	C10H8N2O3
Z212045094	CS(=O)(=O)NC1CCNCC1	178,25	C6H14N2O2S
Z104343432	CC1=C(Cl)C=CC(C(O)=O)=C1N	185,61	C8H8ClNO2
Z235362573	NC1=CC=C(C=C1)C1=CN2CCCC2=N1	199,26	C12H13N3
Z235362259	N#CC1=CC=C(CN2C=NC=N2)C=C1	184,20	C10H8N4
Z235362689	CC1=C(C=CO1)C1=NN=C(N)O1	165,15	C7H7N3O2
Z1250080886	CC1=NN(C(C)=C1)C1=NC=C(N)C=C1	188,23	C10H12N4
Z1222331415	OC(=O)C1=CC2=C(OCCO2)C(Br)=C1	259,06	C9H7BrO4
Z2255112549	OC1C(=O)NCC11CCOCC1	171,20	C8H13NO3
Z2216303314	CC1=C(CCO)N=CC=N1	138,17	C7H10N2O
Z1416200822	FC1=C2NCCOC2=CC=C1	153,16	C8H8FNO
Z1269702389	CC1CC(N)CN1C1=CC=CC=C1	176,26	C11H16N2
Z1346370640	CC(C)C1=NC(=NO1)C1CCCN1	181,24	C9H15N3O
Z1357827251	NC1CCN(C1)C1=CC(Cl)=CC=C1	196,68	C10H13ClN2
Z1626920982	CN1CCC(C)(CO)CC1	143,23	C8H17NO
Z1262252914	CC1=C(SC=C1)C1=NNC(N)=C1	179,24	C8H9N3S
Z183726212	CC1CCCCN1C(=O)C1CCC=CC1	207,32	C13H21NO
Z1259339735	CN(C)CC1=NC(=NO1)C1(N)CCCC1	210,28	C10H18N4O
Z1492980784	CC(C)N1C=NN=C1C1=C(Cl)C=CC=C1	221,69	C11H12ClN3
Z2240999284	FC(F)(F)C1=CC=C(N1)C#N	160,10	C6H3F3N2
Z2240999254	CC(CO)N1C(C)=NC2=C1C=CC(N)=C2	205,26	C11H15N3O
Z2238897966	CN1CCN(CC1)C1=CC=C(S1)C(O)=O	226,29	C10H14N2O2S
Z1255452173	COC1=C2C=CC(N)=CC2=CC=N1	174,20	C10H10N2O
Z2238508600	O=C1NCC2=C1N=CC=C2	134,14	C7H6N2O
Z2236664125	CC1(CCC=CC1)C(O)=O	140,18	C8H12O2
Z1269232519	CC1=CC(Br)=C(CC(O)=O)C=C1	229,07	C9H9BrO2
Z1416201130	C[C@@H]1CNCC(O1)C(N)=O	144,17	C6H12N2O2
Z1333717732	OC(=O)C1CC11CCCC1	140,18	C8H12O2
Z1077667680	CN1C=C(NCC2=CC=CC(C)=C2)C=N1	201,27	C12H15N3
Z823455846	FC1=C(CNC2CCOCC2)C=CC=C1	209,26	C12H16FNO
Z1142870735	O=S1(=O)CCC(C1)N1CCCNCC1	218,32	C9H18N2O2S
Z2255112034		165,19	C9H11NO2
Z2255110079	CC(C1CCC(=O)N1)C(O)=O	157,17	C7H11NO3
Z1993523194	NC1CCCC11CCOCC1	155,24	C9H17NO
Z1250089980	NC1=NC(CO)=C(Cl)C=C1	158,59	C6H7ClN2O
Z1245646686	CC1=C(C)C(=O)C2=C(N1)C(F)=CC=C2F	209,20	C11H9F2NO
Z1992316287	CCNC1=C(C)C=CC=N1	136,20	C8H12N2
Z1925961476	CC(C)C1CC(O)C2=C1C=CC=C2	176,26	C12H16O
Z1255634499	CC1CCC(CC1)N(C)S(=O)(=O)C1CC1	231,35	C11H21NO2S
Z1820007572	CCC1=C(N)C=NN1CC(C)C	167,26	C9H17N3
Z927402744	NC1(CCCCC1)C(=O)NCC=C	182,27	C10H18N2O
Z1601554890	CCS(=O)(=O)C1=CC=C(CN)C=C1	199,27	C9H13NO2S
Z1796855094	CC1CCCN1S(=O)(=O)NC1(C)CCC1	232,34	C10H20N2O2S
Z1618272617	C1CN(CC=C1)C1=C2NC=NC2=NC=N1	201,23	C10H11N5
Z1649893125	CCC1CC(CC(=O)N1)C(O)=O	171,20	C8H13NO3
Z1649677765	N#CC1=C(N=CC=C1)C1CC1	144,18	C9H8N2
Z1708089989	CC(C)S(=O)(=O)C1=CC(=CC=C1)C(O)=O	228,26	C10H12O4S
Z1704732763	CN(C)C1=NC=C(O1)C(O)=O	156,14	C6H8N2O3
Z1704732444	COC1=C(C=NC=C1)C#N	134,14	C7H6N2O
Z85923433	CC1=NC2=C(C(=O)NN2)C(C)=C1	163,18	C8H9N3O
Z1696861466	CN1C=C(C=N1)C1NC(=O)CCC1N	194,24	C9H14N4O
Z1696844698	CC1=NN2C(N=C(C)C=C2C(O)=O)=C1Cl	225,63	C9H8ClN3O2
Z1998636703	NC1=CC(OCC2=CC=CC=C2)=NC=C1	200,24	C12H12N2O
Z1998636256	CCC(O)(C(O)=O)C1=CC(Cl)=CC=C1	214,65	C10H11ClO3

Z1993522714	CC1(CCCO1)C1=NOC(N)=C1	168,20	C8H12N2O2
Z2028214403	CN(CC(N)=O)C(=O)C1=CC(N)=CC=C1	207,23	C10H13N3O2
Z2010051560	COC1(CCOCC1)C(O)=O	160,17	C7H12O4
Z2009848113	CCC1=C(C=NO1)C(O)=O	141,13	C6H7NO3
Z1626921298	NC1=NN=C2CCCCN12	138,17	C6H10N4
Z1622825203	CN1C=NC2=C1N=CC(=C2)C(O)=O	177,16	C8H7N3O2
Z1582262995	OCCOC1CCNCC1	145,20	C7H15NO2
Z458890528	NC(=O)C1CCC(=O)NC1	142,16	C6H10N2O2
Z1575304399	CC1=CC2=C(NC=C2)C(=O)N1	148,17	C8H8N2O
Z1420098164	NC(=O)C1CCN(C1)C(=O)C1=CCCC1	208,26	C11H16N2O2
Z402607752	O=C(CN1CCCNC1=O)N1CCCC1	211,27	C10H17N3O2
Z1230587767	CCN1CCCN(CC1)C(=O)NCC=C	211,31	C11H21N3O
Z253340200	CCN1N=CC=C1NC(=O)C1CCCC1	207,28	C11H17N3O
Z254451326	O=C(C1CC1)N1CCCC2=C1C=CC=N2	202,26	C12H14N2O
Z364579532	CC1COCCN1C(=O)C1=NC=CN=C1	207,23	C10H13N3O2
Z1313483439	CC1=CC=C(C=C1)C1=NC(CN)=CO1	188,23	C11H12N2O
Z1164527757	CN1C=C(NCC2=CC=CS2)C=N1	193,27	C9H11N3S
Z1267882019	NCCC1=CC2=C(OCC2)C=C1	163,22	C10H13NO
Z1267882022	CC(C)C1=NOC(CN)=C1	140,19	C7H12N2O
Z2510258284	CC1COC2=C(C=CC=C12)C(O)=O	178,19	C10H10O3
Z1685775949	CCC1(CC)CCNC1=O	141,21	C8H15NO
Z1675167167	CN(C)C1=CN=C(C=C1)C(O)=O	166,18	C8H10N2O2
Z1675167132	CC1=C(C(O)=O)C(N)=CC(Cl)=C1	185,61	C8H8ClNO2
Z2235810234	OC(=O)CC1=C(N=CS1)C1=CC=CC=C1	219,26	C11H9NO2S
Z2235810230	CN1C=NC2=C1C(CC(=O)N2)C(O)=O	195,18	C8H9N3O3
Z2235790652	COC1=CC(=CC=C1)N1C=C(N)N=N1	190,21	C9H10N4O
Z2235386876	COC1=C(C=CC=C1)C1=NN=C(N)S1	207,25	C9H9N3OS
Z2230911478	OCC1CCNC(=O)CC1	143,19	C7H13NO2
Z2230911328	CC1CC2=C(CC1C)N=CN2	150,23	C9H14N2
Z2218556094	CCN1CC(O)CNC1=O	144,17	C6H12N2O2
Z2217033031	OC(=O)C1=CC(F)=C(OC(F)F)C=C1	206,12	C8H5F3O3
Z2106594212	OC(=O)C1=NN(C=C1Br)C1CCCC1	259,10	C9H11BrN2O2
Z2216711597	OC(=O)C1=C(OC=N1)C1CC1	153,14	C7H7NO3
Z2216711606	CCN1N=CC(=N1)C(O)=O	141,13	C5H7N3O2
Z2216711604	CC1=CC2=C(C=C1)N=C(C=C2)C(O)=O	187,20	C11H9NO2
Z57352018	OC(=O)C1=CC(=CC=C1)N1CCCC1	191,23	C11H13NO2
Z2568757981	CN1C=C(N=N1)C(C)(C)O	141,17	C6H11N3O
Z2568751883		191,27	C12H17NO
Z2568751838	COC1=C(OC)C=C2C(=O)NC=CC2=C1	205,21	C11H11NO3
Z2523459919	COCC1=NC(N)=CC=N1	139,16	C6H9N3O
Z2521982363	CC1=CC(C(O)=O)=C(C)N1C1=NOC=C1	206,20	C10H10N2O3
Z1741972881	CC1=CC(F)=CC(C)=C1C(O)=O	168,17	C9H9FO2
Z2513335975	CC(CC(O)=O)N1CCCC1=O	171,20	C8H13NO3
Z2512942961	O=C1NCCC1C1CCCCC1	167,25	C10H17NO
Z1255450742	OC(=O)C1CCN1C1CCCC1	169,22	C9H15NO2
Z733898624	CC(C)NC(=O)C1=CC(C)=NN1C	181,24	C9H15N3O
Z104378222	CC1=NC2=C(C(N)=C(S2)C(N)=O)C(C)=C1	221,28	C10H11N3OS
Z94599194	OC(=O)CC1=CSC(=N1)C1=CN=CC=C1	220,25	C10H8N2O2S
Z96091883	OCC1=CC=C(C=C1)N1C=CC=N1	174,20	C10H10N2O
Z208331700	CC1=C(F)C=C(C=C1)C(O)=O	154,14	C8H7FO2
Z234898195	NC(=O)N1CCCNCC1	143,19	C6H13N3O
Z33546529	CN(C)S(=O)(=O)C1=CC(=CC=C1)C(N)=O	228,27	C9H12N2O3S
Z30271161	CNC(=O)COC1=C(C)C=C(Cl)C=C1C	227,69	C11H14ClNO2
Z53834531	COC1=CC=C(C=C1)C#N	133,15	C8H7NO
Z46191128	CNCC(=O)NC1=CC=C(C)C=C1	178,24	C10H14N2O
Z56881200	OC(=O)CNC(=O)C1=CC(F)=CC=C1	197,17	C9H8FNO3
Z56839661	OC(=O)C1=CC2=C(OCCO2)C=C1	180,16	C9H8O4
Z56756098	[NH4+].COC1=C(Cl)C=CC(=C1)C([O-])=O	203,62	C8H10ClNO3
Z56762560	CC1(O)C2CC3CC(C2)CC1C3	166,26	C11H18O
Z414477942	CN(C)S(=O)(=O)NC1CCCCNC1=O	235,30	C8H17N3O3S
Z1403325251	CNC1=C(C=CC(Cl)=C1)C(=O)N(C)C	212,68	C10H13ClN2O
Z1407672962	OC(=O)C1CCCC2=C1C=NN2	166,18	C8H10N2O2
Z1407007551	C1CNC2=C(NC1)C=NC=C2	149,20	C8H11N3
Z975826158	NC1=NC(=CN=C1)N1CCCC1	164,21	C8H12N4
Z1405445662	CC1=NNC(CC(O)=O)=C1	140,14	C6H8N2O2
Z1374778829	CC1CCN(C1)C1=NC=CC(=C1)C(O)=O	206,25	C11H14N2O2
Z1365534328	CCN1N=NC(C(O)=O)=C1C(F)(F)F	209,13	C6H6F3N3O2

Z411697334	CCN1C=C(NC(=O)N2CCCC2)C=N1	208,27	C10H16N4O
Z162834430	CNC(C)C1=CC2=C(O1)C=CC=C2	175,23	C11H13NO
Z1222285094	COC1=C(C=CC=C1)N1CCC(CN)C1	206,29	C12H18N2O
Z1238477897	COCC1=C(C=CC=C1)C#N	147,18	C9H9NO
Z1250132474	CC(CC1=C(F)C=CC=C1)C(O)=O	182,19	C10H11FO2
Z993967038	CC(C)NC1CCNCC1	142,25	C8H18N2
Z90530641	CC1CCCCC1NCC1=NC=CC=C1	204,32	C13H20N2
Z1171716843	CN(C)C1CCCCC1N	142,25	C8H18N2
Z2143644460	C1CC(CN1)C1=CC=CO1	137,18	C8H11NO
Z1918541965	CCC1(O)CN(C1)C1=C(Cl)C=NC=N1	213,67	C9H12ClN3O
Z1878647755	CC1CC(C)(C)CC1NCC1=NC=CS1	224,37	C12H20N2S
Z90503761	CC1=C(CNC2CCCCC2)C(C)=NO1	208,31	C12H20N2O
Z1815903106	CC1=C(Br)C(CNC2CCOC2)=CC=C1	270,17	C12H16BrNO
Z1415893880	COCCC1=NC2=C(O1)C=CC(N)=C2	192,22	C10H12N2O2
Z1918541761	CCC1(O)CN(C1)C1=NC(=CC=C1)C#N	203,25	C11H13N3O
Z1263811691	CC(C)(N)C1=CC=C(F)C=C1	153,20	C9H12FN
Z1817978303	CC(=O)N1CCCC2(CCCCC2)CC1	209,33	C13H23NO
Z599664892	BrC1=CN(CC(=O)NC2CCCC2)N=C1	272,15	C10H14BrN3O
Z1127236484	C(NC1=CN=CC=C1)C1=CSN=N1	192,24	C8H8N4S
Z984691402	CC(=O)NC1=CC(=NN1)C1=C(C)C=CS1	221,28	C10H11N3OS
Z30847309	FC1=C(F)C=C(NC(=O)C2CCC2)C=C1	211,21	C11H11F2NO
Z30272492	CNC(=O)C(C)OC1=CC=C(Cl)C=C1	213,66	C10H12ClNO2
Z409533506	BrC1=C(NC(=O)N2CCCC2)C=CC=C1	269,14	C11H13BrN2O
Z1024440952	C1CC1NC1=NC(=NO1)C1=CC=CC=C1	201,23	C11H11N3O
Z239173696	CNC(=O)C1=CC2=C(N=C1)N(C)N=C2C	204,23	C10H12N4O
Z1126744743	CC1=NN(C(N)=C1)S(=O)(=O)C1=CC=CS1	243,30	C8H9N3O2S2
Z33546775	NC(=O)CC1=CC(=CC=C1)C(F)(F)F	203,16	C9H8F3NO
Z994937532	CC1=CC(NC(=O)N2CCC2)=NO1	181,20	C8H11N3O2
Z1318516263	CC1=CC=C(O1)C1CS(=O)(=O)CCN1	215,27	C9H13NO3S
Z1318515156	CNC(C)C1=CC=C(C=C1)S(C)=O	197,30	C10H15NOS
Z991556972	OC(=O)C1CCCN2C=CN=C12	166,18	C8H10N2O2
Z1318147519	CNC(C(O)=O)C1=C(F)C=C(F)C=C1	201,17	C9H9F2NO2
Z1318147455	CC1=NC(C(O)=O)=C2CCCCN12	180,21	C9H12N2O2
Z1318147452	CC1=C(C(O)=O)C(C)=CC(=O)N1	167,16	C8H9NO3
Z29693728	O=C(NCC1=CC=CS1)C1=CC=CO1	207,25	C10H9NO2S
Z1295543903	CC1=CC2=C(C=C1)C(CCC(O)=O)=CN2	203,24	C12H13NO2
Z208333304	O=C(N1CCCCC1)N1CCOCC1	198,27	C10H18N2O2
Z177087780	CN1CCN(CC1)C(=O)N1CCOCC1	213,28	C10H19N3O2
Z1274167323	CC(C(C)C(O)=O)N1N=C(C)C=C1C	196,25	C10H16N2O2
Z1984960549	COC(C)(C)C(=O)NC1=CC(F)=CN=C1	212,22	C10H13FN2O2
Z2219509018	CC1CN(CC2=CC(=CC=C2)C#N)C1C	200,29	C13H16N2
Z1258578173	COC1=C(C)C=CC(CCN)=C1	165,24	C10H15NO
Z279819030	CN(CC1=CC=NC=C1)C1=NC=CN=C1	200,25	C11H12N4
Z1415893978	C(NC1CCCNC1)C1=CC=CC=C1	190,29	C12H18N2
Z1416282822	COCC1=NN2CCCC(N)C2=N1	182,23	C8H14N4O
Z2034464797	CN(CC1CCCO1)C1=NC=C(F)C=C1	210,25	C11H15FN2O
Z1166114668	C1CCC(C1)NC1CCNCC1	168,28	C10H20N2
Z32014663	CN(C)C(=O)C1=C(C)C(C)=CC=C1	177,25	C11H15NO
Z2177153690	OCC1=CC(F)=C(OC(F)F)C=C1	192,14	C8H7F3O2
Z1838500084	CC1OCCCC1C(=O)NC1=CNN=C1	209,25	C10H15N3O2
Z1267773819	OCCNC1CCC2=C1C=CC=C2	177,25	C11H15NO
Z1791178849	O=C(CC1=CCCCC1)NC1=CN=NS1	223,29	C10H13N3OS
Z2065788086	COC1=C2N=CNC2=CC(Br)=C1	227,06	C8H7BrN2O
Z2065788380	CCC1=NC(C(O)=O)=C(C)O1	155,15	C7H9NO3
Z1260623529	FC1=CC(F)=C(C(=O)NC2CC2)C(F)=C1	215,18	C10H8F3NO
Z2039477470	BrC1=C2C(=O)NCC2=CC=C1	212,05	C8H6BrNO
Z2033454315	COC1=C(C=C(Cl)C=C1N)C(N)=O	200,62	C8H9ClN2O2
Z1270640437	O=C1CC2(CN1)CCCNC2	154,21	C8H14N2O
Z1270210792	NC1=NN=C(CC2CC2)O1	139,16	C6H9N3O
Z1270210778	NC(=O)CCN1C=CC(N)=N1	154,17	C6H10N4O
Z57173821	CC(NC(=O)C1=C(Cl)C=CC=C1)C(O)=O	227,64	C10H10ClNO3
Z1269638510	NC1=NNC(=C1)C1CCOCC1	167,21	C8H13N3O
Z1269638437	NC1=NN(CC(=O)N2CCOCC2)C=C1	210,24	C9H14N4O2
Z1268152423	CC(C)NC(=O)C1CCCC(N)C1	184,28	C10H20N2O
Z666291262	CC1=CN2C=CC=NC2=N1	133,15	C7H7N3
Z1262254283	NCC1=CN=C(C=C1)N1CCCC1	177,25	C10H15N3
Z1262250860	NC1=NN(CC2=CN=CC=C2)C=N1	175,20	C8H9N5

Z1262246150	COC1=C(C=CC=C1)C1=NNC(N)=C1	189,22	C10H11N3O
Z1262246109	OCC1=CN=C(O1)C1=CC=CO1	165,15	C8H7NO3
Z2658864411	CC1=CN2C=CN=C2C(=C1)C(O)=O	176,18	C9H8N2O2
Z2658864334	CC1(C)C(=O)NC2=C1C=C(CO)C=C2	191,23	C11H13NO2
Z2630874436	OC(=O)CC1CCCS1(=O)=O	178,20	C6H10O4S
Z2588063790	OC(=O)C1=CC(=CNC1=O)C(F)(F)F	207,11	C7H4F3NO3
Z680888866	CC1=NOC2=C1C=C(Br)C=N2	213,03	C7H5BrN2O
Z2574917679	CN1C(=O)SC2=CC(N)=C(C)C=C12	194,25	C9H10N2OS
Z2522871853	OC(=O)CN1C=C(C=N1)C1CC1	166,18	C8H10N2O2
Z275169912	CCN1C=C(C(O)=O)C(C)=N1	154,17	C7H10N2O2
Z1741969315	CN1C=CC2=C1C=CC(=C2)C(O)=O	175,19	C10H9NO2
Z1741967210	COC1=CC(C(O)=O)=C(Cl)C=C1	186,59	C8H7ClO3
Z275169940	CN1C=C(Cl)C(=N1)C(O)=O	160,56	C5H5ClN2O2
Z2510259389	OC(=O)C(C1CC1)C1=CC=C(Cl)C=C1	210,66	C11H11ClO2
Z2301685460		157,17	C7H11NO3
Z2510258318	OC(=O)C1=C2NCCN2N=C1	153,14	C6H7N3O2
Z1891772637	CC1(CNC(=O)C1)C(O)=O	143,14	C6H9NO3
Z2510258100	NC1=NC=CC(CC(O)=O)=C1	152,15	C7H8N2O2
Z1741959630	NC1=NC=CC(=C1)C(F)(F)F	162,12	C6H5F3N2
Z1255375308	OC(=O)C1=NN2CCOCC2=C1	168,15	C7H8N2O3
Z2380439330	CN1N=C(C=C1C)C(O)C1=CSC(C)=C1	222,31	C11H14N2OS
Z274502046	OC(=O)C1CCC(C1)C(O)=O	158,15	C7H10O4
Z2379802697	CS(=O)(=O)C1=CC=C(CO)S1	192,25	C6H8O3S2
Z2379087743	CC1=NN(C(C)=C1)C1=CN=C(C=C1)C#N	198,23	C11H10N4
Z2379087665	COC1=CC2=NC=C(N2C=C1)C(O)=O	192,17	C9H8N2O3
Z2379087811	COC1=C(Br)C=C2C(=O)NC=NC2=C1	255,07	C9H7BrN2O2
Z2372279536	CC1=C2C(O)C(=O)NC2=CC=C1	163,18	C9H9NO2
Z2327883947	CC1=CC(=O)NC(=N1)C1=CC=CS1	192,24	C9H8N2OS
Z2327883709	CN1C=CC(CCC(O)=O)=C1	153,18	C8H11NO2
Z1263529650	CNS(=O)(=O)C1=CC(=CC=C1)C(C)N	214,28	C9H14N2O2S
Z1263529614	NC1CCN(CC1)C1=NC=C(C=C1)C#N	202,26	C11H14N4
Z1080986230	CCC1=NON=C1NC(=O)C1=CC=CS1	223,25	C9H9N3O2S
Z1134990241	CC1=NC(CN2C=CC(C)=CC2=O)=CS1	220,29	C11H12N2OS
Z2679906873	CC1=CC(=NN1C1CCOCC1)C(O)=O	210,23	C10H14N2O3
Z2658864472	OCC1=CN=NN1C1=C(Cl)C=CC=C1	209,63	C9H8ClN3O
Z90526999	CC(O)CNC1CCCCC1	157,26	C9H19NO
Z1262327442	CC1=CSC(=N1)C1=CC(N)=CC=C1	190,26	C10H10N2S
Z1889909940	NC1CCN(C1)C1=CC(Br)=CC=C1	241,13	C10H13BrN2
Z1889909876	CC(N1CCNCC1)C1=NC(C)=CS1	211,33	C10H17N3S
Z1889709955	NC1=CN=C(OC2CCCC2)C=C1	178,24	C10H14N2O
Z1881620500	OCC1CC2=C(C1)C=CC=C2	148,21	C10H12O
Z1869852043	NCC1CCCCN1C1CC1	154,26	C9H18N2
Z1860991704	NCCC(O)C1=CC(Cl)=C(Cl)C=C1	220,09	C9H11Cl2NO
Z1860991707	COC1(CO)CCOCC1	146,19	C7H14O3
Z1849039889	CCC1=C(CC)C(N)=CC=C1	149,24	C10H15N
Z1839486549	CCC1=C(N)C=NN1C1=C(C)C=CC=C1	201,27	C12H15N3
Z1836338703	COC1=C(CN)C(Cl)=CC=C1	171,62	C8H10ClNO
Z1827898537	C(NC1CCNC1)C1=CC=CC=C1	176,26	C11H16N2
Z1258578979	CC(CC1=CC=CS1)C(O)=O	170,23	C8H10O2S
Z1720603241	CCS(=O)(=O)C1=C(C)OC=C1	174,21	C7H10O3S
Z296139642	CN(C)C(=O)C1=CSC(=N1)C1=CC=CO1	222,26	C10H10N2O2S
Z2238508578	OC(=O)C1(CC2=CC(Cl)=CC=C2)CC1	210,66	C11H11ClO2
Z2327131593	CN1N=CC=C1C1=CC(N)=C(C)C=C1	187,25	C11H13N3
Z2469657408	CN1C=C(C=N1)C1=CNC=C1	147,18	C8H9N3
Z2160887425	CC1=CC=C(C=C1)C1=NNC(=O)C=C1C	200,24	C12H12N2O
Z1020680398	CC1=CC(=CC(C)=C1)C(O)CN	165,24	C10H15NO
Z1874937351	CN1N=CC=C1CC(O)=O	140,14	C6H8N2O2
Z1837073433	COC1=C2N=CC(C)=C(N)C2=CC=C1	188,23	C11H12N2O
Z1137725943	CCNCC1=C2C=CNC2=CC=C1	174,25	C11H14N2
Z1795818230	CN1CC(=O)NC2=C1C=CC(Br)=C2	241,09	C9H9BrN2O
Z1762997349	CN1C=C(C=N1)C1=NN(C)C=C1C(O)=O	206,21	C9H10N4O2
Z1737940727	CN1C=C(C=N1)C1=CNC=C1C(O)=O	191,19	C9H9N3O2
Z2206895891	CC1=NC=NC(CN2CCC=C(F)C2)=C1	207,25	C11H14FN3
Z2234186172	C1CC(CN1)C1=NC2=C(O1)C=CC=C2	188,23	C11H12N2O
Z940781212	BrC1=C(CNC2=NC=CN=C2)SC=C1	270,15	C9H8BrN3S
Z56120255	CNS(=O)(=O)C1=CC2=C(NC(=O)N2)C=C1	227,24	C8H9N3O3S
Z1483950793	NCC1CN2CCN1CC2	141,22	C7H15N3

Z1266854884	NCC1(O)CCCC2=C1C=CC=C2	177,25	C11H15NO
Z2471986955	CC1OC2(CC1=C)CCNCC2	167,25	C10H17NO
Z1549154938	CC#CCNC1=NC2=C(C=C1)N=CC=N2	198,23	C11H10N4
Z1520646254	OC(=O)C1CCC2=C(C1)C=NN2	166,18	C8H10N2O2
Z1561856325	C=CCOC1=CC2=C(NC=C2)C=C1	173,22	C11H11NO
Z1259339741	CC(C)CC1=NNC(N)=C1	139,20	C7H13N3
Z1460224226	FC1=C(Cl)C=CC(NC2CCNC2=O)=C1	228,65	C10H10ClFN2O
Z1480642716	N#CC1=CC2=C(OCC2)C=C1	145,16	C9H7NO
Z1465461251	CNS(=O)(=O)C1=C(C)C(N)=CC=C1	200,26	C8H12N2O2S
Z1454649804	CC1=C(C=CC=C1N)C1=NN=CO1	175,19	C9H9N3O
Z1436473077	CN(C)C1=NN=C(O1)C1=CC=C(N)C=C1	204,23	C10H12N4O
Z99598966	COC1=CC(OC)=C(C=C1)C1CCCN1	207,27	C12H17NO2
Z1168422782	C1CC1C1=CSC(=N1)C1=NC=CN=C1	203,26	C10H9N3S
Z1267773594	CCOC1=C(C)C=C(CN)C=C1	165,24	C10H15NO
Z90515731	CC1=C(CNC2CCCC2)C(C)=NO1	194,28	C11H18N2O
Z1245735294	CN(C)C1=C(CO)C=CC=C1	151,21	C9H13NO
Z2199779515	CC1=NSC(=N1)N1CCC(CC1)C1CC1	223,34	C11H17N3S
Z1333173543	CC1=NC(N[C@H]2C[C@@H](CO)C=C2)=NC=C1	205,26	C11H15N3O
Z1957730368	CC1CN(CCCO1)C1=C(C)C=NC=C1	206,29	C12H18N2O
Z1266823301	CC(N)C(C)N1C=NC=N1	140,19	C6H12N4
Z32968167	CC1CCCCN1C(=O)C1=CC=CO1	193,25	C11H15NO2
Z1268152349		205,31	C12H19N3
Z1262396021	CC1=CSC(=N1)C1(N)CCC1	168,26	C8H12N2S
Z1272494722	CC1=NSC(NC2CCCC2CO)=N1	213,30	C9H15N3OS
Z1925930296	NC1=CC2=C(CC(O)C2)C=C1	149,19	C9H11NO
Z133716556	CS(=O)(=O)NC1CCOC2=C1C=CC=C2	227,28	C10H13NO3S
Z1699554564	CN1N=C(C)N=C1NCC1=CC(Br)=CS1	287,18	C9H11BrN4S
Z1729206226	CC(C)C1=CC=C(CN2C=CN=N2)C=C1	201,27	C12H15N3
Z85878070	OC(=O)C1=CC(NC(=O)C2CC2)=CC=C1	205,21	C11H11NO3
Z600428392	CC1=NOC(=N1)C1=CC(N)=C(C)C=C1	189,22	C10H11N3O
Z317476482	CC(C)NC(=O)C1=CC(=CC=C1)C(N)=O	206,25	C11H14N2O2
Z373775660	CN1C=C(NC(=O)C2=NC=CN=C2)C=N1	203,21	C9H9N5O
Z373775574	CN1C=C(NC(=O)C2=CC=CS2)C=N1	207,25	C9H9N3OS
Z219186376	O=C1CN(CCN1)C1=NC=C(C=C1)C#N	202,22	C10H10N4O
Z136725178	CC1=NOC(=C1)C(=O)NC1=NC=CC=C1	203,20	C10H9N3O2
Z167942650	COCCN1N=C(C)C(C)=C(C#N)C1=O	207,23	C10H13N3O2
Z1259341087	N[C@H]1[C@@H](O)CC2=C1C=CC=C2	149,19	C9H11NO
Z1259273299	OC(=O)CC1CCCC2=C1C=CC=C2	190,24	C12H14O2
Z1238477764	NC1=CC2=C(OC(=N2)C2CC2)C=C1	174,20	C10H10N2O
Z1030080890	CC1=CC(=CC=C1)C1(CCCC1)C(N)=O	203,29	C13H17NO
Z1127124401	O[C@H]1CCN(C1)C1=NC2=C(O1)C=CC=C2	204,23	C11H12N2O2
Z728939692	CN(C)C1=NC2=C(C=CC=C2)N=C1	173,22	C10H11N3
Z1171978873	CC1=CC2=C(NN=C2)C(=C1)C(O)=O	176,18	C9H8N2O2
Z1162470564	CCC1CCC2=C(C1)C=C(C#N)C(=O)N2	202,26	C12H14N2O
Z68248958	CNC(=O)C1=CC2=C(CCCC2)S1	195,28	C10H13NOS
Z955526728	CNC(=O)C1CC1C1=CC(Br)=CC=C1	254,13	C11H12BrNO
Z54747399	CN(C)C(=O)CN1CCC(CC1)C(N)=O	213,28	C10H19N3O2
Z425387838	CC1=CC=C(C=C1)C1=CSC(CN)=N1	204,29	C11H12N2S
Z234895123	CNC(=O)C1CCCCN1	142,20	C7H14N2O
Z2678251704	CC(CC1=C(C)C=CC=C1)C(O)=O	178,23	C11H14O2
Z2678251689	CC(C)COC1=CN=CC=C1	151,21	C9H13NO
Z1373430305	FC1=C(NCC2CCOCC2)N=CC=C1	210,25	C11H15FN2O
Z1601231985	FC1=C(F)C=C(NCC2=NNC=C2)C=C1	209,20	C10H9F2N3
Z1373427763	CC1=NOC(CNC2=C(F)C=CC=N2)=C1	207,21	C10H10FN3O
Z1973746834	CC1=CCCN(C1)C1=NC(C)=CC(C)=N1	203,29	C12H17N3
Z1573518667	CC1=C(NCC2=CSN=N2)C=CC=C1F	223,27	C10H10FN3S
Z1613960888	CC(=O)NCC1=CC=NO1	140,14	C6H8N2O2
Z2216303543	OC(=O)C1CCC(C1)C1=CC=CC=C1	190,24	C12H14O2
Z2197159967	NC1=C(OCC2CCOC2)C=CC(Br)=C1	272,14	C11H14BrNO2
Z1407004013	CC(N)C1=CC(F)=C(C)C=C1	153,20	C9H12FN
Z2584255713	COC1=C(C=CC=C1)C1=NOC(Cl)=C1	209,63	C10H8ClNO2
Z2442592378	CCC1=NOC(CC2CCNCC2)=N1	195,27	C10H17N3O
Z1889975637	CC1(C)CCC(O1)C(O)=O	144,17	C7H12O3
Z2216523547		209,22	C11H12FNO2
Z2216303101	CN1C=NN(CCC(O)=O)C1=O	171,16	C6H9N3O3
Z2216302968	NC1=CC=C(C=C1)C1CCOC1	163,22	C10H13NO
Z2213893377	CN(C)C1=CC=C(C=C1)C1(CC1)C(O)=O	205,26	C12H15NO2

Z31725206	OCCNC(=O)C1=CC=C(Cl)C=C1	199,63	C9H10ClNO2
Z94832620	NC(=O)CNC1=C2C=CSC2=NC=N1	208,24	C8H8N4OS
Z1891776008	CN1CC(CN)CC2=C1C=CC=C2	176,26	C11H16N2
Z2182155697		176,22	C11H12O2
Z2177031159	NC1=NC=C2CCCCC2=C1	148,21	C9H12N2
Z359419600	CN1N=C(C)C2=C1N=C(C)C(=C2)C(O)=O	205,22	C10H11N3O2
Z281802052	CN(C)C1=NC=C(C=C1)C#N	147,18	C8H9N3
Z327224228	CN(C)C1=C(C=C2CCCCC2=N1)C#N	201,27	C12H15N3
Z30281273	CCNC(=O)C1=CC(Br)=CN=C1	229,08	C8H9BrN2O
Z85886818	CC1CC1C(=O)NCC(O)=O	157,17	C7H11NO3
Z57728694	CC1=CC2=C(OC(=O)N2CC(O)=O)C=C1	207,19	C10H9NO4
Z188924172	OC(=O)CCC1=CC2=C(OCCO2)C=C1	208,21	C11H12O4
Z256708392	COC1=C(OC)C(=CC=C1)C(O)=O	182,18	C9H10O4
Z1203890368	OC1CCCCC1NCC1=CC=C(Cl)S1	245,77	C11H16ClNOS
Z416879296	OCC1CCN(CC2=CC=C(Cl)S2)CC1	245,77	C11H16ClNOS
Z1555979444	CC(C)=C(F)C1=NC(=NO1)C1CCOC1	212,22	C10H13FN2O2
Z1657807341	CN1C(C)=NN=C1NC(=O)C1CCCC1	208,27	C10H16N4O
Z1354416046	CCC1=NOC(=N1)C1=CC(N)=C(C)C=C1	203,25	C11H13N3O
Z1347206048	CNS(=O)(=O)C1=C(OC)C=C(C)C(C)=C1	229,29	C10H15NO3S
Z1347206039	CNS(=O)(=O)C1=CC(C)=C(Cl)C=C1	219,68	C8H10ClNO2S
Z905065926	CC(C)N1C2CCC1CC(C2)C(O)=O	197,28	C11H19NO2
Z1335657824	CC(C)C(O)(C(O)=O)C1=CC=CC=C1	194,23	C11H14O3
Z1335657800	COC1=C(CO)C=C(Cl)C=C1	172,61	C8H9ClO2
Z1333043358	COC1=C(N)N=C(C)C=C1	138,17	C7H10N2O
Z1324054916	CN1N=CC2=C1N=CC(=C2)C(O)=O	177,16	C8H7N3O2
Z729603782	OC(=O)C1CN(C(=O)C1)C1=NC=CS1	212,22	C8H8N2O3S
Z857666962	OC(=O)C1=C(Br)C(=NN1)C1CC1	231,05	C7H7BrN2O2
Z1614607167	OC(=O)C1(CC1)C(=O)N1CCCC1	183,21	C9H13NO3
Z1815156923	COC1=C(C=C(Br)C(C)=N1)C(O)=O	246,06	C8H8BrNO3
Z1815155511	COC1=C(C(O)=O)C(N)=C(Br)C=C1	246,06	C8H8BrNO3
Z1815155451	CN1N=CC=C1C(O)C1=CC=CC=C1	188,23	C11H12N2O
Z1815155461	OC(=O)CC1=CC=C(Cl)S1	176,61	C6H5ClO2S
Z1147473989	NC(=O)C1NCCC2=C1C=CC=C2N	191,23	C10H13N3O
Z1741970419	COC1=CN=C(C=C1)C(O)=O	153,14	C7H7NO3
Z1381299162	CN(C(N)=O)C1=CN=CC=C1	151,17	C7H9N3O
Z1067997558	BrC1=CN=C(C=C1)C(=O)N1CCCC1	255,12	C10H11BrN2O
Z90667799	OC(=O)C1=NC=CC(=C1)N1CCCC1	192,22	C10H12N2O2
Z1741962447	CC1=CSC2=C1N=CNC2=O	166,20	C7H6N2OS
Z1787158566	O=C1NCCN1CCN1CCNCC1	198,27	C9H18N4O
Z127111070	COC1=NC(NC2CCCC2)=NC=C1	193,25	C10H15N3O
Z1696946301	CC1OCCC1C(=O)NC1=NC=CN=C1	207,23	C10H13N3O2
Z1778350604	CC1=C(Br)C=C(CC(O)=O)C=C1	229,07	C9H9BrO2
Z409308414	CC1CC1CNC(=O)C1=C(N)N=CC=N1	206,25	C10H14N4O
Z451350338	CN1C=C(C=N1)C1=NC(=CS1)C(O)=O	209,22	C8H7N3O2S
Z332625816	BrC1=CC(=CS1)C(=O)N1CCOCC1	276,15	C9H10BrNO2S
Z57985394	CC1=C(CCC(O)=O)C(C)=NN1	168,20	C8H12N2O2
Z274538288	COC1=CC=C(C=C1)C1=C(N)NN=C1C	203,25	C11H13N3O
Z425389704	CC(C)C1=CC(=NN1)C(O)=O	154,17	C7H10N2O2
Z398513274	CC(C)OC1=NC=C(C=C1)C(O)=O	181,19	C9H11NO3
Z19739447	CNC(=O)COC1=CC=C(C=C1)C#N	190,20	C10H10N2O2
Z405825414	CCC(=O)N1CCCC(C1)NS(C)(=O)=O	234,31	C9H18N2O3S
Z394174378	CC1=CC(=NO1)C(=O)NCC1CCOC1	210,23	C10H14N2O3
Z992716900	CC1=NN2C=C(C(O)=O)C(=O)NC2=C1	193,16	C8H7N3O3
Z226499634	NC(=O)CCN1C=NC2=C(SC=C2)C1=O	223,25	C9H9N3O2S
Z396112684	CN1C=C(NC(=O)C2=CC=C(Br)S2)C=N1	286,15	C9H8BrN3OS
Z216237334	CNC(=O)CN1CCC(CC1)C(=O)NC	213,28	C10H19N3O2
Z1868316890	OC(=O)C1=NN2CCCOC2=C1	168,15	C7H8N2O3
Z2106601577	CC(C)C(N1N=C(C)C=C1C)C(O)=O	196,25	C10H16N2O2
Z2418194250	CN1N=C(N=C1CC(O)=O)C1CC1	181,20	C8H11N3O2
Z444349634	CC(N)C1=CC2=C(NC(=O)N2)C=C1	177,21	C9H11N3O
Z2235390200		190,25	C11H14N2O
Z1070938482	C(NC1CCOC2=C1C=CC=C2)C1CC1	203,29	C13H17NO
Z2053696021	C[C@@H]1CN(C[C@H]1C(N)=O)C1=NC=CC=N1	206,25	C10H14N4O
Z1184890099	NC1=NC=C(C=C1)N1CCCCC1	177,25	C10H15N3
Z159528224	CNC(C)C1=C(F)C=CC=C1	153,20	C9H12FN
Z2327131645	OC(=O)C1(O)CCC2=C(C1)C=CC=C2	192,21	C11H12O3
Z1245537943	CCC(=O)NC1=CC=C(C=C1)C(C)N	192,26	C11H16N2O

Z31255646	FC1=CC=C(CNC2=NC=CC=N2)C=C1	203,22	C11H10FN3
Z1262252950	NCC1=CC2=C(CCCCC2)S1	181,30	C10H15NS
Z1407654394	OCC1CC11CCOCC1	142,20	C8H14O2
Z1266823139	NC(=O)C1NCCC2=C1C=CC=C2	176,22	C10H12N2O
Z56791573	CC(C)CC1=NN=C(N)S1	157,24	C6H11N3S
Z2211231470	O=C1NCCN1CC1=CN=CC=C1	177,21	C9H11N3O
Z1713595338	CS(=O)(=O)N1CCC[C@@H]1CN	178,25	C6H14N2O2S
Z1893225335	NCC1=CC=C(C=C1)N1CCNC1=O	191,23	C10H13N3O
Z1269638430	CC(=O)NCC1CNCCO1	158,20	C7H14N2O2
Z32016429	CNC(=O)CNC(=O)C1=CC=CC=C1	192,22	C10H12N2O2
Z104829962	NC(=O)CNC1=C(F)C=C(Cl)C=C1	202,61	C8H8ClFN2O
Z26743795	COC1=C(Cl)C=C(NC(=O)C(C)C)C=C1	227,69	C11H14ClNO2
Z221429956	OC(=O)C1=CC=C(CN2C=NC=N2)C=C1	203,20	C10H9N3O2
Z57070418	CN1C(=O)NC2=C1C=CC=C2	148,17	C8H8N2O
Z212063048	NC1=CC2=C(C=C1)N=C(S2)N1CCCC1	219,31	C11H13N3S
Z217102760	COC1=NC=C(C=C1)C#N	134,14	C7H6N2O
Z30272262	CNC(=O)C1=CC=C(Br)C=C1	214,06	C8H8BrNO
Z32015103	CN(C)C(=O)C1=CC(NC(C)=O)=CC=C1	206,25	C11H14N2O2
Z740994328	O=C(NCC1=CC=CC=C1)C1=CSN=N1	219,26	C10H9N3OS
Z1863617864	NC1CCN(CC1)C1=NN=CC=C1	178,24	C9H14N4
Z111634612	CC(C)C1=CC(=O)N2N=C(C)N=C2N1	192,22	C9H12N4O
Z1860974908	CN1C=CC(=O)C(=C1)C(N)=O	152,15	C7H8N2O2
Z1849011119	COC1=C(N=CC(C)=C1)C(O)=O	167,16	C8H9NO3
Z1889710290	CC1=CSC(CCC(O)=O)=N1	171,21	C7H9NO2S
Z1713446741	CN1C=NN=C1NC(=O)C1=CSC=C1C	222,27	C9H10N4OS
Z1868431117	CC1(CCCOC1)C(O)=O	144,17	C7H12O3
Z1868430495	CC(C)(C(O)=O)C1=CNC=N1	154,17	C7H10N2O2
Z1222283865	COC1=C(C)C=CC(=C1)C1(CC1)C(O)=O	206,24	C12H14O3
Z1124405402	O=C(NCC1CC1)N1CCS(=O)(=O)CC1	232,30	C9H16N2O3S
Z997948620	NC(=O)CCC1=CC(Cl)=C(Cl)C=C1	218,08	C9H9Cl2NO
Z1229682715	CC1(COC2=C(C=CC=C2)C#N)COC1	203,24	C12H13NO2
Z238708260	COC1=C(C=C(F)C=C1)C(N)=O	169,16	C8H8FNO2
Z56851231	CC1=NC2=C(C=CC=C2)C(N)=C1	158,20	C10H10N2
Z216238752	CNC(=O)C1CCN(CC(C)=C)CC1	196,29	C11H20N2O
Z1184882872	CN1CCC(O)(CC1)C1=CC=CC=C1	191,27	C12H17NO
Z1722750874	CN(CC1=NC=CS1)C1=NC(C)=CC=C1	219,31	C11H13N3S
Z1245633080	NC1=C(CN2CCCC2)C=CC=C1	176,26	C11H16N2
Z154053600	CC(=C)CN1N=C(C)C(C)=C(C#N)C1=O	203,25	C11H13N3O
Z1267882017	NCC1CCCN1CC1=CC=CC=C1	190,29	C12H18N2
Z1495385325	C1CC(CN1)C1CCOC1	141,21	C8H15NO
Z30718674	CN(CC1=CC=CS1)C(=O)C1CCC1	209,31	C11H15NOS
Z374427992	CN(C)C1=NC=CC(CNC(C)=O)=C1	193,25	C10H15N3O
Z87002128	COC1=CC2=C(OC(C)=C2C(O)=O)C=C1	206,20	C11H10O4
Z1439422127	CC1OCCN(C1C)C1=NC(C)=NS1	213,30	C9H15N3OS
Z1331054519	CC(C(O)=O)C1=CC=C(C)S1	170,23	C8H10O2S
Z373768900	CCN1C=C(NC(=O)C2CCC2)C=N1	193,25	C10H15N3O
Z1266823336	CN(CCN)C1CCCC1	142,25	C8H18N2
Z1188324959	CCN(C)C(=O)NCC1=CC(Cl)=CC=C1	226,70	C11H15ClN2O
Z1250132786	NC1CCN(CC1)C1=CC(F)=CC=C1	194,25	C11H15FN2
Z224449904	ClC1=CC=C(O1)C(=O)N1CCCCCC1	227,69	C11H14ClNO2
Z2509351076	CNS(=O)(=O)C1=C(N)C=CC=N1	187,22	C6H9N3O2S
Z2492800177	COC1=CC2=C(CCCC2C(O)=O)C=C1	206,24	C12H14O3
Z2492774599	COC1=CN2N=CC(C(O)=O)=C2C=C1	192,17	C9H8N2O3
Z963502702	OC(=O)C1=C2OCCOC2=CS1	186,18	C7H6O4S
Z2471262567	COC1=C2C(O)=NC=NC2=CC=C1	176,18	C9H8N2O2
Z2510258230	OC(=O)C1=CN(N=N1)C1CC1	153,14	C6H7N3O2
Z2510258122	OC(=O)CN1NC2=C(C=CC=C2)C1=O	192,17	C9H8N2O3
Z1982493921	NCCC1=NC(=CC=C1)C(O)=O	166,18	C8H10N2O2
Z353243890	CC(CO)NC(=O)C1=C(Br)C=C(F)C=C1	276,11	C10H11BrFNO2
Z1966567481	CN(C)C(=O)C1=CC(N)=CC(C)=C1	178,24	C10H14N2O
Z1948933405	NC1=NC=CC(=C1)S(=O)(=O)N1CCCC1	227,28	C9H13N3O2S
Z1946684528	NC1(CCC2=C(C1)C=CC=C2)C(O)=O	191,23	C11H13NO2
Z1729209128	COC1=C(CN2C=CN=N2)C=C(Cl)C=C1	223,66	C10H10ClN3O
Z1983079741	COC1=CC2=C(OC=C2C(O)=O)C=C1	192,17	C10H8O4
Z68590139	NC(=O)C1=C(C=CC=C1)C(F)(F)F	189,14	C8H6F3NO
Z827056754	CN(C(C)=O)C1=NC(CS(C)(=O)=O)=CS1	248,32	C8H12N2O3S2
Z992717056	CC1=CSC(=N1)C1=CC=C(C=C1)C(O)=O	219,26	C11H9NO2S

Z744930860	CCC(C)(C)NC(=O)C1=CC(C)=NN1	195,27	C10H17N3O
Z56760747	COCC1=C(N)N=C(C)N=C1	153,19	C7H11N3O
Z2408594799	CNC1=NN=C(O1)C1=CC=CC=C1	175,19	C9H9N3O
Z2692095125	CN1CC(=O)NC2=C1C=C(Cl)C=C2	196,63	C9H9ClN2O
Z2692093683	NC1=CC(=CC=C1)C1CCOCC1	177,25	C11H15NO
Z274538286	CC1=NNC(N)=C1C1=CC=C(F)C=C1	191,21	C10H10FN3
Z1820178775	COC1=C(N)C2=C(NC=C2)C=C1	162,19	C9H10N2O
Z1716711716	CN(C)C1=C(F)C=C(C=C1)C(O)=O	183,18	C9H10FNO2
Z1816507484	OC(=O)C1=COC(=N1)C1CCC1	167,16	C8H9NO3
Z1695756662	CN1C=NN=C1NC(=O)C1=COC(Br)=C1	271,07	C8H7BrN4O2
Z1836338827	NC1=NN=C(NCC2=CC=CC=C2)O1	190,21	C9H10N4O
Z1653345844	CCC(=O)N(C)C1=C(Br)C=CC=N1	243,10	C9H11BrN2O
Z118458694	COC1=C(C=CC(Cl)=C1)C(O)=O	186,59	C8H7ClO3
Z1258992647	COC1=CC(CO)=NC=C1	139,15	C7H9NO2
Z56820874	CC1=C(C)C=C(OCC(O)=O)C=C1	180,20	C10H12O3
Z1258943242	CN1C(=O)NC2=C1C=CN=C2	149,15	C7H7N3O
Z224746000	CNC(=O)C1=CC=C(C=C1)N1C=NC=N1	202,22	C10H10N4O
Z1258578343	NCC1(CC(O)=O)CCCCC1	171,24	C9H17NO2
Z1255523357	CC1=CN=C(C=C1)N1CCC(N)CC1	191,28	C11H17N3
Z1250208655	CC1CCCC(N)(C1)C(O)=O	157,21	C8H15NO2
Z1238835893	NCC(O)C1=CC2=C(OCCO2)C(Cl)=C1	229,66	C10H12ClNO3
Z927405036	NC1=C(C=C(Cl)C=C1)C(=O)NC1CC1	210,66	C10H11ClN2O
Z1259340984	NC(C(O)=O)C1=CC(F)=CC=C1	169,16	C8H8FNO2
Z1259339927	CN(C)C1=C(C=C(Br)C=N1)C(O)=O	245,08	C8H9BrN2O2
Z1259335906	CC(C)C1=C(N=CO1)C(O)=O	155,15	C7H9NO3
Z1259335908	CC1=NC(=NN1C1=CC=CC=C1)C(O)=O	203,20	C10H9N3O2
Z360882520	OC(=O)CCN1N=C2C=CC=CN2C1=O	207,19	C9H9N3O3
Z1259335818	OC(=O)CC1=NOC(=C1)C1=CC=CC=C1	203,20	C11H9NO3
Z228584328	OC(=O)CCC1=NC2=C(S1)C=CC(Cl)=C2	241,69	C10H8ClNO2S
Z1259273127	OC(=O)C1CNC2=C1C=CC=C2	163,18	C9H9NO2
Z1259162083	Cl.CC(N)C1=C(C=CC=C1)C(F)(F)F	225,64	C9H11ClF3N
Z57915930	COC1=C(C=C(Br)C=C1)C(O)=O	231,05	C8H7BrO3
Z1741977234	COC1=NC2=C(NC=C2)C=C1	148,17	C8H8N2O
Z2689031548	COC1=CC(=CC=C1)C(O)CO	168,19	C9H12O3
Z1974213299	CC1=C(NC=C1)C(=O)N1CCC=C(F)C1	208,24	C11H13FN2O
Z1267773497	CN1N=C(C)C=C1N1CCC(N)C1=O	194,24	C9H14N4O
Z1263529814	CC(CN)OC1=CC(C)=CC=C1	165,24	C10H15NO
Z2687203880	COCC1CCNC(=O)C1	143,19	C7H13NO2
Z1724883809	CNS(=O)(=O)C1=C(F)C=CC=C1C	203,23	C8H10FNO2S
Z1741958230	OC(=O)CC1=CC(Cl)=C(F)C=C1	188,58	C8H6ClFO2
Z2684296842	OC(=O)C1=CC=C(CN2C=CN=N2)C=C1	203,20	C10H9N3O2
Z1530133038	CS(=O)(=O)NCC1=NC=CN1	175,21	C5H9N3O2S
Z2680629434	CC1=CC(F)=C(C(O)=O)C(N)=C1	169,16	C8H8FNO2
Z1171979211	CC(C)NC1=NC=C(C=C1)C(O)=O	180,21	C9H12N2O2
Z1171978798	CC1OC2=C(NC1=O)C=CC=C2C(O)=O	207,19	C10H9NO4
Z246686976	COC1=C(C=CC(Cl)=C1)S(=O)(=O)N(C)C	249,71	C9H12ClNO3S
Z1170039625	OC(=O)CN1C(=O)CCC2=C1C=CC=C2	205,21	C11H11NO3
Z1769859572	OC(=O)C1=C(Br)C=NC(=N1)C1CC1	243,06	C8H7BrN2O2
Z52584368	CNC(=O)CNC1=C2C=CSC2=NC=N1	222,27	C9H10N4OS
Z1762658221	NC(CC1=CC=CO1)C(O)=O	155,15	C7H9NO3
Z1753012806	CC1=NN2CCC(=O)N(CC(O)=O)C2=C1	209,21	C9H11N3O3
Z1751961091	Cl.Cl.CC(N)C1=C(C)N(C)N=C1	212,12	C7H15Cl2N3
Z1742055044	CNC1(CO)CCC(C)C1	143,23	C8H17NO
Z1742055041	OC(C1=CC=CO1)C1=NC=CN1	164,16	C8H8N2O2
Z929061422	CC(C)(NC(=O)C1=C(Br)C=CS1)C(O)=O	292,15	C9H10BrNO3S
Z394693484	CN(CC(O)=O)C1=NC=C(Cl)C=C1	200,62	C8H9ClN2O2
Z1603606795	CC1=C(OC=C1)C(=O)NC1(CC1)C(O)=O	209,20	C10H11NO4
Z1718361633	COC1=C(Cl)C(OC)=C(C=C1)C(O)=O	216,62	C9H9ClO4
Z1270484159	FC(F)(F)CN1CCCNCC1	182,19	C7H13F3N2
Z1398473882	CC(N)COC1CCOC1	145,20	C7H15NO2
Z1578665941	CC1=NSC(=N1)N1CCCNCC1	198,29	C8H14N4S
Z234898257	CNCC1=NN=C2C=CC=CN12	162,20	C8H10N4
Z1744662180	CC(O)CN1CCNCC1	144,22	C7H16N2O
Z2146566307	NCC1COC2=C1C=CC=C2	149,19	C9H11NO
Z2146513917	CC1=CC=C(O1)C1=CNC(=O)C=N1	176,18	C9H8N2O2
Z2111526430		158,20	C8H14O3
Z1911007933	CC1N(CCNC1=O)C1=C(Br)C=CC=N1	270,13	C10H12BrN3O

Z2092369612	OCC1=C(CC2CCNCC2)C=CC=C1	205,30	C13H19NO
Z2081962638	CNC(=O)C1=C2CNCCN2N=C1	180,21	C8H12N4O
Z1918536387	CN1C=C(OC2=NC=C(Cl)C=N2)C=N1	210,62	C8H7ClN4O
Z1839021932	CNC(=O)COC1=CC(C)=C(Br)C(C)=C1	272,14	C11H14BrNO2
Z2160914783	OC(=O)C1(CNC(=O)C1)C1=CC=CC=C1	205,21	C11H11NO3
Z1674937530	CCN1C=C(CCO)C=N1	140,19	C7H12N2O
Z1768415350	OC(=O)C1CC2(C1)CCCCC2	168,24	C10H16O2
Z1498346202	CN(CC1(O)CCCC1)C1=NC=CC=N1	207,28	C11H17N3O
Z414834692	NC1C2CCCC1CN(C2)C1CC1	180,30	C11H20N2
Z360055638	CCOC1=CC=C(C=C1)[C@@H](C)O	166,22	C10H14O2
Z1431002654	NCC1(CCO)CCOC1	145,20	C7H15NO2
Z1497165919	CC1CCC(N)C2=C1C=CC=C2	161,25	C11H15N
Z1324054926	NCCC1=NC(=CS1)C1CC1	168,26	C8H12N2S
Z1324054929	C1CNCC2(C1)CCCOC2	155,24	C9H17NO
Z1522182920	CC1=C(Br)C=CC(=C1)C(=O)N1CC(O)C1	270,13	C11H12BrNO2
Z1744658498	CCN1CCNC(C)C1=O	142,20	C7H14N2O
Z1270207989	CN1C=C(C=N1)N1CCC(N)C1=O	180,21	C8H12N4O
Z56983120	CC1=C(C=CC=C1)C(=O)NCC(O)=O	193,20	C10H11NO3
Z422398218	CCC(C)(C)NC(=O)C1=C(C)N=CS1	212,31	C10H16N2OS
Z31384892	CC(C)NC(=O)CC1=CSC(C)=N1	198,28	C9H14N2OS
Z49527506	BrC1=CC=C(C=C1)C1=NNC(=O)CC1	253,10	C10H9BrN2O
Z237528064	CCC(N1C=NC2=C1C=CC=C2)C(O)=O	204,23	C11H12N2O2
Z57085731	CC(O)C1=NC2=C(C=CC=C2)N1C	176,22	C10H12N2O
Z18430744	NC(=O)COC1=CC(Cl)=C(F)C=C1	203,60	C8H7ClFNO2
Z18532934	CN(C)C(=O)COC1=C(Cl)C(Cl)=CC=C1	248,10	C10H11Cl2NO2
Z234796412	CC1=CC=C(C#N)C(=O)N1	134,14	C7H6N2O
Z29693812	O=C(NCC1=CC=CS1)C1=NC=CN=C1	219,26	C10H9N3OS
Z992422554	CC(CCN)C1=CC=CC=C1	149,24	C10H15N
Z108545254	CN1C=CC(NC(=O)C2=CC=C(C)O2)=N1	205,22	C10H11N3O2
Z729726726	CC1=NOC(NC(=O)C2=CC=CC=C2)=N1	203,20	C10H9N3O2
Z26794351	CCC(=O)NC1=CC2=C(NC(=O)N2)C=C1	205,22	C10H11N3O2
Z55928783	OC(=O)CNC(=O)C1=CC(Br)=CC=C1	258,07	C9H8BrNO3
Z1483951153	COC1(C)CC(O)C1(C)C	144,21	C8H16O2
Z90507426	C(NC1CCCCCC1)C1=CC=NC=C1	204,32	C13H20N2
Z1192046301	NCC1=NC(=NC=C1)N1CCCC1	178,24	C9H14N4
Z254582744	CN(C)C(=O)C1=C(F)C=CC=C1F	185,17	C9H9F2NO
Z32455036	CN(CC1=CC=CO1)C(=O)C1CCCO1	209,25	C11H15NO3
Z969563028	NC1CCCC2=C1C=CC(OC(F)F)=C2	213,23	C11H13F2NO
Z223428262	CC1CC1C1=CC=C(CN(C)C(C)=O)O1	207,27	C12H17NO2
Z915781054	CCC(C)(C)NC(=O)C1=CC=NN1C	195,27	C10H17N3O
Z204776284	CNC(=O)CN(C)C1CCCCCC1	198,31	C11H22N2O
Z265547286	CC1CCN(CC2=NC(C)=NO2)CC1	195,27	C10H17N3O
Z422522084	CN(C)C(=O)C1=C(C)N=CS1	170,23	C7H10N2OS
Z100642434	CN(C)C(=O)CCN1C(C)=CSC1=O	214,28	C9H14N2O2S
Z271004888	CNC1=NC(OC)=CC=C1	138,17	C7H10N2O
Z2235814825	CC1(CO)CCC(=O)NC1	143,19	C7H13NO2
Z2327226192	OC(=O)C1CN(C1)C1=C(C=CC=C1)C#N	202,21	C11H10N2O2
Z2327040060	COC1=CC2=C(C(=O)NC(C)=N2)C(Br)=C1	269,10	C10H9BrN2O2
Z111423554	COC1=CC(N)=C(C=C1)N1C=CC=C1	188,23	C11H12N2O
Z274764492	NC1=NN=C(COC2=CC=C(Cl)C=C2)S1	241,69	C9H8ClN3OS
Z2301438511	CC1=C(CCC(O)=O)SC=N1	171,21	C7H9NO2S
Z2284385174	CC1=NC(C)=C(Cl)C(N)=C1Cl	191,06	C7H8Cl2N2
Z736586356	CC(=O)NC1CCCN(C1)S(C)(=O)=O	220,29	C8H16N2O3S
Z1267881684	CC(C)N1C=C2C=C(N)C=CC2=N1	175,24	C10H13N3
Z1267773786	CCNC1=NN=C(C)C=C1	137,19	C7H11N3
Z1267773695	NC1=C(C=C2CCCCC2=N1)C#N	173,22	C10H11N3
Z1266933933	CCOC(C)C1=NC=CN1	140,19	C7H12N2O
Z941495390	BrC1=C(CNC(=O)C2CC2)SC=C1	260,15	C9H10BrNOS
Z1263714107	CC(C)NC1=CC(C)=C(C=C1)C(N)=O	192,26	C11H16N2O
Z1263714081	CNCC1=CC=C(O1)C(=O)NC	168,20	C8H12N2O2
Z1263602450	CNS(=O)(=O)C1=C(N)C=C(OC)C=C1	216,26	C8H12N2O3S
Z57796090	CC1=NN(C(=O)C1)C1=NC(C)=CC(C)=N1	204,23	C10H12N4O
Z1741815243	NCCN1CCC2(CCCCC2)CC1	196,34	C12H24N2
Z1675323023	C1CC1C1COCCN1C1=NC=CC=N1	205,26	C11H15N3O
Z2234185661	O=C1COCC(N1)C1=CC=CC=C1	177,20	C10H11NO2
Z416879132	CC1=CC(CN2CCC(CO)CC2)=NO1	210,28	C11H18N2O2
Z2052057458	CCS(=O)(=O)N1CCO[C@H](C)[C@H]1C(N)=O	236,29	C8H16N2O4S

Z354991212	CC1CN(CC2=NOC(C)=C2)C(C)CO1	210,28	C11H18N2O2
Z228570974	NCC(N1CCCC1)C1=CC(Cl)=CC=C1	224,73	C12H17ClN2
Z216450634	CN(C1CCCCCC1)S(C)(=O)=O	205,32	C9H19NO2S
Z31152880	CN(CC1=CC=C(C)O1)C1=NC=CC=N1	203,25	C11H13N3O
Z85895292	CN(CC(O)=O)C(=O)C1=CC=C(Br)O1	262,06	C8H8BrNO4
Z1373429822	OC[C@H]1CCCN1C1=C(F)C=CC=N1	196,23	C10H13FN2O
Z2272595387	CC1=CC(=CO1)C(=O)NC(C)(C)C(O)=O	211,22	C10H13NO4
Z2017991856	CC(=O)NC1=CN=C(OC(F)F)C=C1	202,16	C8H8F2N2O2
Z2273418869	FC1=CCCN(CC2=CN=NS2)C1	199,25	C8H10FN3S
Z1460223235	FC1=C(NC2CCNC2=O)C=C(Cl)C=C1	228,65	C10H10ClFN2O
Z1796224493	CN1CCCC1C(=O)N1CCC=C(F)C1	212,27	C11H17FN2O
Z1766614316	CC1=CC(=CO1)C(=O)N1CCC=C(F)C1	209,22	C11H12FNO2
Z2274076372	CC(C)C1CCN1C1=NC=C(F)C=N1	195,24	C10H14FN3
Z1481134589	FC1=CN=C(N=C1)N1CCC(=C)CC1	193,23	C10H12FN3
Z1267885776	COC1=C(N)C(C)=NN1C	141,17	C6H11N3O
Z1262396035	CCOC1=C(CN)C=CC=N1	152,20	C8H12N2O
Z2234631183	OC(=O)C1CCN(C1)C1=CC=CC=C1	191,23	C11H13NO2
Z2327224883	O[C@@H]1CC[C@H]1OCC1=CC=CC=C1	178,23	C11H14O2
Z2235790909	OCC1CN(CCO1)C1=CC=CC=C1	193,25	C11H15NO2
Z2327226290	NC1CCN(C1)C1=CC=C(F)C=C1	180,23	C10H13FN2
Z2327226304	CNC(C)(C)CN1CCOCC1	172,27	C9H20N2O
Z85919065	CC1=C(CC(O)=O)C(C)=NO1	155,15	C7H9NO3
Z57052849	OC(=O)CCN1NC(=O)C=CC1=O	184,15	C7H8N2O4
Z55928802	FC(F)OC1=CC=C(C=C1)C#N	169,13	C8H5F2NO
Z57473788	NC1=NN(CC1)C1=CC=CC=C1	161,21	C9H11N3
Z89283361	NC(C1=CC=CS1)C1=CC=CC=C1	189,28	C11H11NS
Z57042068	OC(=O)COC1=C(Cl)C=CC=C1	186,59	C8H7ClO3
Z56347545	OC(=O)CN1C(=O)C=NC2=C1C=CC=C2	204,19	C10H8N2O3
Z56347236	NC1=C(C=CC=C1)C(=O)NC1CCCC1	204,27	C12H16N2O
Z56347132	CC(C1CC1)N1N=CC=C1N	151,21	C8H13N3
Z31205964	CN(CCO)C1=C2C=CSC2=NC=N1	209,27	C9H11N3OS
Z57001599	CC1=CC(=O)NC(O)=C1C#N	150,14	C7H6N2O2
Z56973555	CCC1=C(C(O)=O)C(C)=NO1	155,15	C7H9NO3
Z29671587	CC1=CC=C(O1)C(=O)NC1=NN=C(C)S1	223,25	C9H9N3O2S
Z53038500	CCNS(=O)(=O)C1=CC2=C(OCC2)C=C1	227,28	C10H13NO3S
Z818727094	CC(C)CC1=NOC(N)=C1	140,19	C7H12N2O
Z993967022	CC1=NOC(Cl)=C1CC(O)=O	175,57	C6H6ClNO3
Z87660867	CNC(=O)CN(C)C(=O)C1=CC=C(Br)S1	291,16	C9H11BrN2O2S
Z364575442	CC1COCCN1C(=O)C1=CC(C)=NO1	210,23	C10H14N2O3
Z31407955	CC1=C(SC=C1)C(=O)NCC1=CC=CO1	221,27	C11H11NO2S
Z432742414	BrC1=CC(CN2CCNC(=O)C2)=CS1	275,16	C9H11BrN2OS
Z98655413	C[C@H](NC(=O)C1CCCCC1)C(O)=O	199,25	C10H17NO3
Z381785592	CCC1=C(C=NN1)C(O)=O	140,14	C6H8N2O2
Z55992974	COC1=C(OC)C(Cl)=CC(=C1)C(O)=O	216,62	C9H9ClO4
Z235354629	CC1=C(N=C(S1)C1=CC=CO1)C(O)=O	209,22	C9H7NO3S
Z228588958	CC1=CC(=CC=C1)C1(CCC1)C(O)=O	190,24	C12H14O2
Z32014551	CN(C)C(=O)C1=CC=C(C=C1)C#N	174,20	C10H10N2O
Z30612234	CC(C)C(=O)NC1=CC=C(C=C1)C#N	188,23	C11H12N2O
Z372854136	CC1=CC=C(C=C1)C1=NOC(=C1)C(O)=O	203,20	C11H9NO3
Z367452140	NC1=NN=C(O1)C1CCCCC1	167,21	C8H13N3O
Z2467523306	CNC(=O)C1=NN=C(N)C=C1	152,16	C6H8N4O
Z2467454517	CC(C)C1CCCC2=C1NC(C)=CC2=O	205,30	C13H19NO
Z1741975083	OCCC1=NC2=C(C=CC=C2)C=C1	173,22	C11H11NO
Z1265689880	CC1=C(C=C(Br)C=C1)C(N)=O	214,06	C8H8BrNO
Z2418194164	CCN1C(=O)NN=C1CC	141,17	C6H11N3O
Z2418193660	OC(=O)C1=CN(CC=C)N=N1	153,14	C6H7N3O2
Z2418193634	C1CC2=C(NC=N2)C2(CCOCC2)N1	193,25	C10H15N3O
Z1389112660	NC(=O)CC1=C(F)C=CC=C1F	171,15	C8H7F2NO
Z2412196555	COC1=C(C(O)=O)C(F)=CC=C1Br	249,04	C8H6BrFO3
Z1266823324	COCC1=C(F)C=C(CN)C=C1	169,20	C9H12FNO
Z1266823338	CC(N)C(C)N1C=C(C)C=N1	153,23	C8H15N3
Z927398482	NCCC(=O)NC1CCCCC1	170,26	C9H18N2O
Z1359429514	NC1(CCO)CCCCC1	143,23	C8H17NO
Z1359429530	CC1=CN=C(S1)C(C)(C)N	156,25	C7H12N2S
Z1436181071	CC1(C)CCC(CO)CC1	142,24	C9H18O
Z1263811673	CN1CCN(CC1)C1=C(CN)C=CC=C1	205,31	C12H19N3
Z1483951161	CN1C=C(CCN)C(C)=N1	139,20	C7H13N3

Z1263529758	CC(N)C1=CC(C)=C(C)C=C1	149,24	C10H15N
Z1335709095	CC(CN)OC1=C(Br)C=CC=C1	230,11	C9H12BrNO
Z1270312194	CC(OC1CCCCC1)C(O)=O	172,22	C9H16O3
Z1404661574	CC(C)N1CC(C)C(N)C1	142,25	C8H18N2
Z1431920316	CC(C)C1CCC(N)CC1	141,26	C9H19N
Z235336615	COC1=C(C=C(F)C=C1)C(O)=O	170,14	C8H7FO3
Z223688256	CC(=O)NC1=NC(=CC=C1)C(O)=O	180,16	C8H8N2O3
Z185578978	OC(=O)C12CC(CN1)CCC2	155,20	C8H13NO2
Z1250080944	CC1=C(N)C=C(CO)C=C1	137,18	C8H11NO
Z1250080919	CC1CCN(CC1)C1=CC(N)=CC=C1	190,29	C12H18N2
Z1342206615	CC(C)S(=O)(=O)N1CCC(N)C(C)C1	220,33	C9H20N2O2S
Z2218586413	CCOC1=C(N)C=C(C)C=N1	152,20	C8H12N2O
Z1245735228	COC1=C(Cl)C=C(C=C1)C(C)O	186,64	C9H11ClO2
Z1263529766	CCOC1=C(N)C=C(F)C=C1	155,17	C8H10FNO
Z1267773652	CN(C)C1=NC(C)=C(CCN)S1	185,29	C8H15N3S
Z2240989990	CN1C=C(C=N1)C1=CC=C(O1)C(O)=O	192,17	C9H8N2O3
Z27769921	CC(=O)N1CCN(CC1)C1=CC=CC=C1	204,27	C12H16N2O
Z2238508666	N#CC1=NC(=NN1)C1CC1	134,14	C6H6N4
Z1504688330	COCC1=NN(C)C=C1C(O)=O	170,17	C7H10N2O3
Z788000702	OC(C(O)=O)C1=CC(Cl)=C(Cl)C=C1	221,03	C8H6Cl2O3
Z1201620504	COC1=C2C=CNC(=O)C2=CC=C1	175,19	C10H9NO2
Z1157836416	CC1=NC2=CC=NN2C=C1C(O)=O	177,16	C8H7N3O2
Z1101435035	OC(=O)C1CCC2=C1C=CC=C2	162,19	C10H10O2
Z1416200845	CC1CCC(CC(O)=O)C1	142,20	C8H14O2
Z1359429533	NC1CCC(CC1)N1CCCCC1	182,31	C11H22N2
Z1259339846	NC1=C2OCCOC2=CC=C1	151,17	C8H9NO2
Z1245664685	CC(N)C1=C(C)SC(C)=C1	155,26	C8H13NS
Z50145861	CC1=NN(C(=O)C1)C1=CC=CC=C1	174,20	C10H10N2O
Z1245635810	OC(C1=CC=NC=C1)C1=CC=C(F)C=C1	203,22	C12H10FNO
Z1245635739	NC1=CC(=C(Cl)C=C1)C(F)(F)F	195,57	C7H5ClF3N
Z751810922	O=S(=O)(N1CCOCC1)C1=CN=CC=C1	228,27	C9H12N2O3S
Z1245633364	COC1=C(C=CC=C1)C1=CSC(=O)N1	207,25	C10H9NO2S
Z1575325613	CC(N)C1=C(C)ON=C1C	140,19	C7H12N2O
Z1626612682	CCOC1=NC(C)=CC=C1	137,18	C8H11NO
Z133743260	CC(NS(C)(=O)=O)C1=CC(F)=C(F)C=C1	235,25	C9H11F2NO2S
Z729353192	ClC1=CC=C(CN2CCCS2(=O)=O)S1	251,74	C8H10ClNO2S2
Z1238477831	NCC1CCN(C1)C1CC1	140,23	C8H16N2
Z1020680172	NCC(O)C1=C(F)C=C(F)C=C1	173,16	C8H9F2NO
Z1127662837	O[C@H]1CCN(CC2=CC=C(F)C=C2)C1	195,24	C11H14FNO
Z1649677579	Cl.NC1(CCC2=C1C=CC=C2)C(F)(F)F	237,65	C10H11ClF3N
Z929055810	CC(C)(NC(=O)C1=COC(Br)=C1)C(O)=O	276,09	C9H10BrNO4
Z1696860273	NCC1=C(CC(O)=O)C=CC=C1	165,19	C9H11NO2
Z1312590981	NC(=O)C(O)C1CCOCC1	159,19	C7H13NO3
Z2004547966	CC1=C(C=CC=C1C#N)C(O)=O	161,16	C9H7NO2
Z1993522642	COC1=C(C=CC=C1)C1CNCCN1	192,26	C11H16N2O
Z1992316420	CC(C)(CO)N1CCNC1=O	158,20	C7H14N2O2
Z1992316412	CC1=CC(=O)C(=CN1C1CC1)C(O)=O	193,20	C10H11NO3
Z2028214428	NC1=C(CO)C=C(Cl)C(Cl)=C1	192,04	C7H7Cl2NO
Z1537047698	O=C1NC(CC2CC2)=NC2=C1C=CC=C2	200,24	C12H12N2O
Z2024187616	NCCC1=C2C=CNC2=CC=C1	160,22	C10H12N2
Z1591894147	CC(C)(CO)N1CCN(CC1)S(C)(=O)=O	236,33	C9H20N2O3S
Z1642061028	CN1N=CN=C1C1=CC=C(C=C1)C(O)=O	203,20	C10H9N3O2
Z1642061040	CNCC1=CN=C(N)C=C1	137,19	C7H11N3
Z1625822275	CC1CCCC1(N)C(N)=O	142,20	C7H14N2O
Z1582263000	CC1=CNC=C(C(O)=O)C1=O	153,14	C7H7NO3
Z1582263014	CC1=CC=CN2C(=O)C(=CN=C12)C(O)=O	204,19	C10H8N2O3
Z1575311832	COCCC1=NOC(N)=C1	142,16	C6H10N2O2
Z1575304428	NC1=NN=C(CC2CCCC2)O1	167,21	C8H13N3O
Z1575143756	CC1=C(N)C=C(S1)C(N)=O	156,20	C6H8N2OS
Z1642389904	OC(=O)CN1C=NNC1=O	143,10	C4H5N3O3
Z1642389889	OCC1(CO)CCCCC1	144,21	C8H16O2
Z1700091114	CC1=NC(OCC2=NC=CC=C2)=CC=C1	200,24	C12H12N2O
Z1607437929	CC1=NC(CCN2C=CN=C2C)=CS1	207,30	C10H13N3S
Z1139583522	CC(=C)CN1CCC2=C(C1)N=C(C)N=C2	203,29	C12H17N3
Z196024084	CN(C)C(=O)C1CCCN1CC=C(C)C	210,32	C12H22N2O
Z268316368	CCC1=NC(CN2C=NC(=N2)C#N)=CS1	219,27	C9H9N5S
Z236893404	CN(C1CCCC1)C(=O)C1=CC=CN1C	206,29	C12H18N2O

Z1815155563	CC(C)C(CN)C1=CC=NN1C	167,26	C9H17N3
Z1266823352	CC(N)CCN1CCCC1	142,25	C8H18N2
Z57102477	CCOC1=C(CN)C=CC=C1	151,21	C9H13NO
Z1238477832	CCC(CN)N1CCCC1	142,25	C8H18N2
Z54751796	CC1CC(C)CN(CC(=O)N(C)C)C1	198,31	C11H22N2O
Z1695797886	NC(C(O)=O)C1=C(F)C=CC(F)=C1	187,15	C8H7F2NO2
Z1692788303	COCC1=CC(=CN=C1)C(O)=O	167,16	C8H9NO3
Z1691545032	OC(=O)C1(CCC1)C(O)=O	144,13	C6H8O4
Z1474242573	CC1CCCN(CC1)C(=O)C1=NNC=N1	208,27	C10H16N4O
Z1664966434	O=S(=O)(C1CCCNC1)N1CCOCC1	234,31	C9H18N2O3S
Z2235790793	CCC1=C(N)NC(C)=NC1=O	153,19	C7H11N3O
Z2235681425	CC1=C(N)C=NN1C1CCOC1	167,21	C8H13N3O
Z2235678973	CC1=C2OC(N)=NC2=CC(Cl)=C1	182,61	C8H7ClN2O
Z1983181712	CC1=NC(NC(=O)C2CC=CC2)=NC=C1	203,25	C11H13N3O
Z2235386865	OCC1(CNC(=O)C1)C1=CC=CC=C1	191,23	C11H13NO2
Z2230911760	CC1=C(C=CN=C1)C(O)C1=CC=CC=C1	199,25	C13H13NO
Z2230911737	CC1=NC(C(O)=O)=C(C)O1	141,13	C6H7NO3
Z2218556016	N#CC1=CC2=C(NCCC2)C=C1	158,20	C10H10N2
Z2218555426	CC1=C(N)C=C(C(O)=O)C(Cl)=C1	185,61	C8H8ClNO2
Z2218556033	OC(=O)CC1=CSC=C1C(O)=O	186,18	C7H6O4S
Z1891776064	OCC1=CN(N=N1)C1=CC(Cl)=C(Cl)C=C1	244,08	C9H7Cl2N3O
Z2217033016	CC1(C)C(=O)NC2=C1C=CC(=C2)C(O)=O	205,21	C11H11NO3
Z2216720825	CC(=O)NC1=C(C=CC(Br)=C1)C(O)=O	258,07	C9H8BrNO3
Z1741965319	CC1=CC=C(C=C1)S(=O)(=O)N1C=CN=C1	222,26	C10H10N2O2S
Z2568757995	CNC(C(O)=O)C1=C(Cl)C(Cl)=CC=C1	234,08	C9H9Cl2NO2
Z1129283193	CC(=O)NC1=C(C)C=CN=C1	150,18	C8H10N2O
Z2527856103	CC(C)C(O)C(N)C1=CC=CC=C1	179,26	C11H17NO
Z2526439730	CCN1C(=O)NC2=C1C=CC(=C2)C(O)=O	206,20	C10H10N2O3
Z2522503408	OC1CCNC11CCCCC1	155,24	C9H17NO
Z1891775536	CN(C)C1=NC(N)=NC=C1	138,17	C6H10N4
Z2515238980	OCC#CC1=NC=C(Br)C=C1	212,05	C8H6BrNO
Z104378280	OC(C(O)=O)C1=CC=C(F)C=C1	170,14	C8H7FO3
Z53825020	CCS(=O)(=O)N1CCC(CC1)C(O)=O	221,27	C8H15NO4S
Z96092051	OC(=O)CC1=CSC(=N1)N1CCCC1=O	226,25	C9H10N2O3S
Z203038516	OC(=O)C1=NOC(=C1)C1CC1	153,14	C7H7NO3
Z90662852	CS(=O)(=O)CC1=CC(=CC=C1)C(O)=O	214,24	C9H10O4S
Z90664673	OC(=O)C1=CC2=C(OCCCO2)C(Cl)=C1	228,63	C10H9ClO4
Z57169536	COC1=C(F)C=C(C=C1)C(O)=O	170,14	C8H7FO3
Z234898245	OC(=O)CC1CCC(=O)N1	143,14	C6H9NO3
Z27797569	COC1=C(NC(=O)C2CC2C)C=CC=C1	205,26	C12H15NO2
Z56960248	CNCC1COC2=C(O1)C=CC=C2	179,22	C10H13NO2
Z56936647	C[C@H](NC(=O)C1=CC=C(F)C=C1)C(O)=O	211,19	C10H10FNO3
Z54794538	CCOC1=C(OC)C=C(C=C1)C#N	177,20	C10H11NO2
Z26824672	CCC1=NN=C(NC(=O)C2CC2)S1	197,26	C8H11N3OS
Z56913174	CC1(C)NC2=C(NC1=O)C=CC=C2	176,22	C10H12N2O
Z45527666	CNS(=O)(=O)C1=CC2=C(OCCO2)C=C1	229,25	C9H11NO4S
Z56886305	CC1=C(Cl)C=C(Cl)C(N)=N1	177,03	C6H6Cl2N2
Z56869297	CN1N(C(=O)C=C1C)C1=CC=CC=C1	188,23	C11H12N2O
Z56827661	CC1=CC(=CC=C1)C(=O)NCC(O)=O	193,20	C10H11NO3
Z19629920	CN1N=C(C(O)=O)C2=C(C=CC=C2)C1=O	204,19	C10H8N2O3
Z56791215	NC1=NC2=C(CCCC2)C(N)=N1	164,21	C8H12N4
Z56822380	OC(=O)C1=CC2=C(CCC2)S1	168,21	C8H8O2S
Z56788806	O[Cl](=O)(=O)=O.CC1=NN(C(C)=C1)C1=NC=CC=C1	273,67	C10H12ClN3O4
Z56754157	CC1=NN(CCC(N)=O)C(C)=C1	167,21	C8H13N3O
Z1429378656	CC1=CC(=O)NC=C1C(O)=O	153,14	C7H7NO3
Z168552936	CCC(=O)N(C)C(C)C1=CC=CS1	197,30	C10H15NOS
Z54849993	COC1=C2N=CC=CC2=CC=C1	159,19	C10H9NO
Z1365534337	CNCC1=CC2=C(N1)C=CC=C2	160,22	C10H12N2
Z104346732	CNCC1=C(OC)C=CC(Cl)=C1	185,65	C9H12ClNO
Z314451142	CCN(C1CC1)C(=O)C1=C(C)OC=C1	193,25	C11H15NO2
Z277959532	NCC(C1CCOC1)N1CCOCC1	200,28	C10H20N2O2
Z959151572	CC(C)N1C=C(C=N1)C(C)N	153,23	C8H15N3
Z1267882046	CN(C)C1(CN)CCOCC1	158,25	C8H18N2O
Z1309496197	CC1CCC(CCC(O)=O)O1	158,20	C8H14O3
Z262221634	C1CN(CCN1)C1=NC=CC=N1	164,21	C8H12N4
Z284779336	CC(=O)N1CCC(CC1)C1=CC=CC=C1	203,29	C13H17NO
Z1267773588	CN1CCN(C)C(CN)C1	143,23	C7H17N3

Z1267773565	CC(C)C(N)C1=C(C)C=CC=C1	163,26	C11H17N
Z57036344	C1CNC(C1)C1=CC2=C(OCCO2)C=C1	205,26	C12H15NO2
Z2108806948	CC1(CC(O)=O)CCCC1	142,20	C8H14O2
Z2092396826	CN(C)C(=O)C1CCCC(N)C1	170,26	C9H18N2O
Z1881545321	CC1=CC(=NS1)C(=O)NCC1CCCO1	226,29	C10H14N2O2S
Z1723547340	COC1=C(C=CC=C1)N1C=CC=N1	174,20	C10H10N2O
Z2017694687	CCN1CCC2NCCOC2C1	170,26	C9H18N2O
Z2004563998	NCC1CN2CCN1CCC2	155,25	C8H17N3
Z422410426	CN(C)C(=O)CNC(=O)C1=C(C)N=CS1	227,28	C9H13N3O2S
Z821829386	CN(CC(N)=O)C1CC2=C(C1)C=CC=C2	204,27	C12H16N2O
Z32064555	FC1=CC=C(C=C1)C(=O)NCC=C	179,19	C10H10FNO
Z68093999	COCC(=O)NC1=NC2=C(N1)C=CC=C2	205,22	C10H11N3O2
Z168290016	CN1C(C)=CC=C1C(=O)NC1CCCC1	206,29	C12H18N2O
Z432141618	CC(N(C)S(C)(=O)=O)C1=CC=C(C)C=C1	227,32	C11H17NO2S
Z221220996	CC(=O)N1CCC(CC1)NS(C)(=O)=O	220,29	C8H16N2O3S
Z422952360	CN(CC(N)=O)C(=O)C1=CC=C(Cl)S1	232,68	C8H9ClN2O2S
Z280480820	O=C(CN1C=CC=N1)NC1=CN=CC=C1	202,22	C10H10N4O
Z1272739430	OC(CC1CCCN1)C1=CC=CC=C1	191,27	C12H17NO
Z1318516269	CN1CC(=O)NC2=C1C=CC(Cl)=C2	196,63	C9H9ClN2O
Z1318356934	O=C1CC2(CCCC2)CN1	139,20	C8H13NO
Z193095560	CS(=O)(=O)NC1CCC2=C1C=CC=C2	211,28	C10H13NO2S
Z1318147481	CCC1=C(N)C=C(C=C1)C(O)=O	165,19	C9H11NO2
Z1313483209	COC1=C2C=CC=CC2=NC=C1C(O)=O	203,20	C11H9NO3
Z1310777667	CN1N=C(C=C1N)C1=C(C)C=CC=C1	187,25	C11H13N3
Z1297758312	OC(=O)C1=CC(=NO1)C1CCCC1	181,19	C9H11NO3
Z274553588	CC(CC(O)=O)N1N=C(C)N=C1C	183,21	C8H13N3O2
Z1295543888	CC(C)CC1CNC(=O)C1	141,21	C8H15NO
Z1278604242	COC1=NC2=C(C=CC=C2)C(=C1)C#N	184,20	C11H8N2O
Z274554372	CC1=NN(CCC(O)=O)C(C)=C1Br	247,09	C8H11BrN2O2
Z55290928	CCN(CC)C(=O)NC1=CC=C(F)C=C1	210,25	C11H15FN2O
Z1258855725	ClC1=CC=C(CN2C=CSC2=O)C=C1	225,69	C10H8ClNOS
Z733898630	CC(C)NC(=O)C1=NN(C)C(C)=C1	181,24	C9H15N3O
Z1796014543	CNC(=O)C1(CC2=C(F)C=CC=C2)CC1	207,25	C12H14FNO
Z1661748363	FC1=CC=C(CN2N=CSC2=O)C=C1	210,23	C9H7FN2OS
Z235582156	CNCC1=C(F)C=CC(=C1)C#N	164,18	C9H9FN2
Z2182115595	CCC1=NC(C)=C(CO)O1	141,17	C7H11NO2
Z32013792	CN(C)C(=O)C1=CC(=CC=C1)S(C)(=O)=O	227,28	C10H13NO3S
Z1324054938	CC1CN(CCO1)C1=NC=CC(CN)=C1	207,28	C11H17N3O
Z1318186467	NC1CCCCC1N1C=CC=N1	165,24	C9H15N3
Z2065788160	OC(=O)C1=C2CCOCC2=CO1	168,15	C8H8O4
Z2065788258	CC1=C(C(O)=O)C(=NO1)C1CC1	167,16	C8H9NO3
Z2065464360	OC1=CC(=O)N2CCNC2=N1	153,14	C6H7N3O2
Z373221060	C1CN(C1)C1=C2NC=NC2=NC=N1	175,20	C8H9N5
Z2050688057	OC(=O)C1=CN=NN1CC1=CC=CC=C1	203,20	C10H9N3O2
Z1891773873	Cl.CCOC1=CC=C(C=C1)C(C)N	201,69	C10H16ClNO
Z1791180060	CC1=NC=C(C=C1)C(=O)NC1=CN=NS1	220,25	C9H8N4OS
Z2037266949	OC(=O)CC1=C(C=CC=C1Cl)C(O)=O	214,60	C9H7ClO4
Z1822538122	FC1=CN(CC2=CSC(Br)=C2)C(=O)C=C1	288,13	C10H7BrFNOS
Z54614962	CC1(CCS(=O)(=O)C1)NC(=O)C1CCC1	231,31	C10H17NO3S
Z1874203205	CC1CCC(C)N1C1=C(Cl)C(N)=NC=N1	226,71	C10H15ClN4
Z1270387207	CC1=CC(OC(F)F)=C(S1)C(O)=O	208,18	C7H6F2O3S
Z1270384393		198,17	C10H8F2O2
Z1270358403	CC1=C(OCC(O)=O)C(Cl)=CC=C1	200,62	C9H9ClO3
Z50146693	CC1=NN(C(=O)C1)C1=CC=C(F)C=C1	192,19	C10H9FN2O
Z1270246402	COC1=CC(=CC(OC)=C1C)C(O)=O	196,20	C10H12O4
Z32368096	O=C(NC1CC1)C1=NNC2=C1C=CC=C2	201,23	C11H11N3O
Z228588366	O=S(=O)(N1CCCCC1)N1CCNCC1	233,33	C9H19N3O2S
Z1259159887	C1CC(CCN1)NC1=NN=CC=C1	178,24	C9H14N4
Z1268152412	NCC1(CCCC1)C(N)=O	142,20	C7H14N2O
Z1268152328	NCCN1N=CC2=C(C=CC=C2)C1=O	189,22	C10H11N3O
Z1267881725	O=C1CCCN1CC1=CC(=CC=C1)C#N	200,24	C12H12N2O
Z235538124	COC1=CC=C(C=C1)C1=NN=C(N)O1	191,19	C9H9N3O2
Z274575916	CC1=NC(C)=NC(=C1)N1CCNCC1	192,27	C10H16N4
Z1262246174	NC1CCN(CC1)C1=CC=C(C=C1)C#N	201,27	C12H15N3
Z1262245653	CS(=O)(=O)CC1=C(N)C=C(Cl)C=C1	219,68	C8H10ClNO2S
Z2658866526		247,11	C8H7BrO2S
Z2658863336	OC1(CNC1)C1=CC(Br)=CC=C1	228,09	C9H10BrNO

Z2588063750	OC(=O)C1=CC=NN1C1CCOCC1	196,21	C9H12N2O3
Z2587654834	CC1=NOC(=C1)C1(CC1)C(O)=O	167,16	C8H9NO3
Z2106593039	OC(=O)CN1C=C(Br)C(=N1)C1CC1	245,08	C8H9BrN2O2
Z2583036236	NC1=NC(CC2CC2)=NS1	155,22	C6H9N3S
Z2583036218	CCOC1=C(C)N=C(N)C=C1	152,20	C8H12N2O
Z2054890075	CC(C)=CCN1CCCC(O)(C1)C(N)=O	212,29	C11H20N2O2
Z2574909701	CN1C=C(N=N1)C1=CC(N)=CC=C1	174,21	C9H10N4
Z2106593715	CC1=CN2N=CC(C(O)=O)=C2N=C1	177,16	C8H7N3O2
Z729606832	CN1C=C(C=N1)N1CC(CC1=O)C(O)=O	209,21	C9H11N3O3
Z2106598193	CCN1N=C(C=C1C(O)=O)C1CC1	180,21	C9H12N2O2
Z2167281379	CC1=C(SC(=N1)C1CCCO1)C(O)=O	213,25	C9H11NO3S
Z2106601217	CC(C)C1=C(SN=N1)C(O)=O	172,20	C6H8N2O2S
Z2510259349	CC1=C2C=C(C=CN2C=N1)C(O)=O	176,18	C9H8N2O2
Z2510259461	CCC1=C(OC=N1)C(O)=O	141,13	C6H7NO3
Z1738984510	OC(=O)C1CN(C1)S(=O)(=O)C1CC1	205,23	C7H11NO4S
Z2510258170	OCC1=C(F)C=C(C=C1)C(O)=O	170,14	C8H7FO3
Z2379087719	OCC1=CN=C(C=C1)C1=NC=CC=C1	186,21	C11H10N2O
Z1561886252	CC1=CC=C(CN2CCC(C2)C(N)=O)S1	224,32	C11H16N2OS
Z2353496673	COC1=C(Br)C=C(C=N1)C(O)=O	232,03	C7H6BrNO3
Z2346401081	COC1=CC=CN2C(=O)NN=C12	165,15	C7H7N3O2
Z594413678	OC(=O)CN1C=C(Br)C=N1	205,01	C5H5BrN2O2
Z2327893661	CS(=O)(=O)N1C=C(N)C=N1	161,18	C4H7N3O2S
Z2327883941	COC1=C(N)C=C(Br)C(F)=C1	220,04	C7H7BrFNO
Z2327883919	CC1=C(C=CC(=C1)C#N)N1C=CC=N1	183,21	C11H9N3
Z2327883382	CN1C=CC(=CC1=O)C#N	134,14	C7H6N2O
Z2327673239		191,23	C11H13NO2
Z2327531717	CC1=C(C)C(=CC=C1)C(O)C1=CNC=N1	202,26	C12H14N2O
Z960282416	CCC1=NC(C)=C(S1)C(=O)NC(C)C	212,31	C10H16N2OS
Z1262691690	CC1CC(N)C(=O)N1C1=CC=CC=C1	190,25	C11H14N2O
Z1262398489	CCC1=NC(C)=NC(N)=C1	137,19	C7H11N3
Z1262398455	CNS(=O)(=O)C1=C(OC)C=C(N)C=C1	216,26	C8H12N2O3S
Z2671044820	CC1=NN=C(C)C(CO)=C1	138,17	C7H10N2O
Z90519865	CC1CCC(CC1)NC1CCC(O)CC1	211,35	C13H25NO
Z1262327462	CC1=C(NC(=O)CCN)C=CC(Br)=C1	257,13	C10H13BrN2O
Z1262254308	COC1=C2CNCCC2=NC=N1	165,20	C8H11N3O
Z1881620389	CN1C=CN=C1C1CCCNC1	165,24	C9H15N3
Z1849039509	COC1(CO)CCCCC1	144,21	C8H16O2
Z1834223476	C(OC1=CC=CC=C1)C1CNCCO1	193,25	C11H15NO2
Z1706008539	CNCC1=NC=C(C)C=C1	136,20	C8H12N2
Z1333717726	NCC1=C(F)C=CC=C1N	140,16	C7H9FN2
Z1436311340	FC1=CC2=C(NC3(CCC3)C(=O)N2)C=C1	206,22	C11H11FN2O
Z2065464333	NC1=CC2=C(C=C1)N=C(S2)C(F)F	200,21	C8H6F2N2S
Z2017679560		190,67	C8H15ClN2O
Z1945984231	CC1=NC2=C(O1)C=CC=C2C(O)=O	177,16	C9H7NO3
Z1918018570	OC1CNC2=C(C1)C=CC=C2	149,19	C9H11NO
Z1913660888	NC(=O)CN1CCC2=C(C1)C=C(N)C=C2	205,26	C11H15N3O
Z1889706359	CN1C=C(C=N1)C1=NN(C)C(N)=C1	177,21	C8H11N5
Z1839480878	COC1=C(OC)C=C2C(=O)C=CNC2=C1	205,21	C11H11NO3
Z1837073451	CC1=C(C=CC(=C1)N1C=CN=C1)C(O)=O	202,21	C11H10N2O2
Z1401276297	C1CC2(CCN(C2)C2=NC=CN=C2)CO1	205,26	C11H15N3O
Z57186564	COC1=CC=C(NC2=NCCN2)C=C1	191,23	C10H13N3O
Z2217052350	CC(C)C1CNCCCO1	143,23	C8H17NO
Z56347426	CC(N)C1=C(F)C=CC=C1	139,17	C8H10FN
Z1954439425	CC1CCC(CNC(=O)C2=CNN=C2)C1	207,28	C11H17N3O
Z1515773863	CC1=CC(Br)=C(C=C1)C(=O)N1CC(O)C1	270,13	C11H12BrNO2
Z1454840342	CC(=O)N1CCOC(CO)C1	159,19	C7H13NO3
Z46280391	NCC(=O)NC1=CC2=C(OCCO2)C=C1	208,22	C10H12N2O3
Z1891776561	CN1CCN(CCC(C)(C)N)CC1	185,32	C10H23N3
Z1079003794	CN1N=CC=C1CNC1CC1	151,21	C8H13N3
Z1552026873	OCCN1CCCCC1=O	143,19	C7H13NO2
Z1552027610	CCN1C(N)=NC2=C1C=CC(=C2)C(O)=O	205,22	C10H11N3O2
Z1532540940	CC1=NC2=CC=NN2C(C)=C1C(O)=O	191,19	C9H9N3O2
Z1532540939	COC1=CC2=C(C=C1)C(CC(O)=O)CO2	208,21	C11H12O4
Z1444783243	CC(=O)NC1=C(CC(O)=O)C=CC=C1	193,20	C10H11NO3
Z1516394657	CC(C)OC1=NC(=CC=C1)C(O)=O	181,19	C9H11NO3
Z755016692	O=C(N1CCCCCC1)C1=NC(=O)NN1	210,24	C9H14N4O2
Z1511494958	NC1=NC=C(CCC(O)=O)C=C1	166,18	C8H10N2O2

Z1501469871	CN(C)C1=NC=C(N)C=N1	138,17	C6H10N4
Z1495385201	CN1C(=O)NC2=C1C=C(C=C2)C(O)=O	192,17	C9H8N2O3
Z2405062308	CN1C=CN=C1C(N)C1CC1	151,21	C8H13N3
Z2469698599	CS(=O)(=O)C1=CNC2=C1C=CC=C2	195,24	C9H9NO2S
Z220356550	Cl.CNCC1=NN=C2CCCCCN12	216,71	C9H17ClN4
Z979897846	N#CC1=NN(CCC2=CC=CS2)C=N1	204,25	C9H8N4S
Z803153228	COC1=C(CN)C=CC(C)=N1	152,20	C8H12N2O
Z1436473525	COC1=CC=C(C=C1)C1CC(C)CN1	191,27	C12H17NO
Z1250132763	OCC1CC2=C(O1)C=CC(F)=C2	168,17	C9H9FO2
Z1257966114	CNC(=O)N1CC(CC1C)C1=CC=CS1	224,32	C11H16N2OS
Z1266823302	NCCC1=C(F)C=C(Br)C=C1	218,07	C8H9BrFN
Z1259086951	OC1CCC2=C(C1)C=CC=C2	148,21	C10H12O
Z1649880140	CCC1=NOC(C)=C1C#N	136,15	C7H8N2O
Z1245633276	O=C(CC1=CC=CC=C1)N1CCNCC1	204,27	C12H16N2O
Z228572562	CC(CN)N(C)C1=CC=CC=C1	164,25	C10H16N2
Z1263714192	OCCN1CCCC2=C1C=CC=C2	177,25	C11H15NO
Z67735551	COC1=C(F)C=C(CN(C)C(C)=O)C=C1	211,24	C11H14FNO2
Z1483947758	OC1CCC2=C(F)C=CC(F)=C12	170,16	C9H8F2O
Z268462794	CN(CC1=NC(C)=NO1)C1CCCC1	195,27	C10H17N3O
Z220654802	O=C(CCN1C=CC=N1)N1CCOCC1	209,25	C10H15N3O2
Z1945707500	NC1=CC(CCC2=CC=CO2)=NO1	178,19	C9H10N2O2
Z1945695967	NC1=NN2C(C=C1)=NN=C2C(F)(F)F	203,13	C6H4F3N5
Z1898727512	NC1=C(CN2C=CSC2=O)C=C(F)C=C1	224,25	C10H9FN2OS
Z1763483008	CN1N=CC(Cl)=C1C=CC1=NC=NC=C1	220,66	C10H9ClN4
Z1895741719	CCOC1=NC=C(N=C1)C#N	149,15	C7H7N3O
Z1702887695	CC1CN(C1)C(=O)C1=CNC(=C1)C#N	189,22	C10H11N3O
Z432840754	BrC1=C(SC=C1)C(=O)N1CCNC(=O)C1	289,15	C9H9BrN2O2S
Z138863066	CC1CCCCN1CC1=C(Cl)SN=N1	231,74	C9H14ClN3S
Z600428468	CC1=NOC(=N1)C1=CC(C)=C(N)C=C1	189,22	C10H11N3O
Z225891644	CC1=CSC(CNC(=O)C2=CC=CO2)=N1	222,26	C10H10N2O2S
Z372500342	CC1=CC=C(S1)C(=O)NC1=CNN=C1	207,25	C9H9N3OS
Z372501562	CC1=C(C=CO1)C(=O)NC1=CNN=C1	191,19	C9H9N3O2
Z183987204	O=C(CC1=CC=CS1)NC1=NC=CC=C1	218,27	C11H10N2OS
Z31067607	CN(C)C(=O)CN(C)C1=NC=CN=C1	194,24	C9H14N4O
Z31721308	ClC1=CC=C(S1)C(=O)N1CCOCC1	231,69	C9H10ClNO2S
Z332685420	CNC(=O)CNC(=O)C1=CSC(Br)=C1	277,14	C8H9BrN2O2S
Z271188802	CCC1=NC(CN2CCNC(=O)C2)=CS1	225,31	C10H15N3OS
Z220784562	ClC1=CC=C(O1)C(=O)N1CCNC(=O)C1	228,63	C9H9ClN2O3
Z1258992664	OC1(CC2=CC=CC=C2)CCCC1	176,26	C12H16O
Z1225460101	OC1CN(C1)C1=NC=C(C=C1)C(F)(F)F	218,18	C9H9F3N2O
Z409524162	CN(C(N)=O)C1=CC=CC=C1	150,18	C8H10N2O
Z1222423417	CN(C)C1=NC2=C(C=C1)C=C(N)C=C2	187,25	C11H13N3
Z230671654	O=C(NC1CC1)N1CCC2=C(C1)C=CS2	222,31	C11H14N2OS
Z285642082	O=C(NC1CC1)C1=C2N=CC=CN2N=C1	202,22	C10H10N4O
Z197276438	OCCN1CCCC(O)C1	145,20	C7H15NO2
Z239533106	CN1N=C(C)C2=C1N=CC(NC(C)=O)=C2	204,23	C10H12N4O
Z2678251679	OCCC1=CC2=C(CCC2)C=C1	162,23	C11H14O
Z137630580	CN1C=C(CNC2=CC=C(F)C=C2)C=N1	205,24	C11H12FN3
Z68247201	CN(C)C(=O)C1=C(C=CC=C1)C(F)(F)F	217,19	C10H10F3NO
Z1699556036	CN1N=C(C)N=C1NCC1=C(C)N=CS1	223,30	C9H13N5S
Z1562812679	CN(CC1=CSN=N1)C1CCCC1	197,30	C9H15N3S
Z274787914	CC1(C)CC2=C(O1)C=CC(CO)=C2	178,23	C11H14O2
Z2379767449	CC(C)CC1=CSC(=N1)C1=C(N)SN=N1	240,34	C9H12N4S2
Z2002368339	CCC(C)N1CCS(=O)(=O)CC(C)C1	219,34	C10H21NO2S
Z1747823555	CC(C)CC(=O)N1CCCC1CO	185,27	C10H19NO2
Z992717128	NC(C(O)=O)C1=CC=C(Br)C=C1	230,06	C8H8BrNO2
Z2216314212		205,26	C12H15NO2
Z2213893364	CC1=NC2=C(CCCC2)N1	136,20	C8H12N2
Z2213893403	CC1(C)CCCC1(O)CN	143,23	C8H17NO
Z1273312153	CNC(=O)C1=C2NC=CC2=CC=C1	174,20	C10H10N2O
Z2211231211	CCC1=NC(C)=C(O1)C(O)=O	155,15	C7H9NO3
Z85878916	CCC(=O)NC1=C(C=CC=C1)C(O)=O	193,20	C10H11NO3
Z2182115458	CC1=C(C(O)=O)C(C)=NS1	157,19	C6H7NO2S
Z1988116721	OCC(=O)NC1CC(C1)C1=CC=CC=C1	205,26	C12H15NO2
Z285707260	CNC(=O)CN(C)C1=NC(C)=CC(C)=N1	208,27	C10H16N4O
Z336481640	Cl.CCNCC1=NC2=C(C=CC=C2)C(=O)N1	239,70	C11H14ClN3O
Z55149005	CC1=CSC(=N1)N1CCCC1=O	182,24	C8H10N2OS

Z57101192	CN(C)C1=C2C=NN(C)C2=NC=N1	177,21	C8H11N5
Z278101784	N[C@@H](C(O)=O)C1=CC=CC=C1	151,17	C8H9NO2
Z118458680	COC1=C(C=C(Cl)C=C1)C(O)=O	186,59	C8H7ClO3
Z57790409	CCN1C(O)=CC(C)=C(C#N)C1=O	178,19	C9H10N2O2
Z256709352	CN1C=C(C)C(C(O)=O)=C1CC(O)=O	197,19	C9H11NO4
Z149205804	COC1=CC(OC)=C(C=C1)C(O)=O	182,18	C9H10O4
Z57127001	CC1=C(N)C=C(C=C1)C(O)=O	151,17	C8H9NO2
Z234895245	OC(=O)C1CCCN(C1)C1=NC=CC=C1	206,25	C11H14N2O2
Z1357827202	CCC1(C)CCC(N)CC1	141,26	C9H19N
Z1692721911	CN1N=CC=C1NCC1=C(Br)C=CS1	272,16	C9H10BrN3S
Z138328496	CNCCN1CCOCC1	144,22	C7H16N2O
Z228589394	NCC1=CC2=C(OCCO2)C(Cl)=C1	199,63	C9H10ClNO2
Z1259339759	CC(OC1CCCC1)C(O)=O	158,20	C8H14O3
Z1333717723	CC1(CN)CCCCC1O	143,23	C8H17NO
Z1333043418	OC(=O)COC1CCOC1	146,14	C6H10O4
Z2218585849	CN(C)CCN1N=CC=C1N	154,22	C7H14N4
Z1347206051	CNS(=O)(=O)C1=CC(OC)=C(Cl)C=C1	235,68	C8H10ClNO3S
Z1346385378	CCN1N=C(N)C=C1C1CC1	151,21	C8H13N3
Z1342879026	COC(C)C1=NC(C)=C(S1)C(O)=O	201,24	C8H11NO3S
Z905065930	OC(=O)C1CC2CCC(C1)N2C1CC1	195,26	C11H17NO2
Z1336457495	CCOC1=C(N=CC=C1)C(O)=O	167,16	C8H9NO3
Z1333761729	OC1CCC2=C(C1)C=NN2	138,17	C7H10N2O
Z905065908	O=C1CC2CCC(CN1)N2	140,19	C7H12N2O
Z100449516	CC1=C(C=CO1)C(=O)NCC1=CC=CO1	205,21	C11H11NO3
Z1333043381	CC1=CC2=C(OC(N)=N2)C(C)=C1	162,19	C9H10N2O
Z1333043379	NC1=NN(CC2=CC=C(Cl)C=C2)C=N1	208,65	C9H9ClN4
Z1333043373	CNC(C)(C(O)=O)C1=CC=C(F)C=C1	197,21	C10H12FNO2
Z1815155448	CC1=NN2C(CN)=NN=C2C=C1	163,18	C7H9N5
Z85891019	OC(=O)C1CCCN1C(=O)C1=CC=CS1	225,26	C10H11NO3S
Z1814381679	CNC1=C(C=CC(F)=C1)C(O)=O	169,16	C8H8FNO2
Z1693055082	CC1CN(CCS1(=O)=O)C1=NC=CC=C1	226,29	C10H14N2O2S
Z1787355378	OCC1=C(Cl)C=C(C=C1)C#N	167,59	C8H6ClNO
Z444349690	OC(=O)CC1=NN(N=C1)C1=CC=CC=C1	203,20	C10H9N3O2
Z440824948	OC(=O)CC1=CC(=O)NN1	142,11	C5H6N2O3
Z432066922	CC1=NC(=O)NC(=C1)C(O)=O	154,13	C6H6N2O3
Z431973752	COCC1=NNC(N)=C1C1=CC=CC=C1	203,25	C11H13N3O
Z31112127	N#CC1=C(NCC2=CC=CS2)N=CC=C1	215,27	C11H9N3S
Z425484984	CN1C=C(C=CC1=O)C(O)=O	153,14	C7H7NO3
Z425449752	OC(=O)CC1=CSC(=N1)N1CCNC1=O	227,24	C8H9N3O3S
Z425389558	CC1=C(C=CC(=N1)C(F)(F)F)C(O)=O	205,14	C8H6F3NO2
Z415636674	NC1CCN(CC1)C1=NC=CC=N1	178,24	C9H14N4
Z839575642	CC1=NOC(Cl)=C1CCC(O)=O	189,60	C7H8ClNO3
Z857666964	NC1=CC2=C(OC(=N2)C(F)(F)F)C=C1	202,14	C8H5F3N2O
Z839575850	CC1CCC(CC1)(NC(C)=O)C(O)=O	199,25	C10H17NO3
Z839028500	OC(=O)CN1C=CC(=O)C2=C1C=CC=C2	203,20	C11H9NO3
Z992716964	NC1=NN=C(CCC2=CC=CC=C2)O1	189,22	C10H11N3O
Z645562380	N#CC1=CN=C(NCC2=NC=CS2)C=C1	216,26	C10H8N4S
Z33545911	CCC1=C(C)SC(=C1)C(N)=O	169,24	C8H11NOS
Z225892438	CC1CC1C(=O)NCC1=NC(C)=CS1	210,30	C10H14N2OS
Z32366804	CC1=NC(CC(=O)NC2CC2)=CS1	196,27	C9H12N2OS
Z437351256	CC1=CC=C(CNC(=O)C2=CC=CO2)S1	221,27	C11H11NO2S
Z432725268	BrC1=C(SC=C1)C(=O)N1CCOCC1	276,15	C9H10BrNO2S
Z384362790	O=C(NCC1CCOC1)C1=NC=CN=C1	207,23	C10H13N3O2
Z1832147138	ClC1=CN=C(NCC2=CSN=N2)C=C1	226,68	C8H7ClN4S
Z169851686	CC1=CC(C)=C(N1)C1=CC=NC=C1	172,23	C11H12N2
Z2106600022	CN1C=CC(=N1)N1CC(CC1=O)C(O)=O	209,21	C9H11N3O3
Z2510258114	CC(O)(CN1C=CC=CC1=O)C(O)=O	197,19	C9H11NO4
Z240127660	CC1=NN=C(O)C(N1)=CC1=CC=CC=C1	201,23	C11H11N3O
Z57842305	CC1=NC2=C(C(N)=NS2)C(C)=C1	179,24	C8H9N3S
Z223656256	CC(N1C=CC=C1)C(O)=O	139,15	C7H9NO2
Z135439900	CCN(CC)C(=O)C1=CC(C)=NO1	182,22	C9H14N2O2
Z1259086957	OCCN1CCC2=C(C1)C=CC=C2	177,25	C11H15NO
Z1267773500	CN1N=C(C)C(C2CCCN2)=C1C	179,27	C10H17N3
Z1465369619	CC1=C(C=CC(F)=C1)C(=O)N1CCCC1	207,25	C12H14FNO
Z228589876	CC(C)C(N)C1=NN=C2C=CC=CN12	190,25	C10H14N4
Z1415893926	CN1N=C(C)C(OCC(O)=O)=C1C	184,20	C8H12N2O3
Z228584062	COC1=C(CN2CCNCC2)C=CC=C1	206,29	C12H18N2O

Z1263820389	OCCN1C(=O)NCC2=C1C=CC=C2	192,22	C10H12N2O2
Z228569070	CC(CN)N1CCN(C)CC1	157,26	C8H19N3
Z2213893395	NC1=NC(C2CC2)=C(Br)C=C1	213,08	C8H9BrN2
Z26823525	CCC1=NN=C(NC(=O)C2CCCO2)S1	227,28	C9H13N3O2S
Z285925540	CC1=C(F)C=C(C=C1)C(=O)NCCN	196,23	C10H13FN2O
Z86430604	O=C(NC1CCS(=O)(=O)C1)C1CCCC1	231,31	C10H17NO3S
Z234898151	CC(=O)NC1(CCCC1)C(O)=O	171,20	C8H13NO3
Z234898083	O=C1NCCOC2=C1C=CC=C2	163,18	C9H9NO2
Z118257746	NC1=CC(=CC=C1)C(=O)NC1CC1	176,22	C10H12N2O
Z234897391	NCC1=CC=C(CN2CCCC2=O)C=C1	204,27	C12H16N2O
Z32412988	CC1=NC(CC(=O)NC2CCCC2)=CS1	224,32	C11H16N2OS
Z104753984	CC1=CC(Cl)=C(NCC(N)=O)C=C1	198,65	C9H11ClN2O
Z26743843	COC1=C(Cl)C=C(NC(=O)C2CC2)C=C1	225,67	C11H12ClNO2
Z220616780	C1CNCC(C1)C1=NC2=C(N1)C=CC=C2	201,27	C12H15N3
Z220383530	CN1NC(=O)C2=C(N=CC=N2)C1=O	178,15	C7H6N4O2
Z1750885079	CN1C=NN=C1NC(=O)C1CCCCO1	210,24	C9H14N4O2
Z111634624	CC1=NN2C(NC(C)=C(C)C2=O)=N1	178,20	C8H10N4O
Z1860991525	COC1=C(N)C=C(Cl)C(Cl)=C1	192,04	C7H7Cl2NO
Z1889710301	CN1CCC2=C(C1)C(=O)NC(C)=N2	179,22	C9H13N3O
Z1222278672	CN(C)C(C)(C(O)=O)C1=CC=C(F)C=C1	211,24	C11H14FNO2
Z1222278659	CC(C)C1=C(Br)C(=NN1)C(O)=O	233,07	C7H9BrN2O2
Z1166180442	CC1CN(C)CCN1C(=O)C1=CSN=N1	226,30	C9H14N4OS
Z433657690	CN(C)C(=O)CNC(=O)C1=C(Br)C=CS1	291,16	C9H11BrN2O2S
Z1022803746	CNC(=O)C(C)(C)C1=CC(Br)=CC=C1	256,14	C11H14BrNO
Z1836339155	C1CC(C1)C1=NC(=CS1)C1=NC=CC=C1	216,30	C12H12N2S
Z300114476	CC1CC(C)CN(C1)S(=O)(=O)N(C)C	220,33	C9H20N2O2S
Z1171979208	CNC(=O)C1(CCC1)C(O)=O	157,17	C7H11NO3
Z1205221212	CN(C)C(=O)C(C)(C)NC1CCCC=C1	210,32	C12H22N2O
Z1262237488	CC1=CC(=CC=C1)C(C)(C)CN	163,26	C11H17N
Z1269702257	CC(CN)OC1=CC=C(Br)C=C1	230,11	C9H12BrNO
Z1220645147	CC(C)N1C=CC(CN2C=C(Cl)C=N2)=N1	224,69	C10H13ClN4
Z1103238697	CC(C)CN1CCCC1C1=CN(C)N=C1	207,32	C12H21N3
Z1262237327	OC(=O)C1=C(OC2CCC2)C=CC=C1	192,21	C11H12O3
Z2217052364	FC1=CN=C(C=C1)C1CCCCN1	180,23	C10H13FN2
Z2509351114	OC(=O)C1=CC2=C(COCC2)C=C1	178,19	C10H10O3
Z1955122823	COC1=C(N=CC=C1)C#N	134,14	C7H6N2O
Z2179017020	CC1=NC(NC2CC2)=CC(=C1)C#N	173,22	C10H11N3
Z2507919897	C1CC(CCO1)C1=NC=CN1	152,20	C8H12N2O
Z2507870689	CC1=NC(N)=C(C(N)=O)C(C)=C1	165,20	C8H11N3O
Z2492774535	CC(C)CCC1=NN=C(N)S1	171,26	C7H13N3S
Z2492774459	CC(CC(O)=O)N1CCC2=C1C=CC=C2	205,26	C12H15NO2
Z2379767265	CC(C)CC1=CSC(=N1)C1=NN=CN1	208,28	C9H12N4S
Z2379767447	NC1=C(N=NS1)C1=NC(=CS1)C1CC1	224,30	C8H8N4S2
Z2472860002	NC(C(O)=O)C1=C(F)C=C(F)C=C1	187,15	C8H7F2NO2
Z2088385280	N#CC1=NC=CC(=C1)N1CCCCC1	187,25	C11H13N3
Z1269799982	CC1=CC=C(O1)C(=O)NCC1=CC=CN1	204,23	C11H12N2O2
Z1238477757	CN(C)C(=O)N1CCNCC1	157,22	C7H15N3O
Z71177490	CNC1=C(C=CC=N1)C(O)=O	152,15	C7H8N2O2
Z2471262531	OCC1=NN2C=CC=NC2=N1	150,14	C6H6N4O
Z2510258098	CC1NC2=C(NC1=O)C=CC=C2C(O)=O	206,20	C10H10N2O3
Z2510258288	OC(=O)C1=NC=CC(=C1)C1CC1	163,18	C9H9NO2
Z2510258172	CC(C)C1=NC(C)=C(O1)C(O)=O	169,18	C8H11NO3
Z2509351168	COC1=CC=C(C=C1)C1=NC(N)=NC=C1	201,23	C11H11N3O
Z2509351196	CC(C(N)C(O)=O)C1=CC=CC=C1	179,22	C10H13NO2
Z1982493931	OC(=O)CC1CC(C1)C1=CC=CC=C1	190,24	C12H14O2
Z353247326	CC(CO)NC(=O)C1=C(Cl)C=C(F)C=C1	231,65	C10H11ClFNO2
Z1966493094	CC(C)(C1CCOCC1)C(O)=O	172,22	C9H16O3
Z1966492233	CC(N)(C(O)=O)C1=C(F)C=CC(Br)=C1	262,08	C9H9BrFNO2
Z1966487993	CCC1NC(=O)CCN(C)C1=O	170,21	C8H14N2O2
Z1948935031	COC1=CC=C(C=C1)C1=C(N)N=CC=N1	201,23	C11H11N3O
Z1947444015	CCC(C)C1=NC=C(N)C=N1	151,21	C8H13N3
Z839706072	CN(C1CC1)C(=O)CN1C=C(Br)C=N1	258,12	C9H12BrN3O
Z29418354	CC(C)C(=O)NC1=CC(Cl)=C(C=C1)C#N	222,67	C11H11ClN2O
Z1272426173	CN1CCC(NC2=NC(C)=NS2)C1=O	212,27	C8H12N4OS
Z1983080486	OC1CCC(CC1)N1CCCC1=O	183,25	C10H17NO2
Z360008080	BrC1=CN(CC(=O)N2CCC2)C(=O)C=C1	271,11	C10H11BrN2O2
Z1664380857	COC1=NC(N)=C(N)C=C1	139,16	C6H9N3O

Z1632890379	NC(CC(O)=O)C1=CC(Cl)=CC(Cl)=C1	234,08	C9H9Cl2NO2
Z915781066	CCC(C)(C)NC(=O)C1=CN=C(C)S1	212,31	C10H16N2OS
Z235344291	CC1=C(SC2=C1C(C)=NC(C)=N2)C(O)=O	222,26	C10H10N2O2S
Z403317908	COCC(=O)NC1CCCCNC1=O	200,24	C9H16N2O3
Z56948440	CC(C)C(N)C1=NC2=C(N1)C=CC=C2	189,26	C11H15N3
Z56822248	OC(=O)CNC(=O)C1=C(F)C=CC=C1	197,17	C9H8FNO3
Z57967260	O=C(NC1=NN=CS1)C12CCC(CC1)C2	223,29	C10H13N3OS
Z56782009	COC1=C(N)C=C(Cl)C=C1	157,60	C7H8ClNO
Z56764669	OC1CS(=O)(=O)CC1N1CCCCC1	219,30	C9H17NO3S
Z56763571	CN(C)C(=O)NC1CCS(=O)(=O)C1	206,26	C7H14N2O3S
Z1230023781	CC(=O)NC1=C2N=CNC2=CC=C1	175,19	C9H9N3O
Z2692095039	OC(=O)C1=CC=C(O1)C(F)F	162,09	C6H4F2O3
Z2692095137		152,20	C7H12N4
Z409512682	CC1CCCN(C1)C(=O)NC1=NN=CS1	226,30	C9H14N4OS
Z744843848	CC1=NN(C(=O)C1)C1=CC(N)=CC=C1	189,22	C10H11N3O
Z228589684	COC1=C(N)C(C(O)=O)=C(C)C=C1	181,19	C9H11NO3
Z1741957061	OC(=O)C1=C(C=C(F)C=C1)C(F)(F)F	208,11	C8H4F4O2
Z1749294408	ClC1=C(CN2N=CSC2=O)N=NS1	234,68	C5H3ClN4OS2
Z1839480414		170,21	C9H14O3
Z1348371854	N#CC1=NC=C(C=C1)N1CCCOCC1	203,25	C11H13N3O
Z1348403112	CCC1=NSC(NC2CCN(C)C2=O)=N1	226,30	C9H14N4OS
Z1744194310	COC1=CC(OC)=C(Br)C=C1NC(C)=O	274,11	C10H12BrNO3
Z1699554930	CN1N=C(C)N=C1NCC1=CC=CS1	208,28	C9H12N4S
Z1331708554	O=C(NCC1=CNN=N1)C1CCCCC1	208,27	C10H16N4O
Z1824511110	CC1=C(OC(CC2CC2)=N1)C(O)=O	181,19	C9H11NO3
Z1627023416	CC1=NN(CCC(N)=O)C=C1Br	232,08	C7H10BrN3O
Z1258992483	CCC1=NNC(=O)C(C#N)=C1CC	177,21	C9H11N3O
Z31602731	CC1=C(SC=C1)C(=O)N1CCNC(=O)C1	224,28	C10H12N2O2S
Z822382694	CS(=O)(=O)CC(O)C1=CC=CC=C1	200,25	C9H12O3S
Z1255523318	OCCNC(=O)C1=NC=C(Br)C=C1	245,08	C8H9BrN2O2
Z1123870588	CNC(=O)COC1=C(F)C=CC(F)=C1	201,17	C9H9F2NO2
Z1250132721	CC(N)(CO)C1CCCCC1	157,26	C9H19NO
Z1250132677	NC1=C(OC(F)F)C=CC(=C1)C#N	184,15	C8H6F2N2O
Z1250132631	NCC1=CC(=CC=C1)N1CC=CC1	174,25	C11H14N2
Z1250132344	CN1N=NC2=C1C=CC(CO)=C2	163,18	C8H9N3O
Z1250132320	CC1=NC2=C(N1)C=C(Br)C=N2	212,05	C7H6BrN3
Z1196037098	OCCC1CCCN(C1)C1CCCC=C1	209,33	C13H23NO
Z816005728	CCNC(=O)N(C)CC1=CSC(Br)=C1	277,18	C9H13BrN2OS
Z1259339957	CC(C)C1=C(C=NC(C)=N1)C(O)=O	180,21	C9H12N2O2
Z275170094	CCN1C=C(C=N1)C(O)=O	140,14	C6H8N2O2
Z275170014	CN1C=C(C(O)=O)C(C)=N1	140,14	C6H8N2O2
Z1259335802	CC1=C(C)C(=O)N=C(N1)C1=NC=CC=C1	201,23	C11H11N3O
Z1259273393		198,17	C10H8F2O2
Z85886697	OC(=O)CNC(=O)C1=C(Cl)C=CC=C1F	231,61	C9H7ClFNO3
Z1259273098	NC(C(O)=O)C1=C(F)C=CC=C1	169,16	C8H8FNO2
Z2689031620	OCC1=NN=C(S1)C1CC1	156,20	C6H8N2OS
Z1259339726	CC(O)(CN)C1=CC=CO1	141,17	C7H11NO2
Z1984948429	CC1(COC1)C(=O)NC1=CC(F)=CN=C1	210,21	C10H11FN2O2
Z1578034630	CN1C=C(C=N1)C(C)(O)CN	155,20	C7H13N3O
Z106307058	CC(C)N1CCN(CC1)C(=O)C(C)C	198,31	C11H22N2O
Z2527680084	CC1=CC(C(O)=O)=C(C)N1C1CCOC1	209,25	C11H15NO3
Z1994237215	FC(F)(F)C1=CC(CN2CCCC2)=NN1	219,21	C9H12F3N3
Z383325512	CC(=O)NCC1=CNN=C1	139,16	C6H9N3O
Z1171979275	CN1C=CC2=C1C=CC=C2C(O)=O	175,19	C10H9NO2
Z1171978796	CS(=O)(=O)C1=C(SC=C1)C(O)=O	206,23	C6H6O4S2
Z57335261	OC(=O)CN1CC2=C(C1)C=CC=C2	177,20	C10H11NO2
Z1171978814	OC(=O)CC1=COC(=N1)C1=CC=CS1	209,22	C9H7NO3S
Z1171978854	CC(C)CC1=NC(=CS1)C(O)=O	185,24	C8H11NO2S
Z32016484	CNC(=O)C1=CC=C(Cl)S1	175,63	C6H6ClNOS
Z438096750	CC(=O)N1CCCC(CNS(C)(=O)=O)C1	234,31	C9H18N2O3S
Z1162910729	OC(C1CCNCC1)C1=CC=C(Cl)C=C1	225,72	C12H16ClNO
Z296759562	CC1=NOC(=N1)C1=CC=C(C=C1)C(N)=O	203,20	C10H9N3O2
Z203520994	ClC1=C(C=C(NC(=O)C2CC2)C=C1)C#N	220,66	C11H9ClN2O
Z19739280	CN(C)C(=O)COC1=CC=C(C=C1)C#N	204,23	C11H12N2O2
Z1160900191	NCCN1C(=O)CC2=C1C=CC=C2	176,22	C10H12N2O
Z1770193433	CC1=CC(=CS1)C1=C(N)C=CC=N1	190,26	C10H10N2S
Z1768428824	OC(=O)C1=CC(=NC=C1)C1CC1	163,18	C9H9NO2

Z1768160437	CC(=O)NC1(CCCCCC1)C(O)=O	199,25	C10H17NO3
Z1259335792	OC(=O)C1=NC(=NC=C1)C1CC1	164,16	C8H8N2O2
Z1762997314	CC1=C(Cl)C=CC(=C1)C(N)C(O)=O	199,63	C9H10ClNO2
Z1762772460	CN(CC(O)=O)C1=CC=NC=C1	166,18	C8H10N2O2
Z1079512010	CC1=NOC(CN2N=C(C)C=CC2=O)=C1	205,22	C10H11N3O2
Z2467208649	CC(C)NC1=NC2=C(N1)C=CC=C2	175,24	C10H13N3
Z1603606316	OC(=O)C1(CC1)NC(=O)C1=CC=C(Cl)S1	245,68	C9H8ClNO3S
Z1715153919	CN1C(CCC1=O)C(O)=O	143,14	C6H9NO3
Z2211246663	COCCN1N=NC2=C1CCNC2	182,23	C8H14N4O
Z1263714198	C(N1CCNCC1)C1=NOC=C1	167,21	C8H13N3O
Z45537374	O=S(=O)(NC1CCCC1)C1=CC=CS1	231,33	C9H13NO2S2
Z2065616520	CCNC1CCN(CC1)C1=CC=NC=C1	205,31	C12H19N3
Z2073741691	C[C@H]1CN(C[C@@H](C)O1)C(=O)COC(F)F	223,22	C9H15F2NO3
Z1583292089	CC1=NN=C(CNC2=CC(F)=CC=C2)S1	223,27	C10H10FN3S
Z2146515384	COC(C)(C)C1=NC(C)=C(S1)C(O)=O	215,27	C9H13NO3S
Z85923165	OCC1=CNC2=C1C=CC=C2	147,18	C9H9NO
Z2111525966	Cl.Cl.NC1CCCN(CC(F)(F)F)C1	255,11	C7H15Cl2F3N2
Z2111526094	CN(C)C1=C(N=CC=C1)C#N	147,18	C8H9N3
Z2096659384	OC(=O)CCC1=C2C=CC=CC2=CN=C1	201,23	C12H11NO2
Z1273141584	NC(=O)C1=CC=C(OC2CCC2)C=C1	191,23	C11H13NO2
Z1218259484	CC1=C2CCN(CC(O)=O)CC2=CC=C1	205,26	C12H15NO2
Z2160887284	C1CCC2NCCNC2C1	140,23	C8H16N2
Z2160887173	NC(=O)C1CCC1C(O)=O	143,14	C6H9NO3
Z2160887463	COC1=CNC(C)=NC1=O	140,14	C6H8N2O2
Z1438447167	CCN1CCC2=C(C1)C=CC=C2N	176,26	C11H16N2
Z1348417096	CN(CC1CCOC1)C1=C(Cl)C=NC=C1	226,70	C11H15ClN2O
Z1694645445	NC1CCOC(C1)C1CC1	141,21	C8H15NO
Z265638758	NCC1=C(OC(F)F)C=CC(Cl)=C1	207,60	C8H8ClF2NO
Z1230130478	CC(C)NC1=C(C=CC=N1)C(=O)N(C)C	207,28	C11H17N3O
Z212232234	CCC1=NN=C(CN2CCCC(C)C2)O1	209,29	C11H19N3O
Z1497321453	CC1=NC(C(=O)N2CCCC2)=C(C)S1	210,30	C10H14N2OS
Z1359502847	C1CN(C2CCCCC12)C1=NC=CN=C1	203,29	C12H17N3
Z1130244753	OC[C@@H]1CCCN1CC1=C(Cl)SN=N1	233,71	C8H12ClN3OS
Z1723418946	CC1=C(C=CC(N)=C1)C1CC1	147,22	C10H13N
Z1266823327	CC1=C(CCN)C(C)=NO1	140,19	C7H12N2O
Z1238477877	CC1=CC=C(O1)C1CCCN1	151,21	C9H13NO
Z234895145	NCC1CCCC2=C1C=CC=C2	161,25	C11H15N
Z2065543066	CC(C)OC1=CC2=C(NC=C2CN)C=C1	204,27	C12H16N2O
Z1276496842	CC(N)C1=CC(=CC=C1)C#N	146,19	C9H10N2
Z1142870697	CCC(CN)N1CCC2=C(C1)C=CC=C2	204,32	C13H20N2
Z422516090	CC1=C(SC=N1)C(=O)NCC1CCC1	210,30	C10H14N2OS
Z45527702	CNS(=O)(=O)C1=CC=C(Cl)S1	211,68	C5H6ClNO2S2
Z57229604	CC(C)(OC1=CC=C(Br)C=C1)C(O)=O	259,10	C10H11BrO3
Z373771058	CN1C=C(NC(=O)C2=CSC(Br)=C2)C=N1	286,15	C9H8BrN3OS
Z927756816	CC1=CN=C(NC(=O)CCN)C=C1	179,22	C9H13N3O
Z375692646	CN(CC1=NOC(C)=C1)C(=O)C1CCC1	208,26	C11H16N2O2
Z90507763	CC1CCC(CC1)NCC1=CN=CC=C1	204,32	C13H20N2
Z220996120	CC(NC(=O)C1CC1)C1=CN=CC=C1	190,25	C11H14N2O
Z31720228	CC1=NC(CC(=O)N2CCOCC2)=CS1	226,29	C10H14N2O2S
Z208155164	NC1CCC2=C1C=CC=C2	133,19	C9H11N
Z458888310	CC1=C(N=C(O1)C1=CC=CS1)C(N)=O	208,24	C9H8N2O2S
Z1891776347	OCC1CCCCN1C1=NC=NC=C1	193,25	C10H15N3O
Z1273141646	COCC1=CC(=NO1)C(N)=O	156,14	C6H8N2O3
Z2327039876	CC1=CC2=CNC(=O)C=C2C=C1	159,19	C10H9NO
Z2309759195	CN1N=CC=C1C1=NN=C(N)C=C1	175,20	C8H9N5
Z2309759186	NC1=CC(=CC=C1)C1CCC(=O)N1	176,22	C10H12N2O
Z2306625664	OC1=NC2=C(CCCC2)N=C1	150,18	C8H10N2O
Z2301438417	CN1N=C(C)C2=C1C(O)=NN=C2	164,17	C7H8N4O
Z2293607285	OCC1(CCOCC1)C(O)=O	160,17	C7H12O4
Z2284393895	CN1C(CC(O)=O)CCC1=O	157,17	C7H11NO3
Z1267881643	CC1NC2=C(NC1=O)C=CC=N2	163,18	C8H9N3O
Z1267773729	CC(C)S(=O)(=O)CC1=CC(N)=CC=C1	213,30	C10H15NO2S
Z1266933803	OC(=O)CN1CCOC2=C1C=C(Cl)C=C2	227,64	C10H10ClNO3
Z1258519607	CC1=CN=C(O1)C1=CC(N)=CC=C1	174,20	C10H10N2O
Z1266854838	CC(C)C1=NN(C)C(N)=N1	140,19	C6H12N4
Z1266854833	CC(C)(C1=NN=C(N)O1)C1=CC=CC=C1	203,25	C11H13N3O
Z1266854824	COC1=C(N)C(=CC(F)=C1)C(O)=O	185,15	C8H8FNO3

Z1263820422	CN1C=C(C=N1)C1=NN=C2CNCCN12	204,24	C9H12N6
Z1263820367	OCC(=O)N1CCOCC1	145,16	C6H11NO3
Z111583018	COC1=CC=C(C=C1)C1=NC(CN)=NO1	205,22	C10H11N3O2
Z1263716020	CN(C)C1=CC(=CC=C1)C1=NN=C(N)O1	204,23	C10H12N4O
Z1263602457	CCC1=CC=C(C=C1)C1=NOC(N)=C1	188,23	C11H12N2O
Z1263602430	CC(C)C1NCCNC1=O	142,20	C7H14N2O
Z2233919746	CC1=NSC(NCC2=C(C)N=CO2)=N1	210,26	C8H10N4OS
Z2417927388	CC1=CC(=O)N(CCC2(O)CCC2)C=C1	207,27	C12H17NO2
Z2234185684	O=C1CCCC(N1)C1=CC=CC=C1	175,23	C11H13NO
Z1413780550	CC(C)C(N)C1=C(F)C=C(F)C=C1	185,22	C10H13F2N
Z1569715342	COCCC1(N)CCCC1	143,23	C8H17NO
Z275189668	NC1=C(F)C=CC(=C1)C(F)(F)F	179,12	C7H5F4N
Z1263529765	NCC1=CC2=C(OCCCO2)C=C1	179,22	C10H13NO2
Z275728064	CCC1=NOC(=N1)C(C)N1CCOCC1	211,27	C10H17N3O2
Z90517862	CC1=CC=C(CNC2CC2)O1	151,21	C9H13NO
Z1354376234	OC1CN(C1)C1=C(F)C=C(Cl)C=N1	202,61	C8H8ClFN2O
Z1250120913		196,67	C11H13ClO
Z1575143883	CC1=NOC(=N1)C(N)CO	143,15	C5H9N3O2
Z744843378	CC1=C(N=NN1C1=CC=CC=C1)C(O)=O	203,20	C10H9N3O2
Z744754626	COC1=C(C=CC(C)=N1)C#N	148,17	C8H8N2O
Z2179640362	CC1=C(N=CC=N1)N1CCC=C(F)C1	193,23	C10H12FN3
Z1373447292	CN(CC1=NC=CS1)C1=C(F)C=CC=N1	223,27	C10H10FN3S
Z2272040458	FC1=CCCN(C1)C(=O)C1=NSC=C1	212,24	C9H9FN2OS
Z1798482785	CC1CCN(C1CO)C1=C(F)C=CC=N1	210,25	C11H15FN2O
Z1270783121	CN(C)S(=O)(=O)C1=C(C)C(F)=CC=C1	217,26	C9H12FNO2S
Z1583292057	CC1=NN=C(CNC2=CC=C(F)C=C2)S1	223,27	C10H10FN3S
Z172492308	NCC1=C(OC(F)(F)F)C=CC=C1	191,15	C8H8F3NO
Z2272532508	NC1=CC(CC2CC2)=NN1	137,19	C7H11N3
Z2235831703	CN1C=C(N)C(=N1)C(=O)NC1CCCC1	208,27	C10H16N4O
Z2235831719	CN1C=C(N)C(=N1)C(=O)NC1CCC1	194,24	C9H14N4O
Z31424008	O=C(CC1=CC=CS1)N1CCCCC1	209,31	C11H15NOS
Z1255451841	NC1CCC2=C(CC1)C=CC=C2	161,25	C11H15N
Z1849012580	COC1(CCCC1)C(O)=O	144,17	C7H12O3
Z2235681153	COC1COCCC1CC(O)=O	174,20	C8H14O4
Z2309761167	COCC1CCC(N)CC1	143,23	C8H17NO
Z2442270563	C(C1CCNCC1)C1=NC(=NO1)C1CC1	207,28	C11H17N3O
Z2240988424	COCC1(CN)CCCC1	143,23	C8H17NO
Z2293643743	COCC1(CCCC1)C(O)=O	158,20	C8H14O3
Z85935506	OCC1=CC2=C(OCCO2)C(Cl)=C1	200,62	C9H9ClO3
Z57054208	CC1=CC=C(CN2N=CC=C2N)O1	177,21	C9H11N3O
Z85923459	CC(=O)N1CCC[C@H]1C(O)=O	157,17	C7H11NO3
Z85920643	CC1=NC2=C(O1)C=CC(N)=C2	148,17	C8H8N2O
Z27749489	CC(=O)NCC1=CC=CC=C1	149,19	C9H11NO
Z57900336	COC1=CC(OC)=C(OC)C=C1C#N	193,20	C10H11NO3
Z57980675	CC1=C(CN2N=CC=C2N)SC=C1	193,27	C9H11N3S
Z57049319	CCNC1=NN=C(S1)C1=CC=NC=C1	206,27	C9H10N4S
Z57047005	CC(C)(OC1=CC=C(F)C=C1)C(O)=O	198,19	C10H11FO3
Z57041992	CC1=NN(N=C1C(O)=O)C1=CC=CC=C1	203,20	C10H9N3O2
Z57002442	Cl.CN(C)S(=O)(=O)C1=C(Cl)C=CC(N)=C1	271,16	C8H12Cl2N2O2S
Z55692894	CC1=NC2=C(N1)C=CC=C2	132,17	C8H8N2
Z823291756	CC1CN(CCO1)C1=NC=C(CN)C=C1	207,28	C11H17N3O
Z45527656	CNS(=O)(=O)C1=CC=CS1	177,24	C5H7NO2S2
Z815264188	OC(=O)C1=CC2=C(NC(=O)C2)C=C1	177,16	C9H7NO3
Z815264300	CC1=C(F)C=C(C=C1)C1=NN=C(N)O1	193,18	C9H8FN3O
Z815264098	CC1=C(C=CC=C1N)C(=O)NCCO	194,23	C10H14N2O2
Z815264032	COC1=CC(N)=C(NC(C)=O)C=C1	180,21	C9H12N2O2
Z396120220	CN1C=C(NC(=O)CC2=CC=CS2)C=N1	221,28	C10H11N3OS
Z332767782	CNC(=O)CN(C)C(=O)C1=CSC(Br)=C1	291,16	C9H11BrN2O2S
Z351739896	CN(C)S(=O)(=O)N1CCN(CC1)C(C)=O	235,30	C8H17N3O3S
Z25081029	NC(=O)COC1=C(Br)C=C(F)C=C1	248,05	C8H7BrFNO2
Z79434398	ClC1=CC=C(O1)C(=O)N1CCOCC1	215,63	C9H10ClNO3
Z752989054	CC1=NN2C=C(C=NC2=C1)C#N	158,16	C8H6N4
Z752371208	N#CC1=CN=C(C=C1)N1CCNCC1	188,23	C10H12N4
Z19727416	CC(OC1=CC=C(Cl)C=C1)C(N)=O	199,63	C9H10ClNO2
Z228465246	FC1=CC(CN2C=NC(=N2)C#N)=CC=C1	202,19	C10H7FN4
Z57451303	CC(=O)NC1C2CC3CC(C2)CC1C3	193,29	C12H19NO
Z31490284	CC1=CC=C(S1)C(=O)NCC1CCCO1	225,31	C11H15NO2S

Z394693564	CS(=O)(=O)NC1CCCNC1	178,25	C6H14N2O2S
Z285782398	CN(C)C1=NC=CC(=C1)C(O)=O	166,18	C8H10N2O2
Z381429064	CC1=C(C(=CC=C1N)C(N)=O	150,18	C8H10N2O
Z228463944	FC1=CC=C(CN2C=NC(=N2)C#N)C=C1	202,19	C10H7FN4
Z344080996	ClC1=CC=C(CNC(=O)C2=CC=CO2)S1	241,69	C10H8ClNO2S
Z219098968	CN(CCO)C1=NC=C(C=C1)C#N	177,21	C9H11N3O
Z367678734	CN(C)C1=NC=CC(=C1)C#N	147,18	C8H9N3
Z367451180	OC(=O)CN1C=CC=NC1=O	154,13	C6H6N2O3
Z367450968	CC1=CC=C(O1)C1=NC(CC(O)=O)=CS1	223,25	C10H9NO3S
Z63851050	ClC1=CC=C(O1)C(=O)NCC1=CC=CS1	241,69	C10H8ClNO2S
Z2469998600	O=C1CCC2=C(N1)N=CC=C2	148,17	C8H8N2O
Z2469606500	COC1=CC2=C(C=C1)C(=O)NCCO2	193,20	C10H11NO3
Z2466617840	CN1C(C)=CC(N)=C(N)C1=O	153,19	C7H11N3O
Z2442102818	OC(=O)C1CCOC2=C1C=C(Br)C=C2	257,08	C10H9BrO3
Z2007151852	CC(O)CS(=O)(=O)C1=C(C)C=C(C)C=C1	228,31	C11H16O3S
Z2412196571	CN1N=NC2=C1C=CC(N)=C2	148,17	C7H8N4
Z1266823370	CC(N)CN1C=C(C)C=N1	139,20	C7H13N3
Z1263529811	CCN1CCC(CCN)CC1	156,27	C9H20N2
Z1436181107	CC(N)CC1=CC(Br)=C(F)C=C1	232,10	C9H11BrFN
Z1269750463	CC1COC2=C(N1)C=CC=C2	149,19	C9H11NO
Z1269702290	CCC1=CC=C(O1)C1CCCN1	165,24	C10H15NO
Z235336609	COC1=CC(Br)=C(OC)C=C1C(O)=O	261,07	C9H9BrO4
Z223688272	CN(C(C)=O)C1=C(C=CC=C1)C(O)=O	193,20	C10H11NO3
Z234855718	OC(=O)C1=CC2=C(OC1)C=CC(Br)=C2	255,07	C10H7BrO3
Z45509018	FC1=CC=C(C=C1)S(=O)(=O)N1CCCC1	229,27	C10H12FNO2S
Z33546508	NC(=O)CC1=CC=CC=C1	135,17	C8H9NO
Z169856680	CC1=CC=C(S1)S(=O)(=O)N1CCNCC1	246,34	C9H14N2O2S2
Z53111117	OC(=O)CCN1C=CN=C1	140,14	C6H8N2O2
Z1250080950	CC1=C(C)C(=CC=C1)C(N)CC(O)=O	193,25	C11H15NO2
Z335931386	OCCN1C=NC2=C(SC=C2)C1=O	196,22	C8H8N2O2S
Z1250080895	CCC1=NN=C(CO)N1C	141,17	C6H11N3O
Z1238477814	CC(C)C(O)C1=NC=CN1C	154,21	C8H14N2O
Z319545618	CC1=NC=CN1CC1=CC(=CC=C1)C#N	197,24	C12H11N3
Z274553584	CC(CC(O)=O)N1N=C(C)C=C1C	182,22	C9H14N2O2
Z31602953	BrC1=CC=C(S1)C(=O)N1CCNC(=O)C1	289,15	C9H9BrN2O2S
Z228589680	CC1=C(N)C(C(O)=O)=C(F)C=C1	169,16	C8H8FNO2
Z1222423407	CC1=NNC(=N1)C1=CC=C(C=C1)C#N	184,20	C10H8N4
Z1222331426	CC(N)(C(O)=O)C1=C(F)C=CC(F)=C1	201,17	C9H9F2NO2
Z1222331421	CC(N)(C(O)=O)C1=CC(Br)=CC=C1	244,09	C9H10BrNO2
Z31407850	CC(=O)NCC1=CC=CO1	139,15	C7H9NO2
Z1209494589	COCC(C)N1CCN(CC1)C(C)=O	200,28	C10H20N2O2
Z1258955447	CCOC1=C(C)C=C(CO)C=C1C	180,25	C11H16O2
Z1250132782	NC1(CCCCC1)C1=CC=CS1	181,30	C10H15NS
Z1269702473	CC(N1CCC(N)CC1)C1=CC=CC=C1	204,32	C13H20N2
Z1139408804	CC(C)=CCNC1CCC2=NC=NN2C1	206,29	C11H18N4
Z1259087075	CC1=CC2=C(CC(C)(C)CC2N)O1	179,26	C11H17NO
Z1139247397	CNC1CCCN(C1)C1=NN=C(C)C=C1	206,29	C11H18N4
Z2235814827	OCC1(CCC(=O)NC1)C1=CC=CC=C1	205,26	C12H15NO2
Z1255445535	COC1=C(C)N=CC(=C1)C#N	148,17	C8H8N2O
Z361819214	ClC1=CC=C(CNC2=C(C=NN2)C#N)S1	238,69	C9H7ClN4S
Z1509046220	OC(=O)CC1=CC2=C(C=CC=C2)C(=O)N1	203,20	C11H9NO3
Z2236664380		154,21	C8H14N2O
Z2235810124	CC(C)C1=C(SC=N1)C(O)=O	171,21	C7H9NO2S
Z1795826584	COCC1CNCC(C)O1	145,20	C7H15NO2
Z1262254434	CC1=NOC(N)=C1C1=CCCCC1	178,24	C10H14N2O
Z2255110188	COC1=C2C=CC=C(N)C2=CC=N1	174,20	C10H10N2O
Z1262237471	CC(C)OCC1=C(C)C(N)=CC=C1	179,26	C11H17NO
Z85895577	CN(CC(O)=O)C(=O)C1=CC=C(Br)S1	278,12	C8H8BrNO3S
Z1245580492	NCC1=C(C=C(F)C=C1)C(F)(F)F	193,15	C8H7F4N
Z1992316315	CN1CCN(CCN)CC1(C)C	171,29	C9H21N3
Z1962356423	CCC1=C(ON=C1N)C(F)(F)F	180,13	C6H7F3N2O
Z1956875765	CC(C)C1=NN(C)C=C1N	139,20	C7H13N3
Z1318110042	CC#CCN(C)C1CCS(=O)(=O)CC1	215,31	C10H17NO2S
Z168883358	CC(=O)N1CCCNCC1	142,20	C7H14N2O
Z1895554336	CNCC1CCC2=NC=CN2C1	165,24	C9H15N3
Z217102746	NC(C1CC1)C1=CC=CS1	153,24	C8H11NS
Z1649893385	CC1=NOC(=C1C(O)=O)C1=CC=CC=C1	203,20	C11H9NO3

Z1696861512	OC1=NN=C2CCNCC2=C1	151,17	C7H9N3O
Z1696822743	OC(C1CCOCC1)C(O)=O	160,17	C7H12O4
Z1917790067	OCC1=NC(=CS1)C1=NC=CC=C1	192,24	C9H8N2OS
Z1998612790	C1CNC(CN1)C1=CC=NC=C1	163,22	C9H13N3
Z32279730	COCC(=O)N1CCCC(C1)C(N)=O	200,24	C9H16N2O3
Z1741969215	OC(=O)[C@H]1CCCC(=O)N1	143,14	C6H9NO3
Z2017679577	O=C1CC=NN1CC1=CC=CC=C1	174,20	C10H10N2O
Z2010051583	COC(C)C1=NSC(N)=N1	159,21	C5H9N3OS
Z2009850714	NC1=C(C=C2CCCCC2=C1)C(O)=O	191,23	C11H13NO2
Z2009851541	OC(=O)C1=C2NCCC2=CC=C1	163,18	C9H9NO2
Z1642061111	CCC1=NOC(=N1)C1=CC(Cl)=C(N)C=C1	223,66	C10H10ClN3O
Z1625822300	CC(C)CC1CC(CC(=O)N1)C(O)=O	199,25	C10H17NO3
Z1601554962	CC1CC(CCO1)C(O)=O	144,17	C7H12O3
Z1601555025	NC(C(O)=O)C1=CC=C(Cl)C=C1	185,61	C8H8ClNO2
Z227975776	CN(C)C1=NC(C)=C(S1)C(O)=O	186,23	C7H10N2O2S
Z319024806	CC1=C(C)C(C#N)=C(NCC2CC2)N=N1	202,26	C11H14N4
Z1575304440	O=C1CC2=C(N1)C=CC=N2	134,14	C7H6N2O
Z1575143797	CCS(=O)(=O)C1=C(Cl)C=CC(N)=C1	219,68	C8H10ClNO2S
Z1642389924	COC1=C(C=CC=C1N)C(N)=O	166,18	C8H10N2O2
Z1642389727	CCC1=CC(=CC(=O)N1)C(O)=O	167,16	C8H9NO3
Z1699556624	CN1C=C(CNC2=NC(C)=NN2C)C=N1	206,25	C9H14N6
Z228586822	O=C(CC1=CC=CS1)N1CCNCC1	210,30	C10H14N2OS
Z241190530	O=C(CCN1C=NC=N1)N1CCOCC1	210,24	C9H14N4O2
Z1270246351	OC(=O)C1CCCCCC1	142,20	C8H14O2
Z1269702273	NCCN1C=C(C=N1)C#N	136,16	C6H8N4
Z27797391	COC1=C(NC(=O)C(C)C)C=CC=C1	193,25	C11H15NO2
Z1357827195	CC1CCC2=C(C=CC=C2F)C1N	179,24	C11H14FN
Z1160899527	CC1(N)CCC2=C1C=CC=C2	147,22	C10H13N
Z1979756072	CC1CN(CC2=C(C)N(C)N=C2C)C1	193,29	C11H19N3
Z1262252948	NC1=CC=NN1CC1CCOC1	167,21	C8H13N3O
Z927756398	CC1=NC(NC(=O)C(C)(C)N)=CC=C1	193,25	C10H15N3O
Z228589692	CC1=C(C(O)=O)C(N)=C(F)C=C1	169,16	C8H8FNO2
Z1675167140	CN1N=NC2=C1C=C(C=C2)C(O)=O	177,16	C8H7N3O2
Z2235810235	CC1=C(CO)N=CC=C1N	138,17	C7H10N2O
Z2235790653	OC(=O)CC1=CNC(=O)C=C1	153,14	C7H7NO3
Z2235790638	CC1=C(F)C=C(CC(O)=O)C(F)=C1	186,16	C9H8F2O2
Z2235681414	OC(=O)C1=C2CCCN2N=C1	152,15	C7H8N2O2
Z2235681291	CCC1=NC(=O)C(C)=C(N)N1	153,19	C7H11N3O
Z2230911536	O=C1CC2=C(CN1)C=CC=C2	147,18	C9H9NO
Z2230911380	CC1=CC2=C(C=NN2C=C1)C(O)=O	176,18	C9H8N2O2
Z2218555757	CCN1N=NC(C)=C1C(O)=O	155,16	C6H9N3O2
Z2218555806	NCCC1=CN2N=CC(Cl)=C2N=C1	196,64	C8H9ClN4
Z1781322218	BrC1=CC=C(CNC(=O)C2=NNC=C2)O1	270,09	C9H8BrN3O2
Z2574360289	CNC1=C(F)C=C(C=C1)C(O)=O	169,16	C8H8FNO2
Z2568751830	NC(=O)C1CNCCN1C1CC1	169,23	C8H15N3O
Z2568751751	OC(=O)C1=C2CCCCN2C=C1	165,19	C9H11NO2
Z2523459941	CC1=CC=CN2C(N)=CN=C12	147,18	C8H9N3
Z2466639957	COC1=C(C=CC=C1)C1=NC(N)=NC=C1	201,23	C11H11N3O
Z2517219392	CC1(C)CNC(=O)CC(C)(C)C1	169,27	C10H19NO
Z2515399703	O=C1NCC(N1)C1=CC=CC=C1	162,19	C9H10N2O
Z2515207584	FC(F)C1=NC=C(C=C1)C#N	154,12	C7H4F2N2
Z2512942913	OCC1=CC2=C(O1)C=CN=C2	149,15	C8H7NO2
Z2512942927		143,19	C7H13NO2
Z2512942883	CC1=NC(NC2CC2)=CC(=C1)C(O)=O	192,22	C10H12N2O2
Z1245537934	CC(C)C1=CC(=NO1)C(O)=O	155,15	C7H9NO3
Z1924086866	NC(=O)C1=C(CC(O)=O)C=CC=C1	179,18	C9H9NO3
Z57111831	CC1=C(C=NN1C1=CC=CC=C1)C(O)=O	202,21	C11H10N2O2
Z137579644	C(NC1=CC=CC=C1)C1=CSC=N1	190,26	C10H10N2S
Z90666965	CC1=NNC(=O)C(C#N)=C1C	149,15	C7H7N3O
Z94599150	COC1=C(F)C=C(C=C1)C(C)N	169,20	C9H12FNO
Z94601171	NC(CC(O)=O)C1=CC=CC=C1	165,19	C9H11NO2
Z90123329	CC(N)C1=CC2=C(CCCC2)C=C1	175,28	C12H17N
Z31408631	CC1=CC=C(O1)C(=O)NCC1=CC=CO1	205,21	C11H11NO3
Z56887675	CC1=CC(=CC(C)=C1)C(=O)NCC(O)=O	207,23	C11H13NO3
Z56945500	C[C@H](NC(=O)C1=C(C)C=CC=C1)C(O)=O	207,23	C11H13NO3
Z56871698	OC(=O)CC1=NNC(=O)C2=C1C=CC=C2	204,19	C10H8N2O3
Z56912098	NC(=O)N1CCCCCC1	142,20	C7H14N2O

Z56911968	CN1N=C(C=CC1=O)C(O)=O	154,13	C6H6N2O3
Z56907638	CCC(CC)C1=NN=C(N)S1	171,26	C7H13N3S
Z56899123	NC(CC(O)=O)C1=CC(Br)=CC=C1	244,09	C9H10BrNO2
Z56899025	CN1C(=O)CC2=C1C=CC=C2	147,18	C9H9NO
Z56895352	CC1=CC(=O)NC(=N1)N1CCOCC1	195,22	C9H13N3O2
Z56893095	CC(=O)NC1=C(Br)C=CC(OC(F)F)=C1	280,07	C9H8BrF2NO2
Z56887666	OC(=O)CNC(=O)C1CCCCC1	185,22	C9H15NO3
Z56886577	COC1=CC2=C(OC(C(O)=O)=C2C)C=C1	206,20	C11H10O4
Z56858180	CC(=O)NC1=CC(OC(F)F)=C(C)C=C1	215,20	C10H11F2NO2
Z56827662	OC(=O)CNC(=O)C1=CC=C(Br)C=C1	258,07	C9H8BrNO3
Z56824302	NC(CC(O)=O)C1=CC(F)=CC=C1	183,18	C9H10FNO2
Z56798541	C1CCN=C(CC1)NC1=NN=CS1	196,27	C8H12N4S
Z56757309	OC1C2CC3CC(C2)CC1C3	152,24	C10H16O
Z86417440	O=C(NC1=NN=CS1)C1CCC=CC1	209,27	C9H11N3OS
Z281802060	CNC1=NC(OC)=CC=N1	139,16	C6H9N3O
Z1431904023	CS(=O)(=O)C1=CC2=C(OCCN2)C=C1	213,25	C9H11NO3S
Z1428158219	FC(F)(F)C1CCCC(=O)N1	167,13	C6H8F3NO
Z1416282409	NC1=C(N=CN=C1)N1CCCC1	164,21	C8H12N4
Z1407673104	CCC1=NNC(=O)C(C(O)=O)=C1CC	196,21	C9H12N2O3
Z1272495640	OCCC1CCN(C1)C1=NC=C(Br)C=N1	272,15	C10H14BrN3O
Z228589694	CC1=C(N)C(C(O)=O)=C(Cl)C=C1	185,61	C8H8ClNO2
Z1407007540	CC1=C(C)C(=O)NNC1=O	140,14	C6H8N2O2
Z1407007537	COC1=C(OC)C=C(C(O)=O)C(C)=C1	196,20	C10H12O4
Z1275480355	CC1=NSC(NC(=O)C2=CN=CC=C2)=N1	220,25	C9H8N4OS
Z1365534348	CCC1=NOC(=N1)C1=C(N)C=CC(Cl)=C1	223,66	C10H10ClN3O
Z1362754540	C1CNC2=C(N1)C=CN=C2	135,17	C7H9N3
Z1362754516	COC1=COC(CO)=CC1=O	156,14	C7H8O4
Z1359881911	CCC1(C)CCNC(=O)CC1	155,24	C9H17NO
Z1217910533	NCC1CC2=C(O1)C=CC=C2	149,19	C9H11NO
Z1192379004	NCC1=C(C=CC=C1)N1CCNCC1	191,28	C11H17N3
Z1259197166	CC(O)(CN)CN1CCOCC1	174,24	C8H18N2O2
Z1259339799	CC1CCCC(C1)OCC(O)=O	172,22	C9H16O3
Z56957556	CNCC1=C(OC)C=CC(Br)=C1	230,11	C9H12BrNO
Z1252398313	OC1(CNC2=NC=CC=C2)CCOCC1	208,26	C11H16N2O2
Z600584282	CC1=C(CN)C(F)=CC=C1	139,17	C8H10FN
Z1267773566	CC(C)C(N)C1=NC(C)=NO1	155,20	C7H13N3O
Z1267773595	CCC1=NC(=CS1)C(C)N	156,25	C7H12N2S
Z2168515496	COC1=CC2=C(C=CN2)C(F)=C1	165,17	C9H8FNO
Z1891775757	CC(=O)N1CCNCC1(C)C	156,23	C8H16N2O
Z1431002647	CCOC1=C(N)C=CC(Br)=C1	216,08	C8H10BrNO
Z1381271437	CC(O)COC1CCOC1	146,19	C7H14O3
Z2050725052	CC(C)OC1=C(C=CC=N1)C#N	162,19	C9H10N2O
Z1878656559	CC1CC(C)(C)CC1NCC1=NNC=C1	207,32	C12H21N3
Z56347482	CC(N)C1=CC(=CC=C1)C(F)(F)F	189,18	C9H10F3N
Z1878440717	FC1=C(NCC2=NC=CS2)C(Cl)=CC=C1	242,70	C10H8ClFN2S
Z431807512	CC(C)N(C)S(=O)(=O)N1CCNC(=O)C1	235,30	C8H17N3O3S
Z643741552	CC1=CN2N=C(NCC3CC3)SC2=N1	208,28	C9H12N4S
Z391963058	CNC(=O)C1=CC(=NO1)C1=CC=CC=C1	202,21	C11H10N2O2
Z422500504	CNC(=O)C1=C(C)OC(=N1)C1=CC=CS1	222,26	C10H10N2O2S
Z33547160	NC(=O)CC1CCCCC1	141,21	C8H15NO
Z594293916	CCC(C)N(C)C(=O)NC1=NN=CS1	214,29	C8H14N4OS
Z1272684690		191,23	C11H13NO2
Z274554318	CC(CN1N=C(C)C(Br)=C1C)C(O)=O	261,12	C9H13BrN2O2
Z1271843038	CCC1CCNC(=O)CC1	141,21	C8H15NO
Z2680629402	CCC1=NOC(=N1)C1(O)CCNC1	183,21	C8H13N3O2
Z1309300216	CC1=CC2=C(NC=C2CCC(O)=O)C=C1	203,24	C12H13NO2
Z29885101	CC(=O)NCC1COC2=C(O1)C=CC=C2	207,23	C11H13NO3
Z274553704	CN(C)S(=O)(=O)N1N=C(C)C(Cl)=C1C	237,70	C7H12ClN3O2S
Z1717425227	FC1=CCCN(CC2=NNC=C2)C1	181,21	C9H12FN3
Z2001746360	CC1(C)CNC(=O)CN1C(=O)CCC=C	210,28	C11H18N2O2
Z1993937260	CC(O)CS(=O)(=O)C1=C(Cl)C=CC=C1	234,69	C9H11ClO3S
Z57719465	CCN(CC)C(=O)C1=C(N)N=C(C)S1	212,31	C10H16N2OS
Z228576766	CC(C)C(CN)N1CCC(C)CC1	184,33	C11H24N2
Z927400026	CC(C)C(N)C(=O)N1CCOCC1	186,26	C9H18N2O2
Z1259339823	CN1C=C(C=N1)C1CCCN1	151,21	C8H13N3
Z1262396047	CC1=CC(=NO1)C1CCCN1	152,20	C8H12N2O
Z2182115556	CC(C)C1=NC(C)=C(CO)O1	155,20	C8H13NO2

Z2001745807	COCC(=O)N1CC(=O)NCC1(C)C	200,24	C9H16N2O3
Z1891772416	CC1=NOC(C2CC2)=C1C(O)=O	167,16	C8H9NO3
Z2065464277	CCN(C1CCCC1)C1=NC(N)=NC=C1	206,29	C11H18N4
Z1286244967	CC1=NC(=CS1)C(=O)NCC(C)(C)O	214,28	C9H14N2O2S
Z68591039	COC1=CC2=C(OC(C(N)=O)=C2C)C=C1	205,21	C11H11NO3
Z980250586	CN1N=CC=C1NC(=O)C1=CN=CC=C1	202,22	C10H10N4O
Z1796663008	CC1=CSC(=O)N1CC1=NC=C(Cl)C=C1	240,71	C10H9ClN2OS
Z2037273251	CC(C)C1=CC(C(O)=O)=C(C)O1	168,19	C9H12O3
Z1874299552	CC(C)=CCNC1=C(Cl)C(N)=NC=N1	212,68	C9H13ClN4
Z1899186978	CC(=CC1=CC(Cl)=CS1)C1=NC=CN=C1	236,72	C11H9ClN2S
Z2033454399	CCOC1(CCCC1)C(O)=O	158,20	C8H14O3
Z1270502322	CC(O)(CN)C1=CC=CS1	157,23	C7H11NOS
Z1270446342	NCCC(O)C1=CC=CC=C1	151,21	C9H13NO
Z1270399997	N[C@@H](CC(O)=O)C1=C(F)C=CC=C1F	201,17	C9H9F2NO2
Z1270392238	OC(=O)C1=NC=CC(NC2CC2)=C1	178,19	C9H10N2O2
Z1270387220	CC1CC2=C(O1)C(=CC=C2)C(O)=O	178,19	C10H10O3
Z1270358387	COC1=C(SC(C)=C1)C(O)=O	172,20	C7H8O3S
Z1269638567	CS(=O)(=O)C1=C(N)SC=C1	177,24	C5H7NO2S2
Z1269638426	CC(C)C(C(O)=O)C1=CC=CC=C1	178,23	C11H14O2
Z1268152385	CNC1=CC=C(OC2=NC=CC=C2)C=C1	200,24	C12H12N2O
Z1268152298	OC(=O)C1=CN(CC2=CC=CS2)N=N1	209,22	C8H7N3O2S
Z32733538	COCC(=O)NC1=CC(=CC=C1)C#N	190,20	C10H10N2O2
Z1262250857	NC1=NN(CC2=NC=CC=C2)C=N1	175,20	C8H9N5
Z1262246182	CC1=CC(=C(C)C=C1)C1=NC(N)=NN=C1	200,25	C11H12N4
Z2658865738	CCC1CCCC(N)(CC1)C(O)=O	185,27	C10H19NO2
Z2630949151	OCC#CC1=C(F)C(F)=CC=C1	168,14	C9H6F2O
Z2588063768	CN1C=C(C(O)=O)C(=N1)C1=CC=CO1	192,17	C9H8N2O3
Z1741980286	CC1=CC(F)=C(C(O)=O)C(F)=C1	172,13	C8H6F2O2
Z2582143609	OC(CC1=CC=CC=C1)C(O)=O	166,18	C9H10O3
Z2574910555	NC(C(O)=O)C1=C(Cl)C(Cl)=CC=C1	220,05	C8H7Cl2NO2
Z2106599118	CCC1=NOC(=C1)C(O)=O	141,13	C6H7NO3
Z2106594207	CCC(C)N1C=C(Cl)C(=N1)C(O)=O	202,64	C8H11ClN2O2
Z2510259566	CC(C)OC1=C(C=CC=N1)C(O)=O	181,19	C9H11NO3
Z275169746	CCN1C=CC(=N1)C(O)=O	140,14	C6H8N2O2
Z223658280	CCC(N1C=CC=N1)C(O)=O	154,17	C7H10N2O2
Z2510259351		198,17	C10H8F2O2
Z2510259339	CC1=NOC=C1CC(O)=O	141,13	C6H7NO3
Z2106601614	CC1=CC2=CN(CC(O)=O)N=C2N=C1	191,19	C9H9N3O2
Z2412196484	CC1=C(C2CCC(=O)N2)C(C)=NN1	179,22	C9H13N3O
Z2412196603	CCC1=C(C)C(=O)N=CN1	138,17	C7H10N2O
Z1154745738	OC(=O)CN1C=NC=CC1=O	154,13	C6H6N2O3
Z2379802671	CNC1=NC=CC(OC)=C1	138,17	C7H10N2O
Z2379087815	CC1=C2OC=C(C(O)=O)C2=CC=C1	176,17	C10H8O3
Z2372279075	CS(=O)(=O)C1=CC=C(S1)C(O)=O	206,23	C6H6O4S2
Z2350907421	CCN1CCC(=O)NC2(CCCC2)C1=O	210,28	C11H18N2O2
Z1263529630	CS(=O)(=O)C1=C(F)C=C(N)C=C1F	207,19	C7H7F2NO2S
Z1263529612	CC(N)C1=CC(=CC=C1)S(=O)(=O)N(C)C	228,31	C10H16N2O2S
Z1263529583	CC(C)CC1=NN=C(N)O1	141,17	C6H11N3O
Z1213677549	CC1=CC=C(C=C1)C1=NN=C(N)O1	175,19	C9H9N3O
Z86893741	CC(C)C(=O)NC1=NON=C1C	169,18	C7H11N3O2
Z57780905	CC1=C(C#N)C(=O)N(CC=C)C(O)=C1	190,20	C10H10N2O2
Z1262691671	CC1=C(C=CC(=C1)N1C=NC=N1)C(O)=O	203,20	C10H9N3O2
Z1262398517	NC1(CCCCCC1)C(O)=O	157,21	C8H15NO2
Z1262398415	CNC1=NNC(=C1)C1=CC=CC=C1	173,22	C10H11N3
Z1422127285	OC1CN(C1)C1=NC(=CS1)C(F)(F)F	224,20	C7H7F3N2OS
Z2679906885	COC1=NC=C(Br)C(CO)=C1	218,05	C7H8BrNO2
Z1896595977	CNC1=C(F)C(C)=NC(C)=N1	155,18	C7H10FN3
Z2169650265	NC(=O)CC1=C(F)C(Cl)=CC=C1	187,60	C8H7ClFNO
Z138702688	CN(CCO)C1=CC=CC=C1	151,21	C9H13NO
Z1440873590	CC1OCCN(C1C)C(=O)C1=CCCC1	209,29	C12H19NO2
Z1699555218	CN1N=C(C)N=C1NCC1=NC(C)=CS1	223,30	C9H13N5S
Z1169060901	CNC1(CO)CCOCC1	145,20	C7H15NO2
Z1834223506	OC(=O)C1(CC=C)CCC1	140,18	C8H12O2
Z1694636438	CCC1=C(C=C(C)N=N1)C(O)=O	166,18	C8H10N2O2
Z1824497862	CN(C)C1=NC(CN)=CC(C)=N1	166,23	C8H14N4
Z1824497679	CC(N1C=C(C)C(N)=N1)C1=CC=CC=C1	201,27	C12H15N3
Z1823262959	CCNCC1=CC2=C(NC=C2)C=C1	174,25	C11H14N2

Z1703432371	CC(O)C1CCCN(CC2=CSN=N2)C1	227,33	C10H17N3OS
Z1607356994	CNC1=CC(CO)=CC=C1	137,18	C8H11NO
Z1318356911	CNC1=C(OC)N=CC=C1	138,17	C7H10N2O
Z1276751986	C1CC2(CN1)CCOCC2	141,21	C8H15NO
Z1787185107	CNC1=C(OC)C=CC(OC)=C1	167,21	C9H13NO2
Z1787184927	CC1CC(N)CCN1CC1=CC=CC=C1	204,32	C13H20N2
Z1542123042	CC#CCNC(=O)N1CCC2(CCC2)C1	206,29	C12H18N2O
Z2168282741	O=C1CC(CC2=CC=CC=C2)CCN1	189,26	C12H15NO
Z1982493939	CN1C=C2C(CCCC2=N1)C(O)=O	180,21	C9H12N2O2
Z1948024945	FC(F)C1=NN(C(=O)C1)C1=CC=NC=C1	211,17	C9H7F2N3O
Z1889997416	NC(=O)N1CCC2=C1C=C(N)C=C2	177,21	C9H11N3O
Z1868430497	OC(=O)C1=CNC=C1C1CC1	151,17	C8H9NO2
Z1863617849	CN1C=C(C(O)=O)C2=C(C=CC=C2)C1=O	203,20	C11H9NO3
Z1836338798	OC(=O)CC1=CC2=NNC=C2C=C1	176,18	C9H8N2O2
Z1429858214	COC1=CC=C(C=C1)N1C=CC=N1	174,20	C10H10N2O
Z1741785991	OC(=O)CC1=C2CCCCC2=NN1	180,21	C9H12N2O2
Z2087739430	NCC1CCC2=C(C=NN2C1)C(N)=O	194,24	C9H14N4O
Z1198298267	CC1=CSC(=O)N1CC1=CC=C(F)C=C1	223,27	C11H10FNOS
Z1930291176	CC1(C)CN(CC2=CC=CS2)CC1O	211,32	C11H17NOS
Z1602637364	C(NC1CCCOCC1)C1=CC=CS1	211,32	C11H17NOS
Z1440427866	OC1CN(C1)C(=O)C1=C(Br)C=CC=C1F	274,09	C10H9BrFNO2
Z2405262317	CC1CCC(CN)(CN)CC1	156,27	C9H20N2
Z1330142692	CC(=O)N1CCNC(C)(C)C1	156,23	C8H16N2O
Z2092145118	N[C@H]1CC[C@@H](CC1)NCC1=CN=CC=C1	205,31	C12H19N3
Z1891776421	CC(C)OC1=NC=CC(CN)=C1	166,22	C9H14N2O
Z1262396049	CN(C)C1=C2CNCCC2=NC=N1	178,24	C9H14N4
Z763366278	CC(C)N1CCC(CN)C1	142,25	C8H18N2
Z1551463557	CC1=NC(=NC(CO)=C1)N1CCCCC1	207,28	C11H17N3O
Z1536943084	CC(C)N1N=CC(C(N)=O)=C1N	168,20	C7H12N4O
Z1526503457	CCC1=C(C=C(C=C1)S(C)(=O)=O)C(O)=O	228,26	C10H12O4S
Z1520646252	CN1C(=O)OC2=C1C=CC=C2C(O)=O	193,16	C9H7NO4
Z1511502430	CC(CC(O)=O)C1=C(C)ON=C1C	183,21	C9H13NO3
Z1511494443	CN1C=CN=C1C1=NN=C(N)S1	181,22	C6H7N5S
Z1511494808	NC(C(O)=O)C1=C(F)C=CC=C1F	187,15	C8H7F2NO2
Z1495385307	CNC1=CC(NC)=C(Cl)C=C1	170,64	C8H11ClN2
Z1495385214	CC1CNC(=O)CC(C)C1	141,21	C8H15NO
Z56921150	CN1C(=O)CC2=C1C=CC(Br)=C2	226,07	C9H8BrNO
Z952016246	CN1C(=O)N(C)C2=C1C=CC(Br)=C2	241,09	C9H9BrN2O
Z54160747	CNC1=C(Cl)C=C(Cl)C=N1	177,03	C6H6Cl2N2
Z1463349723	CC1=C(C=CC=C1N)S(C)(=O)=O	185,24	C8H11NO2S
Z1454736877	OCC1=CN2C=CN=C2C=C1	148,17	C8H8N2O
Z1272672419	CC1CC(N)C(=O)N1C1=CN(C)N=C1	194,24	C9H14N4O
Z1263811758	CCOC1=C(F)C=C(CN)C=C1	169,20	C9H12FNO
Z1333043417	CC1=NC(=CC=N1)N1CCC(N)CC1	192,27	C10H16N4
Z1431919938	CCC1=NC2=C(S1)C(CCC2)NC	196,31	C10H16N2S
Z105930004	CC(C)N1CCNC(=O)C1	142,20	C7H14N2O
Z1263529744	COC1=C2NCCCC2=C(C)C=C1	177,25	C11H15NO
Z1266823311	COCCN1CCC(N)C1	144,22	C7H16N2O
Z1661371730	COC1CCN(CC1)C1=NC=C(C)S1	212,31	C10H16N2OS
Z228569944	CCN1CCN(CC1)C(C)CN	171,29	C9H21N3
Z287485530	NC(=O)CC1=CSC(=N1)C1=CC=CO1	208,24	C9H8N2O2S
Z1945695969	CC(C)N1N=C(C=C1N)C1=CC=CO1	191,23	C10H13N3O
Z1699554297	CN1N=C(C)N=C1NCC1=CC=C(Br)S1	287,18	C9H11BrN4S
Z1260735442	COC1=C(C=C(Br)C=C1)C(=O)N(C)C	258,12	C10H12BrNO2
Z57815696	CN1C(=O)N(C)C2=C1C=CC=C2	162,19	C9H10N2O
Z1925930040	OC(=O)C1CCCCC(=O)N1	157,17	C7H11NO3
Z1729209079	BrC1=CC=C(CN2C=CN=N2)S1	244,11	C7H6BrN3S
Z1741976870	COC1=C(C=CC(C)=C1)C#N	147,18	C9H9NO
Z1895742026	CC(C)NC1=NC(C)=NO1	141,17	C6H11N3O
Z1889997503	CC1=C(N)C=C(C=C1)N1CCNC1=O	191,23	C10H13N3O
Z1889917114	COC1=CC2=NNC(N)=C2C=C1	163,18	C8H9N3O
Z373773714	CN1C=C(NC(=O)C2=C(C)C=CO2)C=N1	205,22	C10H11N3O2
Z600428546	CNC1=NC=C(C=C1)C(O)=O	152,15	C7H8N2O2
Z600428566	NC1=CC(=CC=C1)C1=NC(=NO1)C1CC1	201,23	C11H11N3O
Z373773750	CN1C=C(NC(=O)C2=CC(Br)=CN2)C=N1	269,10	C9H9BrN4O
Z381114838	CN(C(=O)C1=CC=C(Br)O1)C1=NC=CS1	287,13	C9H7BrN2O2S
Z372501532	CC1=CC(C(=O)NC2=CNN=C2)=C(C)S1	221,28	C10H11N3OS

Z594413662	CC1=C(C=CO1)C(=O)NCC(O)=O	183,16	C8H9NO4
Z594038310	NC1=CN(CC(=O)NC2CC2)N=C1	180,21	C8H12N4O
Z594038204	CNC(=O)CN1C=C(N)C=N1	154,17	C6H10N4O
Z373775848	CN1C=C(NC(=O)C2=C(C)C=CS2)C=N1	221,28	C10H11N3OS
Z317500324	NC(=O)C1=CC(=CC=C1)C(=O)NC1CC1	204,23	C11H12N2O2
Z2186882445	O=S1(=O)NCCC1C1=CC=CC=C1	197,25	C9H11NO2S
Z371827086	CNS(=O)(=O)C1=C(C)C=C(OC)C=C1C	229,29	C10H15NO3S
Z458894644	CCOC1=NC=C(C=C1)C(N)=O	166,18	C8H10N2O2
Z540727674	CCOC1=NC=C(C=C1)C(O)=O	167,16	C8H9NO3
Z212122838	CC1=CSC(CNC(=O)C2CCC2)=N1	210,30	C10H14N2OS
Z372501060	CC1=CC(C(=O)NC2=CNN=C2)=C(C)O1	205,22	C10H11N3O2
Z29672808	CC1=NN=C(NC(=O)C2=CC=NC=C2)S1	220,25	C9H8N4OS
Z45528338	CCNS(=O)(=O)C1=CC=C(C)C=C1	199,27	C9H13NO2S
Z1259155930	FC(F)(F)C1=CC(=CC=C1)C1=NC=CN1	212,18	C10H7F3N2
Z1235963286	OC(=O)CCC1CCNCC1	157,21	C8H15NO2
Z385158968	O=C(NC1CCCCCC1)C1=CSN=N1	225,31	C10H15N3OS
Z190780124	CC(NC(=O)C1=C(O)N=CC=C1)C1CC1	206,25	C11H14N2O2
Z231744492	CC(N1CCC2=C(C1)C=CS2)C(N)=O	210,30	C10H14N2OS
Z1570998378	CC1CC(CCO1)NC1=C(F)C=CC=N1	210,25	C11H15FN2O
Z2067099522	ClC1=C(CN2CCCS2(=O)=O)SC=C1	251,74	C8H10ClNO2S2
Z90502205	CC(C)NCC1=CC=CO1	139,20	C8H13NO
Z1517452179	N#CC1=CN(CC2=CC=CC=C2)C=C1	182,23	C12H10N2
Z1262237386	NCC1CCN(C1)C1=CC(F)=C(F)C=C1	212,24	C11H14F2N2
Z2233922897	CC1C(CCN1C1=NC(C)=NS1)N(C)C	226,34	C10H18N4S
Z2199618152	CC(C)C1=NN(C)C=C1C#N	149,20	C8H11N3
Z31479147	FC1=CC(=CC(F)=C1)C(=O)N1CCCC1	211,21	C11H11F2NO
Z1741973699	COC1=CC(OC)=CN=C1	139,15	C7H9NO2
Z31504642	COCC(=O)NC1=CC=CC=C1	165,19	C9H11NO2
Z1782259387	CNC(C1=NC=CN1)C1=CC=CC=C1	187,25	C11H13N3
Z1954801633	CC1(C)C(C(O)=O)C1(C)C	142,20	C8H14O2
Z2211231747	OC(=O)CC1CCCS(=O)(=O)C1	192,23	C7H12O4S
Z2205958381	CC(C)N1N=NC(C(O)=O)=C1C	169,18	C7H11N3O2
Z90664455	CC(=O)N1CCC(N)CC1	142,20	C7H14N2O
Z2186881894	OC(=O)CCC1=NC=CC=N1	152,15	C7H8N2O2
Z2186882520	OC(=O)C1C(C(O)=O)C1=C	142,11	C6H6O4
Z2034457351	BrC1=C(NC2CCCNC2=O)N=CC=C1	270,13	C10H12BrN3O
Z332370018	CNC(=O)C1=NC2=C(C=CC=C2)C(=O)N1	203,20	C10H9N3O2
Z332722126	CN1C=CC(NC(=O)C2=CSC(Br)=C2)=N1	286,15	C9H8BrN3OS
Z30242049	CCC(=O)NC1=CC=C(C=C1)C(N)=O	192,22	C10H12N2O2
Z228588548	CCS(=O)(=O)N1CCCC(C1)C(O)=O	221,27	C8H15NO4S
Z32414450	OC1=NC=C(C=C1)C(=O)NC1CCCC1	206,25	C11H14N2O2
Z27782626	O=C(CC1=CC=CS1)NC1=CC=CC=C1	217,29	C12H11NOS
Z316170458	OC(=O)CN1N=CC2=C(C=CC=C2)C1=O	204,19	C10H8N2O3
Z228463938	ClC1=C(CN2C=NC(=N2)C#N)C=CC=C1	218,64	C10H7ClN4
Z45527569	CN(C)S(=O)(=O)C1=C(C)C=CC(C)=C1	213,30	C10H15NO2S
Z271099570	[Na+].CC1=NC2=C(C(N)=C(S2)C([O-])=O)C(C)=C1	244,24	C10H9N2NaO2S
Z119916498	CNC1=C(C(=O)NC2CC2)C(C)=NS1	211,28	C9H13N3OS
Z31725198	OCCNC(=O)C1=CC=NC=C1	166,18	C8H10N2O2
Z234895219	COC1=C(OC)C(CN)=CC=C1	167,21	C9H13NO2
Z1653341213	CC1CC1C(=O)N(C)C1=C(Br)C=CC=N1	269,14	C11H13BrN2O
Z1632776942	CC(O)C1CCCN(CC2=CN=CS2)C1	226,34	C11H18N2OS
Z1863667371	CC1(O)CN(C1)C(=O)NC1=NC=CC=C1	207,23	C10H13N3O2
Z1342206622	CS(=O)(=O)CC1=C(CN)C=CC=C1	199,27	C9H13NO2S
Z1295559787	NCC1(CCO)CCCC1	143,23	C8H17NO
Z1250132418	CC1=CC=C(O1)C1CCCCCN1	179,26	C11H17NO
Z1318186459	CC(C)OC1=CC(CO)=CC=C1	166,22	C10H14O2
Z1250132469	NCC1(CC1)C1=C(F)C=CC=C1	165,21	C10H12FN
Z402037718	CN(C)CC1=CSC(=N1)C1=CC=C(C)O1	222,31	C11H14N2OS
Z1891777161	CCN1C=C(C=N1)C1CNCCO1	181,24	C9H15N3O
Z1350606429		173,21	C8H15NO3
Z57611438	CNS(=O)(=O)C1=C(OC)C=CC(C)=C1	215,27	C9H13NO3S
Z1342208354	NC1=NN(CC2=CC=C(C=C2)C#N)C=N1	199,22	C10H9N5
Z1336457504	CN(C)CC1=CC(N)=C(C)C=C1	164,25	C10H16N2
Z1359429544	COC1=C(C=C(Br)C=C1)C(N)CO	246,10	C9H12BrNO2
Z1357774256	CC(C)OC1=C(N=CC=C1)C(O)=O	181,19	C9H11NO3
Z1357774207	CN1CC(=O)NC2=C1N=CC=C2	163,18	C8H9N3O
Z1354602504	O=C1CCN(CCN1)C1CC1	154,21	C8H14N2O

Z1354602501	NCC1CC(=O)NC2=C1C=CC=C2	176,22	C10H12N2O
Z1354432960	COCCN1N=C(C)C(N)=C1C	169,23	C8H15N3O
Z1815155531	O=C1NCCCC11CCCNC1=O	182,22	C9H14N2O2
Z1238956385	CN1C(C)=NN=C1CN1C=C(Cl)C=N1	211,65	C8H10ClN5
Z30847226	CCC(=O)NC1=CC(F)=C(F)C=C1	185,17	C9H9F2NO
Z203976296	NC(=O)C1=CC=C(CN2C=CN=C2)C=C1	201,23	C11H11N3O
Z57301586	CC1=CN2C(S1)=NC=C(C(O)=O)C2=O	210,21	C8H6N2O3S
Z55928828	CC1=C(C)C=C2N=CNC2=C1	146,19	C9H10N2
Z381471538	O=C(NCC1=CC=CS1)C1COCCO1	227,28	C10H13NO3S
Z363109604	CC1=C(C=CC(=N1)C(=O)NCC=C)C#N	201,23	C11H11N3O
Z432066748	CCOC1=CC(=CC=C1)C1=NNC(N)=C1	203,25	C11H13N3O
Z111781372	CN1C=CC=C1C1=NNC(=C1)C(O)=O	191,19	C9H9N3O2
Z18430620	CC(OC1=CC(Cl)=C(F)C=C1)C(N)=O	217,62	C9H9ClFNO2
Z251977366	CC(C)C(C)NC(=O)C1=NNC(=O)C=C1	209,25	C10H15N3O2
Z425484916	CNS(=O)(=O)C1=CC(N)=C(C)C=C1	200,26	C8H12N2O2S
Z228585974	OCC1=C(Br)C=C2OCCOC2=C1	245,07	C9H9BrO3
Z425389776	OCC1=NC(=CS1)C1=CC=CC=C1	191,25	C10H9NOS
Z234895273	NCC1=CN=C(C=C1)N1C=NC=N1	175,20	C8H9N5
Z415727992	CS(=O)(=O)C1=NC=C(N)C=C1	172,20	C6H8N2O2S
Z398556892	CC1=CC=C(O1)C1=NC(C)=C(S1)C(O)=O	223,25	C10H9NO3S
Z398557008	CC(C)S(=O)(=O)C1=C(C=CC=C1)C(O)=O	228,26	C10H12O4S
Z434667014	CCOC1=C(NC(=O)N(C)C)C=CC=N1	209,25	C10H15N3O2
Z857666908	CC(=O)NC1=C(N)C=C(C=C1)C(F)(F)F	218,18	C9H9F3N2O
Z240299288	CN1C=CC(NC(=O)C2=CC(Cl)=CN2)=N1	224,65	C9H9ClN4O
Z491457098	CC1=CSC(CNC(=O)N2CCC2)=N1	211,28	C9H13N3OS
Z283726940	CC1=NSC2=C1C=C(C=N2)C(O)=O	194,21	C8H6N2O2S
Z1868430544	OC(=O)C1=CC2=C(CCC2)N1	151,17	C8H9NO2
Z1868409671	CN1N=NC(C(O)=O)=C1C	141,13	C5H7N3O2
Z1500735155	O=C(NC1CCOC1)C1CCCOC1	199,25	C10H17NO3
Z104341886	CC1=NOC2=C1C=CC=C2	133,15	C8H7NO
Z2522870727	CN1C=C(C(O)=O)C(=N1)C1CCOCC1	210,23	C10H14N2O3
Z1891772659	CN1C=C(C(O)=O)C(=N1)C1CCOC1	196,21	C9H12N2O3
Z381429030	CC(C)NC(=O)N1CCCC(C1)C(O)=O	214,27	C10H18N2O3
Z1597853149	CC(C)NC1CCCCC1O	157,26	C9H19NO
Z2235810242	COC1=C(Cl)C=CC(CC(O)=O)=C1	200,62	C9H9ClO3
Z2234186138	FC1=CC2=C(NC(=N2)C2CCNC2)C=C1	205,24	C11H12FN3
Z2213893306	OC(=O)C12CC(C1)(C2)C1=CC=CC=C1	188,23	C12H12O2
Z1250132459	C1CC(CCO1)N1CCCNCC1	184,28	C10H20N2O
Z2230864881	CC1=CC(C)=C(C=C1)C1(CN)CC1	175,28	C12H17N
Z221429978	NCCC1=CC=C(C=C1)N1C=CC=N1	187,25	C11H13N3
Z1269702310	CC1=NC(=CC=C1)N1CCC(N)CC1	191,28	C11H17N3
Z228588590	NCCN1CCC2=C(C1)C=CC=C2	176,26	C11H16N2
Z1245537816	NCC1=CC(=NC=C1)N1C=CC=N1	174,21	C9H10N4
Z1270210786	NC1=NC(=NC=C1)N1CCCC1	164,21	C8H12N4
Z1430814775	C1CC2=C(CN1)C=NN2C1=CC=CC=C1	199,26	C12H13N3
Z1170135337	NC1=CC(=CC=C1)C1=NOC(=N1)C1CC1	201,23	C11H11N3O
Z803153260	CC1=CNC(=O)N1C1=CC(N)=CC=C1	189,22	C10H11N3O
Z1276745159	OC(=O)C1CC11CCCC2=C1C=CC=C2	202,25	C13H14O2
Z1266933833	CCC1=C2NC=C(CCN)C2=CC=C1	188,27	C12H16N2
Z1263820464	CN1CC(=O)NC2=C(C=CC=C2)C1=O	190,20	C10H10N2O2
Z1946684538	CN1N=CC(C(O)=O)=C1C#N	151,13	C6H5N3O2
Z1820077950	OC(=O)C1CCCC2=CNN=C12	166,18	C8H10N2O2
Z2255682695	CC(C)OCC1CNCCO1	159,23	C8H17NO2
Z2106601922	CC(C)N1C=C(C)C(N)=N1	139,20	C7H13N3
Z26489203	CCC(=O)NC1=C(C=CC=C1)C#N	174,20	C10H10N2O
Z32367961	O=C(NC1CC1)C1CCCC1	153,23	C9H15NO
Z56871585	CC1=NN=C(NC2CCCC2)S1	183,27	C8H13N3S
Z221430028	NC1CCN(CC(N)=O)CC1	157,22	C7H15N3O
Z221429086	OC(=O)C1=CC=C(NC(=O)C2CC2)S1	211,24	C9H9NO3S
Z105004294	CC1=C(NCC(N)=O)C=C(Cl)C=C1	198,65	C9H11ClN2O
Z27073204	CC1=CC=CC(C)=C1NC(=O)C1CCC1	203,29	C13H17NO
Z29414613	CC(C)C(C)NC(=O)C1=C(Br)C=CC=C1	270,17	C12H16BrNO
Z1867069683	CC1=C(SC2=C1C=CC(C)=N2)C(O)=O	207,25	C10H9NO2S
Z1863617803		141,17	C7H11NO2
Z1692557993	CC1=NOC(NCC2=C(Br)C=CS2)=N1	274,14	C8H8BrN3OS
Z1743521947	CN1C=NN=C1NCC1=C(Br)C=CS1	273,15	C8H9BrN4S
Z1738063683	OCC1=CC=C(CN2N=CSC2=O)C=C1	222,26	C10H10N2O2S

Z839988838	CC1=C(CNC2CCCCC2)C(C)=NN1	207,32	C12H21N3
Z1889902453	CNC1=CC=C(C=C1)S(=O)(=O)N(C)C	214,28	C9H14N2O2S
Z1699519010	CN1N=CN=C1NCC1=C(Br)C=CS1	273,15	C8H9BrN4S
Z1699557134	CN1N=C(C)N=C1NCC1=CSC=N1	209,27	C8H11N5S
Z1127203805	O[C@H]1CCN(C1)C(=O)NCC1CCCC1	212,29	C11H20N2O2
Z1222278664	NC(C(O)=O)C1=CC=C(C=C1)C(F)(F)F	219,16	C9H8F3NO2
Z1119739690	OC(CN1C=CN=C1)C1=CC=C(Br)C=C1	267,13	C11H11BrN2O
Z975839228	CN(CC1=CC=CO1)C1=C(N)N=CC=N1	204,23	C10H12N4O
Z1140027744	BrC1=CC=C(S1)C(=O)NC1CCCOC1	290,18	C10H12BrNO2S
Z153345584	CCN1N=C(OC1=O)C1=CC=C(F)C=C1	208,19	C10H9FN2O2
Z221429968	NC1=CN=C(C=C1)N1CCCC1	163,22	C9H13N3
Z404208434	CNC(=O)C1=CC=C(CN2C=CC=N2)O1	205,22	C10H11N3O2
Z2273962468		213,30	C9H15N3OS
Z2273411596	CCN(C1COC1)C1=NC(C)=NS1	199,27	C8H13N3OS
Z1575308473	CN1C=C(C=N1)C(O)CN	141,17	C6H11N3O
Z1245580450	CC1CC(C)CN(CCN)C1	156,27	C9H20N2
Z1245633176	COCC1=CC(CN)=CC=C1	151,21	C9H13NO
Z1245580523	COC1=CC=C(CC2CCCN2)C=C1	191,27	C12H17NO
Z1139253378	CNCC1CCCN1C1=NN=CC=C1	192,27	C10H16N4
Z1575143886	NCC(N)C1=CC=CC=C1	136,20	C8H12N2
Z90122385	OCC1=NC2=C(S1)C=CC=C2	179,24	C9H9NOS
Z134783700	CC(C)N1CCN(CC1)S(C)(=O)=O	206,30	C8H18N2O2S
Z228465586	N#CC1=NN(CC2CCCO2)C=N1	178,20	C8H10N4O
Z1172115923	CC(=O)NCC1=CC=C(S1)C(O)=O	199,22	C8H9NO3S
Z1171979209	CCN1C=C(Cl)C=C1C(O)=O	173,60	C7H8ClNO2
Z85923420	CC(C)CN1N=CC=C1N	139,20	C7H13N3
Z1259339803	CNCC1=C(OC)C(Br)=CC=C1	230,11	C9H12BrNO
Z996001650	CCN(C)C(=O)CC1=CC(Br)=CC=C1	256,14	C11H14BrNO
Z2509346437	NC1=NC=C(CC2CCCNC2)C=C1	191,28	C11H17N3
Z1255497795	CC1=NNC2=C1C=C(C=C2)C(O)=O	176,18	C9H8N2O2
Z2507870736	C1CC1C1=C2NCCCN2N=C1	163,22	C9H13N3
Z2507870657	CC1=C(Br)C=C2N=CC=NC2=N1	224,06	C8H6BrN3
Z2572825303	CCNS(=O)(=O)C1=C(C)C=C(C)C(C)=C1	227,32	C11H17NO2S
Z1267881617	OC(=O)C1=C(Cl)C=C2NC(=O)CC2=C1	211,60	C9H6ClNO3
Z1741967647	CC1=C(F)C(Br)=CC=C1N	204,04	C7H7BrFN
Z57475114	CC1=NC(=CS1)C1=CC=C(C=C1)C(O)=O	219,26	C11H9NO2S
Z57101151	CC1=NC2=C(C=NN2)C(N)=N1	149,16	C6H7N5
Z1981870422	CC1=CC(N)=CC(C)=C1Cl	155,63	C8H10ClN
Z1954856453	CC1C(=O)NC2=C1C=CC=C2	147,18	C9H9NO
Z1945984155	CC(C)C(N)(C(O)=O)C1=CC=CC=C1	193,25	C11H15NO2
Z1992316394	CC1=C(C=C(C=C1)C(O)=O)C#N	161,16	C9H7NO2
Z1398461990	OC1CCC2=C(C1)NN=C2	138,17	C7H10N2O
Z276130164	CC1=C(Br)C=C(N)C(N)=N1	202,06	C6H8BrN3
Z992946910	O=C1NN=C2CCCC2=C1	136,15	C7H8N2O
Z84563384	CCC1=C(C)C=C(S1)C(N)=O	169,24	C8H11NOS
Z135394292	CC(C)NC(=O)C1=CC(C)=NO1	168,20	C8H12N2O2
Z17829253	NC(=O)COC1=C(Br)C=CC=C1	230,06	C8H8BrNO2
Z2692093593	NC1=C(C=CC=C1)C1CCC(=O)N1	176,22	C10H12N2O
Z2677920990	NC(=O)C1CCN(CC2=CC(Br)=CN2)C1	272,15	C10H14BrN3O
Z1259155959	CC1=C(CCO)C(=O)NN1	142,16	C6H10N2O2
Z1741969107	COC1=C(C=CC=C1Br)C(O)=O	231,05	C8H7BrO3
Z1694794850	CN1C=NN=C1NC(=O)C1=C(C)OC=C1	206,21	C9H10N4O2
Z1728624024	CC(=CC1=CN=CS1)C1=NC=CN=C1	203,26	C10H9N3S
Z1728193453	CC1=CC(C)=C(O1)C(=O)N1CC(CO)C1	209,25	C11H15NO3
Z1743520862	CN1C=NN=C1NCC1=CC=C(S1)C#N	219,27	C9H9N5S
Z909942618	NC(=O)CN1CC2CC3CC(C2)CC1C3	208,31	C12H20N2O
Z1834265833	CC1CNC2=C(C=CC=C2)S1=O	181,25	C9H11NOS
Z1694793088	CN1C=NN=C1NC(=O)C1CCC=CC1	206,25	C10H14N4O
Z1824482611	COC1=C(C(O)=O)C(=O)NC=C1	169,14	C7H7NO4
Z1259041047	COC1=C(C=CC=C1)C(C)(C)C(O)=O	194,23	C11H14O3
Z1258943241	CC1(CC1C(O)=O)C1=C(F)C=CC=C1F	212,20	C11H10F2O2
Z1258620048	CC1=NOC2=C1C=C(N)C=N2	149,15	C7H7N3O
Z445914158	CCC(C)NC(=O)N1CCC1	156,23	C8H16N2O
Z1255523297	NC1=CC(CCC2=CC=CC=C2)=NN1	187,25	C11H13N3
Z1250888348	CCOC1=C(N)C(C(O)=O)=C(F)C=C1	199,18	C9H10FNO3
Z927535612	NC1=C(C=C(Cl)C=C1)C(=O)NCC1CC1	224,69	C11H13ClN2O
Z1250132669	CCN1C=C(C(O)=O)C(=N1)C(C)C	182,22	C9H14N2O2

Z1250100774	NCC1=CC(=CC=C1)C1=CC=C(S1)C#N	214,29	C12H10N2S
Z1823256834	CCNC1=CC2=C(NC(=O)N2)C=C1	177,21	C9H11N3O
Z1262237449	CN(C)C1=CN=C(N)C=C1	137,19	C7H11N3
Z1262237268	CC1=C(C(N)=NN1)C1=CC(Cl)=CC=C1	207,66	C10H10ClN3
Z1262237225	OC1(CCOCC1)C1=CC=CC=C1	178,23	C11H14O2
Z1259273388	CN(C)C(=O)CN1C=NC(N)=N1	169,19	C6H11N5O
Z1259273094	NC(C(O)=O)C1=CC(=CC=C1)C(F)(F)F	219,16	C9H8F3NO2
Z1259162157	OC(=O)CCN1CCCC1=O	157,17	C7H11NO3
Z162878494	CCN1C(CNC)=NC2=C1C=CC=C2	189,26	C11H15N3
Z1262327517	CC1=NC(NCC2CCNC2)=CC=C1	191,28	C11H17N3
Z1176190687	BrC1=CN(CCN2CCCC2)N=C1	244,14	C9H14BrN3
Z905065786	CCC1=NC(CCN)=CS1	156,25	C7H12N2S
Z1262237439	CC(C)N(C)C(=O)C1CCCNC1	184,28	C10H20N2O
Z137907758	CN1C=C(CNC2=C(F)C=CC=C2)C=N1	205,24	C11H12FN3
Z1673972503	CS(=O)(=O)NCC1=CN=CS1	192,25	C5H8N2O2S2
Z2687203707	OCC(O)C1=C(Cl)C(Cl)=CC=C1	207,05	C8H8Cl2O2
Z2687203820		151,21	C9H13NO
Z165143678	CC(=O)NC1=C(OC(F)(F)F)C=CC=C1	219,16	C9H8F3NO2
Z2218204415	CC#CCN(C)C1=NC=C(F)C=N1	179,20	C9H10FN3
Z1262515715	CC1=NSC(CC(O)=O)=C1	157,19	C6H7NO2S
Z1171979266	CC1(CCOCC1)C(O)=O	144,17	C7H12O3
Z1171979201	CN(C)C(=O)C1=NC(C)=C(C=C1)C(O)=O	208,22	C10H12N2O3
Z432792294	CN1CCN(CC1)C(=O)C1=C(Br)C=CS1	289,19	C10H13BrN2OS
Z2177030758	FC(F)C1=NC2=C(C=C(F)C=C2)C(=O)N1	214,15	C9H5F3N2O
Z1171978900	CC1=CC(C(O)=O)=C(C)N1CC1CC1	193,25	C11H15NO2
Z1171978887	CC(NC(=O)C1CCC1)C(O)=O	171,20	C8H13NO3
Z373102456	OC(=O)C1CCCN(C1)C1=NC=CS1	212,27	C9H12N2O2S
Z1171978885	OC(=O)CC1=CC2=C(O1)C=CC=C2	176,17	C10H8O3
Z1171978844	CN1C(=O)NC2=C1C=CC(=C2)C(O)=O	192,17	C9H8N2O3
Z1171978786	CN1C=CC=C(C(O)=O)C1=O	153,14	C7H7NO3
Z751623148	CC(N1CCNC(=O)C1)C1=NN=C(C)O1	210,24	C9H14N4O2
Z955198822	OCCN1C(=O)ON=C1C1=CC=CC=C1	206,20	C10H10N2O3
Z355202286	CCC1CN(CCO1)S(=O)(=O)N(C)C	222,30	C8H18N2O3S
Z220816104	CC(=O)N1CCCC(C1)C(N)=O	170,21	C8H14N2O2
Z32399802	CC(=O)N1CCC(CC1)C(N)=O	170,21	C8H14N2O2
Z370745496	CNC(=O)C1=NC=C(C=C1)C(O)=O	180,16	C8H8N2O3
Z1768428780	CN1C=C(C(O)=O)C(=N1)C1=CC=CS1	208,24	C9H8N2O2S
Z1762997351	CC(N)(C(O)=O)C1=C(F)C=CC=C1	183,18	C9H10FNO2
Z458897272	CC1=C(C=CC=C1)C1=NNC(=C1)C(N)=O	201,23	C11H11N3O
Z1737940706	COC1=C(C=CN=C1)C#N	134,14	C7H6N2O
Z1723849112	CC1CC(C(C1)C(O)=O)C(O)=O	172,18	C8H12O4
Z1723549277	COC1=CC(=NN1C)C(O)=O	156,14	C6H8N2O3
Z1723549311	COC1=C(F)C=C(C=C1)C(N)C(O)=O	199,18	C9H10FNO3
Z1602246704	NC(=O)C1=C(C=C(Br)C=C1)C(F)(F)F	268,03	C8H5BrF3NO
Z1618338338	CC1=CC(Br)=C(C)C=C1OCC(N)=O	258,12	C10H12BrNO2
Z90539275	CC1CCC(CC1)NCC1=CSC(C)=N1	224,37	C12H20N2S
Z90713719	CCC1=C(SN=N1)C(=O)N1CCOCC1	227,28	C9H13N3O2S
Z2754910894	Cl.CC(C)C1OCCC1CN	179,69	C8H18ClNO
Z1416282851	CN1CCOC2CNCC12	142,20	C7H14N2O
Z1259339139	NC1=CN=C(NC2CCCC2)C=C1	177,25	C10H15N3
Z927323122	CC(C)C(=O)N1CCCNCC1	170,26	C9H18N2O
Z285973104	CNCC1=NC2=C(C=CC=C2)C(C)=N1	187,25	C11H13N3
Z1359761587	CC(C)N1N=C2CCNCC2=CC1=O	193,25	C10H15N3O
Z1263771201	C1CC(CN1)C1=NC=CC=C1	148,21	C9H12N2
Z2689031550	CN1C=C(C(O)=O)C(=N1)C1=CC=C(C)O1	206,20	C10H10N2O3
Z2146515842	FC(F)C1=C(C#N)C(F)=CC=C1	171,12	C8H4F3N
Z2146515164	CC(C)C1=C(N=CS1)C(O)=O	171,21	C7H9NO2S
Z2146515720	CC1(C)CNCC(C)(C)N1	142,25	C8H18N2
Z2146515522	OC(=O)C1=C(NC=C1)C1CCC1	165,19	C9H11NO2
Z2144245215	CC1(C)CC(O)CC(C)(C)N1	157,26	C9H19NO
Z2143570482	CN1C=C(C=N1)C1NCCNC1=O	180,21	C8H12N4O
Z2130644351	OC(=O)CC1CCCC(=O)N1	157,17	C7H11NO3
Z2122400671	CC1=C(N)C=C(C=C1)C1CNCCN1	191,28	C11H17N3
Z2120060941	FC(F)C1=CC(=O)C2=C(N1)C=CC(Cl)=C2	229,61	C10H6ClF2NO
Z2111526120	OC(=O)C1=C(N=CO1)C1CC1	153,14	C7H7NO3
Z2096088066	OCC1=CN=NN1C1=CC=CC=C1	175,19	C9H9N3O
Z2096088430	CC1=C(C=CC(O)=N1)C#N	134,14	C7H6N2O

Z1803709444	NC(=O)CN1CCC=CC2=C1C=CC=C2	202,26	C12H14N2O
Z2070537331	CC1=NOC(CC(O)=O)=C1	141,13	C6H7NO3
Z2168282733	CC1=C(C)C2=C(NC=N2)C=C1	146,19	C9H10N2
Z2160887345	CCC1=C(C=CN1C)C(O)=O	153,18	C8H11NO2
Z2160887321	COCC1=CC(=O)C2=C(N1)C=CC(F)=C2	207,20	C11H10FNO2
Z1741981838	OC(=O)C1=C(N=NS1)C1CC1	170,19	C6H6N2O2S
Z1258578224	NC(CCO)C1=CC=CS1	157,23	C7H11NOS
Z1501469697	CN1C(C)=CC=C(N)C1=O	138,17	C7H10N2O
Z1337810783	CN(CC1CCCCO1)C1=CN(C)N=C1	209,29	C11H19N3O
Z1428018534	OC1CC(C1)C1=CC=CC=C1	148,21	C10H12O
Z1139545993	CC1=CN(N=C1)C1CN(CC(C)(C)O)C1	209,29	C11H19N3O
Z1741794236	CN(C)C1=NOC(CO)=N1	143,15	C5H9N3O2
Z104346692	CN(C)CC(N)C1=CC=CC=C1	164,25	C10H16N2
Z1172115976	OC(=O)CN1CCCS1(=O)=O	179,19	C5H9NO4S
Z1404661584	CC(N)C1=CC(=CC=C1)N1CCCC1	190,29	C12H18N2
Z227830216	NC1=CN=C(C=C1)N1CCCCC1	177,25	C10H15N3
Z910841110	CC(=O)NC1=C(C=C(C)C=C1Br)C(O)=O	272,10	C10H10BrNO3
Z234897285	CN1C(=O)OC2=C1C=C(C)C=C2	163,18	C9H9NO2
Z30272045	CNC(=O)C1=CC=C(CS(C)(=O)=O)C=C1	227,28	C10H13NO3S
Z234895869	NCC(O)C1CCCCC1	143,23	C8H17NO
Z19648288	NC(=O)CN1C=NC2=C1C=CC=C2	175,19	C9H9N3O
Z969560530	NC(=O)CC1CCCCN1	142,20	C7H14N2O
Z33545772	CS(=O)(=O)C1=CC=C(C=C1)C(N)=O	199,22	C8H9NO3S
Z370277954	CCC1=CC=C(O1)C(=O)N(C)C	167,21	C9H13NO2
Z370278114	COCC1=C(C=CC=C1)C(=O)N(C)C	193,25	C11H15NO2
Z102767808	CNCC1=C(C)ON=C1C	140,19	C7H12N2O
Z52314092	CC(=O)NCCC1=C(C)C=CC=C1	177,25	C11H15NO
Z140637446	COCC(=O)NC1=C(F)C=C(C)C=C1	197,21	C10H12FNO2
Z203974470	COC1=C(CN2C=CN=C2)C=C(C)C=C1	202,26	C12H14N2O
Z138108940	CNCC1=CC(=CC=C1)C#N	146,19	C9H10N2
Z2327226104	CC1=C(C=CC=C1)N1C=C(N=N1)C(O)=O	203,20	C10H9N3O2
Z2327224819	NC(=O)C1(CCCCC1)C1=CC=NC=C1	204,27	C12H16N2O
Z2327224800	CN1N=C(C=C1N)C1=CN=NC=C1	175,20	C8H9N5
Z1526503475	N#CC1=CC(OC2CCC2)=NC=C1	174,20	C10H10N2O
Z2301499792	COC1=CN=C(C=C1)N1CCNCC1	193,25	C10H15N3O
Z2301438391	NC1=C(C#N)C(OC(F)(F)F)=CC=C1	202,14	C8H5F3N2O
Z2301438477	CCN1C=CC=C(N)C1=O	138,17	C7H10N2O
Z2284386121	CC(N)(C(O)=O)C1=CC(Cl)=CC(Cl)=C1	234,08	C9H9Cl2NO2
Z1896597864	CNC1=C(N=CC=N1)C#N	134,14	C6H6N4
Z1267875535	CC(C)C1=NN(C)C2=C1C=C(S2)C(O)=O	224,28	C10H12N2O2S
Z1266933952	OC(=O)CC1CCOCC1	144,17	C7H12O3
Z1020680242	NCC(O)C1=C(Cl)C=C(Cl)C=C1	206,07	C8H9Cl2NO
Z1266854919	CCNC(=O)CN1C=CC(N)=N1	168,20	C7H12N4O
Z1266854886	CCC(O)C1CCCCN1	143,23	C8H17NO
Z927746932	CC(C)(N)C(=O)NC1=CC=CC=C1	178,24	C10H14N2O
Z277110394	NC1=NN=C(CC2CCCC2)S1	183,27	C8H13N3S
Z1263602433	COC1=C(C=CC=C1)C1=NN=C(N)O1	191,19	C9H9N3O2
Z52214433	CC1=C(CC(N)=O)C=CC=C1	149,19	C9H11NO
Z1259161694	OC(=O)C1CC(C1)C1=CC(F)=CC=C1	194,21	C11H11FO2
Z992422476	NC1CCC2=NC=CN2C1	137,19	C7H11N3
Z275728452	CC(N1CCC(C)CC1)C1=NC(C)=NO1	209,29	C11H19N3O
Z2177153697	C1CC1C1=NOC(=N1)C1CCNC1	179,22	C9H13N3O
Z2234631226	OCC1=CC(Br)=C(OC(F)F)C=C1	253,04	C8H7BrF2O2
Z123977486	C(NC1CC1)C1=NC2=C(O1)C=CC=C2	188,23	C11H12N2O
Z172492200	COC1=CC=C(C=C1)C(N)C1CC1	177,25	C11H15NO
Z198249910	CC(C)NC(=O)C1=CC2=C(NN=N2)C=C1	204,23	C10H12N4O
Z2379560414	CC1CN(C1C)C1=NC(C)=NS1	183,27	C8H13N3S
Z1259339810	COC1=CC(=CC=C1)C(O)C(O)=O	182,18	C9H10O4
Z1259087019	OC(=O)C1CC(C1)C1=CC=CC=C1	176,22	C11H12O2
Z2037257441	NCCN1C=CC2=C1C=CC(Cl)=C2	194,66	C10H11ClN2
Z744843458	NC1(CC2=C(C1)C=CC=C2)C(O)=O	177,20	C10H11NO2
Z2026432004	CS(=O)C1=NN=CN1CC1(C)CCC1	213,30	C9H15N3OS
Z1988459579	CN1N=C(C=C1C)C(O)C1=CN=CN=C1	204,23	C10H12N4O
Z2273074160	OC(C1CCC1)C(=O)N1CCC=C(F)C1	213,25	C11H16FNO2
Z1885626052	CC1=CC(=NS1)C(=O)N1CCC=C(F)C1	226,27	C10H11FN2OS
Z1389189457	FC1=C(F)C=C(CC(=O)N2CCC2)C=C1	211,21	C11H11F2NO
Z738792498	COCC(=O)N(C)C1=CC=C(F)C=C1	197,21	C10H12FNO2

Z1644841773	CC1(COC1)C(=O)N1CCC=C(F)C1	199,23	C10H14FNO2
Z2362618963	CN1N=C(C)C=C1CN1CCC=C(F)C1	209,27	C11H16FN3
Z2074076908	FC(F)OCC(=O)N1CCCC2(CC2)C1	219,23	C10H15F2NO2
Z2277255954	CC1(CC1)S(=O)(=O)N1CCC=C(F)C1	219,27	C9H14FNO2S
Z1573313752	CC1=C(NCC2=CSN=N2)C=CC(F)=C1	223,27	C10H10FN3S
Z1613883823	CC1=CC=C(O1)C(=O)N1CCC=C(F)C1	209,22	C11H12FNO2
Z1231644380	CN(C)C(=O)C1(CC1)C1=C(F)C=CC=C1	207,25	C12H14FNO
Z1395924173	NC(=O)CN1CCOC2=C1C=C(F)C=C2	210,21	C10H11FN2O2
Z1337784682	OCCN1CCOC2=C1C=CC(F)=C2	197,21	C10H12FNO2
Z32664952	CC1CCN(CC1)C(=O)CC1=CC=CS1	223,33	C12H17NOS
Z2234631196	NCC1CCCCN1CCO	158,25	C8H18N2O
Z2235330340	CCN1N=C(C)C(CO)=N1	141,17	C6H11N3O
Z1250132377	CC(C)N1C=C(Br)C=C1C(O)=O	232,08	C8H10BrNO2
Z2379087997	OCCC1CCC2=C(N1)C=CC=C2	177,25	C11H15NO
Z2234185629	OC(=O)C1=C2CCCCC2=NO1	167,16	C8H9NO3
Z2236619777	C1CCC2(CC1)COCCN2	155,24	C9H17NO
Z2240988379	COCC1(CCOC1)C(O)=O	160,17	C7H12O4
Z2106594617	CC(C)(N)CN1CCOCC1	158,25	C8H18N2O
Z29693719	O=C(NCC1=CC=CS1)C1=CN=CC=C1	218,27	C11H10N2OS
Z56884346	CC1=C(C)C=C(C=C1)C(=O)NCC(O)=O	207,23	C11H13NO3
Z57040448	OC(=O)CN1NC(=O)C=CC1=O	170,12	C6H6N2O4
Z89283367	CC1=CC2=NC(=CN2C=C1)C(O)=O	176,18	C9H8N2O2
Z85923408	CC1=NN2C=NN=C2NC1=O	151,13	C5H5N5O
Z85921050	OC(=O)[C@@H]1CCCN1C(=O)C1=CC=CO1	209,20	C10H11NO4
Z29672921	CC1=NN=C(NC(=O)C2CCCC2)S1	211,28	C9H13N3OS
Z57069978	OC(=O)CN1N=NC2=C1C=CC=C2	177,16	C8H7N3O2
Z57050098	COC1=CC(C(O)=O)=C(OC)C=C1	182,18	C9H10O4
Z56791867	CCN(CC)C1=CC(C)=NC2=NC=NN12	205,27	C10H15N5
Z53116464	NC(=O)CCN1C=NC2=C1C=CC=C2	189,22	C10H11N3O
Z219094774	N#CC1=CN=C(NCC2=CC=CO2)C=C1	199,21	C11H9N3O
Z815264310	NC1=CC(=CC=C1)N1CCS(=O)(=O)CC1	226,29	C10H14N2O2S
Z18769001	CC(OC1=C(C=CC=C1)C#N)C(N)=O	190,20	C10H10N2O2
Z810541796	NC(CC(O)=O)C1=CC(Cl)=CC=C1	199,63	C9H10ClNO2
Z812517122	CC1=CC(C)=C(C=C1)C1=NN=C(N)O1	189,22	C10H11N3O
Z412132834	CC(C)C1=NN=C(NC(=O)N(C)C)S1	214,29	C8H14N4OS
Z803145534	CCC(=O)NC1=C(N)C=C(OC)C=C1	194,23	C10H14N2O2
Z384359552	ClC1=CC=C(O1)C(=O)NCC1CCOC1	229,66	C10H12ClNO3
Z281113396	CCC1N(CCC2=C1C=CS2)C(=O)NC	224,32	C11H16N2OS
Z19743974	NC(=O)COC1=C(Cl)C=C(Cl)C=C1	220,05	C8H7Cl2NO2
Z802665960	CC1=CC(N)=C(C=C1)S(C)(=O)=O	185,24	C8H11NO2S
Z31791484	CC1=CC=C(S1)C(=O)NC1=CN=CC=C1	218,27	C11H10N2OS
Z235361249	OC(=O)C1CCCC2=C1C=CC=C2	176,22	C11H12O2
Z27381628	CC(=O)NC1=CC(Cl)=C(OC(F)F)C=C1	235,61	C9H8ClF2NO2
Z57472350	CN1CC(CC1=O)C(O)=O	143,14	C6H9NO3
Z361993816	NCCNC1=NC=C(C=C1)C#N	162,20	C8H10N4
Z360010094	OC(=O)CN1C=C(Br)C=CC1=O	232,03	C7H6BrNO3
Z955369596	COC1=C(CNC(C)=O)C=C(Br)C=C1	258,12	C10H12BrNO2
Z394693584	CC1CN(CC(C)O1)S(=O)(=O)CCN	222,30	C8H18N2O3S
Z45498050	O=S(=O)(N1CCCCC1)C1=CC=CS1	231,33	C9H13NO2S2
Z235353833	CC1=NC2=C(O1)C=C(N)C=C2	148,17	C8H8N2O
Z235344767	COC1=NC=CC(=C1)C#N	134,14	C7H6N2O
Z235344617	CN1N=C(C)C2=C1N=C(C)C=C2C(O)=O	205,22	C10H11N3O2
Z27167104	CC(=O)NC1=NN=C(S1)C1CC1	183,23	C7H9N3OS
Z381061488	NCC1(CCOCC1)C(N)=O	158,20	C7H14N2O2
Z33546338	CN1N=C(C(N)=O)C2=C(C=CC=C2)C1=O	203,20	C10H9N3O2
Z30271799	CNC(=O)C1=C(C)C=CC(=C1)S(C)(=O)=O	227,28	C10H13NO3S
Z219104216	CCNC1=NC=C(C=C1)C#N	147,18	C8H9N3
Z367452400	O=S(=O)(N1CCNCC1)C1=CN=CC=C1	227,28	C9H13N3O2S
Z367678526	CC(C)C1=NC2=C(O1)C=CC(N)=C2	176,22	C10H12N2O
Z367452324	CC1=CC2=C(N1)C=CC(N)=C2	146,19	C9H10N2
Z228586424	CC(N)C1=CC2=C(OCC(=O)N2)C=C1	192,22	C10H12N2O2
Z85895273	CN(CC(O)=O)C(=O)C1=CC=CS1	199,22	C8H9NO3S
Z230661148	CC(C)NC(=O)N1CCC2=C(C1)C=CS2	224,32	C11H16N2OS
Z285675390	CC1=C(C)C(C#N)=C(N=N1)N1CCCC1	202,26	C11H14N4
Z2469606466	CN1N=NC2=C1CCC(N)C2	152,20	C7H12N4
Z2467523230	OCC1=C2CCCCN2N=C1	152,20	C8H12N2O
Z2466617838	OC(=O)C1=C2OCCCOC2=CC=C1	194,19	C10H10O4

Z2442047935	O[C@H]1C[C@H](C1)C1=C(F)C=CC=C1	166,20	C10H11FO
Z2440429242	FC(F)(F)OC1=CNC(=O)C=C1	179,10	C6H4F3NO2
Z2418194328	NC1=CN(CCO)C(=O)C=C1	154,17	C7H10N2O2
Z2418194268	OC(=O)C1(CC1)NC1=CC=CC=C1	177,20	C10H11NO2
Z1891775689	CNC1=NNC(=C1)C1=C(OC)C=CC=C1	203,25	C11H13N3O
Z2418194188	O=S1(=O)CC2=C(C1)C=CC=C2	168,21	C8H8O2S
Z2418194280	OC1CCC2=C(NC1=O)C=CC=C2	177,20	C10H11NO2
Z1421484484	CCC(=O)N1CCC(N)C1	142,20	C7H14N2O
Z1263529721	CC(NC1CC1)C1=CN=CC=C1	162,24	C10H14N2
Z1507563282	CC(C)(CN)C1CCCC1	141,26	C9H19N
Z1427941552	CN1N=CC(CCN)=C1C	139,20	C7H13N3
Z1415893948	CCOC1=C(N)C=CC(Cl)=C1	171,62	C8H10ClNO
Z1350579458	CC(C)(N)C1=CC(F)=C(F)C=C1	171,19	C9H11F2N
Z1342206625	CN1CCC(N)(CCO)CC1	158,25	C8H18N2O
Z1262395898	OC1CCCC1CC1=CC=CC=C1	176,26	C12H16O
Z235336569	Cl.CC(=O)NC1=CC=C(CN)C=C1	200,67	C9H13ClN2O
Z53827650	CS(=O)(=O)N1CCCCC1C(O)=O	207,24	C7H13NO4S
Z212045062	O=S(=O)(NC1CCNCC1)C1=CC=CS1	246,34	C9H14N2O2S2
Z210803598	CC1=C(C=C(F)C=C1)C(O)=O	154,14	C8H7FO2
Z55148803	CC1=NN(C(C)=C1)C1=NC(O)=CC(C)=N1	204,23	C10H12N4O
Z235362709	CN1N=C(C)C(C)=C(C(O)=O)C1=O	182,18	C8H10N2O3
Z826553418	NC(=O)CN1C(=O)ON=C1C1=CC=CS1	225,22	C8H7N3O3S
Z906021418	CC(C)C1=NC(C(N)=O)=C(Cl)C=N1	199,64	C8H10ClN3O
Z1250080926	CCNC1=NN=C2CCNCC2=C1	178,24	C9H14N4
Z1139740116	CN1C=NC2=C1CCN(CC=C(C)C)C2	205,31	C12H19N3
Z1222331433	OCC1=CC=C(OC2=CN=CC=C2)C=C1	201,23	C12H11NO2
Z1222331423	CC1=CSC(=N1)N1CCC(CC1)C(O)=O	226,29	C10H14N2O2S
Z1335657489	OCC1CCCN1CC1CCCN1	184,28	C10H20N2O
Z1269702243	CC(N)C1=C(Cl)C(Cl)=CC=C1	190,07	C8H9Cl2N
Z1354432957	COC1=CC(F)=C(C(C)O)C(F)=C1	188,17	C9H10F2O2
Z1160899480	CC1=CN=C(NCC2CCNC2)C=C1	191,28	C11H17N3
Z1259339808	CNC1=NNC(=C1)C1=C(C)C=CC=C1	187,25	C11H13N3
Z1276745040	CCC1=NSC(=N1)N1CCCNCC1	212,32	C9H16N4S
Z1602958158	C[C@@H](O)CN1CCOCC1	145,20	C7H15NO2
Z1741972644	OC(=O)C1=NN2CCCCC2=C1	166,18	C8H10N2O2
Z2158786398	CC1=NN=C(S1)C(=O)N1CCCCN1	212,27	C8H12N4OS
Z1741778448	CN1N=CC=C1C1CC(N)CCO1	181,24	C9H15N3O
Z1592032458	CN1CCC2=C1C=C(N)C=C2	148,21	C9H12N2
Z1272672333	CC(CN)C1=CC(F)=C(F)C=C1	171,19	C9H11F2N
Z2255112965	CNCC1=NC=C(C=C1)C(O)=O	166,18	C8H10N2O2
Z2255110176	CC(C)N1CC2=C(NC1=O)C=CC=C2	190,25	C11H14N2O
Z1741967101	ClC1=C(C=CC=C1)C1CNCCN1	196,68	C10H13ClN2
Z1238949196	FC(F)N1C=CN=C1CN1C=C(Br)C=N1	277,07	C8H7BrF2N4
Z1250089954	CC1=NN=C(O1)C1=CC=C(N)C=C1	175,19	C9H9N3O
Z113296902	CC1=CC(COC2=C(Br)C=CC=C2)=NO1	268,11	C11H10BrNO2
Z1245646830	OC1C(=O)NC2=C1C=C(F)C=C2	167,14	C8H6FNO2
Z1245635827	CCOC1=NC=C(C=C1)C#N	148,17	C8H8N2O
Z104584152	CN(C)C(=O)CNC1=CC=CC=C1	178,24	C10H14N2O
Z104590676	CC1=C(NCC(N)=O)C=CC=C1	164,21	C9H12N2O
Z228585534	CC1CCCN(C1)C(=O)CO	157,21	C8H15NO2
Z1983132682	CN1C=C(C=N1)C(O)C1=CC=CC=C1	188,23	C11H12N2O
Z1925961378	NC1=NNC(=C1)C1CCCOC1	167,21	C8H13N3O
Z1172449162	CCN1N=C(CC)C(CC)=C(C#N)C1=O	205,26	C11H15N3O
Z1966400979	OC(=O)COC1CCCC2=C1C=CC=C2	206,24	C12H14O3
Z1741977827	NC1=C2CCOC2=CC=C1	135,17	C8H9NO
Z1945984179	OC(=O)CC1CCCOC1	144,17	C7H12O3
Z31723394	CCN(CC)C(=O)C1=C(C)C=CC=C1	191,27	C12H17NO
Z1267773582	CC(N)CCN1CCCC1=O	156,23	C8H16N2O
Z104378298	NC(CN1CCOCC1)C1=CC=CC=C1	206,29	C12H18N2O
Z1655104004	CNC1=CN=C(C=C1)N1CCOCC1	193,25	C10H15N3O
Z1649677768	NC1=CC(=NO1)C1CCCO1	154,17	C7H10N2O2
Z1708089972	CC1=CC(=O)C(CO)=NN1	140,14	C6H8N2O2
Z1708089985	NC1=C(OC(F)(F)F)C=CC(=C1)C(O)=O	221,14	C8H6F3NO3
Z1708089983	CC1=CC(=CC(C)=C1)C(N)C(O)=O	179,22	C10H13NO2
Z31182213	NC(=O)C1CCCN(C1)C1=NC=CN=C1	206,25	C10H14N4O
Z1696861484	CC(N)CN1C=NC2=C(C=NN2C)C1=O	207,24	C9H13N5O
Z1696844471	OC(=O)CN1N=C(CC1=O)C(F)(F)F	210,11	C6H5F3N2O3

Z53116498	CC1=NC2=C(C=CC=C2)N1CCC(N)=O	203,25	C11H13N3O
Z1427430205	OC(=O)C1=COC(CN2C=C(Br)C=N2)=C1	271,07	C9H7BrN2O3
Z1696091826	CC(C)C1=C(C(O)=O)C(C)=NN1	168,20	C8H12N2O2
Z1515654336	CN1C=C(C(N)=O)C(=N1)C(F)F	175,14	C6H7F2N3O
Z2004547654	CC1=C2C=CC=CC2=C(N)N=N1	159,19	C9H9N3
Z1998636718	O=C1C=CNC(=N1)N1CCCCC1	179,22	C9H13N3O
Z1993522733	CN(C)C(=O)NC1=CC(N)=C(C)C=C1	193,25	C10H15N3O
Z1993522712	OCC(O)C1=CC(F)=C(Cl)C=C1	190,60	C8H8ClFO2
Z423238660	OCCNC(=O)C1=CC(Br)=CC(F)=C1	262,08	C9H9BrFNO2
Z1992316428	CC1=NSC2=C1C(=O)NC=N2	167,19	C6H5N3OS
Z2028214430	CC1=NC=C2C=CC(=CN12)C(O)=O	176,18	C9H8N2O2
Z54615640	CC1(CCS(=O)(=O)C1)NC(=O)C1CC1	217,28	C9H15NO3S
Z2027049450	CC1(C)CNC2=NC(O)=CC(=O)N2C1	195,22	C9H13N3O2
Z1631503821	CCC(=O)NC1=NC=CC(=C1)C(F)(F)F	218,18	C9H9F3N2O
Z2017843061	CS(=O)(=O)C1=CC(N)=NC=C1	172,20	C6H8N2O2S
Z1791180142	CC(C(=O)NC1=CN=NS1)C1=CC=CS1	239,31	C9H9N3OS2
Z2010009899		190,24	C12H14O2
Z1837073420	CCOC1=C(C=CC(C)=N1)C(O)=O	181,19	C9H11NO3
Z1737000014	CC1=NC(=CO1)C(=O)NCC=C	166,18	C8H10N2O2
Z1416537705	CNC(=O)CC1=NOC(C=C2CCC2)=N1	207,23	C10H13N3O2
Z1642061119	OC(=O)C1=C(C=NC=C1)N1CCCC1	192,22	C10H12N2O2
Z1642061357	CCC1=NN=C(O1)C1=CC(N)=CC=C1	189,22	C10H11N3O
Z445856640	CC(C)NC(=O)N(C)C1CCS(=O)(=O)C1	234,31	C9H18N2O3S
Z1419630209	CC1=NN(CC2CCOC2)C(N)=C1C#N	206,25	C10H14N4O
Z1349163663	CN(C1CCC1)C1=NN=C(C=C1)C(N)=O	206,25	C10H14N4O
Z1575312101	CC1=C(OC=C1)C1=NN=C(N)S1	181,21	C7H7N3OS
Z56767838	CNC1=NC2=C(N1)C=CC=C2	147,18	C8H9N3
Z1082510666	CC1N(CCNC1=O)C(=O)C1=CC=CO1	208,22	C10H12N2O3
Z1569709044	COC1CNC(C1)C(O)=O	145,16	C6H11NO3
Z829561790	CCC(=O)NC1CCCN(C1)S(C)(=O)=O	234,31	C9H18N2O3S
Z741559898	NC(=O)CN1C=C(Br)C=N1	204,03	C5H6BrN3O
Z1642389859	CC1=CN2CCNCC2=N1	137,19	C7H11N3
Z1738061746	CCN1C=CN=C1CN1N=CSC1=O	210,26	C8H10N4OS
Z1632777925	CC(O)C1CCCN(CC2=CC=CO2)C1	209,29	C12H19NO2
Z1667561760	CN1N=C(C)N=C1NC(=O)C1CCCO1	210,24	C9H14N4O2
Z1609052448	COC1=NC(C)=C(NC2CCOC2)C=C1	208,26	C11H16N2O2
Z1241573127	CC1=CSC(CCN2CCCC2=O)=N1	210,30	C10H14N2OS
Z280806554	CCC(=O)N(C)C(C)C1CC1	155,24	C9H17NO
Z1235963386	COC1=C(CN)C=CC=N1	138,17	C7H10N2O
Z48858593	CC1=CSC(=N1)N1CCOCC1	184,26	C8H12N2OS
Z1269702383	NCC1=C(F)C(Cl)=CC=C1	159,59	C7H7ClFN
Z265437038	CCC1=NOC(CN2CCCCC2)=N1	195,27	C10H17N3O
Z729603874	OC(=O)C1CN(C(=O)C1)C1=CN=CC=C1	206,20	C10H10N2O3
Z1683068139	O=C1NN=C2CNCCN12	140,15	C5H8N4O
Z1373975464	CNC(=O)C1=CC2=C(O1)C(Cl)=CC=C2	209,63	C10H8ClNO2
Z2235681444	COC1=NN=C(C=C1)C#N	135,13	C6H5N3O
Z2235681457	CC1=C(C=C(N1)C#N)C(O)=O	150,14	C7H6N2O2
Z2033637875	CN(CC(N)=O)C1=C(C#N)C(C)=NS1	210,26	C8H10N4OS
Z1741972405	OC(=O)C1=CN=C(N=C1)C1CC1	164,16	C8H8N2O2
Z2034495247	CN(C)C1CN(C1)C1=C(C#N)C(C)=NS1	222,31	C10H14N4S
Z1741976372	OC(=O)C1=NN2CCCC2=C1	152,15	C7H8N2O2
Z2235387992	NC(=O)[C@@H]1CCC[C@@H]1NC1CCCC1	196,29	C11H20N2O
Z2218554991	CCN1N=NC=C1C(O)=O	141,13	C5H7N3O2
Z2217033067		157,17	C7H11NO3
Z1419497132	OCCN1C=NC=CC1=O	140,14	C6H8N2O2
Z2522503195	OCC1=CN=C(N=C1)C1=CC=CC=C1	186,21	C11H10N2O
Z1255428434	C1CC(CCN1)C1=CSC=N1	168,26	C8H12N2S
Z2519037609	COC1=C(C=CC=C1)C1=NOC(N)=C1	190,20	C10H10N2O2
Z2519002105	CN1N=CC(C2CC2)=C1C(O)=O	166,18	C8H10N2O2
Z2515207596	CC(C)(CO)N1C=CC=N1	140,19	C7H12N2O
Z2513337808	CN1C=CC(C(O)=O)=C1C1=CC=CC=C1	201,23	C12H11NO2
Z2512942993	OC(=O)C1=C2OCCOC2=CC(Br)=C1	259,06	C9H7BrO4
Z2512942973	COC1=C(C=CC(=C1)C(O)=O)C#N	177,16	C9H7NO3
Z1172115949	CC1=NN(CCO)C=C1C(O)=O	170,17	C7H10N2O3
Z94598730	COC1=C(F)C=C(CC(O)=O)C=C1	184,17	C9H9FO3
Z172492234	CC1=C(C=C(CO)O1)C(O)=O	156,14	C7H8O4
Z188924454	OC(=O)CC1=CC2=C(OCCO2)C(Cl)=C1	228,63	C10H9ClO4

Z45681495	CS(=O)(=O)N1CCCC1C(O)=O	193,22	C6H11NO4S
Z57127432	CC1(C)C(CCC1(C)C(O)=O)C(O)=O	200,23	C10H16O4
Z90664474	COC1=CC(C2CCCN2)=C(OC)C=C1	207,27	C12H17NO2
Z30841040	BrC1=C(C=CC=C1)C(=O)NC1CC1	240,10	C10H10BrNO
Z57101199	CCN(CC)C1=C2C=NN(C)C2=NC=N1	205,27	C10H15N5
Z45527646	CNS(=O)(=O)C1=CC=C(Cl)C=C1	205,66	C7H8ClNO2S
Z56931797	OC(=O)C1CN(CC2=CC=CO2)C(=O)C1	209,20	C10H11NO4
Z56928941	CC1=C(CNC2=CC=CC=C2)C(C)=NO1	202,26	C12H14N2O
Z56921108	CC1=CC(=O)N2N=C(N)SC2=N1	182,20	C6H6N4OS
Z29672791	CC(=O)NC1=NN=C(C)S1	157,19	C5H7N3OS
Z55692907	CCC1=NC2=C(N1)C=CC=C2	146,19	C9H10N2
Z56889385	NC(=O)N1CCCC1C(O)=O	158,16	C6H10N2O3
Z56865289	NC(=O)CN1CCCC1=O	142,16	C6H10N2O2
Z56853704	CNC(=O)C12CC3CC(CC(C3)C1)C2	193,29	C12H19NO
Z56854346	NC(CC(O)=O)C1=C(Cl)C=CC=C1	199,63	C9H10ClNO2
Z234969377	N#CC1=CC(=NC=C1)N1CCOCC1	189,22	C10H11N3O
Z234969369	CC1=C(C=NN1CCO)C(O)=O	170,17	C7H10N2O3
Z56757306	NC(=O)C12CC3CC(CC(C3)C1)C2	179,26	C11H17NO
Z1416282382	OC(=O)CC1=CNC2=C1C=CC=N2	176,18	C9H8N2O2
Z285690798	CC1=NC(C)=NC(=C1)N1CCNC(=O)C1	206,25	C10H14N4O
Z1166191036	CC(C)N1C=C(NC2=NC=CC=N2)C=N1	203,25	C10H13N5
Z1312590836	NC(=O)C1(O)CC2=C(C1)C=CC=C2	177,20	C10H11NO2
Z1365534371	COC1=C(C=C(Br)C=C1)C(N)C(O)=O	260,09	C9H10BrNO3
Z166605294	OCC1=CC2=C(O1)C=CC=C2	148,16	C9H8O2
Z54524377	CC(=C)COC1=CC(=CC=C1)C#N	173,22	C11H11NO
Z1263714204	CC(N)CCC1=CC=C(C)O1	153,23	C9H15NO
Z927412236	CC(N)C(=O)N1CCCCC1C	170,26	C9H18N2O
Z905065808	NCCC1=CC(F)=CC(F)=C1	157,16	C8H9F2N
Z314452408	CCN(C1CC1)C(=O)C1=C(C)C=CO1	193,25	C11H15NO2
Z1162911836	NC1CCCN(C1)C1CCCCC1	182,31	C11H22N2
Z1272684551	NCCC1=NC(=CS1)C(F)(F)F	196,19	C6H7F3N2S
Z204695454	FC1=C(CN2CCCC2)C=CC(=C1)C#N	204,25	C12H13FN2
Z26286176	CC1=NN=C(COC2=C(Br)C=CC=C2)O1	269,10	C10H9BrN2O2
Z1267773609	CCN1CCCCC1CN	142,25	C8H18N2
Z197277614	CC1CCN(CCO)CC1	143,23	C8H17NO
Z1768918374	CN(C1CCC(O)CC1)C(=O)C1CCC1	211,31	C12H21NO2
Z2168515525	OC(=O)C1CCN(C1)C1=C(Cl)C=CC=C1	225,67	C11H12ClNO2
Z1318153868	OCCN1CCC2=C1C=CC=C2	163,22	C10H13NO
Z1583117108	CC1(C)CCCC1NCC1(O)CCOC1	213,32	C12H23NO2
Z2094144722	NCC1CCC2=NC=CN2C1	151,21	C8H13N3
Z1263529726	COC1=C(C=CC=C1)C1CCCN1	177,25	C11H15NO
Z2092396921	CCOC1CC(C1)C(O)=O	144,17	C7H12O3
Z1267882023	CC(C)(CN)C1=CC=CS1	155,26	C8H13NS
Z1891775046	COCC1=C(F)C=CC(CN)=C1	169,20	C9H12FNO
Z2044949055	OCC1=CC2=C(OC=C2)C=C1	148,16	C9H8O2
Z1945710531	COC(C)C1=NC(=CS1)C1=C(C)ON=C1	224,28	C10H12N2O2S
Z1741970163	CC1=C(CCN)N=CC=C1	136,20	C8H12N2
Z285675006	CC1=NC(N)=NC(=C1)N1CCCCCC1	206,29	C11H18N4
Z82283343	O=C(NCC1=CC=CO1)C1CCOCC1	209,25	C11H15NO3
Z105653968	CN(C)C(=O)C1CCN(CC(C)=C)CC1	210,32	C12H22N2O
Z434911400	CN(CC(N)=O)C(=O)C1=CSC(Br)=C1	277,14	C8H9BrN2O2S
Z404922778	CC(N1C=CC=N1)C(=O)N(C)C	167,21	C8H13N3O
Z534296060	CCN1C=NN=C1CNC(=O)N1CCC1	209,25	C9H15N5O
Z729024430	CCN(CC)C(=O)C1=NC(C)=NC=C1	193,25	C10H15N3O
Z119516856	CC(NC(=O)C1=C(C)C=CO1)C1CC1	193,25	C11H15NO2
Z395781192	CC(=O)N1CCCCC1CNS(C)(=O)=O	234,31	C9H18N2O3S
Z756724188	CC1=CSC(CNC(=O)C2=NSN=C2)=N1	240,30	C8H8N4OS2
Z409512732	O=C(NC1=NN=CS1)N1CCCCCC1	226,30	C9H14N4OS
Z131516158	CC(NC(C)=O)C1=NN=C2C=CC=CN12	204,23	C10H12N4O
Z915925122	OC[C@H]1CCCN1C1=NC=C(C=C1)C#N	203,25	C11H13N3O
Z274554552	CC(CN1N=C(C)C(Cl)=C1C)C(O)=O	216,67	C9H13ClN2O2
Z1318356910	CC1=C2C(CCC(O)=O)=CNC2=CC=C1	203,24	C12H13NO2
Z1318268690	CC(N)(C(O)=O)C1=C(Cl)C=CC=C1	199,63	C9H10ClNO2
Z1318147454	COC1=C(Br)C=CC(N)=C1	202,05	C7H8BrNO
Z1313483213	NC1=C(C=CC=C1)C1=NC(=NO1)C1CC1	201,23	C11H11N3O
Z208334664	CN(C)C(=O)NC1=CC(Cl)=CC(Cl)=C1	233,09	C9H10Cl2N2O
Z1213687273	CN1C(=O)COC2=C1C=C(N)C=C2	178,19	C9H10N2O2

Z274553506	CC(CC(O)=O)N1N=C(C)C(C)=C1C	196,25	C10H16N2O2
Z1208912529	CNC(=O)CN1CCCC(CC1)C(C)C	212,34	C12H24N2O
Z818727262	C(NC1CC1)C1CCOC1	141,21	C8H15NO
Z1609161188	CCN1C=C(NC2CCCC2C)C=N1	193,29	C11H19N3
Z1263529780	COC1=C(CN)C(C)=CC(C)=N1	166,22	C9H14N2O
Z1162448860	CN(C)C1=NC=CC(CN)=C1	151,21	C8H13N3
Z1190318575	CC(C)OC1=CC(CN)=CC=C1	165,24	C10H15NO
Z223353618	CN1N=C(C)C=C1NC(=O)C1CCCO1	209,25	C10H15N3O2
Z265541128	CCC1=NOC(CN2CCCC(O)C2)=N1	211,27	C10H17N3O2
Z389867078	NCC1=CC(=CC=C1)C(=O)N1CCCC1	204,27	C12H16N2O
Z2574918328	CN1N=CC=C1CC1CCNCC1	179,27	C10H17N3
Z1416282827	CNC1CCN(CC1)C1=NN=CC=C1	192,27	C10H16N4
Z2065788426	CC1=C(N)C=C(C(O)=O)C(Br)=C1	230,06	C8H8BrNO2
Z2065788347	COC1=C(CO)C(C)=CC(C)=N1	167,21	C9H13NO2
Z2050687995	CCC1=C(C=CN1)C(O)=O	139,15	C7H9NO2
Z2050688026	ClC1=C2CNC(=O)C2=CC=C1	167,59	C8H6ClNO
Z2045034941	NC1=CN=C(CC2CC2)N=C1	149,20	C8H11N3
Z2037273275	CC(CN1C=CC2=C1C=CC=C2)C(O)=O	203,24	C12H13NO2
Z1827603335	O=C(NC1=CN=NS1)C1CCCCCC1	225,31	C10H15N3OS
Z2034364269	CNC1=NC=C(OC)C=C1	138,17	C7H10N2O
Z1270446309	CC(C)N1C(=O)CNCC1(C)C	170,26	C9H18N2O
Z1218252078	OCC1CCCN1CC(O)=O	159,19	C7H13NO3
Z1270384361	COC1=C(C=CC(Br)=C1)C(O)=O	231,05	C8H7BrO3
Z274535144	CC(C)(OC1=CC(Cl)=CC=C1)C(O)=O	214,65	C10H11ClO3
Z1270326281	CCOC1=C(Cl)C=C(C=N1)C(O)=O	201,61	C8H8ClNO3
Z31725404	OCCNC(=O)C1=CC(Br)=CN=C1	245,08	C8H9BrN2O2
Z57229047	CC(NC(=O)C1=CC=C(Cl)C=C1)C(O)=O	227,64	C10H10ClNO3
Z57229045	CC(NC(=O)C1=CC=C(Br)C=C1)C(O)=O	272,10	C10H10BrNO3
Z1269702266	CS(=O)CC1=CC(N)=C(Cl)C=C1	203,68	C8H10ClNOS
Z1268703711	CC1=NN=C(O1)C1=CC(N)=CC=C1	175,19	C9H9N3O
Z1267881751	CC1=C(N)C=CC(=C1)S(C)(=O)=O	185,24	C8H11NO2S
Z1267881746	NC1(CO)CC2=C(C1)C=CC=C2	163,22	C10H13NO
Z1262250750	C1CC(CN1)NC1=CC=CC=C1	162,24	C10H14N2
Z1262246117	CC1=NC2=C(S1)C(O)CCC2	169,24	C8H11NOS
Z1262246115	CC(O)CS(=O)(=O)C1=CC=C(C)C=C1	214,28	C10H14O3S
Z2658865272	COC1=C(OC)N=CC(N)=C1	154,17	C7H10N2O2
Z2597964385	NC(CO)C1=CC=CC=C1	137,18	C8H11NO
Z2588063658	OC(=O)C1CN(C1)C1CCOC1	171,20	C8H13NO3
Z2446040567	CC(C)C1=NC=CN1CC(O)=O	168,20	C8H12N2O2
Z2583036300	O=C1NCCCN1C1=CC=C(C=C1)C#N	201,23	C11H11N3O
Z270979606	CC(C)(C(N)=O)C1=CC=CC=C1	163,22	C10H13NO
Z2106594221	CC(C)N1N=CC(Cl)=C1C(O)=O	188,61	C7H9ClN2O2
Z1431005396	CC(C)C(N1C=C(C)C=N1)C(O)=O	182,22	C9H14N2O2
Z2106598636	CCC(N1N=CC=C1C)C(O)=O	168,20	C8H12N2O2
Z2510944731	CC1=C(C=CC(Cl)=C1)C1(CC1)C(O)=O	210,66	C11H11ClO2
Z1741958104	COC1=C(F)C=CC(=C1)C(O)=O	170,14	C8H7FO3
Z2106594216	CCC(C)N1N=CC=C1C(O)=O	168,20	C8H12N2O2
Z2510259379	CC(C)N1C=C(N=N1)C(O)=O	155,16	C6H9N3O2
Z2235389495		166,18	C8H10N2O2
Z221603924	CC(=O)NC1=C(C=CC=C1C)C(O)=O	193,20	C10H11NO3
Z2510259343	CC1=NN=C2C=CC=C(N12)C(O)=O	177,16	C8H7N3O2
Z431537238	CC(CC(O)=O)N1C=CC=N1	154,17	C7H10N2O2
Z2510259511	OC(=O)CC1CS(=O)(=O)C1	164,18	C5H8O4S
Z2510259303	OC(=O)C1=CN(CC2CC2)N=N1	167,17	C7H9N3O2
Z1741960937	CC1=C(C=CC=C1Cl)C(O)=O	170,59	C8H7ClO2
Z2510258250	COC1=C(C=CC(CO)=C1)C(O)=O	182,18	C9H10O4
Z2510258302	COC1=C(C=CC(=C1)C(F)(F)F)C(O)=O	220,15	C9H7F3O3
Z1741978912	NC1CCCC(C1)C(O)=O	143,19	C7H13NO2
Z2379802567	FC(F)(F)C1=CC(=O)NN1	152,08	C4H3F3N2O
Z2379087723	OCC1=C2CCCN2N=C1	138,17	C7H10N2O
Z1741980032	CC1=C2C=CNC(=O)C2=CC=C1	159,19	C10H9NO
Z2372279593	OC(C1CCCCC1)C1=NC=CN1	180,25	C10H16N2O
Z2372279480	CC1=CC(C(O)=O)=C(C=C1)C1CC1	176,22	C11H12O2
Z235344899	N#CC1=CC(=NC=C1)N1CCNCC1	188,23	C10H12N4
Z2327883779	CN(C(C)=O)C1=NC=C(N)C=C1	165,20	C8H11N3O
Z2327531729	CC(C)CC1=CNC(=C1)C(O)=O	167,21	C9H13NO2
Z1213674920	CCOC1=CC=C(C=C1)C1=NON=C1N	205,22	C10H11N3O2

Z1151517069	CC1=CC(=CC(C)=C1OC(F)F)C(N)=O	215,20	C10H11F2NO2
Z1262398505	NCC(O)C1=CC=CC=C1	137,18	C8H11NO
Z2680629340	OC(=O)C1CCC2=C(CC1)C=CC=C2	190,24	C12H14O2
Z2680629316	CC(=O)N1CC(C1)OCC(O)=O	173,17	C7H11NO4
Z2679906771	FC(F)(F)OC1=CC(=O)NC=C1	179,10	C6H4F3NO2
Z2678251571	CC1=C(N)C(=O)N2C=CC=CC2=N1	175,19	C9H9N3O
Z2678251327	CC1=NN(C2CCOCC2)C(=C1)C(O)=O	210,23	C10H14N2O3
Z2283225344	CCC1(CC1)NC(=O)C1=CC(F)=CN1	196,23	C10H13FN2O
Z1262327411	CNC(=O)CN1C=CC2=C1C=C(N)C=C2	203,25	C11H13N3O
Z198194364	CC1CCCN(C1)C(N)=O	142,20	C7H14N2O
Z1889909829	CC1COCCC1C(O)=O	144,17	C7H12O3
Z1889709964	CC1=CC=C(O1)C1=NNC(N)=C1	163,18	C8H9N3O
Z1699554788	CN1N=C(C)N=C1NCC1=C(Br)C=CS1	287,18	C9H11BrN4S
Z1705151001	CN(C)CC(=O)N1CCCC1C(C)(C)O	214,31	C11H22N2O2
Z1681936395	CN(CC1=NNC=C1)C1=CC(C)=CC=C1	201,27	C12H15N3
Z1787627869	CN(CC1CCOC1)C1=C(Cl)C=NC=N1	227,69	C10H14ClN3O
Z1407659698	CC1CN(CC1N)C1CC1	140,23	C8H16N2
Z1416200832	COC1=NC(C)=C(N)C=C1	138,17	C7H10N2O
Z1259161721	OCCOC1=CN=CC=C1	139,15	C7H9NO2
Z2156626388	O[C@H]1C[C@H](C1)C1=CC=CC=C1	148,21	C10H12O
Z221429096	NCC1=CN=C(C=C1)N1C=CC=N1	174,21	C9H10N4
Z2327226367	COC1=CC2=C(OCC[C@H]2O)C=C1	180,20	C10H12O3
Z2177030625	CC(C)C1(CNC(=O)C1)C(O)=O	171,20	C8H13NO3
Z2027049523	CC1=C(N=NN1)C1=CC=C(Cl)C=C1	193,63	C9H8ClN3
Z1945702023	NC1=CN2C(C=C1)=NN=C2C(F)F	184,15	C7H6F2N4
Z1495385242	C1CC1N1C=NC2=C1C=CN=C2	159,19	C9H9N3
Z1717424191	CN1C=C(CN2CCC=C(F)C2)C(C)=N1	209,27	C11H16FN3
Z1220672383	NC(=O)C1CCCN1CC1=CC(Br)=CS1	289,19	C10H13BrN2OS
Z1624945762	CC(=CC1=CSC(Br)=C1)C1=NC=CN=C1	281,17	C11H9BrN2S
Z2012924471	NC(=O)C1=CC(OC(F)(F)F)=C(Br)C=C1	284,03	C8H5BrF3NO2
Z1455023936	OC(=O)CNC(=O)C1=CC(Br)=CS1	264,09	C7H6BrNO3S
Z2293643386	CC(=O)N1CCCC(O)(C1)C(O)=O	187,20	C8H13NO4
Z55997155	CC(C)NCC(=O)N1CCCCCC1	198,31	C11H22N2O
Z2680304681	CS(=O)(=O)NCC1=NNC=C1	175,21	C5H9N3O2S
Z1558209795	CS(=O)(=O)C1=CC(=CS1)C(O)=O	206,23	C6H6O4S2
Z1520645392	OC(=O)C1=CSC(=N1)C1CCCO1	199,22	C8H9NO3S
Z1516115797	CN1C=C(C(N)=O)C(N)=N1	140,15	C5H8N4O
Z1414093197	COC1=C(C)C=CC(=C1)C#N	147,18	C9H9NO
Z1511483984	COC1=C(OC)C=C(C(O)=O)C(Cl)=C1	216,62	C9H9ClO4
Z1507502325	OC(=O)C(C1CC1)C1CC1	140,18	C8H12O2
Z196118774	OCCNC1=C2SC=CC2=NC=N1	195,24	C8H9N3OS
Z2442033986	NCC1=C(C=CC=C1F)N1CCCC1	194,25	C11H15FN2
Z2442033938	NC1CCN(CC1)C1=C(F)C=CC=C1	194,25	C11H15FN2
Z285718998	CC1=C(C)C(C#N)=C(N=N1)N1CCC1	188,23	C10H12N4
Z30904272	CC1=C(NC(=O)C2CCC2)C=C(F)C=C1	207,25	C12H14FNO
Z32016173	CNC(=O)CNC(=O)C1=CC(Br)=CC=C1	271,11	C10H11BrN2O2
Z30271975	CNC(=O)C1=CC2=C(S1)C=CC=C2F	209,24	C10H8FNOS
Z241832786	CN1CCN(CC1)C(=O)C1CCCN1	197,28	C10H19N3O
Z1258578137	CC(C(O)=O)C1=CC=C(C)O1	154,17	C8H10O3
Z1245633158	COC1=CC(CCN)=CC=C1	151,21	C9H13NO
Z1245633104	NCC1=CN(CC2=CC=CC=C2)N=C1	187,25	C11H13N3
Z1235963376	CC(N)CC1=CC=C(C)S1	155,26	C8H13NS
Z33546668	COC1=C(OC)C(=CC=C1)C(N)=O	181,19	C9H11NO3
Z1945700452	COCCC1=NSC(N)=N1	159,21	C5H9N3OS
Z1729206778	COC1=C(F)C=C(CN2C=CN=N2)C=C1	207,21	C10H10FN3O
Z1272517105	CC1=NSC(NC2CCCC2(C)CO)=N1	227,33	C10H17N3OS
Z1925930638	CC1=C2NC(=O)NC2=NC=C1	149,15	C7H7N3O
Z1925930237	OC(=O)C1=NN(C=C1)C1CCCCC1	194,23	C10H14N2O2
Z1741978268	OC(=O)CC1=CC(F)=C(Cl)C=C1	188,58	C8H6ClFO2
Z1729210493	BrC1=CC(CN2C=CN=N2)=CS1	244,11	C7H6BrN3S
Z54572643	OCC1CCCCN1C1=NC=CN=C1	193,25	C10H15N3O
Z1699556994	CN1N=C(C)N=C1NCC1=C(C)C=CS1	222,31	C10H14N4S
Z1343696013	CNC(=O)C1=C(OC)C=C(Cl)C(Cl)=C1	234,08	C9H9Cl2NO2
Z1895583063	CN(C)C1=NC=NC(N)=C1	138,17	C6H10N4
Z1889917335	CC1=C(Br)C(C(O)=O)=C(F)C=C1	233,04	C8H6BrFO2
Z85886894	CC1=CC(C(=O)NCC(O)=O)=C(C)O1	197,19	C9H11NO4
Z85890614	CC(NC(=O)C1=CC=C(Br)S1)C(O)=O	278,12	C8H8BrNO3S

Z600428548	OC(=O)C1CCCN1C1CCNCC1	198,27	C10H18N2O2
Z237880160	CNC(=O)C1=C(OC)C(Br)=CC(OC)=C1	274,11	C10H12BrNO3
Z373774388	CN1C=C(NC(=O)C2=C(C)OC=C2)C=N1	205,22	C10H11N3O2
Z135384154	CC1=NOC(=C1)C(=O)NCC1=CC=CO1	206,20	C10H10N2O3
Z86949260	CC1=CC=C(O1)C(=O)NC1=NOC(C)=C1	206,20	C10H10N2O3
Z225339168	CC1=CC(NC(=O)C2=CC=C(Cl)O2)=NO1	226,62	C9H7ClN2O3
Z1255523423	NC1=NC2=C(C=CC=C2)N1C1CCCC1	201,27	C12H15N3
Z1222283871	C1CNCC(C1)N1C=NC2=C1C=CC=C2	201,27	C12H15N3
Z2350941316	CN1N=C(CC(O)=O)C2=C1C=CC=C2	190,20	C10H10N2O2
Z415252234	CC(N1C=CN=C1)C(=O)NC1=NN=CS1	223,25	C8H9N5OS
Z422396686	CC1CCCCN1C(=O)C1=C(C)N=CS1	224,32	C11H16N2OS
Z68248055	CN(C)C(=O)C1=C(OC(F)F)C=CC=C1	215,20	C10H11F2NO2
Z1318161309	CC1CN(CCO)CCO1	145,20	C7H15NO2
Z280757432	CC(C)N(C)C(=O)C1=C(C)N(C)C(C)=C1	208,31	C12H20N2O
Z1511499407	OCCN1CCC(CC1)C1=NC=CC=C1	206,29	C12H18N2O
Z802671662	NCCNC1=NC=CN=C1	138,17	C6H10N4
Z2582143817	FC(F)(F)C1=NC(=CO1)C#N	162,07	C5HF3N2O
Z1324255779	CS(=O)(=O)NCC1=NOC=C1	176,19	C5H8N2O3S
Z68639747	CCN1C(NC(C)=O)=NC2=C1C=CC=C2	203,25	C11H13N3O
Z2186895169	CC1=C(Cl)C=CC(CCN)=C1	169,65	C9H12ClN
Z2011901248	BrC1=C(CNC(=O)C2CC=CC2)C=CS1	286,19	C11H12BrNOS
Z2216303045	CCN1N=C(C)C(=N1)C(O)=O	155,16	C6H9N3O2
Z2216303131	CN1C=NC=C1C(O)C1=CN=C(C)C=C1	203,25	C11H13N3O
Z2213893339	CN1C=CC(C(O)=O)=C1C1CCC1	179,22	C10H13NO2
Z2213893326	COC1=C(Cl)C=CC(C(O)=O)=C1N	201,61	C8H8ClNO3
Z2205958928	CC1CC(O)C2=NC=CN12	138,17	C7H10N2O
Z2197092525	CC1=C2C(O)CCOC2=CC=C1	164,20	C10H12O2
Z2197092181	C[C@H]1NCC[C@H]1N(C)CCO	158,25	C8H18N2O
Z2186882337	CN1N=CC=C1[C@@H]1NC(=O)CC[C@H]1N	194,24	C9H14N4O
Z2177030724	CN1C=C(N)C(=N1)C1CC1	137,19	C7H11N3
Z2168564218	CNC1=NOC2=C1C=C(Cl)C=C2	182,61	C8H7ClN2O
Z2168541790	CC1=C(C=CC=C1N)N1C=NNC1=O	190,21	C9H10N4O
Z2168390419	COC1=C(N=C(C)C=C1)C(O)=O	167,16	C8H9NO3
Z2168282687	CN(C)C1=NC=CC(N)=C1	137,19	C7H11N3
Z89404895	CC1=C(C=CO1)C(=O)NCC1=CC=CS1	221,27	C11H11NO2S
Z235362323	CCC1=NC(C)=C(C#N)C(=O)N1	163,18	C8H9N3O
Z68162877	OCCNC(=O)C1=CC2=C(CCCC2)S1	225,31	C11H15NO2S
Z32024416	CCNC(=O)C1=CC(=CC=C1)C(F)(F)F	217,19	C10H10F3NO
Z271004940	NC(CC(O)=O)C1=CC=CS1	171,21	C7H9NO2S
Z237578888	C1CCN(C1)C1=C2C=NNC2=NC=N1	189,22	C9H11N5
Z33545777	CS(=O)(=O)CC1=CC=C(C=C1)C(N)=O	213,25	C9H11NO3S
Z234897455	OC(=O)C1=CC(F)=C(C=C1)N1CCCC1	209,22	C11H12FNO2
Z432788010	BrC1=C(SC=C1)C(=O)NCC1=CC=CO1	286,14	C10H8BrNO2S
Z267487136	CC1=NOC(CN2C=C(Br)C=CC2=O)=N1	270,09	C9H8BrN3O2
Z1700082131	CC1=NN(CC2=NC=C(Cl)C=C2)C(C)=C1	221,69	C11H12ClN3
Z231745152	CN(C)C(=O)CN1CCC2=C(C1)C=CS2	224,32	C11H16N2OS
Z32384710	CC(C)CNC(=O)C1=NN(C)C(=O)C=C1	209,25	C10H15N3O2
Z1262396038	COC1=C(N)C=CC(F)=C1	141,15	C7H8FNO
Z1664359523	COC1=C(N)C=C(F)C=C1	141,15	C7H8FNO
Z1218186922	CC1=CC=C(O1)C1CCCN1S(C)(=O)=O	229,29	C10H15NO3S
Z1267882033	CCN1C=C(CNC)C=N1	139,20	C7H13N3
Z1335657447	COC1=CC(=CC=C1)C(O)C1CC1	178,23	C11H14O2
Z1276496841	CC1CCNC(C1)C1=CC=C(C)O1	179,26	C11H17NO
Z415735030	CC1=CC(Br)=CC(C)=C1CN	214,11	C9H12BrN
Z1262395953	CC1=C(C=CC=C1)C1(CN)CC1	161,25	C11H15N
Z1259339816	OC(=O)CC1=CCCCC1	140,18	C8H12O2
Z1324054923	CC(N)(C)C1=CC=CC=C1	149,24	C10H15N
Z275170058	CCN1N=C(C)C(CN)=C1C	153,23	C8H15N3
Z1262237455	CC1=CN=C(S1)C(N)C1CC1	168,26	C8H12N2S
Z103706784	CNC(=O)CN(C)C1CCS(=O)(=O)C1	220,29	C8H16N2O3S
Z1263828503	CC1CN(CC2=CN=C(C)S2)CCC1N	225,35	C11H19N3S
Z1295619820	CC1CNC(CO1)C1=CC=CC=C1	177,25	C11H15NO
Z57269988	CNS(=O)(=O)C1=CC(C)=C(OC)C=C1	215,27	C9H13NO3S
Z1347206062	CNS(=O)(=O)C1=C(OC)C=C(C)C(Cl)=C1	249,71	C9H12ClNO3S
Z45528324	CCNS(=O)(=O)C1=CC=C(Cl)C=C1	219,68	C8H10ClNO2S
Z1347206044	CNS(=O)(=O)C1=C(C)C=C(C)C(C)=C1	213,30	C10H15NO2S
Z1347206038	CNS(=O)(=O)C1=C(C)C=C(C)C(OC)=C1	229,29	C10H15NO3S

Z1286244717	CC(C)(O)CNC(=O)C1=NC=C(Cl)C=C1	228,68	C10H13ClN2O2
Z1342208419	CC(O)C1=NC2=C(C=CC(Cl)=C2)N1C	210,66	C10H11ClN2O
Z1342208411	C1COC2=C(N1)C=CC=N2	136,15	C7H8N2O
Z1333717633	CC(C)N1CCC(N)C1=O	142,20	C7H14N2O
Z31767568	CC1CC(C)CN(C1)C(=O)C1CCC1	195,31	C12H21NO
Z133629916	CS(=O)(=O)NC1CCC(O)CC1	193,26	C7H15NO3S
Z1357774184	CNC1=CC2=C(C=C1)N=C(C)S2	178,25	C9H10N2S
Z1815157369	COC1=C(C(O)=O)C(N)=C(F)C=C1	185,15	C8H8FNO3
Z1815157352	CC1=NC(=CS1)C1=CNC=C1C(O)=O	208,24	C9H8N2O2S
Z992717294	COC1=CC(Br)=C(C=C1)C(O)=O	231,05	C8H7BrO3
Z1815157424	CCOC1=C(N=C(C)C=C1)C(O)=O	181,19	C9H11NO3
Z1815156880	CS(=O)(=O)C1=C(C(O)=O)C(Cl)=CC=C1	234,65	C8H7ClO4S
Z1741980868	COC1=C(OC)C(C(O)=O)=C(F)C=C1	200,17	C9H9FO4
Z1068186288	CNC(=O)C1=NC=C(Br)C=C1	215,05	C7H7BrN2O
Z183864280	CCNC(=O)C1=CC(Br)=CN1	217,07	C7H9BrN2O
Z1653341133	CN(C(C)=O)C1=C(Br)C=CC=N1	229,08	C8H9BrN2O
Z1809367755	CC(C)CCC1=NNC(N)=C1	153,23	C8H15N3
Z1809367538	OC(CC1=CC(Cl)=CC=C1)C(O)=O	200,62	C9H9ClO3
Z1262398472	CC1=CC(=C(C)O1)C1=NN=C(N)O1	179,18	C8H9N3O2
Z1801307715	BrC1=CC2=C(OCCN2)C=C1	214,06	C8H8BrNO
Z1680940545	CC1CC(C)(C)CC(CO)(C1)NC(C)=O	213,32	C12H23NO2
Z1795818456	CC(CC(O)=O)N1CCCCC1=O	185,22	C9H15NO3
Z1778350515	CC(O)C1=NOC(=N1)C1=CC=CC=C1	190,20	C10H10N2O2
Z316170380	CN1C=CC(=CC1=O)C(O)=O	153,14	C7H7NO3
Z94602039	CCC1=C(SN=N1)C(O)=O	158,18	C5H6N2O2S
Z228586390	OCC1=CN2C=CC=CC2=N1	148,17	C8H8N2O
Z382906830	CCNC(=O)C1=C(C)C2=C(S1)C=CS2	225,32	C10H11NOS2
Z449368988	CC1=C(C(N)=O)C(N)=CC=C1	150,18	C8H10N2O
Z449369198	CC1=C(C=C(C=C1)C(O)=O)S(C)(=O)=O	214,24	C9H10O4S
Z445129464	CC(=O)NC1CCCNC1	142,20	C7H14N2O
Z119815790	CC(C)C1=NN=C(NC(=O)C2CCC2)S1	225,31	C10H15N3OS
Z397585700	CC1=NC(=CC(=O)N1)C1=CC=C(N)C=C1	201,23	C11H11N3O
Z408255174	CS(=O)(=O)NCC(=O)N1CCCCC1	220,29	C8H16N2O3S
Z857667024	COCC1=NC(=CS1)C(O)=O	173,19	C6H7NO3S
Z168931250	CC1=NOC(CN2CCC(O)CC2)=C1	196,25	C10H16N2O2
Z134785326	CC(N1CCN(CC1)S(C)(=O)=O)C(N)=O	235,30	C8H17N3O3S
Z56887653	C[C@H](NC(C)=O)C1=NC2=C(N1)C=CC=C2	203,25	C11H13N3O
Z108544944	CN1C=CC(NC(=O)C2=CC=C(Cl)S2)=N1	241,69	C9H8ClN3OS
Z27167233	O=C(NC1=NN=C(S1)C1CC1)C1CCC1	223,29	C10H13N3OS
Z992717178	NC1=NC(=CC=C1)C(F)(F)F	162,12	C6H5F3N2
Z992717236	NC(=O)COC1=CN=CC=C1	152,15	C7H8N2O2
Z98976549	NC(=O)COC1=C(Cl)C=C(C=C1)C#N	210,62	C9H7ClN2O2
Z216777778	CN1C=C(Cl)C=C1C(=O)NC1=NN=CS1	242,68	C8H7ClN4OS
Z275127650	NCC1=CC2=C(N1)C=CC=C2	146,19	C9H10N2
Z1868316800	CC1=CN2C=CN=C(C(O)=O)C2=N1	177,16	C8H7N3O2
Z1473994889	CC(C)C(N1CCCS1(=O)=O)C(O)=O	221,27	C8H15NO4S
Z94598966	OCC1=NC2=C(C=CC=C2)C(=O)N1	176,18	C9H8N2O2
Z1266823190	CC(C)C1=NC2=C(CCNC2)N1C	179,27	C10H17N3
Z2104548225	CN1CCCC1C1COCCN1	170,26	C9H18N2O
Z1989522832	CC(O)CN1CCOC(C)C1	159,23	C8H17NO2
Z204781884	COC1CCN(CC1)C(C)C(=O)N(C)C	214,31	C11H22N2O2
Z238676600	CN1N=CC2=C1N=CN=C2N1CCC1	189,22	C9H11N5
Z1980876316	C[C@H]1CC[C@@H](CNC2=NC(C)=NS2)N1C	226,34	C10H18N4S
Z2161542370	CC1(C)N(CC2=CN=NS2)CCNC1=O	226,30	C9H14N4OS
Z228590106	CC1=NN(CCC(O)=O)C(C)=C1Cl	202,64	C8H11ClN2O2
Z234897683	C1CC(CCN1)C1=CNC2=C1C=CC=N2	201,27	C12H15N3
Z1262327430	COC1=C(N)C=C2SC(C)=NC2=C1	194,25	C9H10N2OS
Z1603826937	Cl.COC1=C(F)C=C2CNCCC2=C1	217,67	C10H13ClFNO
Z1413218090	CC(C)C1=NOC(=C1)C(=O)N1CC(O)C1	210,23	C10H14N2O3
Z1333043518	OC(=O)CN1C=C2C=CC=CC2=N1	176,18	C9H8N2O2
Z1266854821	CN1CC(=O)NC2=C1C=CC(F)=C2	180,18	C9H9FN2O
Z228586964	O=C(CCC1CCCC1)N1CCNCC1	210,32	C12H22N2O
Z2213893368	O=C1NCCCNC1CC1=CC=CC=C1	204,27	C12H16N2O
Z2327131607	COC1=C2CC(N)CC2=CC=C1	163,22	C10H13NO
Z108567110	NC(=O)N1CCCC(C1)C(O)=O	172,18	C7H12N2O3
Z57728664	NC(=O)CCN1C(=O)OC2=C1C=CC=C2	206,20	C10H10N2O3
Z104869826	NC(=O)CNC1=C(Cl)C=C(F)C=C1	202,61	C8H8ClFN2O

Z44585798	CC1=CC=C(NC(=O)N2CCCC2)C=C1	204,27	C12H16N2O
Z221429944	COC1=CC2=C(C=C1)C=C(CO2)C(O)=O	206,20	C11H10O4
Z220565334	OC(=O)CC1=CC2=C(OCC2)C=C1	178,19	C10H10O3
Z220564246	CN1N=CC(C(O)=O)=C1C	140,14	C6H8N2O2
Z220564190	CC1=CC(=O)N2NC(=NC2=C1)C(F)(F)F	217,15	C8H6F3N3O
Z1741976084	CC1(C)C(=O)NC2=C1C=CC=C2	161,20	C10H11NO
Z1863617863	CCN1C=C(N)C(=N1)C(N)=O	154,17	C6H10N4O
Z1729281082	CC(O)C1=NN(CC2=NC=CC=C2)C=C1	203,25	C11H13N3O
Z1860991849	O=C1NC=CC2=C1CNCC2	150,18	C8H10N2O
Z1849011609	NC1=C(C#N)C(=NN1)C1CC1	148,17	C7H8N4
Z1889902522	CC(CC(O)=O)C1=CC2=C(N1)C=CC=C2	203,24	C12H13NO2
Z1889902451	NC1=NC(=CC=N1)C1CCCNC1	178,24	C9H14N4
Z1889861358	CNC1=NN=C(C=C1)C(F)(F)F	177,13	C6H6F3N3
Z1889710303	CC1CC2=C(CN1)C=CC=C2N	162,24	C10H14N2
Z1889706343	CN1N=C(C=C1N)C1=CC(F)=CC(F)=C1	209,20	C10H9F2N3
Z1881599363	OC1COC2=C1C=CC(Cl)=C2	170,59	C8H7ClO2
Z1869801297	CC(C)OC1=CN=C(C=C1)C(O)=O	181,19	C9H11NO3
Z1868431107	CC1OCCC1(C)C(O)=O	144,17	C7H12O3
Z1222283852	CC1=CSC(=N1)N1CCCC(C1)C(O)=O	226,29	C10H14N2O2S
Z234898661	OC(=O)CCC1=CC(Cl)=CS1	190,64	C7H7ClO2S
Z1022691264	CC(C)(C(N)=O)C1=CC(Br)=CC=C1	242,12	C10H12BrNO
Z998307488	CNC(=O)C(C)(C)N1C=C(Br)C=N1	246,11	C8H12BrN3O
Z786289060	ClC1=CN(CC2=C(C=CC=C2)C#N)N=C1	217,66	C11H8ClN3
Z26824740	CCC1=NN=C(NC(=O)C2CCCC2)S1	225,31	C10H15N3OS
Z1116960368	CC1CCC(CN1CC=C(C)C)C(N)=O	210,32	C12H22N2O
Z375694448	CC1COCCN1C(=O)C1=CNC(=O)N1	211,22	C9H13N3O3
Z423217540	CNC(=O)C1=CN=C(S1)C1=CC=CC=C1	218,27	C11H10N2OS
Z287121492	COC1CCN(CC1)C(=O)NCC1CC1	212,29	C11H20N2O2
Z989260264	CN1CCC2=C(C1)C(=NC=N2)N1CCC1	204,28	C11H16N4
Z1575304447	OC1COC2=C1C=CC=C2	136,15	C8H8O2
Z2217052426	CC1C(O)CCCN1CC1=CC=CC=C1	205,30	C13H19NO
Z1569715418	NCC1=CC(OC2CCOC2)=NC=C1	194,23	C10H14N2O2
Z373768838	CCN1C=C(NC(=O)C2=CC=CS2)C=N1	221,28	C10H11N3OS
Z1172115961	CC1=NN=C2CCC(CN12)C(O)=O	181,20	C8H11N3O2
Z1172068249	CC1=CC=NC2=C(C=NN12)C(O)=O	177,16	C8H7N3O2
Z1171979272	OC(=O)C1=CC2=C(NC(=N2)C(F)F)C=C1	212,16	C9H6F2N2O2
Z354989594	CC(C)NC(=O)CN1CC(C)OCC1C	214,31	C11H22N2O2
Z177089660	CCN(CC1=CC=C(Cl)S1)C(=O)NC	232,73	C9H13ClN2OS
Z1188327367	CCN(C)C(=O)NCC1=CC(Br)=CC=C1	271,16	C11H15BrN2O
Z1661228897	CC1=NN=C(O1)C1=CC(Br)=CN=C1	240,06	C8H6BrN3O
Z1429880392	CN1N=C(C)C(C#N)=C1N	136,16	C6H8N4
Z2492800236		210,23	C10H14N2O3
Z2492774613	NC1=C(N=C(CC2=CC=CC=C2)O1)C#N	199,21	C11H9N3O
Z2492774455	O=C1C=CNC2=C1CCCC2	149,19	C9H11NO
Z2489418741	OC(=O)C1=CN(C2CC2)C(=O)C=C1	179,18	C9H9NO3
Z2489418705		141,21	C8H15NO
Z818732094	NC(C(O)=O)C1=C(Br)C=CC=C1	230,06	C8H8BrNO2
Z345570098	CN(CC1=C(Cl)C=CC=C1Cl)C(C)=O	232,10	C10H11Cl2NO
Z2472860092	OCC1CC2=C(CO1)C=CC=C2	164,20	C10H12O2
Z270828822	CN(C)C(C(O)=O)C1=CC=C(Cl)C=C1	213,66	C10H12ClNO2
Z2510258126	CN1C(=O)CC2=C1C=C(C=C2)C(O)=O	191,19	C10H9NO3
Z2167281359	CC1=C(SC(=N1)C1CCOC1)C(O)=O	213,25	C9H11NO3S
Z2510258228	OC(=O)C1=C(C=CO1)C1CC1	152,15	C8H8O3
Z2510258200	CN1C=C(C(O)=O)C2=C1C=CC(F)=C2	193,18	C10H8FNO2
Z1982493938	CC1=C(N)C=C(C=C1)C1=NC(=O)NN1	190,21	C9H10N4O
Z1981870468	CC1=C(C(O)=O)C(F)=CC(Br)=C1	233,04	C8H6BrFO2
Z1551688335	CC(O)C(=O)NCC1=NC=CC=C1	180,21	C9H12N2O2
Z199534086	CC1OC2=C(OC1C(O)=O)C=CC=C2	194,19	C10H10O4
Z1966565729	CN1N=NC2=C1C=CC(Br)=C2	212,05	C7H6BrN3
Z1891772277	CN1N=C(C)C2=C1N=C(C=C2)C(O)=O	191,19	C9H9N3O2
Z1956828432	C1CN2C(CN1)=NN=C2C1=CN=CC=C1	201,23	C10H11N5
Z1956828705	CC1=C(C)C(N)=CC(=C1)S(C)(=O)=O	199,27	C9H13NO2S
Z1328078283	CC1(C)CN(C(=O)NCC2CC2)C1(C)C	210,32	C12H22N2O
Z1948933973	OC(=O)C1=CON=C1C1CC1	153,14	C7H7NO3
Z1948934180	CC(C)OC1=C(C=CN=C1)C(O)=O	181,19	C9H11NO3
Z1947444027	NC1=C(C=CC=C1)C(O)C1=NC=CC=C1	200,24	C12H12N2O
Z734661922	CCNC(=O)C1=CC2=C(C=CC=C2)N1C	202,26	C12H14N2O

Z1946684524	CC1CCC(CC1)OCC(O)=O	172,22	C9H16O3
Z1945984086	OC(=O)CCC1=NC=CN=C1	152,15	C7H8N2O2
Z1270207942	NCC(O)C1=CC=C(Cl)S1	177,65	C6H8ClNOS
Z228585324	CC1=CC(CO)=C(C)N1C1CC1	165,24	C10H15NO
Z993967192	CC(N1CCCCC1=O)C(O)=O	171,20	C8H13NO3
Z404993336	CN1C=C(NC(=O)C2=NOC(C)=C2)C=N1	206,21	C9H10N4O2
Z237578996	CC(C)CNC1=C2C=NNC2=NC=N1	191,24	C9H13N5
Z17828979	CNC(=O)COC1=C(Br)C=CC=C1	244,09	C9H10BrNO2
Z56789829	O=C(NC1CCS(=O)(=O)C1)C1=CC=CO1	229,25	C9H11NO4S
Z56774274	NC(=O)CN1N=NC2=C1C=CC=C2	176,18	C8H8N4O
Z2692093659	C1CC1C1=NC2=C(CNCC2)C=N1	175,24	C10H13N3
Z2692093591	OC(=O)C1(O)CCC2=C(C1)C=CC(Br)=C2	271,11	C11H11BrO3
Z2690050348	NC(CC(O)=O)C1=CC(Cl)=CS1	205,66	C7H8ClNO2S
Z1653345710	CC(C)C(=O)N(C)C1=C(Br)C=CC=N1	257,13	C10H13BrN2O
Z1342879039	COC1=C(C=C(Cl)C(Cl)=C1)C(O)=O	221,03	C8H6Cl2O3
Z1834265808	COC1=C(N)C2=C(CCC2)C=C1	163,22	C10H13NO
Z1259041039	CC1CCC(N)(CC1)C(O)=O	157,21	C8H15NO2
Z1259040932	CC1=C(C=CC(F)=C1)C#N	135,14	C8H6FN
Z1258992717	O=C(NC1CCCNC1)C1=CC=CC=C1	204,27	C12H16N2O
Z1232889846	CC(NS(C)(=O)=O)C1=C(Cl)C=C(F)C=C1	251,70	C9H11ClFNO2S
Z1258578221	CC(CN)C1=CC(C)=CC=C1	149,24	C10H15N
Z228588744	OC(=O)CNC(=O)C1=NC=CC=C1	180,16	C8H8N2O3
Z1250132697	OC(=O)C1=CC=C(C=C1)N1CC=CC1	189,21	C11H11NO2
Z1250132662		176,22	C11H12O2
Z1250132611	CC(C)NC1CCOCC1	143,23	C8H17NO
Z33545896	CS(=O)(=O)C1=CC(=CC=C1)C(N)=O	199,22	C8H9NO3S
Z929052328	CC(C)(NC(=O)C1=CSC(Br)=C1)C(O)=O	292,15	C9H10BrNO3S
Z1262237317	CS(=O)(=O)C1=C(F)C=C(N)C=C1	189,20	C7H8FNO2S
Z57275697	CC1=CC2=C(NC=C(C(O)=O)C2=O)C=C1	203,20	C11H9NO3
Z1262237202	CCC1=CC=C(C=C1)C1=NC(N)=NN=C1	200,25	C11H12N4
Z1259341106	CC1=CC(C(O)=O)=C(S1)C(O)=O	186,18	C7H6O4S
Z1259341078	CC1=CC(C)=C(C=C1)C1=NOC(N)=C1	188,23	C11H12N2O
Z1259340992	OC(=O)CC1CCOC2=C1C=CC=C2	192,21	C11H12O3
Z1259340995	O=C1CCCC2=C(N1)C=CS2	167,23	C8H9NOS
Z1259340009	CNC1(CCOCC1)C(O)=O	159,19	C7H13NO3
Z1259339936	CC(C)C1=NOC(=C1)C(O)=O	155,15	C7H9NO3
Z1259339892	FC1=C(NC2CCNCC2)C=CC=C1	194,25	C11H15FN2
Z57469736	CC1=CC=C(C=C1)C1=NON=C1N	175,19	C9H9N3O
Z1259339887	CS(=O)(=O)C1=CC=C(CCN)C=C1	199,27	C9H13NO2S
Z1259339747	C(C1CCCN1)N1CCOCC1	170,26	C9H18N2O
Z1259335898	CC1=C(F)C=CC(=C1)C(O)=O	154,14	C8H7FO2
Z1259335889	OC(=O)C1CC1C1=C(F)C=CC(F)=C1	198,17	C10H8F2O2
Z1259335801	OC1=NC(=NC=C1)C1CC1	136,15	C7H8N2O
Z1259335793	CC(C)C1=NC(O)=CC=N1	138,17	C7H10N2O
Z1259273395	CC1=C2NC=C(CCC(O)=O)C2=CC=C1	203,24	C12H13NO2
Z1259273129	CCN(C)C(=O)C1=CC(=CC=C1)C(O)=O	207,23	C11H13NO3
Z1259273099	OC(=O)COC1=C(F)C=CC(F)=C1	188,13	C8H6F2O3
Z1259162080	OC(=O)C1CC1C1CCCCC1	168,24	C10H16O2
Z1259161894	CC1=CC(C(O)=O)=C(F)C=C1	154,14	C8H7FO2
Z1259161842	CCC(C)C(=O)N1CCCC(C1)C(O)=O	213,28	C11H19NO3
Z1462145959	CC1=CC(NC2CCNC2=O)=CC(F)=C1	208,24	C11H13FN2O
Z2142244288	CCNC1=C(F)C=C(C=C1)S(C)(=O)=O	217,26	C9H12FNO2S
Z1267882073	CNC1CCN(CC1)C1=NC=CC=N1	192,27	C10H16N4
Z1263811740	CC(=O)N1CCC(CN)CC1	156,23	C8H16N2O
Z1951532824	C(N1CCC[C@H]2OCC[C@@H]12)C1=CSN=N1	225,31	C10H15N3OS
Z2219060251	CC1=NOC=C1CNC1=CC(F)=CC=C1	206,22	C11H11FN2O
Z137632340	FC1=CC=C(NCC2=CSC=N2)C=C1	208,25	C10H9FN2S
Z137740702	CN1C=C(CNC2=CC(F)=CC=C2)C=N1	205,24	C11H12FN3
Z2687203888	OC(=O)CC1(CC1)C1=CC=CC=C1	176,22	C11H12O2
Z2271007255	CCNC1=NC=C(C=N1)C(F)F	173,17	C7H9F2N3
Z2684296057	CCC1=NN(C(=O)C1)C1=NC=C(N)C=C1	204,23	C10H12N4O
Z1171979224	CC1=CC(=CC=C1)N1C=C(N=N1)C(O)=O	203,20	C10H9N3O2
Z57481220	OC(=O)C1=CC=C(CN2C=CC=C2)C=C1	201,23	C12H11NO2
Z1171978785	CN1N=NC2=C1C=CC(=C2)C(O)=O	177,16	C8H7N3O2
Z221226202	CC1CN(CCO1)C(=O)C1=CC(Cl)=CN1	228,68	C10H13ClN2O2
Z1165167999	CC(NC(=O)C1CCOCC1)C(O)=O	201,22	C9H15NO4
Z1162910738	NC(=O)C1(N)CCOCC1	144,17	C6H12N2O2

Z32364066	COCC(=O)NC1=C(Cl)C=C(F)C=C1	217,62	C9H9ClFNO2
Z373769120	CCN1C=C(NC(=O)C2=CC=CO2)C=N1	205,22	C10H11N3O2
Z1160899528	OC(=O)CC1OCCC2=C1SC=C2	198,24	C9H10O3S
Z196120788	CC(O)CNC1=C2SC=CC2=NC=N1	209,27	C9H11N3OS
Z166702366	O=C(NC1=CN=CC=C1)N1CCCC1	191,23	C10H13N3O
Z1768160698	COCC1=NC(C)=C(S1)C(O)=O	187,21	C7H9NO3S
Z332722148	CN1C=CC(NC(=O)C2=COC(Br)=C2)=N1	270,09	C9H8BrN3O2
Z1271581412	CC1=NOC(CN2C=CC=C(C)C2=O)=N1	205,22	C10H11N3O2
Z1762997350	Cl.COC1=C(F)C(=CC=C1)C1CCNCC1	245,72	C12H17ClFNO
Z1762997277	CN(C)C1=C(C=NC=C1)C(O)=O	166,18	C8H10N2O2
Z1762658189	NC1=C(OC(F)F)C=CC(=C1)C(O)=O	203,15	C8H7F2NO3
Z1742055002	CNC1=NC=C(OC)C=N1	139,16	C6H9N3O
Z1696936039	O=C(NC1=NC=CN=C1)C1CC1C1CC1	203,25	C11H13N3O
Z1651637958	NC(=O)COC1=C(Br)C=C(F)C=C1F	266,04	C8H6BrF2NO2
Z1551999220	CC(CC1=CC=C(Br)C=C1)C(N)=O	242,12	C10H12BrNO
Z1649283124	CCC(C)N1CCNC(=O)C1C(C)C	198,31	C11H22N2O
Z286103228	CNC(=O)CCN1N=C(C)C=C1C	181,24	C9H15N3O
Z1262252971	OCCN1CCCCC1=O	157,21	C8H15NO2
Z1346370629	CC1CN(CCC1N)S(C)(=O)=O	192,28	C7H16N2O2S
Z1566599209	C[C@@H]1CN(CCN1)C1CCOCC1	184,28	C10H20N2O
Z1741966630	CC(C)(N)CN1CCCC1	142,25	C8H18N2
Z1245580425	CC1CCCCN1CCN	142,25	C8H18N2
Z1954803140	C1CN(CCO1)C1CCCNCC1	184,28	C10H20N2O
Z2677388736	COC1=C(SC=C1)S(=O)(=O)N1CC=CC1	245,31	C9H11NO3S2
Z2146513998	CC1=CC=C(CCC(O)=O)S1	170,23	C8H10O2S
Z1891775084	CNC1=NN=C2CCNCC2=C1	164,21	C8H12N4
Z1823337665	CC1=C(C=CO1)S(=O)CC1=CC=CC=C1	220,29	C12H12O2S
Z1741981630	NC12CC3CC(C1)CC(O)(C3)C2	167,25	C10H17NO
Z2111525701	CC1=C(C)C(CO)=C(O)N=N1	154,17	C7H10N2O2
Z2111524565	COC1=CC=C(C=C1)C1=C(N)ON=C1	190,20	C10H10N2O2
Z2092370890	NC1=C2CNC(=O)C2=CC=C1	148,17	C8H8N2O
Z2092370157	COC1=C(F)C(N)=C(C=C1)C(O)=O	185,15	C8H8FNO3
Z2082999988	NC1=CN=C(CCO)C=C1	138,17	C7H10N2O
Z1942253409	OC1CCC(CC1)C(=O)NC1CC=CC1	209,29	C12H19NO2
Z2106610741	OC(=O)C1=NN2CCOC2=C1	154,13	C6H6N2O3
Z2158595086	NC1=C(C=C(F)C(F)=C1)N1CCCCC1	212,24	C11H14F2N2
Z228574284	CN(C)C(CN)C1CCCCC1	170,30	C10H22N2
Z1246465616	CC(CC1=C(C)ON=C1C)C(=O)N(C)C	210,28	C11H18N2O2
Z1501469877	CN1C=C(C=N1)C(N)CN	140,19	C6H12N4
Z1577369655	CC1=NC=C(CNC2CCCOCC2)S1	226,34	C11H18N2OS
Z1250132788	CCC(NC)C1=CC=NC=C1	150,23	C9H14N2
Z1262237477	COC1=C(C=CC=C1)C(C)(C)CN	179,26	C11H17NO
Z168922792	COCCN1CCCC(O)C1	159,23	C8H17NO2
Z803153678	CC(N)COC1=C(C)C=CC=C1	165,24	C10H15NO
Z228570960	CC(C)C(CN)N1CCCC1	156,27	C9H20N2
Z1444214989	CC(C)(NC(=O)C1=CSC=N1)C(O)=O	214,24	C8H10N2O3S
Z1696844496	CN1C=C(C=N1)C(O)CN1CCNCC1	210,28	C10H18N4O
Z857667076	NCC1=NC(=CS1)C1=CC=CC=C1	190,26	C10H10N2S
Z910842756	CC(C)C1=NC(C(O)=O)=C(Cl)C=N1	200,62	C8H9ClN2O2
Z234897013	O=C1NC2=C(COCC2)C=C1C#N	176,18	C9H8N2O2
Z234896997	CCNC(=O)C1=CC=C2C(C)NCCN12	207,28	C11H17N3O
Z234896787	CC1=C2C(CCN)=CNC2=CC=C1	174,25	C11H14N2
Z29418402	ClC1=C(C=CC(NC(=O)C2CC2)=C1)C#N	220,66	C11H9ClN2O
Z55293743	CN(C)C(=O)NC1=CC=C(C)C=C1	178,24	C10H14N2O
Z57981094	CC1=C(CCC(O)=O)C(C)=NO1	169,18	C8H11NO3
Z227830198	CN(C)S(=O)(=O)C1=C(CN)C=CC=C1	214,28	C9H14N2O2S
Z45527644	CN(C)S(=O)(=O)C1=C(C)ON=C1C	204,24	C7H12N2O3S
Z33545306	CN(C)S(=O)(=O)C1=CC=C(C=C1)C(N)=O	228,27	C9H12N2O3S
Z970066372	CC1=CC=C(C=C1)S(=O)(=O)N1C=CC=N1	222,26	C10H10N2O2S
Z90507809	CC1CCCCC1NCC1=CN=CC=C1	204,32	C13H20N2
Z90539031	CC1CCC(CC1)NCC1=CC=NC=C1	204,32	C13H20N2
Z768399682	CC1CN(C(C)CO1)C(=O)C1=NSN=C1	227,28	C9H13N3O2S
Z373768898	CCN1C=C(NC(=O)C2CCCC2)C=N1	207,28	C11H17N3O
Z275727840	CC(N1CCCCCC1)C1=NC(C)=NO1	209,29	C11H19N3O
Z134991794	CC(N)C(=O)NCC1=CC=CO1	168,20	C8H12N2O2
Z916847036	CC(N)C1=C(C)OC(C)=C1	139,20	C8H13NO
Z184825330	CCOC1=C(C=CC=N1)C(=O)N(C)C	194,23	C10H14N2O2

Z369936976	CN(CC1=NOC(C)=C1)C(=O)NC1CC1	209,25	C10H15N3O2
Z32014360	CN(C)C(=O)CC1=CC=CS1	169,24	C8H11NOS
Z416877194	CC1=NOC(CN2CCC(CO)CC2)=N1	211,27	C10H17N3O2
Z812517094	CN1C=CC2=C1C=C(N)C=C2	146,19	C9H10N2
Z763367884	CC(N)COC1=C(F)C=CC=C1	169,20	C9H12FNO
Z234897025	COC1=CC(N)=C(OC(F)F)C=C1	189,16	C8H9F2NO2
Z2327226136	CC1=C(C(N)=CC=C1)S(C)(=O)=O	185,24	C8H11NO2S
Z2327226154	CC(CC(O)=O)C1=CN(C)N=C1	168,20	C8H12N2O2
Z2235389984	CN1NC2=C(C=C(Br)C=N2)C1=O	228,05	C7H6BrN3O
Z2306625465	OC(=O)C1(CCC1)NC1CC1	155,20	C8H13NO2
Z57101324	CNC1=C2C=NNC2=NC=N1	149,16	C6H7N5
Z2301499922	COC1=CC(C(O)=O)=C(F)C(Cl)=C1	204,58	C8H6ClFO3
Z1954802631	OC(=O)[C@H]1CC[C@H](C1)C(O)=O	158,15	C7H10O4
Z2293604761	CN1N=CC2=C1CCCNC2=O	165,20	C8H11N3O
Z1891775475	C(CC1=CC=CC=C1)C1CNCCO1	191,27	C12H17NO
Z1229289858	NC(=O)N1CCC[C@H]1CO	144,17	C6H12N2O2
Z1223130943	CC(C)C1CCC(C)CCN1CC(N)=O	212,34	C12H24N2O
Z1267773736	CC(NC1=C(N)C=NC=C1)C1CCCO1	207,28	C11H17N3O
Z1266933958	CN1CCC2=C(C1)C=C(C#N)C(N)=N2	188,23	C10H12N4
Z1266933873	CC1=NC(=NC(N)=N1)C1=CC=CS1	192,24	C8H8N4S
Z1266823254	CC1=NN=C2CNCCN12	138,17	C6H10N4
Z1266823144	CCOCC1=NN=C(N)O1	143,15	C5H9N3O2
Z1263820357	CC(C)(C1=NN=C(N)S1)C1=CC=CC=C1	219,31	C11H13N3S
Z1263820310	C1CN(CCN1)C1=NC2=C(O1)C=CC=C2	203,25	C11H13N3O
Z1263714070	NC(=O)C1CNC2=C1C=CC=C2	162,19	C9H10N2O
Z1263602416	CS(=O)(=O)C1=C(Cl)C=C(N)C=C1	205,66	C7H8ClNO2S
Z2234185700	OC(=O)CC1=C(Br)C=CS1	221,07	C6H5BrO2S
Z2379767235	C1CC1C1=CSC(=N1)C1=NN=CN1	192,24	C8H8N4S
Z1437108487	CC1CCC(NCCO)C2=C1C=CC=C2	205,30	C13H19NO
Z2417926821	CC(C)CN1C=NN2C=CC=C2C1=O	191,23	C10H13N3O
Z90507767	CN1CCC(CC1)NCC1=CN=CC=C1	205,31	C12H19N3
Z1263811784	CCC(C)(N)C1=CC=CC=C1	149,24	C10H15N
Z1840712742	CC1OCCCC1C(=O)N1CC(CO)C1	213,28	C11H19NO3
Z1266933909	CC(N)C1=NC2=C(N1)C=CC(Cl)=C2	195,65	C9H10ClN3
Z1250132798	C1CNCC(C1)C1=NC=NC=C1	163,22	C9H13N3
Z90122149	CNC(C)C1=NC2=C(S1)C=CC=C2	192,28	C10H12N2S
Z1949075848	NC1CCC(C1)C(=O)N1CCCC1	182,27	C10H18N2O
Z1262252935	NCC1=CN=C(OC2=CC=CC=C2)C=C1	200,24	C12H12N2O
Z744843724	CCN(C)S(=O)(=O)C1=C(C)NN=C1C	217,29	C8H15N3O2S
Z1863624700	COC1CCN(CC1)C1=C(C)C=NC=C1	206,29	C12H18N2O
Z57161818	C(N1C=NC=N1)C1=CC=CC=C1	159,19	C9H9N3
Z1614439780	CN1C=CC=C1C(=O)N1CCC=C(F)C1	208,24	C11H13FN2O
Z1815390993	FC1=CN=C(CN2N=CSC2=O)C=C1	211,21	C8H6FN3OS
Z1619979835	CN1C=NC=C1CN1CCC=C(F)C1	195,24	C10H14FN3
Z1587273210	CC1=NC(CN2CCC=C(F)C2)=NO1	197,21	C9H12FN3O
Z1636210119	CC1=NSC=C1C(=O)N1CCC=C(F)C1	226,27	C10H11FN2OS
Z1815382056	CCC1=NC=CN1CC1=NC=C(F)C=C1	205,24	C11H12FN3
Z2017168803	C[C@@H](CO)N(C)C1=C(F)C=CC=N1	184,21	C9H13FN2O
Z1796028750	NC(=O)C1(CC2=C(F)C=CC=C2)CC1	193,22	C11H12FNO
Z2273523339	C[C@H](NC1=NC=C(F)C=N1)C1CC1	181,21	C9H12FN3
Z281802048	COC1=NC(=NC=C1)N(C)C	153,19	C7H11N3O
Z2255142757	CN1C=CN=C1C1CC(N)CCO1	181,24	C9H15N3O
Z1262254353	CCC(=O)NC1CCCNC1	156,23	C8H16N2O
Z28870646	CC1CCC2=C(C=CC=C2)N1C(C)=O	189,26	C12H15NO
Z2213892991	CC1(C)CC(CC(O)=O)C1	142,20	C8H14O2
Z1918536193	CN1C=C(OC2=C(Cl)C=NC=N2)C=N1	210,62	C8H7ClN4O
Z2067304833	CCNC1=C(C)N(N=C1)C1=CC=CC=C1	201,27	C12H15N3
Z2326990059	CNCC1=CC(Cl)=C(OC)C=C1	185,65	C9H12ClNO
Z2235790913	CC(C)OC1=C(N)C=C(Cl)C=N1	186,64	C8H11ClN2O
Z2301499709	C1CC(C1)C1CNCCO1	141,21	C8H15NO
Z172492286	CN1CCN(CCN)CC1	143,23	C7H17N3
Z2327884032	CC1CN(CCO)C(C)CN1	158,25	C8H18N2O
Z1354785445	O[C@H]1CN(CC(F)F)C[C@H]1O	167,16	C6H11F2NO2
Z104442660	CC1OC2=C(NC1=O)C=C(N)C=C2	178,19	C9H10N2O2
Z89283375	OCC1=CC2=C(OCCCO2)C(Cl)=C1	214,65	C10H11ClO3
Z57756572	CN(C)C1=CC=C(C=C1)C#N	146,19	C9H10N2
Z85927317	CC1=C(Cl)C=C(NC(=O)CO)C=C1	199,63	C9H10ClNO2

Z85922627	OC(=O)CCC1=NC2=C(C=CC=C2)C=C1	201,23	C12H11NO2
Z85923150	CCS(=O)C1=C(C=CC=C1)C(O)=O	198,24	C9H10O3S
Z85923398	CCC1=NOC(C)=C1C(O)=O	155,15	C7H9NO3
Z85921034	CC1=NN(C(C)=C1CO)C1=CC=CC=C1	202,26	C12H14N2O
Z57980672	OC(=O)CN1CCCC1=O	143,14	C6H9NO3
Z56974908	OC(=O)CN1C(=O)OC2=C1C=CC=C2	193,16	C9H7NO4
Z818810474	CC1=CSC(=N1)N1CCCNCC1	197,30	C9H15N3S
Z433017950	CNC(=O)CN(C)C(=O)C1=C(Br)C=CS1	291,16	C9H11BrN2O2S
Z133755368	CN(CC(=O)N1CCOCC1)S(C)(=O)=O	236,29	C8H16N2O4S
Z382906588	CNC(=O)C1=C(C)C2=C(S1)C=CS2	211,30	C9H9NOS2
Z420008560	CC1=C(SC=N1)C(=O)NCC1=CC=CO1	222,26	C10H10N2O2S
Z31182072	NC(=O)C1CCCN(C1)C1=NC=CC=N1	206,25	C10H14N4O
Z419994690	CC1CCCN(C1)C(=O)C1=C(C)N=CS1	224,32	C11H16N2OS
Z422368058	CC1CN(CCO1)C(=O)C1=C(C)N=CS1	226,29	C10H14N2O2S
Z216777796	O=C(CN1C=CC=N1)NC1=NN=CS1	209,23	C7H7N5OS
Z140107416	CN(C)C(=O)CNC(=O)C1=CC(Br)=CN1	274,12	C9H12BrN3O2
Z752370852	OC(=O)CC1=CC(Br)=C(F)C=C1	233,04	C8H6BrFO2
Z237732034	CC1=C(N)C=C(C=C1N)C(O)=O	166,18	C8H10N2O2
Z235361315	CCC(=O)NC1=C(C)C(N)=CC=C1	178,24	C10H14N2O
Z32023832	CCNC(=O)C1=CC(=CC=C1)S(C)(=O)=O	227,28	C10H13NO3S
Z381785674	CN(C)C(=O)N1CCCC(C1)C(O)=O	200,24	C9H16N2O3
Z235354537	OCCN1C=C2CNCCC2=N1	167,21	C8H13N3O
Z133632670	CS(=O)(=O)N1CCCC(C1)C(O)=O	207,24	C7H13NO4S
Z355180744	CN(C)S(=O)(=O)N1CCC2=C(C1)C=CS2	246,34	C9H14N2O2S2
Z32016296	CNC(=O)C1=CC2=C(C=C1)N=CS2	192,24	C9H8N2OS
Z367678716	CC1=NNC(=O)C(C(O)=O)=C1C	168,15	C7H8N2O3
Z367678540	NCC(O)C1=C(F)C=CC=C1	155,17	C8H10FNO
Z367452368	CC1=C(C=NN1C1=NC=CC=C1)C(O)=O	203,20	C10H9N3O2
Z367452182	NC1=CC(=CC=C1)S(=O)(=O)C1CCCC1	225,31	C11H15NO2S
Z2470036279	OC(=O)CC1OCC2=C1C=CC(Cl)=C2	212,63	C10H9ClO3
Z1716711827	CN(C)C1=C(Cl)C=C(C=N1)C(O)=O	200,62	C8H9ClN2O2
Z208331706	COC1=CC2=C(NC=C2)C=C1	147,18	C9H9NO
Z1741972926	OC(=O)C1=CSC(=N1)N1CCCCC1	212,27	C9H12N2O2S
Z1741982217	OC(=O)C1CCC2=C(C1)C=CC=C2	176,22	C11H12O2
Z2467454615	CS(=O)(=O)N1C=CC(N)=N1	161,18	C4H7N3O2S
Z2466331661	CC1=C(Br)C=C2NC(=O)NC2=N1	228,05	C7H6BrN3O
Z2465619864	CN1N=NC2=C1C=CN=C2	134,14	C6H6N4
Z2465619834	CN1C=C(C=N1)C1=C(OC=C1)C(O)=O	192,17	C9H8N2O3
Z68259725	OCC1=C(NC(=O)C2CC2)C=CC=C1	191,23	C11H13NO2
Z2429425665	CC1=NN=C(C=C1)C1=CC=C(CO)C=C1	200,24	C12H12N2O
Z360149094	CN1N=CC(=O)C2=C1C=CC=C2	160,18	C9H8N2O
Z2418194212	OC(=O)C1=CN(N=N1)C1CCC1	167,17	C7H9N3O2
Z2418193716	O=C1NCCCC2=C1C=NN2	151,17	C7H9N3O
Z2418193710	CN1C=CC=C1CCC(O)=O	153,18	C8H11NO2
Z1266823365	CCC1=NN2CC(N)CCC2=N1	166,23	C8H14N4
Z1263811750	CCC(N)C1=NC(C)=CS1	156,25	C7H12N2S
Z1020684212	CC(O)(CN)C1=CC=CC=C1	151,21	C9H13NO
Z1258948114	CCC(C)(N)C1=NC(C)=CS1	170,27	C8H14N2S
Z1263811761	CC(C)OC1=C(F)C=C(CN)C=C1	183,23	C10H14FNO
Z1269702204	COC1=NC=C(CO)C=C1	139,15	C7H9NO2
Z1354432973	COC1CCN(CCN)CC1	158,25	C8H18N2O
Z1675167270	CC1=NC=C(CCO)N=C1	138,17	C7H10N2O
Z54813718	CS(=O)(=O)N1CCC(CC1)C(O)=O	207,24	C7H13NO4S
Z31131631	COC1=C(C=CC=N1)C#N	134,14	C7H6N2O
Z1238477803	CC1=C(C)N(CCC(O)=O)C(=O)S1	201,24	C8H11NO3S
Z1222331427	OC(=O)C1=CC2=C(NCCC(=O)N2)C=C1	206,20	C10H10N2O3
Z285777804	CC(C)NC1=C(C=CC=C1)S(C)(=O)=O	213,30	C10H15NO2S
Z839522486	CC1=NOC(=C1)C1CCCN1	152,20	C8H12N2O
Z2060794587	O=C(C1CCOC1)N1CCN2N=CC=C12	207,23	C10H13N3O2
Z32015055	CN(C)C(=O)CC1=CC(F)=CC=C1	181,21	C10H12FNO
Z1258578164	CCC1=NC(C)=C(S1)C(C)NC	184,30	C9H16N2S
Z1267773577	CNC1CCCC2=C1SC(C)=N2	182,29	C9H14N2S
Z228587862	C1CNCCN(C1)C1=NC=CS1	183,27	C8H13N3S
Z220564284	NCCC1=NN=C2CCCCCN12	180,26	C9H16N4
Z1333043406	CCC1=NC=NC(=C1)N1CCC(N)CC1	206,29	C11H18N4
Z360056130	COC1=C(Br)C=C(C=C1)[C@@H](C)O	231,09	C9H11BrO2
Z1275599911	CC(=O)N1CCCC2(C1)CCCCC2	209,33	C13H23NO

Z2240999730	OC(=O)C1CC(O)(C1)C1CCCCC1	198,26	C11H18O3
Z145119960	NC(=O)C1=NC=CC(=C1)N1CCCC1	191,23	C10H13N3O
Z2238508676	O=C1NC(CC2=CC=NC=C2)=NO1	177,16	C8H7N3O2
Z1954804097	CN1N=C(C=C1C1=CN=CC=C1)C(O)=O	203,20	C10H9N3O2
Z1166114418	OC(=O)C1=CC2=C(CCCC2)N1	165,19	C9H11NO2
Z1463415030	CCC1=C(C)NC2=NC(C)=NN2C1=O	192,22	C9H12N4O
Z970076882	CN1N=C(C=C1C1=CC=CC=C1)C(O)=O	202,21	C11H10N2O2
Z2235810181	OC(=O)CC1COC2=C(O1)C=CC=C2	194,19	C10H10O4
Z2235810193	CCOC1=NC2=C(O1)C=CC(N)=C2	178,19	C9H10N2O2
Z354992234	CC1CN(CC2=C(Cl)SN=N2)C(C)CO1	247,74	C9H14ClN3OS
Z2255112293	OC(=O)C1(CCC1)C1CCC1	154,21	C9H14O2
Z1796678844	COC1=NC(CN2N=CSC2=O)=CC=C1	223,25	C9H9N3O2S
Z94599212	CCOC1=CC=C(C=C1)C1CCCN1	191,27	C12H17NO
Z1127932252	CNC(=O)CC1=C(C=CC=C1)C(F)(F)F	217,19	C10H10F3NO
Z1143401905	CNC(=O)C1=C(Br)C(=NN1)C(C)C	246,11	C8H12BrN3O
Z1250288840	CC(=O)N(CC(N)=O)C1=CC=C(C)C=C1	206,25	C11H14N2O2
Z1245537949	CC(C)CC(C)N1N=CC=C1N	167,26	C9H17N3
Z1939437573	CC1=C(C(C)=NO1)C1=CC=CC=C1	173,22	C11H11NO
Z234894327	NCC1=CC(=NC=C1)N1CCCC1	177,25	C10H15N3
Z1259339822	CC(CN)CC1=CC=CS1	155,26	C8H13NS
Z1269702487	CCN1CCC(N)CC1C	142,25	C8H18N2
Z991556980	CN(C)C1=NC(CN)=CS1	157,24	C6H11N3S
Z1258948093	CN1C=C(C=N1)C(C)(C)N	139,20	C7H13N3
Z1798265228	CC(O)CN(C)C1=C(C=CC=C1Cl)C#N	224,69	C11H13ClN2O
Z278210580	CCS(=O)(=O)N1CCC(N)CC1	192,28	C7H16N2O2S
Z1798264062	CC(O)CN(C)C1=C2C=COC2=CC=N1	206,25	C11H14N2O2
Z1185276742	CCOC1=C(Cl)C=C(C=N1)C(=O)N(C)C	228,68	C10H13ClN2O2
Z1591867242	CS(=O)(=O)N1CCN(CC1)C(=O)CO	222,26	C7H14N2O4S
Z1696844737	N[C@H](C(O)=O)C1=CC=C(F)C=C1	169,16	C8H8FNO2
Z1695922870	CCS(=O)(=O)C1=CC(C=C(Cl)C=C1)C(O)=O	248,68	C9H9ClO4S
Z32016909	CNC(=O)C(C)NC(=O)C1=CC=CC=C1	206,25	C11H14N2O2
Z1679733364	O=C1N(CC2CCC2)N=C2C=NC=CN12	204,23	C10H12N4O
Z1996939123	NC(C(N)=O)C1=CNC2=C1C=CC=C2	189,22	C10H11N3O
Z1095000458	CC(C)(O)CNC1=C(C#N)C(F)=CC=C1	208,24	C11H13FN2O
Z2028214375	COC1=C(C=C(Cl)C=C1N)C(=O)N(C)C	228,68	C10H13ClN2O2
Z2027049516	OCC1=CN(N=N1)C1=C(Br)C=CC=C1	254,09	C9H8BrN3O
Z1791180120	CN1N=C(C)C=C1C(=O)NC1=CN=NS1	223,25	C8H9N5OS
Z1741976022	BrC1=C2CNC(=O)C2=CC=C1	212,05	C8H6BrNO
Z2017843139	CN1C=C(C=N1)C1CCCCN1	165,24	C9H15N3
Z1820273488	CC1=CSC=C1C(=O)NC1=CN=NS1	225,28	C8H7N3OS2
Z2010041964	COC1=NC(C)=CC(=C1)C#N	148,17	C8H8N2O
Z2010010272	OC(=O)C1=NC=C2CCCCC2=C1	177,20	C10H11NO2
Z1822510252	CC1=C(CN2N=CSC2=O)N=CC=C1	207,25	C9H9N3OS
Z1623872182	CC(C)CC1=CC(=CC(=O)N1)C(O)=O	195,22	C10H13NO3
Z446250928	CN(C1CCS(=O)(=O)C1)C(=O)NCC=C	232,30	C9H16N2O3S
Z1603826964	CC1=CC(=CC(=O)N1)C(O)=O	153,14	C7H7NO3
Z1578014146	CNC1=C(OC)C=CC=N1	138,17	C7H10N2O
Z1575304381	CC1=CC2=C(N=C1)N(CC(O)=O)N=C2	191,19	C9H9N3O2
Z1508069143	O[C@H]1CC[C@@H](CC1)NC1=NC=C(Cl)C=N1	227,69	C10H14ClN3O
Z1642389730	N#CC1=NC=CC(=C1)C1CC1	144,18	C9H8N2
Z1679724508	CC1CCCC(CN2C=CN=C2C#N)C1	203,29	C12H17N3
Z1610969401	CN1N=CC(CNC2=NN=CS2)=C1C	209,27	C8H11N5S
Z1269638507	NC1=CN(CC2=CC=C(Cl)C=C2)N=C1	207,66	C10H10ClN3
Z1699555768	CN1N=C(C)N=C1NCC1=CC=C(C)O1	206,25	C10H14N4O
Z415735006	C1CC2(CCN1)CCOCC2	155,24	C9H17NO
Z145120146	CCC1=C(SN=N1)C(N)=O	157,19	C5H7N3OS
Z1935338995	CC1(CNC2=CN=CC=C2)COC1	178,24	C10H14N2O
Z1262237209	NCC(O)CN1CCCC1=O	158,20	C7H14N2O2
Z1259339724	NC1(CO)CN2CCC1CC2	156,23	C8H16N2O
Z1245633173	CC(C)OC1=CC(=CC=C1)C(C)N	179,26	C11H17NO
Z1407801656	CC1CCCN(CC2(O)CCC2)C1	183,30	C11H21NO
Z51119298	CCOC1=C(C=CC=C1)C#N	147,18	C9H9NO
Z1695906714	CC1=NN(C=C1C(O)=O)C1CCOCC1	210,23	C10H14N2O3
Z1695729010	NC(C(O)=O)C1=C(Cl)C=CC=C1Cl	220,05	C8H7Cl2NO2
Z1546783878	CC1=C(C)N=C(NC2CCCOC2)N=C1	207,28	C11H17N3O
Z19645843	NC(=O)COC1=C2N=CC=CC2=CC=C1	202,21	C11H10N2O2
Z317545418	CN1C=CC(=CC1=O)C(N)=O	152,15	C7H8N2O2

Z1333173819	OC[C@@H]1C[C@H](NC2=NC=C(Br)C=N2)C=C1	270,13	C10H12BrN3O
Z1675167189	COC1=CC2=C(NC=NC2=O)C=C1	176,18	C9H8N2O2
Z1434233645	CN1N=C(CC2CC2)C=C1N	151,21	C8H13N3
Z2230911781	NC1=NN=C(S1)C1CCOCC1	185,25	C7H11N3OS
Z1983458260	OC[C@@H]1C[C@H](NC2=C(Br)C=NC=C2)C=C1	269,14	C11H13BrN2O
Z2217033058	OC(=O)C1=CC(C#N)=C(N=C1)C(F)(F)F	216,12	C8H3F3N2O2
Z2217033040	CS(=O)(=O)C1=CN=C(N)C=C1	172,20	C6H8N2O2S
Z2217033072	COC1=CC=C(C=C1)C1=CN=C(N)N1C	203,25	C11H13N3O
Z2216711599	CCC1=NC=NC(CC)=C1C(O)=O	180,21	C9H12N2O2
Z1148747945	CC(=O)NC1=C(C)C=NC=C1	150,18	C8H10N2O
Z2527856105	OCC1=NN2C=C(Br)C=NC2=N1	229,04	C6H5BrN4O
Z1509609013	OC1CCCC2=C1C=C(C(O)=O)C(=O)N2	209,20	C10H11NO4
Z2517219338	CC1(C)OCC2CC12C(O)=O	156,18	C8H12O3
Z2512942891	O=C1NCCCC1C1CCCCC1	181,28	C11H19NO
Z401537596	CCC1=NC(C)=C(S1)C(O)=O	171,21	C7H9NO2S
Z164680268	CC1OC2=C(NC1=O)C=CC=C2	163,18	C9H9NO2
Z99600452	CC1=CC(C)=C(C(N)=O)C(=O)N1	166,18	C8H10N2O2
Z29672917	CC1=NN=C(NC(=O)C2=CC=C(Br)O2)S1	288,12	C8H6BrN3O2S
Z50147845	CC1=NN(C2CCS(=O)(=O)C2)C(=O)C1	216,26	C8H12N2O3S
Z168362656	CN1C(C)=CC=C1C(N)=O	138,17	C7H10N2O
Z56921840	OC(=O)CC1=CC2=C(OCCO2)C=C1	194,19	C10H10O4
Z56911972	CC1=C(C)C(OCC(O)=O)=CC=C1	180,20	C10H12O3
Z45527663	CNS(=O)(=O)C1=CC=C(F)C=C1	189,20	C7H8FNO2S
Z56899213	CC1=CC(OCC(O)=O)=C(Cl)C=C1	200,62	C9H9ClO3
Z56891397	CC1=CC=C(S1)C(=O)NCC(O)=O	199,22	C8H9NO3S
Z56891216	O=C(CC(=O)N1CCCC1)N1CCCC1	210,28	C11H18N2O2
Z56870732	O=S(=O)(N1CCNCC1)C1=CC=CC=C1	226,29	C10H14N2O2S
Z56858239	CS(=O)(=O)C1=CC(N)=C(Cl)C=C1	205,66	C7H8ClNO2S
Z56852724	CC12CC3CC(C)(C1)CC(O)(C3)C2	180,29	C12H20O
Z56823032	CC1=C(Cl)C=CC(OCC(O)=O)=C1	200,62	C9H9ClO3
Z56788608	NC1=C(C=CC(=C1)C(F)(F)F)C(O)=O	205,14	C8H6F3NO2
Z56792153	CN1C(C)=CC(C(O)=O)=C1C	153,18	C8H11NO2
Z56754093	[Li+].NC(CC1=CNC2=C1C=CC=C2)C([O-])=O	210,16	C11H11LiN2O2
Z56756861	CC1=NC(C#N)=C(O1)N1CCOCC1	193,21	C9H11N3O2
Z116453054	C=CCNC(=O)C1CCS(=O)(=O)C1	203,26	C8H13NO3S
Z1428158225	OCC1=CN=C(C=C1)N1CCOCC1	194,23	C10H14N2O2
Z1428159815	CC1=CC(C)=C(N1)C(O)=O	139,15	C7H9NO2
Z1421527684	OC(=O)CN1C=C(N=N1)C1=CC=CC=C1	203,20	C10H9N3O2
Z1132044191	CN1CCCC(CCO)C1	143,23	C8H17NO
Z1416203195	CC(C)OC1=NC=CC(=C1)C(O)=O	181,19	C9H11NO3
Z1413780465	O=C1CC2(CCOC2)CN1	141,17	C7H11NO2
Z1407004178	COC1=C(OC)C=C2C=NCCC2=C1	191,23	C11H13NO2
Z1404660409	OC(=O)CC1CCCCO1	144,17	C7H12O3
Z1381287296	CC(C)OC1=C(N)C=C(C=C1)C(O)=O	195,22	C10H13NO3
Z959151850	O[C@H]1CCC2=C1C=CC(Cl)=C2	168,62	C9H9ClO
Z1375682862	CNC1=NN=C(C=C1)C1=CC=CC=C1	185,23	C11H11N3
Z1359429584	CN1C=C(Br)C=C(N)C1=O	203,04	C6H7BrN2O
Z1359429546	OC(CC1CCCCN1)C1=CC=CS1	225,35	C12H19NOS
Z1343518214	COC1CN(C1)S(=O)(=O)N1CCCC1	220,29	C8H16N2O3S
Z237375076	COC1=C(OC)C=C(C=C1)C1CCCN1	207,27	C12H17NO2
Z228584850	C1COC2=C(N1)C=CC=C2	135,17	C8H9NO
Z51119297	COC1=C(C=CC=C1)C#N	133,15	C8H7NO
Z839522560	OC(=O)COC1CCCC1	144,17	C7H12O3
Z57102389	COC1=C(CC(C)N)C=CC=C1	165,24	C10H15NO
Z1217910541	CC(C)(CN)C1=CC=CC=C1	149,24	C10H15N
Z1190318571	CCOC1=CC(CN)=CC=C1	151,21	C9H13NO
Z1342128874	C(CC1=CC=CC=C1)N1C=NC=N1	173,22	C10H11N3
Z54464104	CCOC1=CC(=CC=C1)C#N	147,18	C9H9NO
Z992422546	COC1=CC=C(C=C1)C1CCNC1	177,25	C11H15NO
Z927525866	NCCC(=O)N1CCC2=C(C1)C=CS2	210,30	C10H14N2OS
Z28879653	CC(NC(=O)C1CCCO1)C1=CC=CS1	225,31	C11H15NO2S
Z927854532	NCCC(=O)N1CCCCC1	156,23	C8H16N2O
Z204695214	FC1=C(CN2CCCC2)C=C(C=C1)C#N	204,25	C12H13FN2
Z1348288726	CC1=CN=C(OCC2=NC=CC=C2)C=C1	200,24	C12H12N2O
Z343737504	CC1CN(C(C)CO1)C(=O)C1=CC=CO1	209,25	C11H15NO3
Z265488240	CC1CC(C)CN(CC2=NC(C)=NO2)C1	209,29	C11H19N3O
Z31255398	OCC1CCCN(C1)C1=NC=CC=N1	193,25	C10H15N3O

Z2235681150		142,20	C8H14O2
Z2168515541	CC(C)(O)C1=NC=CC=N1	138,17	C7H10N2O
Z2168542068	CC1=C(N=CC=C1)C(C)(C)CN	164,25	C10H16N2
Z1887715429	OC1CN(CC2=CC=C(Cl)S2)CC1O	233,71	C9H12ClNO2S
Z1796688128	FC1=CC(CN2N=CSC2=O)=CC(F)=C1	228,22	C9H6F2N2OS
Z2017694733	CN1N=CN=C1C1COCCN1	168,20	C7H12N4O
Z254580986	CN(C)C(=O)C1=NOC(=C1)C1=CC=CS1	222,26	C10H10N2O2S
Z907784200	CCNC(=O)C1=CC(F)=C(C=C1)N(C)C	210,25	C11H15FN2O
Z916759702	CC(C)NC(=O)C1=CN=C(C)S1	184,26	C8H12N2OS
Z432808946	OC1CCCN(CC2=CSC(Br)=C2)C1	276,19	C10H14BrNOS
Z420007172	CC(C)NC(=O)C1=C(C)N=CS1	184,26	C8H12N2OS
Z406451888	CNC(=O)CCC1=C(Cl)C(Cl)=CC=C1	232,10	C10H11Cl2NO
Z133751890	CC(NS(C)(=O)=O)C1=C(C)C=CC(C)=C1	227,32	C11H17NO2S
Z875490688	CC1=NC(=CS1)C(=O)NCC1CCC1	210,30	C10H14N2OS
Z54825231	CC(C)NC(=O)C1=C(C)N=C(C)S1	198,28	C9H14N2OS
Z26824802	CCC1=NN=C(NC(=O)C2CC2C)S1	211,28	C9H13N3OS
Z140088802	CNC(=O)CNC(=O)C1=C(C)OC(C)=C1	210,23	C10H14N2O3
Z1272948929	CC1=NC(=CS1)C1=CC(=CC=C1)C(O)=O	219,26	C11H9NO2S
Z1272739859	CC1=C(N)C(=CC(Br)=C1)C(O)=O	230,06	C8H8BrNO2
Z1318356961	CC(C)N1C=C(C#N)C(N)=N1	150,19	C7H10N4
Z1318356924	CCC1=CC(=O)C2=C(N1)C=CC(F)=C2	191,21	C11H10FNO
Z642326330	CCN(C)C(=O)NC1=CC=C(C)C=C1	192,26	C11H16N2O
Z1318147486	COC1=C(N)C=C(C)C=N1	138,17	C7H10N2O
Z1318147477	OC(=O)C1=CC=C(C=C1)N1CCCCC1	205,26	C12H15NO2
Z1318147472	CC(O)CC1=NC2=C(C=CC=C2)C=C1	187,24	C12H13NO
Z1172202072	CC(CO)NC1=C(C=CC=N1)C#N	177,21	C9H11N3O
Z274553582	CC(CC(O)=O)N1C=NC=N1	155,16	C6H9N3O2
Z224209256	CCC(=O)N(C)C1=C(C=CC=C1)C(O)=O	207,23	C11H13NO3
Z227823068	OC(=O)C1=CC(NC2CCCC2)=NC=C1	206,25	C11H14N2O2
Z228588852	COC1=CC2=C(OCC(=C2)C(O)=O)C=C1	206,20	C11H10O4
Z1276745158	OCC1=CC2=C(CCC2)C=C1	148,21	C10H12O
Z2186895525	CCC1=NN(C)C=C1C#N	135,17	C7H9N3
Z1203252645	CN(C(C)=O)C1=CN=CC=C1	150,18	C8H10N2O
Z2692095260	COCC1(C)CNCCO1	145,20	C7H15NO2
Z1610314560	OCCNC1CCOCC1	145,20	C7H15NO2
Z2182115562	CC1(C)CNC1C1=CN=CC=C1	162,24	C10H14N2
Z274787916	CC1CC2=C(O1)C=CC(CO)=C2	164,20	C10H12O2
Z1276496838	CN(C)C1=NC2=C(C=CC=C2)C(CN)=C1	201,27	C12H15N3
Z1103351268	CC(C)(O)CNC1CCOCC1	173,26	C9H19NO2
Z318711254	CN(CCO)C1=NC(C)=CC(C)=N1	181,24	C9H15N3O
Z1413780552	COCC1=NN2CC(N)CCC2=N1	182,23	C8H14N4O
Z1955566419	CC1=CC(=O)C(OCC2=NC=CS2)=CO1	223,25	C10H9NO3S
Z2067000095	N[C@H]1CC[C@@H](CC1)NC1=NC=CC=C1	191,28	C11H17N3
Z2065788064	OC(=O)C1=C2CCCC2=CO1	152,15	C8H8O3
Z2065788326	OCCC1=CN2C=CC=CC2=N1	162,19	C9H10N2O
Z2065464249	COC1=C(Br)C=C(C(O)=O)C(N)=C1	246,06	C8H8BrNO3
Z2065464302	NC1=NN2CCCCC2=C1	137,19	C7H11N3
Z2065464238	OC(=O)C1=CC(NC(=O)C2CC2)=NC=C1	206,20	C10H10N2O3
Z1820272539	CC1=CC(C)=C(O1)C(=O)NC1=CN=NS1	223,25	C9H9N3O2S
Z2050688202	CC(C)C1=C(C=CN1)C(O)=O	153,18	C8H11NO2
Z1896596077	CNC1=NC2=C(C=C(Cl)C=C2)C(=C1)C#N	217,66	C11H8ClN3
Z2048189261	CC(C)C1COCC(=O)N1	143,19	C7H13NO2
Z2044768809	CC1=CC=C(C=C1)N1CCNCC1=O	190,25	C11H14N2O
Z1791178999	O=C(NC1=CN=NS1)C1CCC=CC1	209,27	C9H11N3OS
Z1848954486	N#CC1=CSC(CNC2=NC=CN=C2)=C1	216,26	C10H8N4S
Z1848950527	CC1=CC=C(CNC2=C(F)C=NC=C2)O1	206,22	C11H11FN2O
Z1741966151	CS(=O)(=O)NCC1CCNCC1	192,28	C7H16N2O2S
Z1791179552	O=C(NC1=CN=NS1)C1CCCCC1	211,28	C9H13N3OS
Z1820275462	CC1=C(C=CC=N1)C(=O)NC1=CN=NS1	220,25	C9H8N4OS
Z1271843019	CN(C)C1=CC(C)=C(C=C1)C#N	160,22	C10H12N2
Z1270640441	OC(=O)CCN1C=CC=C1	139,15	C7H9NO2
Z1270446348	CC1=CC=C(O1)C(N)CC(O)=O	169,18	C8H11NO3
Z1270446318	CC(C(O)=O)C1=CC=C(Br)S1	235,10	C7H7BrO2S
Z1270443614	CCC(C)(N)C1=NC(=O)C(C)=C(C)N1	195,27	C10H17N3O
Z1270403496	OC(=O)C1(CC1)C(=O)NC1CC1	169,18	C8H11NO3
Z1270312106	Cl.FC(F)(F)C1=NNC2=C1CNCC2	227,62	C7H9ClF3N3
Z255149896	CC1CC(C)N2N=CC=C2N1	151,21	C8H13N3

Z1268152350	C1CCC(NC1)C1=CNN=C1	151,21	C8H13N3
Z31725250	OCCNC(=O)C1=CC=CC=C1	165,19	C9H11NO2
Z1267881753	OC1CNC(C1)C1CCCCC1	169,27	C10H19NO
Z1262246218	NC1=CN(CC(=O)NC2CCCC2)N=C1	208,27	C10H16N4O
Z1262246220	NC1=CN=C(N=C1)C1CC1	135,17	C7H9N3
Z2658863616	CN1N=CC=C1C(O)C1CCNCC1	195,27	C10H17N3O
Z2630877631	COC1=C2N=C(N)C=CC2=CC=C1	174,20	C10H10N2O
Z2588063784	CC1=NC2=C(N1)C=C(F)C(=C2)C(O)=O	194,17	C9H7FN2O2
Z1741966131	OC(=O)CC1NC(=O)C2=C1C=CC=C2	191,19	C10H9NO3
Z2587654820	OC(=O)C1=CC(=NO1)C1CCNCC1	196,21	C9H12N2O3
Z2583036222	OCC1=CNC(CC2=CC=CC=C2)=N1	188,23	C11H12N2O
Z2234185679	CN1C(=O)N2CCCCC2=C1C(O)=O	196,21	C9H12N2O3
Z2106593034	CCN1C=C(C(O)=O)C(=N1)C1CC1	180,21	C9H12N2O2
Z2106598235	CNC(=O)C1=CC(=NN1C)C(O)=O	183,17	C7H9N3O3
Z2106594244	CCN1N=C(N)C=C1C(O)=O	155,16	C6H9N3O2
Z1741968837	CC1=CC(Cl)=C(C=C1)C(O)=O	170,59	C8H7ClO2
Z2510259497	OC(=O)C1=NC=C(Cl)C(=C1)C1CC1	197,62	C9H8ClNO2
Z2106596435	COC1=C(SC=C1C)C(O)=O	172,20	C7H8O3S
Z2235681686	COC1=C(C#N)C(F)=CC(=C1)C(O)=O	195,15	C9H6FNO3
Z2510259319	CC(N1CCNC1=O)C(O)=O	158,16	C6H10N2O3
Z2510259419	CCC1=NOC=C1C(O)=O	141,13	C6H7NO3
Z2510258108	CC1(CCC2=C1C=CC=C2)C(O)=O	176,22	C11H12O2
Z1741968426	CC1=C(C=CC=C1F)C(O)=O	154,14	C8H7FO2
Z1863941693	OCC1CN(C1)C1=NC=CC=C1	164,21	C9H12N2O
Z2412196601	CC1=CC(C)=C(CCO)N=C1	151,21	C9H13NO
Z2045701767	NC(=O)CN1CCC2=C(C1)C=C(Br)C=C2	269,14	C11H13BrN2O
Z2380439813	OC(=O)C1=CN2CCCCC2=C1	165,19	C9H11NO2
Z2379802743	CN1C=C(NC1=O)C(O)=O	142,11	C5H6N2O3
Z1020680530	CNCC1(O)CCC2=C1C=CC=C2	177,25	C11H15NO
Z183854878	CNC(=O)C1CCC=CC1	139,20	C8H13NO
Z2350906816	CC1=C(C=CC(=C1)C(O)=O)N1C=CC=N1	202,21	C11H10N2O2
Z2327883817	CC1=C(NC(=O)N1)C(O)=O	142,11	C5H6N2O3
Z1509609880	COC1=C(C=C(Br)C=C1)C1(CC1)C(O)=O	271,11	C11H11BrO3
Z2327883913	CN1CC(NC1=O)C(O)=O	144,13	C5H8N2O3
Z1033301244	CC(C)N1C=CN=C1CO	140,19	C7H12N2O
Z86430593	O=C(NC1CCS(=O)(=O)C1)C1=CC=CS1	245,31	C9H11NO3S2
Z1262691705	CC1=C(C=CC(=C1)C#N)N1C=NC=N1	184,20	C10H8N4
Z1262398566	CNC(=O)CN1C=NC(N)=N1	155,16	C5H9N5O
Z1262398499	CC(C(O)=O)C1=C(C=CC=C1)C(F)(F)F	218,18	C10H9F3O2
Z1262398379	CC1=NN2CC(N)CCC2=N1	152,20	C7H12N4
Z2680629344	CC1=NNC(N)=C1C1=CC(C)=CC=C1	187,25	C11H13N3
Z2680629322	COC1=C2N=C(N)C=CC2=C(Cl)C=C1	208,65	C10H9ClN2O
Z2677009987	CC1(C)CC(C(O)=O)C(C)(C)O1	172,22	C9H16O3
Z2679906821	O=C1NCCC2=C1NC=N2	137,14	C6H7N3O
Z2678251339	CC1(CC1)C1=NSC(N)=N1	155,22	C6H9N3S
Z1204332192	CC1=CC(OC(F)F)=C(S1)C(N)=O	207,19	C7H7F2NO2S
Z1889709700	OC(=O)C1=CN(N=C1)C1CC1	152,15	C7H8N2O2
Z1262254315	CCOC1=C2CNCCC2=NC=N1	179,22	C9H13N3O
Z1262254306	CC1=CN2CC(N)CCC2=N1	151,21	C8H13N3
Z1262254299	Cl.Cl.C1CNCC(C1)C1=NC=CS1	241,17	C8H14Cl2N2S
Z1741814701	CCC1CN(CCN)CCO1	158,25	C8H18N2O
Z1824028566	OC[C@@H]1CCCN1CC1=CC=C(Br)O1	260,13	C10H14BrNO2
Z1259339814	OCC1CCC2=C1C=CC=C2	148,21	C10H12O
Z2111525346	O=C1NCCCN1CC1=CC=CC=C1	190,25	C11H14N2O
Z2050688001	COC1=C(Cl)C=C2NC=CC2=C1	181,62	C9H8ClNO
Z2044768696	CN1CC(=O)NC2=C1C=C(Br)C=C2	241,09	C9H9BrN2O
Z2027049497	OC(=O)C1=NNC(CC2=CC=CC=C2)=C1	202,21	C11H10N2O2
Z1896598998	CNC1=C2C=CC=CC2=NC(=N1)C(F)F	209,20	C10H9F2N3
Z1982689193	COC1=C(C=CC=C1)C1=C(N)C=CC=N1	200,24	C12H12N2O
Z1945707487	O=C1CC2CCCN2C2=C(N1)C=CC=C2	202,26	C12H14N2O
Z1881598945	COC1=C(C=C(C=C1)C(O)=O)C1CC1	192,21	C11H12O3
Z1868316875	CN1C(=O)OC2=C1C=CC(CO)=C2	179,18	C9H9NO3
Z1827897989	CC1=C(N)C=C(C=C1)N1C=CC=N1	173,22	C10H11N3
Z1824334032	CCOC1=CC=C(C=C1)C1=CNN=C1N	203,25	C11H13N3O
Z1815157395	CN1CCC2=C1C=CC(=C2)C(O)=O	177,20	C10H11NO2
Z1318171181	CN(CCO)C1=CC=C(F)C=C1	169,20	C9H12FNO
Z1337753275	OCCN1CCCC1C1=CC(F)=CC=C1	209,26	C12H16FNO

Z636447806	ClC1=CC=C(CNC2=NC=CC=C2)S1	224,71	C10H9ClN2S
Z940781276	BrC1=C(CNC2=NC=CC=N2)SC=C1	270,15	C9H8BrN3S
Z153217142	CC(=C)CN1N=NC2=C(C=CC=C2)C1=O	201,23	C11H11N3O
Z458900954	CC(C)S(=O)(=O)C1=CC=C(C=C1)C(N)=O	227,28	C10H13NO3S
Z2583384752	OC1CCOC2(CCOCC2)C1	172,22	C9H16O3
Z1552027336	CC1=CC(=C(CN)C=C1)S(C)(=O)=O	199,27	C9H13NO2S
Z1552027084	CC1=C(C=NN1C1CCOCC1)C(O)=O	210,23	C10H14N2O3
Z1551463531	NC1=NOC(COC2=CC=C(F)C=C2)=N1	209,18	C9H8FN3O2
Z1536942816	OC(=O)CN1C=NC(=N1)C1=CC=CC=C1	203,20	C10H9N3O2
Z220616874	CN1N=C(C)C2=C1SC(=C2)C(O)=O	196,22	C8H8N2O2S
Z1501468148	CC1=NC2=C(NC=C(C#N)C2=O)C=C1	185,19	C10H7N3O
Z1495385160	CN(C)C1=C(N)C=CC(C)=N1	151,21	C8H13N3
Z1203840176	CC(N1CC[C@H](O)C1)C1=CC=C(F)C=C1	209,26	C12H16FNO
Z1465461599	OC1(CCC1)C1=CC=CC=C1	148,21	C10H12O
Z133718524	CS(=O)(=O)N1CCNC(=O)C1	178,21	C5H10N2O3S
Z1463254711	C[C@@H](O)C1=CC(=CC=C1)C#N	147,18	C9H9NO
Z1309454334	N[C@H]1CC[C@@H](CC1)N1CCCS1(=O)=O	218,32	C9H18N2O2S
Z1416203541	CC1CCC(=O)N1CC1=CC=CC=C1	189,26	C12H15NO
Z1255526837	COC1=CC(=CC=C1)C#CCO	162,19	C10H10O2
Z90504166	CC1CCC(CC1)NCC1CCCO1	197,32	C12H23NO
Z1259087027	CC1=CC(O)CC(C)(C)C1	140,23	C9H16O
Z1336428394	CS(=O)(=O)C1CCC(O)C1	164,22	C6H12O3S
Z1587237771	CN(CC1=NC=CN=C1)C1=NC=CC=C1	200,25	C11H12N4
Z52665456	C=CCN1CCN(CC1)C1=NC=CC=N1	204,28	C11H16N4
Z228588982	NCCN1CCCCCC1	142,25	C8H18N2
Z1267882018	CC1=C(CN)OC2=C1C=CC=C2	161,20	C10H11NO
Z57756573	N#CC1=CC=C(C=C1)N1CCCC1	172,23	C11H12N2
Z956073828	CS(=O)(=O)N1CCCC1CN1CCCC1	232,34	C10H20N2O2S
Z1747831414	OCC1CCCN1C(=O)C1=CC(Br)=CS1	290,18	C10H12BrNO2S
Z1924626474	CC1=C(C=C(F)C=C1)C1=NN=C(N)S1	209,24	C9H8FN3S
Z1741972095	COC1=C(Cl)C=CC(N)=C1	157,60	C7H8ClNO
Z1898799515	CC1=NC2=C(N1)C=CC=C2F	150,16	C8H7FN2
Z1889975631	CN1N=C(C(F)F)C2=C1SC(=C2)C(O)=O	232,20	C8H6F2N2O2S
Z744593170	NC1=NN=C(O1)C1CC2=C(O1)C=CC=C2	203,20	C10H9N3O2
Z373775692	CN1C=C(NC(=O)C2=CC=C(Cl)S2)C=N1	241,69	C9H8ClN3OS
Z57728689	OC(=O)CCN1C(=O)SC2=C1C=CC=C2	223,25	C10H9NO3S
Z372500344	CC1=C(SC=C1)C(=O)NC1=CNN=C1	207,25	C9H9N3OS
Z373775856	CN1C=C(NC(=O)C2=CC=CO2)C=N1	191,19	C9H9N3O2
Z57346035	CC1=CC(NC(=O)C2=CC=CS2)=NO1	208,24	C9H8N2O2S
Z85887064	OC(=O)CNC(=O)C1=CC(F)=CC(F)=C1	215,16	C9H7F2NO3
Z32014170	CN(C)C(=O)CNC(=O)C1=CC=C(Br)O1	275,10	C9H11BrN2O3
Z1259335845	OC(=O)C1CC(C1)C1=CC=C(F)C=C1	194,21	C11H11FO2
Z1259273114	NC(C(O)=O)C1=CC(Cl)=CC=C1	185,61	C8H8ClNO2
Z1259162044	Cl.FC1=CC(CN2CCNCC2=O)=CC=C1	244,69	C11H14ClFN2O
Z1259162021	CC1CC(=O)N(C)C2=C(N1)C=CC=C2	190,25	C11H14N2O
Z1250100761	NCC1=CC=C(C=C1)C1=CC=NC=C1	184,24	C12H12N2
Z1245537799	NC1=CC(CN2C=CC=N2)=CC=C1	173,22	C10H11N3
Z1143333160	CC(C)C1=NNC(C(=O)N(C)C)=C1Br	260,14	C9H14BrN3O
Z1171979231	CC1=NN(C=C1)C1=CC(=CC=C1)C(O)=O	202,21	C11H10N2O2
Z68249055	CNC(=O)C1(CC1)C1=CC=C(Cl)C=C1	209,67	C11H12ClNO
Z790348314	FC1=CC2=C(C=C1)N=C(NC2=O)C1CC1	204,20	C11H9FN2O
Z28880832	CC(NC(=O)C1=CC=CO1)C1=CC=CS1	221,27	C11H11NO2S
Z425484910	CC1=CC(=O)NC(=N1)C1=CC(N)=CC=C1	201,23	C11H11N3O
Z1900549400	CC1=CN=C(CNC2=C(F)C=CC=N2)O1	207,21	C10H10FN3O
Z2054373621	CO[C@H]1C[C@H](N(C1)C1CCCC1)C(N)=O	212,29	C11H20N2O2
Z1601232164	FC1=C(F)C=C(NCC2=CSN=N2)C=C1	227,23	C9H7F2N3S
Z871834842	NCC1CCN(CC1)C1CCOCC1	198,31	C11H22N2O
Z2447618800	CCN(C(=O)C(C)N)C1=CC=C(F)C=C1	210,25	C11H15FN2O
Z1667545918	CC(=O)NCC1=CN=CS1	156,20	C6H8N2OS
Z1419216922	CN(C)C(=O)C1=CC2=C(SC=C2)C=C1	205,28	C11H11NOS
Z275170288	CN1C=C(Br)C(=N1)C(O)=O	205,01	C5H5BrN2O2
Z2205958920	CC1=NSC(NC2CC2)=C1C(O)=O	198,24	C8H10N2O2S
Z2197092708	CC1=CC2=C(C=C1)N=C(N)N2	147,18	C8H9N3
Z2188171939	COC1=CC2=C(C=C1)C(N)=NO2	164,16	C8H8N2O2
Z2182115389	NC(=O)[C@H]1CCO[C@@H]1C1CCNCC1	198,27	C10H18N2O2
Z2177031060	FC(F)C1=NC2=C(C=C(Cl)C=C2)C(=O)N1	230,60	C9H5ClF2N2O
Z2177031006	OCC1(CCNC1)C1=CC=CC=C1	177,25	C11H15NO

Z1899665464	ClC1=CC=C(CN2N=CC=CC2=O)C=C1	220,66	C11H9ClN2O
Z2168496123	COCC1(CCOCC1)C(O)=O	174,20	C8H14O4
Z2168282707	CN1N=NC2=C1CC(CC2)C(O)=O	181,20	C8H11N3O2
Z2168282799	OC(C(O)=O)C1=C(F)C=C(Cl)C=C1	204,58	C8H6ClFO3
Z85517151	CC1=CC=C(S1)C(=O)NC1=CC=NC=C1	218,27	C11H10N2OS
Z32024593	CCNC(=O)C1CCCC1	141,21	C8H15NO
Z31479594	CC(C)C(NC(C)=O)C(=O)N1CCCC1	212,29	C11H20N2O2
Z32064488	C=CCNC(=O)C1=CC2=C(C=C1)N=CS2	218,27	C11H10N2OS
Z111479616	OC(=O)CN1C(=O)SC2=C1C=CC=C2	209,22	C9H7NO3S
Z30841099	BrC1=CC(=CN=C1)C(=O)NC1CC1	241,09	C9H9BrN2O
Z57745260	C1CCN(C1)C1=C2N=CNC2=NC=N1	189,22	C9H11N5
Z140637338	COCC(=O)NC1=CC=C(C=C1)C#N	190,20	C10H10N2O2
Z262222238	CC1=C(C(O)=O)C(=NO1)C1=CC=CC=C1	203,20	C11H9NO3
Z57040473	NC1=CC=NN1CC1=CC=CO1	163,18	C8H9N3O
Z1193326527	CN1C=C(CN2C=C(Br)C=N2)C=N1	241,09	C8H9BrN4
Z1396758266	CC1(C)CN(CCS1=O)C1=NC=CN=C1	225,31	C10H15N3OS
Z991187420	Cl.C(N1CCCC1)C1=CN2C=CC=NC2=N1	238,72	C11H15ClN4
Z1169950314	CC1CC1C1=NC(=NO1)C1=CN=CC=C1	201,23	C11H11N3O
Z1245633121	CN1N=CC2=C1CCCC2N	151,21	C8H13N3
Z239081196	CC(N)C(N1CCCC1)C1=CC=CC=C1	204,32	C13H20N2
Z1262252990	CC1=C(C=CC=C1)C(C)(C)CN	163,26	C11H17N
Z1313429074	COC1(C)CC(N)C1(C)C	143,23	C8H17NO
Z1318147319	CC(CN)NC1=CC=CC=C1	150,23	C9H14N2
Z1146147705	CC(=O)NCC1=CC=C(Br)C=C1	228,09	C9H10BrNO
Z1346385372	CC1=CC(=CC=C1)C1=NON=C1N	175,19	C9H9N3O
Z1346385327	OC(=O)CCC1=NC2=C(N1)C=CC=C2F	208,19	C10H9FN2O2
Z740170614	CNS(=O)(=O)C1=CSC(=C1)C(O)=O	221,25	C6H7NO4S2
Z1340444601	CC(=O)N1CCC(C1)C(O)=O	157,17	C7H11NO3
Z1220685184	CN(CC(N)=O)C1=CC(C)=C(Br)C=C1	257,13	C10H13BrN2O
Z1335722261	CCS(=O)(=O)C1=NC2=C(S1)C=C(N)C=C2	242,31	C9H10N2O2S2
Z340632658	CC(=O)NCC1=CC(Br)=CC=C1	228,09	C9H10BrNO
Z1268436514	CC1=CC(=CC(C)=C1OCC(N)=O)C#N	204,23	C11H12N2O2
Z1333043367	CN1N=CC2=C1N=C(C)C=C2C(O)=O	191,19	C9H9N3O2
Z133742684	CC(NS(C)(=O)=O)C1=CC=C(Cl)C=C1	233,71	C9H12ClNO2S
Z1357774220	CC(=O)NC1=NC(C(O)=O)=C(Br)C=C1	259,06	C8H7BrN2O3
Z1354487571	COCC1=NC2=C(O1)C=CC(N)=C2	178,19	C9H10N2O2
Z1815149156	CN(C)C1(CN)CC2=C(C1)C=CC=C2	190,29	C12H18N2
Z1814381728	CC1=CCC(CC1)C(O)=O	140,18	C8H12O2
Z1614607478	OC(=O)C1(CC1)C(=O)NC1=CC=CC=C1	205,21	C11H11NO3
Z31408238	O=C(NCC1=CC=CO1)C1=CC=CS1	207,25	C10H9NO2S
Z25117855	NC(=O)COC1=C(F)C=C(Br)C=C1	248,05	C8H7BrFNO2
Z439257438	CN1N=C(N)C2=C1CCC2	137,19	C7H11N3
Z415636582	CC1=CC=C(C=C1)N1C=C(N=N1)C(O)=O	203,20	C10H9N3O2
Z274797730	BrC1=CN=C(N=C1)N1CCNCC1	243,11	C8H11BrN4
Z32367696	O=C(NC1CC1)C1=CC2=C(C=C1)N=CS2	218,27	C11H10N2OS
Z26283147	NC(=O)COC1=CC(F)=C(F)C=C1	187,15	C8H7F2NO2
Z728935218	NC(=O)C1=CC(Cl)=C(N=C1)N1CCCC1	225,68	C10H12ClN3O
Z401435400	CC(NS(C)(=O)=O)C1=C(F)C=CC(F)=C1	235,25	C9H11F2NO2S
Z594970990	O=C(NCC1=CC=CS1)C1=NSN=C1	225,28	C8H7N3OS2
Z649822028	CC1=C(SC=N1)C(=O)NCC1=NNC=C1	222,27	C9H10N4OS
Z108544658	CN1C=CC(NC(=O)C2=CC=C(Br)O2)=N1	270,09	C9H8BrN3O2
Z332753086	BrC1=CC(=CS1)C(=O)N1CCNC(=O)C1	289,15	C9H9BrN2O2S
Z235785166	OC(=O)CN1CCC2=C(C1)C=CS2	197,25	C9H11NO2S
Z85886787	OC(=O)CNC(=O)C1CCC1	157,17	C7H11NO3
Z992716974	CC(C(O)=O)C1=C(C)ON=C1C	169,18	C8H11NO3
Z275163834	OC(=O)C1=CC=C(CN2C=C(Cl)C=N2)O1	226,62	C9H7ClN2O3
Z992716784	CC1=NN=C2C=CC(=CN12)C(O)=O	177,16	C8H7N3O2
Z29672816	CC1=NN=C(NC(=O)C2=CN=CC=C2)S1	220,25	C9H8N4OS
Z108545268	CN1C=CC(NC(=O)C2=CC=C(Br)S2)=N1	286,15	C9H8BrN3OS
Z680882022	CCN1N=CC2=C1N=C(C)C=C2C(O)=O	205,22	C10H11N3O2
Z57912417	CC1=C(N)C=C(C=C1)C(N)=O	150,18	C8H10N2O
Z193091370	CS(=O)(=O)N1CCCC1C(N)=O	192,23	C6H12N2O3S
Z434632738	CN1C=C(NC(=O)C2=C(Br)C=CS2)C=N1	286,15	C9H8BrN3OS
Z211302168	NC1=C(N=CC=N1)C(=O)N1CCCCC1	206,25	C10H14N4O
Z729725920	CC1=NOC(NC(=O)C2CCC=CC2)=N1	207,23	C10H13N3O2
Z339569398	O=C(NC1CCS(=O)(=O)C1)N1CCCC1	232,30	C9H16N2O3S
Z1783461887	CN1C=NN(CCC2=CC=CS2)C1=O	209,27	C9H11N3OS

Z1694794721	CN1C=NN=C1NC(=O)C1=CC=C(C)O1	206,21	C9H10N4O2
Z1102357527	O=C(NC1CCC(=O)NC1)C1=CSC=N1	225,27	C9H11N3O2S
Z211003642	OC(=O)C1CCN1C(=O)C1=CC=CC=C1	205,21	C11H11NO3
Z383822506	OC1=C(C=CC=N1)C(=O)NCC1CCC1	206,25	C11H14N2O2
Z803129884	CN1C=C(C(O)=O)C(=N1)C1=CN=CC=C1	203,20	C10H9N3O2
Z1262246184	NC1=CC2=C(C=C1)N=C(S2)C1CC1	190,26	C10H10N2S
Z2372280348	CN1C(=O)NCC11CCNCC1	169,23	C8H15N3O
Z29693757	O=C(NCC1=CC=CS1)C1CC1	181,25	C9H11NOS
Z1222331974	CCOC1=NC(=CC=C1)N1CCNCC1	207,28	C11H17N3O
Z839818900	CC(N1CCCC(O)C1)C1=NN=C(C)O1	211,27	C10H17N3O2
Z1891775791	FC1=CN=C(N=C1)N1CCNCC1	182,20	C8H11FN4
Z744843682	OC(=O)C1=CC=CN1CC1=CC=CS1	207,25	C10H9NO2S
Z1139751314	NC1(CC(C1)C1=CC=CC=C1)C(O)=O	191,23	C11H13NO2
Z1626921271	CC1CC(CC(=O)N1)C(O)=O	157,17	C7H11NO3
Z1443588488	CC1=C(N)C=C(CN2CCCC2)C=C1	190,29	C12H18N2
Z1415893841	C1CNC2=C(NC1)N=CC=C2	149,20	C8H11N3
Z1354416054	NC1=CC2=C(C=C1)N=C(O2)C1CC1	174,20	C10H10N2O
Z1272670423	CCC1=NN=C(O1)C1=CC=C(N)C=C1	189,22	C10H11N3O
Z1270207933	O=C1CC2(CN1)CCCCC2	153,23	C9H15NO
Z2227687433	O=C1NC(=NC2=C1CCC2)C1CC1	176,22	C10H12N2O
Z2327131587	CC1C(N)CC2=C1C=CC=C2	147,22	C10H13N
Z199471388	CC(=O)NC1=NN=C(S1)C1=CC=NC=C1	220,25	C9H8N4OS
Z33598681	CC1CCC(CC1)NC(=O)CNC(C)=O	212,29	C11H20N2O2
Z57299924	CC(C)CN1C(CO)=NC2=C1C=CC=C2	204,27	C12H16N2O
Z45527714	CNS(=O)(=O)C1=C(Cl)C=CC=C1	205,66	C7H8ClNO2S
Z220564330	COC1=CC(=CC(OC)=C1Br)C(O)=O	261,07	C9H9BrO4
Z220383490	COC1=CC2=C(C=C1)N=C(C=C2)C(O)=O	203,20	C11H9NO3
Z31212209	CC1=C(C)C2=C(S1)N=CN=C2NCCO	223,29	C10H13N3OS
Z220533930	COC1=CC(C(O)=O)=C(Br)C=C1	231,05	C8H7BrO3
Z217102814	N#CC1=CN=C(C=C1)N1CCOCC1	189,22	C10H11N3O
Z1860991862	CC1=CC(=O)C2=C(N1)C(F)=C(F)C=C2	195,17	C10H7F2NO
Z1860991543	OC(=O)C1COC2=C1C=C(Cl)C=C2	198,60	C9H7ClO3
Z1889902486	OC(=O)C1CCCC2=C1N=CN2	166,18	C8H10N2O2
Z1898825882	CC1=C(Br)C=CC(N)=C1F	204,04	C7H7BrFN
Z1817048016	NC(=O)N1CCCC2(CCCCC2)CC1	210,32	C12H22N2O
Z1868430549	CC1=C(CC(O)=O)C=CS1	156,20	C7H8O2S
Z1868430535	CC(=O)N1CC(C1)C(O)=O	143,14	C6H9NO3
Z1127124482	O[C@@H]1CCN(C1)C1=C2SC=CC2=NC=N1	221,28	C10H11N3OS
Z288780094	CN1C(C)=CC=C1C(=O)NCC1CC1	192,26	C11H16N2O
Z199538186	C1CN(CCN1)C1=CC=NC=C1	163,22	C9H13N3
Z56869298	CN1N(C(=O)C(N)=C1C)C1=CC=CC=C1	203,25	C11H13N3O
Z1166180298	CC1CN(C)CCN1C(=O)C1=CN=NS1	226,30	C9H14N4OS
Z340495298	CC(C)N(C1CC1)C(=O)C1=NOC(C)=C1	208,26	C11H16N2O2
Z279850696	COCC(=O)NC1=C(F)C=CC=C1F	201,17	C9H9F2NO2
Z2216711746	NC1=NC=C(C=C1)C1CCC1	148,21	C9H12N2
Z1245580414	CCOC1=C(N)C=CC=N1	138,17	C7H10N2O
Z1569715489	CC1(CCC(O)=O)CCC(=O)N1	171,20	C8H13NO3
Z1551469282	C1CCC2(C1)COCCN2	141,21	C8H15NO
Z31183312	CN(CC1=CC=CO1)C1=NC=CC=N1	189,22	C10H11N3O
Z1262395901	CC1=NN=C(C=C1)N1CCCC1CO	193,25	C10H15N3O
Z1183468884	OCC1=CN2C=CN=C2C(Cl)=C1	182,61	C8H7ClN2O
Z998307088	CC(C(=O)N(C)C)C1=C(Cl)C=CC=C1Cl	246,13	C11H13Cl2NO
Z319845694	CC(C)C(=O)NC1CCCC2=C1C=CO2	207,27	C12H17NO2
Z369044210	CC1COCCN1CC1=C(Cl)SN=N1	233,71	C8H12ClN3OS
Z1266823292	CC(N)C1=CC(C)=CC(C)=C1	149,24	C10H15N
Z1262254168	CC1=CC(NCC2CCNC2)=NC=C1	191,28	C11H17N3
Z1255406112	N#CC1=CC2=C(OCCO2)N=C1	162,15	C8H6N2O2
Z2507919883	CC1=NC(N)=C(C#N)C(C)=C1	147,18	C8H9N3
Z2492774517	CC(C)CC1=NC(C#N)=C(N)O1	165,20	C8H11N3O
Z2492774641		141,21	C8H15NO
Z2492774577	CC(CC(O)=O)N1C=CC(C)=N1	168,20	C8H12N2O2
Z2492774555	CN(C)C1=C(N)C=C(C)N=N1	152,20	C7H12N4
Z1945710573	CC1(CC1)C1=CSC(=N1)C1=CC=NN1	205,28	C10H11N3S
Z335451356	CC1=CC(=CC=C1)C(N)CC(O)=O	179,22	C10H13NO2
Z2472860086	COC1=C(Cl)C=C(C2CC2)C(N)=C1	197,66	C10H12ClNO
Z2472860018	CC1=C(NC(=O)C2=C1C=CC=C2)C(O)=O	203,20	C11H9NO3
Z2471547970	FC(F)(F)C1=C2OCC(=O)NC2=CC=C1	217,15	C9H6F3NO2

Z2510258174	CC1=C(CC(O)=O)SC(=N1)C1CC1	197,25	C9H11NO2S
Z1982493954	CC1=NC(C2CC2)=C(N)C=N1	149,20	C8H11N3
Z1981870462	CN1C=CC(C(O)=O)=C1C	139,15	C7H9NO2
Z1973465452	NC1=C(CO)C=CC(Br)=C1	202,05	C7H8BrNO
Z914115824	CN(C)C1=CN=C(NC(C)=O)C=C1	179,22	C9H13N3O
Z1948934309	CC(C)C1=NC=C(N)C=N1	137,19	C7H11N3
Z1232688349	N#CC1=C(NC2CCC=CC2)N=CC=N1	200,25	C11H12N4
Z1838000858	CN1N=CN=C1S(=O)(=O)CC1CC1	201,24	C7H11N3O2S
Z1992316392	O=C1CN2CCCC2CN1	140,19	C7H12N2O
Z1259041014	OC(=O)C1COC2=C1C=CC=C2	164,16	C9H8O3
Z1516187297	COC1=C(C#N)C(F)=CC=C1	151,14	C8H6FNO
Z291280256	OC(=O)C1=CC2=C(NC(=O)CCC2)C=C1	205,21	C11H11NO3
Z2692095047	CN1C=CC(=N1)C1=NC(=CS1)C#N	190,22	C8H6N4S
Z2692093609	CN1C=NN=C1C1=CC(Br)=C(C)C=C1	252,12	C10H10BrN3
Z2692093569	OC(=O)C1=C(Br)C(=CC=C1F)C(F)(F)F	287,01	C8H3BrF4O2
Z2692093940	CCC1=C(N)N=CC(Cl)=C1	156,61	C7H9ClN2
Z2092555279	CCC1(O)CCN(C1)C1=NC=C(F)C=C1	210,25	C11H15FN2O
Z1259155983	CN(C)C1=C(N)C=NC=C1	137,19	C7H11N3
Z1259155970	COC1=C(N)C(=CC=N1)N(C)C	167,21	C8H13N3O
Z1820007629	CCC1=NC2=C(C=CC=C2)N1CCN	189,26	C11H15N3
Z98976533	OCCOC1=C(Cl)C=C(C=C1)C#N	197,62	C9H8ClNO2
Z1395852355	CC1=CSC(CN2CCS(=O)CC2)=N1	230,34	C9H14N2OS2
Z1837073461	CCC(C)N1N=CC(C(O)=O)=C1C	182,22	C9H14N2O2
Z1231613812	NC(=O)C1=C(F)C=C(OC(F)F)C=C1F	223,13	C8H5F4NO2
Z1836338758	COC1=C(Br)C=NC(C)=C1	202,05	C7H8BrNO
Z1513499619	O=C1CN(CCN1)C1=C(C=CN=C1)C#N	202,22	C10H10N4O
Z1750884927	CN1C=NN=C1NC(=O)C1=CSN=C1C	223,25	C8H9N5OS
Z1827111442	O=C1C=CNC(CCC2=CC=CC=C2)=N1	200,24	C12H12N2O
Z1824511371	OCC1(CCCC1)C1=CC=CC=C1	176,26	C12H16O
Z1258992644	OC1C(=O)NC2=C1C=CC=C2	149,15	C8H7NO2
Z57900343	COC1=C(C)C=C(C=C1)C#N	147,18	C9H9NO
Z1258992472	OCC1=CC2=C(NN=N2)C=C1	149,15	C7H7N3O
Z1258932991	CCN1N=NC(C(O)=O)=C1CC	169,18	C7H11N3O2
Z1255523429	CC1=C(C(N)=NN1)C1=CC(Br)=CC=C1	252,12	C10H10BrN3
Z1251219820	CC(C)N1C=CC(CO)=N1	140,19	C7H12N2O
Z1250132686		214,62	C10H8ClFO2
Z1250132683	CN(C)C(=O)NC1=CC(=CC=C1)C(O)=O	208,22	C10H12N2O3
Z1250132389	OCC1=CN=C(O1)C1=CC=CS1	181,21	C8H7NO2S
Z1250132330	CC(C)N1C=CC=C1C(O)=O	153,18	C8H11NO2
Z1262237272	CC(CC1=C(C)N(C)N=C1C)C(O)=O	196,25	C10H16N2O2
Z275128648	NC1=NN(CC2=CC=CC=C2)C=C1	173,22	C10H11N3
Z1259341102	CC1=C(OCC(O)=O)C=CC=C1Cl	200,62	C9H9ClO3
Z1259341103	OC(=O)C1=C(F)C=C(OC(F)F)C=C1F	224,11	C8H4F4O3
Z71176798	CC(C)NC1=C(C=CC=N1)C(O)=O	180,21	C9H12N2O2
Z1259339977	CC(C)(C(O)=O)C1=CC=CS1	170,23	C8H10O2S
Z1229798311	CC(=O)NC1=C(C(O)=O)C(F)=CC=C1	197,17	C9H8FNO3
Z1259335904	CN(C)C(=O)C1=C(C=CC=C1)C(O)=O	193,20	C10H11NO3
Z1259162097	CC(O)(CC1=CC=CC=C1)C(O)=O	180,20	C10H12O3
Z235448344	OC1CCOC2=C1C=C(Br)C=C2	229,07	C9H9BrO2
Z1259161906		192,21	C11H12O3
Z228589672	COC1=C(N)C(C(O)=O)=C(Cl)C=C1	201,61	C8H8ClNO3
Z2689031646	OCC1=CC2=C(OCCCO2)C(Br)=C1	259,10	C10H11BrO3
Z2689031546	CC(C)C1=NOC=C1C(O)=O	155,15	C7H9NO3
Z1460222863	CC1=CC(F)=C(NC2CCNC2=O)C=C1	208,24	C11H13FN2O
Z1613884267	CC1=C(C=CO1)C(=O)N1CCC=C(F)C1	209,22	C11H12FNO2
Z2687735108	CNC1=NC(=CC=N1)C1=C(C)N=C(C)S1	220,29	C10H12N4S
Z1432018343	C[C@@H](CO)NC1=C(F)C=C(Cl)C=N1	204,63	C8H10ClFN2O
Z1267881987	CC(CN)OC1=CC=C(C)C=C1	165,24	C10H15NO
Z1545196101	CC1=NNC=C1CNC1=C(F)C=CC=C1	205,24	C11H12FN3
Z1480753089	FC1=C(NCC2=CN=CS2)C=CC=C1	208,25	C10H9FN2S
Z1354370966	FC1=C(N=CC(Cl)=C1)N1CCNC(=O)C1	229,64	C9H9ClFN3O
Z2687203970	O=C1NCCC2=C1C=CS2	153,20	C7H7NOS
Z2687203908		183,25	C10H17NO2
Z2687203705	NC1=C(Cl)C=C2CCCC2=C1	167,64	C9H10ClN
Z1203191681	CC(=O)NCC1=NC=CN1	139,16	C6H9N3O
Z1481102552	FC1=CN=C(NCC2=CC=CS2)N=C1	209,24	C9H8FN3S
Z1840519037	CNS(=O)(=O)C1=C(F)C=C(OC)C=C1	219,23	C8H10FNO3S

Z192952702	CN(C)S(=O)(=O)C1=CC(F)=CC(F)=C1	221,22	C8H9F2NO2S
Z923951640	CS(=O)(=O)NCC1=C(F)C=C(F)C=C1	221,22	C8H9F2NO2S
Z1171978800	OC(=O)C1=C(OC(F)F)C=CS1	194,15	C6H4F2O3S
Z1171978897	CCC1=NC=C(S1)C(O)=O	157,19	C6H7NO2S
Z118690766	O=C(NC1CCS(=O)(=O)C1)C1CC1	203,26	C8H13NO3S
Z135505014	CNC(=O)C1C2CC3CC(C2)CC1C3	193,29	C12H19NO
Z1167043933	COC1=CC(NC2CCNCC2)=CC=C1	206,29	C12H18N2O
Z227840048	CN(C)S(=O)(=O)C1=C(SC=C1)C(O)=O	235,27	C7H9NO4S2
Z899154150	NC(=O)CN1C=CC(=O)C2=C1C=CC=C2	202,21	C11H10N2O2
Z381479818	O=C(NC1=CC=CC=C1)C1COCCO1	207,23	C11H13NO3
Z126992654	CN(CCO)C1=C2C=CC=CC2=NC=N1	203,25	C11H13N3O
Z62455180	C=CCNC1=C2C=CC=CC2=NC=N1	185,23	C11H11N3
Z139937454	CCNC(=O)C1=CN=C2SC=CN2C1=O	223,25	C9H9N3O2S
Z1160899478	OC1CC(C(O)=O)C2=C1C=CC=C2	178,19	C10H10O3
Z1768428950	COC(C)C1=NC(=CS1)C(O)=O	187,21	C7H9NO3S
Z1768428787	OC(=O)C1CC11CCS(=O)(=O)CC1	204,24	C8H12O4S
Z1768160457	CC1=C(C)N2C(S1)=NC=C(C(O)=O)C2=O	224,23	C9H8N2O3S
Z1768160454	OC(=O)CCC1=CC2=C(CCC2)C=C1	190,24	C12H14O2
Z1762658220	COC1=C(N)C(C(O)=O)=C(Br)C=C1	246,06	C8H8BrNO3
Z1742054999	CN(C)C1=NOC(=N1)C1CCNCC1	196,25	C9H16N4O
Z1603605397	CC1=C(SC=C1)C(=O)NC1(CC1)C(O)=O	225,26	C10H11NO3S
Z1737940684	CC(N)(C(O)=O)C1=CC(F)=CC=C1	183,18	C9H10FNO2
Z1718361702	CC(C)CCN1N=CC(C#N)=C1N	178,24	C9H14N4
Z26794305	CC(=O)NC1=CC2=C(NC(=O)N2)C=C1	191,19	C9H9N3O2
Z1499061380	CN1CCCC(C)(CO)C1	143,23	C8H17NO
Z2326079238	CC1CCC(C)(C)N1C(=O)COC(F)F	221,25	C10H17F2NO2
Z85926259	CC(N1N=CC=C1N)C1=CC=CS1	193,27	C9H11N3S
Z1259087078	CC(CN)CN1C=CC=N1	139,20	C7H13N3
Z2234185640	CC(C)(CC1CCCNC1)C(N)=O	184,28	C10H20N2O
Z1340493763	COCC(C)(N)C1=CC=CC=C1	165,24	C10H15NO
Z2358147253	FC1=CNC(=C1)C(=O)NCC1CC11CC1	208,24	C11H13FN2O
Z1522339024	CNC(=O)CN1CCC(=CC1)C(F)(F)F	222,21	C9H13F3N2O
Z2111525761	OCCC1=NN=C(O1)C1=CC=CC=C1	190,20	C10H10N2O2
Z2111525135	NC1=NC2=C(CCCC2)N=C1	149,20	C8H11N3
Z365255930	CN1CC(CC1=O)C(N)=O	142,16	C6H10N2O2
Z2092370954	CCN(C)C(=O)C1=CC(N)=CC=C1	178,24	C10H14N2O
Z1203329531	CC(=O)NCC1(O)CCCC1	157,21	C8H15NO2
Z2034643365	CC#CCN1C=NC(=N1)C1=NC=CS1	204,25	C9H8N4S
Z1431002634	NC1(CCO)CCOCC1	145,20	C7H15NO2
Z44548922	CC(N)C(=O)N1CCCCC1	156,23	C8H16N2O
Z1258578160	CNC1=NNC(=C1)C(C)C	139,20	C7H13N3
Z1273312142	CCOC1=C(C=CC=C1F)C(=O)N(C)C	211,24	C11H14FNO2
Z280816320	CC(C1CC1)N(C)C(=O)C1=CC(C)=NO1	208,26	C11H16N2O2
Z1406872867	CC1CCCC(CCO)C1	142,24	C9H18O
Z1696946116	CC1CCOC1C(=O)NC1=NC=CN=C1	207,23	C10H13N3O2
Z1723549409	CC(C)C1CCCC(N)C1	141,26	C9H19N
Z1723549378	CC(C)C1(C)CC1C(O)=O	142,20	C8H14O2
Z1238477874	C1CCC(NC1)C1=CC=CO1	151,21	C9H13NO
Z193075552	CC(N(C)S(C)(=O)=O)C1=CC=C(F)C=C1	231,29	C10H14FNO2S
Z1171716847	NCC1CCCN(CC2=CN=CC=C2)C1	205,31	C12H19N3
Z56346996	CCN1N=NC2=C1C=CC(=C2)C(O)=O	191,19	C9H9N3O2
Z103726028	CN(C)CC1=NN=C(O1)C1=CC=C(Br)O1	272,10	C9H10BrN3O2
Z31195264	CC(NC1=NC=CC=N1)C1=CC=CC=C1	199,26	C12H13N3
Z234896505	CC1=C(C)C=C(C=C1)C1=NC(N)=NN=C1	200,25	C11H12N4
Z234896483	CCC(N)(C(O)=O)C1=CC(Cl)=CC=C1	213,66	C10H12ClNO2
Z102895082	COC1=C(OC)C2=C(C=C1)C=NNC2=O	206,20	C10H10N2O3
Z234895477	CN(C)C1=CC(=NC=C1)C(O)=O	166,18	C8H10N2O2
Z26824643	CCC1=NN=C(NC(=O)C2=CC=CO2)S1	223,25	C9H9N3O2S
Z16114793	CNC(=O)COC1=C(Cl)C=CC(C)=C1	213,66	C10H12ClNO2
Z18391402	NC(=O)COC1=C(Cl)C=C(F)C=C1	203,60	C8H7ClFNO2
Z234853588	CCOCC1=C(N)N=C(C)N=C1	167,21	C8H13N3O
Z369263636	CC(NS(C)(=O)=O)C1=C(C)N(C)N=C1	217,29	C8H15N3O2S
Z1483951257	CC(C)N1CCCCC1CN	156,27	C9H20N2
Z90507087	C(NC1CCCCCC1)C1=NC=CC=C1	204,32	C13H20N2
Z435369108	CN(CC1=CC(Br)=CS1)C(=O)C1CC1	274,18	C10H12BrNOS
Z314446712	CCN(C1CC1)C(=O)C1=CC=CS1	195,28	C10H13NOS
Z355728146	CCNC(=O)CN1CC(C)OCC1C	200,28	C10H20N2O2

Z19755216	CN(C)C(=O)COC1=CC(F)=CC=C1	197,21	C10H12FNO2
Z647442662	CC1=NOC(CN2CCCCC2CO)=N1	211,27	C10H17N3O2
Z104652094	CC1=CSC(=O)N1CCN1CCOCC1	228,31	C10H16N2O2S
Z2327226256	COC1=C(OC)C(Cl)=C(C=C1)C#N	197,62	C9H8ClNO2
Z1348316421	N#CC1=NC=C(C=C1)N1CCOCC1	189,22	C10H11N3O
Z2326990066	COC1=C(N)N=CC(Cl)=C1	158,59	C6H7ClN2O
Z2309759377	NC1=C(Br)C=CC(CO)=C1	202,05	C7H8BrNO
Z2306627089	FC1=C(F)C2=C(NCCO2)C=C1	171,15	C8H7F2NO
Z2301499876	COC1=CC(OC)=C(Cl)C(N)=C1	187,62	C8H10ClNO2
Z2301499896	CC1=CC2=C(C=CN=C2N)C=C1	158,20	C10H10N2
Z1741966337	CN1C=C(N)C(=N1)C(N)=O	140,15	C5H8N4O
Z2106597800	CC1=CN(N=C1)C1=CC=C(C=C1)C#N	183,21	C11H9N3
Z2301438405	CCC1=CC(=O)NN=C1C1=CC=CC=C1	200,24	C12H12N2O
Z728940672	FC1=CC2=C(C=C1)N=CN=C2NC1CC1	203,22	C11H10FN3
Z2293608204	CC1=NN(C=C1)C1=CC=C(C=C1)C#N	183,21	C11H9N3
Z959330822	CC(CC1=CN(C)N=C1)C(O)=O	168,20	C8H12N2O2
Z680889192	CN1NC(=O)C2=C1N=CC(Br)=C2	228,05	C7H6BrN3O
Z2293605544	OC(=O)C1=CC2=C(CCOC2)N1	167,16	C8H9NO3
Z1267881663	O=C1CCCN1CC1=CC=C(C=C1)C#N	200,24	C12H12N2O
Z1266933854	CC(N)C(O)C1=CC=C(F)C=C1	169,20	C9H12FNO
Z1266854954	CC1=C(C)C2=C(NN=C2N)N=N1	163,18	C7H9N5
Z1266854829	OC(C1CCNCC1)C1=CC=C(F)C=C1	209,26	C12H16FNO
Z228585842	CN1CCN(CC1)C(=O)CO	158,20	C7H14N2O2
Z1263820262	CC(C)N1C=NC2=C1C=CC(=C2)C(O)=O	204,23	C11H12N2O2
Z1263602424	COC1=CC(C2=NN=C(N)O2)=C(Br)C=C1	270,09	C9H8BrN3O2
Z1263602410	CN1C(=O)OC2=C1C=CC(N)=C2	164,16	C8H8N2O2
Z1741962470	CN1N=C(C=C1N)C1CC1	137,19	C7H11N3
Z367452370	Cl.CN(C)C(=O)C1CCNCC1	192,69	C8H17ClN2O
Z1815733624	CC1=NC(=NC=C1)N1CC(C)(O)C1	179,22	C9H13N3O
Z1956351798	CC(O)CNC1CCCC2=NN(C)C=C12	209,29	C11H19N3O
Z56928625	CNCC1=CC=C(OC)C=C1	151,21	C9H13NO
Z1428018544	CN(C1CCCC1)C1=C(N)C=CC=C1	190,29	C12H18N2
Z57039604	NCC(N1CCCC1)C1=C(Cl)C=CC=C1	224,73	C12H17ClN2
Z1442183892	CC(O)C1=C(C=CC=C1F)N1C=CC=N1	206,22	C11H11FN2O
Z2234631193	CN(C)S(=O)(=O)C1=C(C=CC=C1)C(O)=O	229,25	C9H11NO4S
Z1974660264	CC(=O)N[C@@H]1C[C@H]1C1=CC(F)=C(Cl)C=C1	227,66	C11H11ClFNO
Z1619978933	CC1=NN=C(CN2CCC=C(F)C2)S1	213,27	C9H12FN3S
Z1783230611	CNC(=O)C1(CC2=CC=C(F)C=C2)CC1	207,25	C12H14FNO
Z1251753693	CNC(=O)C1CC1C1=C(Cl)C=CC=C1F	227,66	C11H11ClFNO
Z1619985466	FC1=CCCN(CCN2C=CN=C2)C1	195,24	C10H14FN3
Z2034475738	CN(CC(C)(C)O)C1=NC=C(F)C=C1	198,24	C10H15FN2O
Z1900627254	FC1=CCCN(C1)C1=NC=CN=C1	179,20	C9H10FN3
Z1689442171	FC1=CCCN(C1)C(=O)N1CCOCC1	214,24	C10H15FN2O2
Z1481145839	FC1=CN=C(N=C1)N1CC2CCCC2C1	207,25	C11H14FN3
Z1395929370	OCCN1CCOC2=C1C=C(F)C=C2	197,21	C10H12FNO2
Z758198920	CC(=O)N1CCOC2=C(C1)C=CC=C2	191,23	C11H13NO2
Z1741980282	COC1=C(CO)C=CC=N1	139,15	C7H9NO2
Z1824334043	CNCC1=C(N)C=CC(Br)=C1	215,09	C8H11BrN2
Z1759803490	CCN1C=NN=C1C1CN(CC)CCO1	210,28	C10H18N4O
Z2240988377	CCN1CCN(CC(C)(C)N)CC1	185,32	C10H23N3
Z1741815708	CC1(C)CCCN1CCN	142,25	C8H18N2
Z19025264	NC(=O)CN1C(=O)C=NC2=C1C=CC=C2	203,20	C10H9N3O2
Z107728826	OCC(=O)NC1=CC2=C(OCCO2)C=C1	209,20	C10H11NO4
Z26824739	CCC1=NN=C(NC(=O)C2CCC2)S1	211,28	C9H13N3OS
Z104341242	OC(=O)C1CCS(=O)(=O)C1	164,18	C5H8O4S
Z99601600	CC1=CC(C)=C(NC(=O)CO)C(C)=C1	193,25	C11H15NO2
Z234893819	NC1=C2C(=O)NCC2=CC=C1	148,17	C8H8N2O
Z90663557	NC(=O)CC1=CN2C=CC=CC2=N1	175,19	C9H9N3O
Z85934875	CNC(=O)CN1CCNCC1	157,22	C7H15N3O
Z89283364	OC(=O)C1=CC2=C(OCCCO2)C=C1	194,19	C10H10O4
Z85917566	O=C1CCC2=C(N1)C=CC=C2	147,18	C9H9NO
Z57984669	CC1=NC(=CS1)C1=CC(N)=CC=C1	190,26	C10H10N2S
Z57056172	CC1=CC=C(CC(=O)N2CCCC2)C=C1	203,29	C13H17NO
Z823287468	OC(=O)CC1=CSC(=N1)C1=CC=C(Cl)S1	259,72	C9H6ClNO2S2
Z815264064	CCOC1=NC(C(O)=O)=C(Cl)C=C1	201,61	C8H8ClNO3
Z228588764	COC1=C2OCC(=CC2=CC=C1)C(O)=O	206,20	C11H10O4
Z17834203	NC(=O)COC1=CC(Cl)=CC(Cl)=C1	220,05	C8H7Cl2NO2

Z31490566	O=C(NCC1CCCO1)C1=CC=CS1	211,28	C10H13NO2S
Z744843398	CC1=CN(CC(O)=O)N=C1	140,14	C6H8N2O2
Z85893775	CC(C)C(=O)N1CCCC(C1)C(O)=O	199,25	C10H17NO3
Z85525304	CC1=CC=C(S1)C(=O)NC1=NC=CC=C1	218,27	C11H10N2OS
Z354263674	CC1=CN2N=C(NCC=C)SC2=N1	194,26	C8H10N4S
Z57000601	CC1=C2C=CC=CC2=NC(N)=C1C#N	183,21	C11H9N3
Z235352163	OC(=O)C1=CC=CN1CC1=CC=CC=C1	201,23	C12H11NO2
Z90165620	CC(NC1=NC=CC=C1)C1=CC=CO1	188,23	C11H12N2O
Z85886770	OC(=O)CNC(=O)C1=C(F)C=C(F)C=C1	215,16	C9H7F2NO3
Z235341985	CC(C)CN1N=CC2=C1NC(=O)C=C2C	205,26	C11H15N3O
Z169512032	CC(=O)NC(C1CCCC1)C(O)=O	185,22	C9H15NO3
Z223814814	CN1N=CC2=C1N=CN=C2N1CCCC1	203,25	C10H13N5
Z381061402	COC1=CC(N)=C(C=C1)C(O)=O	167,16	C8H9NO3
Z144495968	CN(C)C(=O)C1=CN=C2SC=CN2C1=O	223,25	C9H9N3O2S
Z372853960	NC(=O)C1(N)CCCCCC1	156,23	C8H16N2O
Z367678518	OC(=O)CNC(=O)C1=CC=NC=C1	180,16	C8H8N2O3
Z367452204	OC(=O)CCC1=CC(F)=C(F)C=C1	186,16	C9H8F2O2
Z367451024	CN(C)C1=NC=C(C=C1)C(O)=O	166,18	C8H10N2O2
Z183417690	CC1=NC(C)=C(S1)C(=O)NC1CC1	196,27	C9H12N2OS
Z26251905	CC1=C(C)C=C2N(CC(N)=O)C=NC2=C1	203,25	C11H13N3O
Z52305549	CNC(=O)CNC(=O)C1=CC(Br)=CN=C1	272,10	C9H10BrN3O2
Z320136522	COC1=C(CO)C=C(Br)C=C1CO	247,09	C9H11BrO3
Z1271630862	CCOC1=C2C=CNC2=CC=C1	161,20	C10H11NO
Z2466617844	CC1=NC2=C(C=CC=C2)C11CCNCC1	200,29	C13H16N2
Z2106596953	CCN1N=CC2=C1C(=O)NC=N2	164,17	C7H8N4O
Z2429425516	CCC1=CC2=C(O1)C=CNC2=O	163,18	C9H9NO2
Z1720581929	NCC1(O)CCC2=C1C=CC(F)=C2	181,21	C10H12FNO
Z1272272409	C[C@H](NC1=NC(C)=NS1)C1=NC=CC=C1	220,29	C10H12N4S
Z1263811594	CC(CN)OC1=C(C)C=CC=C1	165,24	C10H15NO
Z1263811616	CC(C)(CN)C1=CC=C(F)C=C1	167,23	C10H14FN
Z1266823369	NC1CCC2=NC=NN2C1	138,17	C6H10N4
Z1544648160	CN(C)C(=O)C1CCCC1C(=O)N(C)C	212,29	C11H20N2O2
Z1222278743	CC1CCN(CC1)C1=NC=C(CN)C=C1	205,31	C12H19N3
Z1359429502	CC1C(O)CC2=C1C=CC=C2	148,21	C10H12O
Z1416200837	CC(N)C1=NOC(=N1)C1=CC=CC=C1	189,22	C10H11N3O
Z1250132764	CC1=C(C)N=C(S1)C(C)(C)N	170,27	C8H14N2S
Z1258578133	CCC(C)C1=NOC(=C1)C(O)=O	169,18	C8H11NO3
Z1350579414	CCC1=NC(=NO1)C1CCCN1	167,21	C8H13N3O
Z1340493791	CCC1CNCCN1CC(C)O	172,27	C9H20N2O
Z228584490	CC(C)N(C)C(=O)C1=CC=C(CN)C=C1	206,29	C12H18N2O
Z1262237397	NCC1CCN(C1)C1=CC=C(Cl)C=C1	210,71	C11H15ClN2
Z1428019337	CC1CN(CCN1)C(C)=O	142,20	C7H14N2O
Z139565496	CN1CCCCC1CCO	143,23	C8H17NO
Z57162030	OC(=O)CC1CCS(=O)(=O)C1	178,20	C6H10O4S
Z228474600	CC(N1C(=O)OC2=C1C=CC=C2)C(O)=O	207,19	C10H9NO4
Z210803656	CN1N=C(C)C(CCC(O)=O)=C1C	182,22	C9H14N2O2
Z89264974	COC1=CC(F)=C(C=C1)C(O)=O	170,14	C8H7FO3
Z1020680330	NCC1(O)CC2=C(C1)C=CC=C2	163,22	C10H13NO
Z1250080911	CC1=NN=C(N1)C1=CC(=CC=C1)C#N	184,20	C10H8N4
Z1222331846	CNC(C(O)=O)C1=C(F)C=CC(F)=C1	201,17	C9H9F2NO2
Z1416171278	OC(=O)CC1CCC(=O)NC1	157,17	C7H11NO3
Z2218585938	CC(C)OC1=C(N)C=C(C)C=N1	166,22	C9H14N2O
Z241287306	CN(C1CCCC1)C(=O)C1CCC1	181,28	C11H19NO
Z1269702201	CC(CN)OC1=CC=C(Cl)C=C1	185,65	C9H12ClNO
Z1269702301	CN(C)C1CCC2=C(C=CC=C2)C1N	190,29	C12H18N2
Z1267882047	CC(N)C(C)OC1=C(F)C=CC=C1	183,23	C10H14FNO
Z1267773513	CNC1CCCC1S(C)(=O)=O	177,26	C7H15NO2S
Z2216303521	NCC(CO)C1CCCCC1	157,26	C9H19NO
Z1273048529	CC1=CC=C(C=C1)C1OCCCC1CN	205,30	C13H19NO
Z1613492358	CC(O)CCN1C=CC=N1	140,19	C7H12N2O
Z137402504	CC1=CC(CNC2=C(Cl)C=CC=C2)=NO1	222,67	C11H11ClN2O
Z85893824	CC(C)CC(=O)N1CCCC(C1)C(O)=O	213,28	C11H19NO3
Z137654490	CCC1CCCCN1CC1=C(Cl)SN=N1	245,77	C10H16ClN3S
Z2242946632	NC1=CC2=C(CNC2)C=C1	134,18	C8H10N2
Z2235831699	CC(C)NC(=O)C1=NN(C)C=C1N	182,23	C8H14N4O
Z1739363149	CS(=O)(=O)N1CCOC(C1)C1CC1	205,27	C8H15NO3S
Z90504557	OCCNC1CCCCC1	143,23	C8H17NO

Z2238508528	COC1=NC2=C(CCN2)C=C1	150,18	C8H10N2O
Z1509441055	CN(C)C1=C(Br)C=NC(N)=C1	216,08	C7H10BrN3
Z2236735311	NC1=NC(=CN=N1)C(F)(F)F	164,09	C4H3F3N4
Z2236735605	CN1NC(=CC1=O)C(O)=O	142,11	C5H6N2O3
Z2067123286	ClC1=C(CN2C=CSC2=O)SC=C1	231,71	C8H6ClNOS2
Z1255361336	O=C1CCC2=C(CCN1)C=CC=C2	175,23	C11H13NO
Z1198163513	OC(=O)CCC1CCC(=O)N1	157,17	C7H11NO3
Z2235810166	CN1C(=CC=C1C(O)=O)C#N	150,14	C7H6N2O2
Z2235810123	CC1=NC2=C(CCC(O)C2)N1	152,20	C8H12N2O
Z2235810223		194,19	C10H10O4
Z2235810203	CC1=C2CCNC2=NC=C1	134,18	C8H10N2
Z1891775483	CC1(C)CNCC(C)(C)O1	143,23	C8H17NO
Z1365534373	CN1C=CC(C)=C(N)C1=O	138,17	C7H10N2O
Z2255113012	OC(=O)C1CN(C1)C(=O)C1CCCCC1	211,26	C11H17NO3
Z2004563941	CC(O)C1=CN(N=C1)C1CCCC1	180,25	C10H16N2O
Z1076481138	C(NC1CCCCC1)C1=NC=CN1	179,27	C10H17N3
Z426210734	N#CC1=CC=C(OCC2CCOC2)C=C1	203,24	C12H13NO2
Z1250089987	OCCN1N=CC2=C1C=CC=C2	162,19	C9H10N2O
Z1245735303	CC(O)C1=C(C)N=C(C)S1	157,23	C7H11NOS
Z1245646677	OCC1=CN(CC2=CC=C(Br)C=C2)N=C1	267,13	C11H11BrN2O
Z100642432	CN(C)C(=O)C1=C(Br)C=C(F)C=C1	246,08	C9H9BrFNO
Z247511056	CC(=O)N(CC(N)=O)C1=CC=CC=C1	192,22	C10H12N2O2
Z104647358	NC(=O)CNC1=CC=C(Cl)C=C1	184,62	C8H9ClN2O
Z104549838	CC1=CC(NCC(N)=O)=C(C)C=C1	178,24	C10H14N2O
Z1245537957	NCCNC1=C2C=CSC2=NC=N1	194,26	C8H10N4S
Z1983132602	CS(=O)(=O)C1(CO)CCC1	164,22	C6H12O3S
Z1101755952	CC1CCCN(CC1)C(=O)C1=CSC=N1	224,32	C11H16N2OS
Z133729826	CC(NS(C)(=O)=O)C1=CC(Cl)=CC=C1	233,71	C9H12ClNO2S
Z1956875677	NCC(CO)C1CCOC1	145,20	C7H15NO2
Z1262254296	CC(N)C1=CC(NC(C)=O)=CC=C1	178,24	C10H14N2O
Z1263529802	COC1=C(C(C)N)C(F)=CC=C1	169,20	C9H12FNO
Z1295585445	CCC(N)C1CCOCC1	143,23	C8H17NO
Z1362754412	COC(C)C1=NC(CN)=CS1	172,25	C7H12N2OS
Z197266168	OCCN1CCCCCC1	143,23	C8H17NO
Z1838995460	COC1=CC(CN2C=CC=N2)=C(Cl)C=C1	222,67	C11H11ClN2O
Z1655141307	CN1C(C)=NC(C(N)=O)=C1N	154,17	C6H10N4O
Z1649893034	CCC1=NC(=CO1)C(O)=O	141,13	C6H7NO3
Z1606822433	CC1=NC(CNC2=NC(C)=NO2)=CS1	210,26	C8H10N4OS
Z1705831819	CC1=NC2=NC(CC(N)=O)=CN2C=C1	190,21	C9H10N4O
Z1661748238	CC(C)NC(=O)CN1N=CSC1=O	201,24	C7H11N3O2S
Z1698549667	NC(CC(O)=O)C1=C(F)C=C(F)C=C1	201,17	C9H9F2NO2
Z1696824152	NC1=NNC(=O)C1C1=CC=CC=C1	175,19	C9H9N3O
Z1696824078	OC(=O)C1=C2N=C(NC2=CC=C1)C1CC1	202,21	C11H10N2O2
Z2004547594	CC1(C)CC(=O)NC2=C1C=CC=C2	175,23	C11H13NO
Z1741967102	ClC1=CC(=CC=C1)C1CNCCN1	196,68	C10H13ClN2
Z1998637006	OCC1CCCC2=C1N=CC=C2	163,22	C10H13NO
Z1741977642	CCC1=CC=C(C=C1)C1CNCCN1	190,29	C12H18N2
Z2028214420	CC1(C)NC(=O)CCC1N	142,20	C7H14N2O
Z2027896796	CC(C)CN1C(N)=NC=C1C(F)(F)F	207,20	C8H12F3N3
Z1896595030	CNC1=C2C=C(OC)C=CC2=NC(C)=N1	203,25	C11H13N3O
Z1820276480	CN1C=C(Cl)C=C1C(=O)NC1=CN=NS1	242,68	C8H7ClN4OS
Z1623872049	CC1=C(C(O)=O)C(=O)NC=C1	153,14	C7H7NO3
Z319550626	CN(C)S(=O)(=O)C1=CN(C)C(=C1)C(O)=O	232,25	C8H12N2O4S
Z1614605132	O=C1NCCN2N=CC=C12	137,14	C6H7N3O
Z1165945108	CC(O)CN(C)C1=NC=C(Br)C=N1	246,11	C8H12BrN3O
Z1582263013	CCC1(O)CCC(N)CC1	143,23	C8H17NO
Z1575311908	CCNC1=NC=C(C=C1)C(O)=O	166,18	C8H10N2O2
Z1575082073	CC1=NC2=C(N1)N=CC=C2C	147,18	C8H9N3
Z1569708695	CS(=O)(=O)C1=CSC(=C1)C(O)=O	206,23	C6H6O4S2
Z1552026926	CCC1(CO)CCNCC1	143,23	C8H17NO
Z145985740	CC1=NOC(=C1)C(=O)NC1=CN=CC=C1	203,20	C10H9N3O2
Z1333173490	OC[C@@H]1C[C@H](NC2=NC=C(Cl)C=N2)C=C1	225,68	C10H12ClN3O
Z32016410	CNC(=O)C1=CC=CC=C1	135,17	C8H9NO
Z94598702	NC(C1=CC=CS1)C1=CC=C(F)C=C1	207,27	C11H10FNS
Z1238477848	NCC1=C(F)C=CC(Br)=C1	204,04	C7H7BrFN
Z2028236605	C1CC2(CN1)CCCOC2	141,21	C8H15NO
Z1924550188	NCC1=C(F)C=C(C=C1)C1=CC=CO1	191,21	C11H10FNO

Z1266823177	CC(C)C(N)C1=NN=C2CCCN12	180,26	C9H16N4
Z1217910552	COC1=CC(OC)=CC(CN)=C1	167,21	C9H13NO2
Z1444766645	CC(=O)N1CCOCC1C(O)=O	173,17	C7H11NO4
Z1267773518	CC1=C(C=CC=C1)C(N)C1CC1	161,25	C11H15N
Z1262252887	CC(N)C1CCN(C)CC1	142,25	C8H18N2
Z54633859	CN(CCO)C1=NC=CC=C1	152,20	C8H12N2O
Z445904810	COCCNC(=O)N1CCC1	158,20	C7H14N2O2
Z993991216	CS(=O)(=O)CC1=CC(CN)=CC=C1	199,27	C9H13NO2S
Z228474642	NCCC1=CN(N=C1)C1=CC=CC=C1	187,25	C11H13N3
Z1695906872	CCC1=C(C=C(C=C1)C(O)=O)C#N	175,19	C10H9NO2
Z1695709455	CS(=O)(=O)C1=CC(N)=C(F)C=C1	189,20	C7H8FNO2S
Z33545997	NC(=O)CN1C(=O)COC2=C1C=CC=C2	206,20	C10H10N2O3
Z2235681451	CCN1C(CO)=NC2=C1C=CC(OC)=C2	206,25	C11H14N2O2
Z2235681460	OCC1(CNC(=O)C1)C1=NC=CC=C1	192,22	C10H12N2O2
Z2235386824	CCN1N=NC(C)=C1CO	141,17	C6H11N3O
Z1562963009	C(N1CCN(CC1)C1CC1)C1=CSN=N1	224,33	C10H16N4S
Z2235330282	CC1CC2=C(N1)C=CN=C2	134,18	C8H10N2
Z2001240785	CC(=O)NC1=C(C)N=C2C=CC=CC2=C1	200,24	C12H12N2O
Z2218555527	CC1=CC(=O)C(C)=C(C)N1	137,18	C8H11NO
Z2574360326	CN1C=C(CCC(O)=O)N=N1	155,16	C6H9N3O2
Z2568758040	OC(=O)C1=CC=C(NC2=NCCC2)C=C1	204,23	C11H12N2O2
Z2568757968	CN1CCC2=C(C=CC=C2N)C1=O	176,22	C10H12N2O
Z1270392259	NC(=O)C1(CCC1)C(O)=O	143,14	C6H9NO3
Z2568751754	CN1C=NC(=CC1=O)C(O)=O	154,13	C6H6N2O3
Z2526439792	COC(C1CCCCC1)C(O)=O	172,22	C9H16O3
Z2526439760	NC1(CCC1)C1=CC=NC=C1	148,21	C9H12N2
Z2522939860	CC1=C(CO)C=C(O1)C(O)=O	156,14	C7H8O4
Z2584270329	OC(=O)CC1=CC2=C(NC=C2)C=C1	175,19	C10H9NO2
Z2522503334	CC1(CCNC1=O)C(O)=O	143,14	C6H9NO3
Z2522503273	OCC1=CC2=C(S1)C=NC=C2	165,21	C8H7NOS
Z26414122	COC1=NC=C(NC(=O)C2CC2C)C=C1	206,25	C11H14N2O2
Z104378192	COC1=C(Br)C=C(C=C1)C(C)N	230,11	C9H12BrNO
Z94601317	CC1=NN(C(C)=C1)C1=CC=C(CO)C=C1	202,26	C12H14N2O
Z96092819	CC1=NOC(N)=C1C(O)=O	142,11	C5H6N2O3
Z172492258	NC1=C(Cl)C=C2OCCOC2=C1	185,61	C8H8ClNO2
Z166605386	OC(=O)CC1=COC(=N1)C1=CC=CC=C1	203,20	C11H9NO3
Z90662846	CC1=C(SC(=N1)C1=CC=CS1)C(O)=O	225,28	C9H7NO2S2
Z94602022	CCS(=O)(=O)C1=CC2=C(OC(C)=N2)C=C1	225,26	C10H11NO3S
Z57756597	COC1=C(OC)C(Cl)=CC(=C1)C#N	197,62	C9H8ClNO2
Z92741624	OC1CS(=O)(=O)CC1NCC1=CC=CO1	231,27	C9H13NO4S
Z118438292	CNC1=C(C#N)C(C)=NS1	153,20	C6H7N3S
Z44585773	ClC1=CC(NC(=O)N2CCCC2)=CC=C1	224,69	C11H13ClN2O
Z32016122	CNC(=O)CNC(=O)C1=CC=C(Br)C=C1	271,11	C10H11BrN2O2
Z56840113	OC(=O)CNC(=O)C1=CC=C(Cl)C=C1	213,62	C9H8ClNO3
Z19739846	NC(=O)COC1=CC=C(C=C1)C#N	176,18	C9H8N2O2
Z56973918	CCN1N=C(C=CC1=O)C(O)=O	168,15	C7H8N2O3
Z56960250	CCNCC1COC2=C(O1)C=CC=C2	193,25	C11H15NO2
Z53834532	CCOC1=CC=C(C=C1)C#N	147,18	C9H9NO
Z31434237	CN1CCN(CC1)C(=O)C1=CC=C(Br)O1	273,13	C10H13BrN2O2
Z56899132	OC(=O)CNC(=O)C1=CC=CO1	169,14	C7H7NO4
Z56899019	OC(=O)CCC(=O)N1CCOCC1	187,20	C8H13NO4
Z56896220	OC(=O)CNC(=O)C1=CC=C(F)C=C1	197,17	C9H8FNO3
Z28136791	CC(=O)NC1=C(Cl)C=CC(Cl)=C1	204,05	C8H7Cl2NO
Z30849291	COC1=C(OC)C=C(NC(C)=O)C=C1	195,22	C10H13NO3
Z3220055146	CC1=C(C#N)C(C)=CC(=O)N1	148,17	C8H8N2O
Z1258519591	NCC1=NOC(=C1)C1=CC=CC=C1	174,20	C10H10N2O
Z1416200883	COC1=CC(N)=C(C=C1)C#N	148,17	C8H8N2O
Z1416200895	NC1=NN=C2CCNCC2=C1	150,19	C7H10N4
Z1317589334	BrC1=CN=C(NC2CCS(=O)C2)N=C1	276,15	C8H10BrN3OS
Z1407007419	CC(C)(O)C1=NC2=C(S1)C=CC=C2	193,26	C10H11NOS
Z1407007417	CC1=CC2=C(NCCN2)N=C1	149,20	C8H11N3
Z1407007506	C1NCC1C1=NC2=C(N1)C=CC=C2	173,22	C10H11N3
Z1405445908	CN(C)C1=C(N)C=NC=N1	138,17	C6H10N4
Z196118762	OCCNC1=CC=C(C=C1)C#N	162,19	C9H10N2O
Z220616774	COC1=C(F)C=C(C=C1)C(C)O	170,18	C9H11FO2
Z295848548	C(N1CCOCC1)C1=NC(=NO1)C1CC1	209,25	C10H15N3O2
Z86242586	CN(C)C1=C(C=CC=N1)C#N	147,18	C8H9N3

Z1318357723	NC1CCN(CC1)C1CCCCC1	182,31	C11H22N2
Z1250132434	CNC1=C(OC)C=C(F)C=C1	155,17	C8H10FNO
Z1003207278	CNS(=O)(=O)CC1CCCCC1	191,29	C8H17NO2S
Z295124002	CNC(C)C1=CC=C(OC)C=C1	165,24	C10H15NO
Z343738014	CC1CN(C(C)CO1)C(=O)C1=CC=CS1	225,31	C11H15NO2S
Z2234631237	CC(N)(C(O)=O)C1=CC=C(Br)C=C1	244,09	C9H10BrNO2
Z1396280317	CC(=O)N1CCN(CC1)C1CCOC1	198,27	C10H18N2O2
Z2235681143	NC1CCOC(C1)C1=CC=NC=C1	178,24	C10H14N2O
Z1262255346	NCC1=CC2=C(OCC2)C=C1	149,19	C9H11NO
Z641408164	CC(N1CCCC1)C1=NC(=NO1)C1CC1	207,28	C11H17N3O
Z1443588587	NC1CCOC2(CCCC2)C1	155,24	C9H17NO
Z169226638	CC(=O)NCCN1CCCCCC1	184,28	C10H20N2O
Z2235409400	CC1CCCC1(C)C(O)=O	142,20	C8H14O2
Z1685106505	CN1C=CN=C1C1=NOC(=N1)C1CCC1	204,23	C10H12N4O
Z644971758	NC(=O)CN1CCCC1C1CCCCC1	210,32	C12H22N2O
Z50145871	CCC1=NN(C(=O)C1)C1=CC=CC=C1	188,23	C11H12N2O
Z168766620	COCC(=O)NC1=CC(F)=CC(F)=C1	201,17	C9H9F2NO2
Z19733482	CN(C)C(=O)COC1=C(C)C=CC=C1	193,25	C11H15NO2
Z32013275	CN(C)C(=O)C1=CC(F)=CC(F)=C1	185,17	C9H9F2NO
Z32372098	COCC(=O)NC1=CC(F)=C(F)C=C1	201,17	C9H9F2NO2
Z54739316	CN1C=CC=C1C(=O)NC1CCCC1	192,26	C11H16N2O
Z144495580	CN(C)C(=O)C1=CSC(=N1)C1=CC=CS1	238,32	C10H10N2OS2
Z16164828	NC(=O)COC1=C2C=CSC2=NC=N1	209,22	C8H7N3O2S
Z1272096656	OC(=O)C1C2CCCCC12	140,18	C8H12O2
Z1272096654	CN(C)C1=NC2=C(C=CC=C2)C(=C1)C#N	197,24	C12H11N3
Z1271843050	COC1=C2C=CC=CC2=NC=C1C#N	184,20	C11H8N2O
Z266487838	CC1=NOC(CNC2=CC=C(C)C=C2)=C1	202,26	C12H14N2O
Z133729708	CC(NS(C)(=O)=O)C1=C(Cl)C=CC=C1	233,71	C9H12ClNO2S
Z1297772359	CC(N)C(O)C1=C(F)C=CC=C1F	187,19	C9H11F2NO
Z1295543908	CC1NC2=C(NC1=O)C=CC=C2	162,19	C9H10N2O
Z382594240	CN(C)C(=O)N1CCN(CC(C)=C)CC1	211,31	C11H21N3O
Z2685356778	CC(C)C1=NC=C(N1C)C1=CN=CC=C1	201,27	C12H15N3
Z1268153230	CCC1=CN=C(CCN)S1	156,25	C7H12N2S
Z1255460290	CC1=NC(CCO)=NC=C1	138,17	C7H10N2O
Z2034678675	CNC1=C(Cl)C=C(Cl)C(=C1)C(N)=O	219,07	C8H8Cl2N2O
Z1918236928	CN1C=C(OCC2=C(C)SC(C)=C2)C=N1	222,31	C11H14N2OS
Z2065616401	NCC1=CC2=C(N=C1)C(F)=CC=C2	176,19	C10H9FN2
Z2065464300	CC(O)C1=C(F)C=CC(N)=C1	155,17	C8H10FNO
Z1259273081	COC1=CC(=CC=C1)C(N)C(O)=O	181,19	C9H11NO3
Z1827602749	CC1CCOC1C(=O)NC1=CN=NS1	213,26	C8H11N3O2S
Z1840852315	CC(O)C(=O)NC1=CC2=C(SN=C2)C=C1	222,26	C10H10N2O2S
Z338089614	FC1=CC(F)=C(CNC(=O)C2CC2)C=C1	211,21	C11H11F2NO
Z1855630367	CC(NC1=CC(C)=C(Br)C(C)=C1)C(N)=O	271,16	C11H15BrN2O
Z1899690168	CC1=CC(=O)N(CC2=NC=CC=C2)N=C1	201,23	C11H11N3O
Z1791179790	CCC1=CC=C(O1)C(=O)NC1=CN=NS1	223,25	C9H9N3O2S
Z57229049	CC(NC(=O)C1=CC=CC=C1)C(O)=O	193,20	C10H11NO3
Z85915745	CC(=O)NC1=C(C=CC(Cl)=C1)C(O)=O	213,62	C9H8ClNO3
Z220542622	OC(=O)C1=CNC(=C1)C(=O)N1CCCC1	208,22	C10H12N2O3
Z85895198	CN(CC(O)=O)C(=O)C1=CC=C(F)C=C1	211,19	C10H10FNO3
Z56827660	OC(=O)CNC(=O)C1=CC(Cl)=CC=C1	213,62	C9H8ClNO3
Z1269638513	CCC(CO)N1CCNCC1	158,25	C8H18N2O
Z1268152406	CC1=NC(NC2CCNCC2)=CC=C1	191,28	C11H17N3
Z1268152319	NC1=NN=C(C=C1)C1CC1	135,17	C7H9N3
Z1267886876	NC1=C(C=C2CCCCC2=N1)C(O)=O	192,22	C10H12N2O2
Z1267882601	CC1=NNC=C1CNC1=CC=CC=C1	187,25	C11H13N3
Z1262254288	Cl.CC(N)C1=C(C)C=CC(C)=C1	185,70	C10H16ClN
Z1262254244	NC1=CC2=C(OCCC2)C=C1	149,19	C9H11NO
Z1262250855	NC(=O)[C@@H]1CC2=C(N1)C=CC=C2	162,19	C9H10N2O
Z2630881496	OC(=O)C1=C(F)C=CC(=C1)C(F)F	190,12	C8H5F3O2
Z1573270315	CC1=C(F)C=CC(NCC2=CSN=N2)=C1	223,27	C10H10FN3S
Z2588063738	O=C1NCCCCCNC2CCCC12	210,32	C12H22N2O
Z2588063517	OCC1CCC2=NC=NN12	139,16	C6H9N3O
Z2588039584	CN1NC(=O)C2=C1C=CC=C2	148,17	C8H8N2O
Z1148797578	N#CC1=CC=C(NC2CCC2)C=C1	172,23	C11H12N2
Z2574909837	COC1=CC(=CC=C1)C1(O)CCC1	178,23	C11H14O2
Z2584270333	COC1=C(C=CC=C1)C1=NNC(CO)=C1	204,23	C11H12N2O2
Z2106594936	OC(=O)C1=NN2C(=C1)N=CC=C2C1CC1	203,20	C10H9N3O2

Z2106600566	CCC(N1C=C(C)C=N1)C(O)=O	168,20	C8H12N2O2
Z2510970259	CCC(C)N1N=C(N)C=C1C(O)=O	183,21	C8H13N3O2
Z2106593001	CN1C=C(C=N1)C1=NN(CC(O)=O)C=C1	206,21	C9H10N4O2
Z2510259409	COC1=C(C(O)=O)C2=C(C=CC=C2)N=C1	203,20	C11H9NO3
Z2510259525	COC(C(O)=O)C1=CN(C)N=C1	170,17	C7H10N2O3
Z275210998	CC1=NC=C(CC(O)=O)S1	157,19	C6H7NO2S
Z1258540488	OC(=O)C1CC2(C1)CCOCC2	170,21	C9H14O3
Z2106593030	CN1C=C(C(O)=O)C(=N1)C1CC1	166,18	C8H10N2O2
Z2510259477	OC(=O)C1CCC2=C(C1)C=NC=N2	178,19	C9H10N2O2
Z1891772658	CO[C@H]1C[C@@H](N(C1)C(C)=O)C(O)=O	187,20	C8H13NO4
Z2412196607	CC1=C(N=C(O)S1)C(O)=O	159,16	C5H5NO3S
Z2380439289	C1CC2(C1)NCCC1=C2NC=N1	163,22	C9H13N3
Z2380439748	CCC1=NC2=C(S1)C=C(N)C=C2	178,25	C9H10N2S
Z57744712	CN(C)S(=O)(=O)N1CCOCC1	194,25	C6H14N2O3S
Z2350907441	OCC1=NN2CCCC2=C1	138,17	C7H10N2O
Z2350907411	OCC=CC1=CC(Br)=CN=C1	214,06	C8H8BrNO
Z2346401046	NC1=C(N=C(C=C1)C(F)(F)F)C(O)=O	206,12	C7H5F3N2O2
Z2335647002	CC1(C)NC(=O)CC1C1=CC=CC=C1	189,26	C12H15NO
Z927319568	CC1=C(C=CO1)C(=O)NC1CCNCC1	208,26	C11H16N2O2
Z1263529586	CC(C)NC(=O)C1=CC(CN)=CC=C1	192,26	C11H16N2O
Z1263529584	CCC(N)C1=NC2=C(N1)C=CC=C2	175,24	C10H13N3
Z166966904	CNC(=O)CN1C(C)=CC2=C1C=CC=C2	202,26	C12H14N2O
Z1262398421	NC1=C(Cl)C=CC(CO)=C1	157,60	C7H8ClNO
Z275169946	CCN1N=CC(Cl)=C1C(O)=O	174,58	C6H7ClN2O2
Z2679906799	CCN1C(C)=NC2=C1C(Br)=CN=C2	240,10	C9H10BrN3
Z2678251351	O=C1CC2CCC(CN1)S2(=O)=O	189,23	C7H11NO3S
Z2677307838		193,25	C11H15NO2
Z1258519590	COC1=C(C=C(Cl)C=C1)C1=NNC(N)=C1	223,66	C10H10ClN3O
Z1396419547	CC1CCCC1NCCO	143,23	C8H17NO
Z1837772594	C=CCC1(CCC1)C(=O)N1CCCC1	193,29	C12H19NO
Z1874954907	CCC(C)N1N=CC(N)=C1C	153,23	C8H15N3
Z99599002	COC1=CC=C(C=C1)C(N)C(C)C	179,26	C11H17NO
Z1863597297	NCCC1=C(C=CC=C1Cl)N1CCCC1	224,73	C12H17ClN2
Z1860991728	NC1CCCC(C1)C1=CC=CC=C1	175,28	C12H17N
Z1694504496	CC1CN(C)CCC1NC1=CN(C)N=C1	208,31	C11H20N4
Z1738061802	O=C1SC=NN1CC1=CSC(=N1)C1CC1	239,31	C9H9N3OS2
Z1682035317	CC1CN(CC2=NNC=C2)C(C)(C)CO1	209,29	C11H19N3O
Z1815156517	CCN1N=CC=C1CCO	140,19	C7H12N2O
Z1703431439	CC(O)C1CCCN(CC2=CC=CS2)C1	225,35	C12H19NOS
Z1801361776	CC1=C(CN)C=CC(=C1)C(F)(F)F	189,18	C9H10F3N
Z372974252	CC1COCCN1C(=O)C1=CC(Cl)=CN1	228,68	C10H13ClN2O2
Z1509566268	FC(F)(F)C1=CC2=C(C=C1)C(=O)NC=C2	213,16	C10H6F3NO
Z1636723439	CNC(=O)C1CNCCO1	144,17	C6H12N2O2
Z1688142576	O=C1CCN(C2CC2)C2=C(N1)C=CC=C2	202,26	C12H14N2O
Z2026151591	CNC1=CC2=C(C=CC=C2)C(=O)N1	174,20	C10H10N2O
Z1741970224	CC1=NC2=C(O1)C=CC(=C2)C(O)=O	177,16	C9H7NO3
Z1889861361	OCC1=CNC2=C1C(F)=CC=C2	165,17	C9H8FNO
Z1743510287	CC1(CNC2=NN=CS2)CCOCC1	213,30	C9H15N3OS
Z1795818295	CC1(CC2=CC=CC=C2)CCNC1=O	189,26	C12H15NO
Z56755628	CN(C)CC1CCCCC1O	157,26	C9H19NO
Z1902703066	NC1=NC=CC(CN2CCC=C(F)C2)=C1	207,25	C11H14FN3
Z1533185622	CC#CCN1C=NC(=CC1=O)C1CCC1	202,26	C12H14N2O
Z1994443056	CCN(C1CCNC1=O)C1CCCC=C1	208,31	C12H20N2O
Z1262691698	NCC1=CC(CO)=CC=C1	137,18	C8H11NO
Z56007122	CCNCC(=O)N1CCCCCC1	184,28	C10H20N2O
Z1695729061	CC1=C(C=CC(CN)=C1)S(C)(=O)=O	199,27	C9H13NO2S
Z1318147459	NCCC1=NN=C(O1)C1=CC=CC=C1	189,22	C10H11N3O
Z136583730	CC1=NOC(=C1)C(=O)NC1=CC=NC=C1	203,20	C10H9N3O2
Z1551476075	COCC1(O)CCNCC1	145,20	C7H15NO2
Z1551463490	CC1=C(C=NN1C1=CC=NC=C1)C(O)=O	203,20	C10H9N3O2
Z1551463575	O=C1CN2C=CN=C2CN1	137,14	C6H7N3O
Z45509011	O=S(=O)(N1CCCC1)C1=CC=CS1	217,30	C8H11NO2S2
Z1446850417	OCC1(CCC1)NC1=NC=C(C=C1)C#N	203,25	C11H13N3O
Z1511495090	CC(CC(O)=O)C1=C(C)NN=C1C	182,22	C9H14N2O2
Z1266823274	NC(C1CC1)C1=CC=C(F)C=C1	165,21	C10H12FN
Z2301527375	NCC1(CN)CCCCC1	142,25	C8H18N2
Z2162668942	CN1N=CC(C)=C1CN1CCC=C(F)C1	209,27	C11H16FN3

Z1483956890	CC1=C(C=CC(=O)N1)C(O)=O	153,14	C7H7NO3
Z1470841574	OC(=O)C1=C(ON=C1)C1CC1	153,14	C7H7NO3
Z1465463133	CC(C)OC1=CC(=CN=C1)C(O)=O	181,19	C9H11NO3
Z1459946718	CN1C=CN=C1C1=NN=C(N)O1	165,16	C6H7N5O
Z1446854992	CN(C)C1=NC2=C(O1)C=CC(=C2)C(O)=O	206,20	C10H10N2O3
Z33547038	NC(=O)CN1C=NC2=C(C=CC=C2)C1=O	203,20	C10H9N3O2
Z228463904	ClC1=CC=C(CN2C=NC(=N2)C#N)C=C1	218,64	C10H7ClN4
Z234853686	CC(CN1CCOCC1)C(O)=O	173,21	C8H15NO3
Z221226038	CC1CN(CCO1)C(=O)C1=CC(C)=NO1	210,23	C10H14N2O3
Z1259087073	CC(C)OC1=NC=C(CN)C=C1	166,22	C9H14N2O
Z1428018975	CCC1CN(CCC1N)S(=O)(=O)C(C)C	234,36	C10H22N2O2S
Z1335657495	CC(C)C1=NN=C(CN)S1	157,24	C6H11N3S
Z1357774582	COC1=CC(=CC(OC)=C1)C1CCNC1	207,27	C12H17NO2
Z1222331569	COC1=CC(=C(CN)C=C1)C(F)(F)F	205,18	C9H10F3NO
Z1266823348	CC(N)CN1CCN(C)CC1	157,26	C8H19N3
Z1139246057	CNC1CCCN(C1)C1=NN=CC=C1	192,27	C10H16N4
Z1245664706	NCC1=CC(F)=CC(F)=C1	143,14	C7H7F2N
Z1250132688	CCC1=C(N=CO1)C(O)=O	141,13	C6H7NO3
Z1245664618	CC(N)CCC1=CC=CC=C1	149,24	C10H15N
Z235582068	CNCC1=NC=CN1C(F)F	161,16	C6H9F2N3
Z1235963371	CC(C)(N)C1=CC(Cl)=CC=C1	169,65	C9H12ClN
Z32014274	CN(C)C(=O)C1=C(Br)C=CC=C1	228,09	C9H10BrNO
Z314447664	CCN(C1CC1)C(=O)C1=CC=C(Cl)S1	229,72	C10H12ClNOS
Z1262237349	NCC1=CC(F)=C(F)C=C1	143,14	C7H7F2N
Z432590156	CCS(=O)(=O)C1=CC2=C(OCCO2)C=C1	228,26	C10H12O4S
Z381729066	CC(C)NC(=O)C1=NNC=C1	153,19	C7H11N3O
Z339866212	CN(CCO)C(=O)NC1=CC(Cl)=CC=C1	228,68	C10H13ClN2O2
Z1945708064	CC1(C)CCCCC1C(O)=O	156,23	C9H16O2
Z1741975545	CC1=NC2=C(O1)C=C(C=C2)C(O)=O	177,16	C9H7NO3
Z1945695970	CC1=C(N=C(N)N=C1)C1=CC=CC=C1	185,23	C11H11N3
Z1945695971	CN1N=C(N=C1N)C1=CC=CS1	180,23	C7H8N4S
Z1729205124	COC1=CC(Cl)=C(CN2C=CN=N2)C=C1	223,66	C10H10ClN3O
Z1203884549	CN(C)S(=O)(=O)C1=CNC=N1	175,21	C5H9N3O2S
Z1446900057	CCOC1=C(F)C=C(C=C1)C#N	165,17	C9H8FNO
Z1895742107	OCC1=NN=C2CCCCN12	153,19	C7H11N3O
Z1895583434	CN1N=CC2=C1NC=CC2=O	149,15	C7H7N3O
Z1889997417	CN1N=C(CC1=O)C1CCCC1	166,22	C9H14N2O
Z1889996025	COC1=C(CC(O)=O)C=C(Br)C=N1	246,06	C8H8BrNO3
Z1889975639	CC1=C(F)C=CC(=C1)N1C=C(CO)N=N1	207,21	C10H10FN3O
Z133622412	CS(=O)(=O)NC1(CCCCC1)C(O)=O	221,27	C8H15NO4S
Z133263888	CC(=O)NC1=C(C=CC(F)=C1)C(O)=O	197,17	C9H8FNO3
Z594038164	NC(C(O)=O)C1=C(Cl)C=C(Cl)C=C1	220,05	C8H7Cl2NO2
Z44590817	O=C(NC1=CC=CC=C1)N1CCOCC1	206,25	C11H14N2O2
Z140637360	COCC(=O)NC1=CC(Cl)=C(C=C1)C#N	224,64	C10H9ClN2O2
Z228463936	ClC1=CC(CN2C=NC(=N2)C#N)=CC=C1	218,64	C10H7ClN4
Z1245633383	CC1=CNC(=O)N1C1=CC=C(C)C=C1	188,23	C11H12N2O
Z164670582	CC1=NC(=CS1)C1=CC2=C(NCC2)C=C1	216,30	C12H12N2S
Z183932940	CC1=NC(C)=C(S1)C(=O)NCC=C	196,27	C9H12N2OS
Z438067480	CCC(=O)NCC1=NN=C2CCCCN12	208,27	C10H16N4O
Z335929200	OCCN1C=NC2=C(C=CC(Cl)=C2)C1=O	224,64	C10H9ClN2O2
Z751928454	CC1=NNC=C1CNC1=CC=C(F)C=C1	205,24	C11H12FN3
Z2379767448	CC(C)C1=CSC(=N1)C1=C(N)SN=N1	226,32	C8H10N4S2
Z177087622	CNC(=O)N1CCC2=C(C1)C=CC=C2	190,25	C11H14N2O
Z1346370700	OCC1=CC(OC(F)F)=CC=C1	174,15	C8H8F2O2
Z2467541171	OC1CCCC11CCCC1	140,23	C9H16O
Z2216711594	NC1=NC(=NC=N1)C(F)(F)F	164,09	C4H3F3N4
Z2213893378	OC(=O)CCC1=NOC=C1	141,13	C6H7NO3
Z2205958575	OCC1(CCNCC1)C1=CC=CC=C1	191,27	C12H17NO
Z2182115476	COC1=C2N=CC=CC2=CC(N)=C1	174,20	C10H10N2O
Z1172115931	OC(=O)C1CCC2=NN=CN2C1	167,17	C7H9N3O2
Z826117806	CC(=O)NC1CC1C1=CC=CC=C1	175,23	C11H13NO
Z2168541837	CCC1=C(N2C=C(Cl)C=CC2=N1)C(O)=O	224,64	C10H9ClN2O2
Z2168499582	OC(=O)C1=C(F)C=C(C=C1F)C(F)(F)F	226,10	C8H3F5O2
Z2168282689	CC1(C)OC2=C(NC1=O)C=C(C=C2)C#N	202,21	C11H10N2O2
Z29693704	O=C(NCC1=CC=CS1)C1=NC=CC=C1	218,27	C11H10N2OS
Z28775462	CCC(=O)NCC1=NC2=C(S1)C=CC=C2	220,29	C11H12N2OS
Z31490388	ClC1=CC=C(S1)C(=O)NCC1CCCO1	245,72	C10H12ClNO2S

Z45511384	CC1CCCN(C1)S(=O)(=O)C1=CC=CS1	245,36	C10H15NO2S2
Z31602909	BrC1=CC=C(O1)C(=O)N1CCNC(=O)C1	273,09	C9H9BrN2O3
Z86948962	CC1=CC(NC(=O)C2=CC=C(Br)O2)=NO1	271,07	C9H7BrN2O3
Z219095946	N#CC1=CN=C(C=C1)N1CCCC1	173,22	C10H11N3
Z317046512	CCOC1=C(C=CC=N1)C#N	148,17	C8H8N2O
Z277468448	C1CCN(CC1)C1=C2N=CNC2=NC=N1	203,25	C10H13N5
Z19736616	NC(=O)COC1=CC(Br)=CC=C1	230,06	C8H8BrNO2
Z432813996	BrC1=C(SC=C1)C(=O)NCC1CCCO1	290,18	C10H12BrNO2S
Z422343286	CC1=C(SC=N1)C(=O)NCC1CCCO1	226,29	C10H14N2O2S
Z1893465070	OCC1CCC(N1)C(F)(F)F	169,15	C6H10F3NO
Z1342206610	CC1=NC=CN1CC1CCCN1	165,24	C9H15N3
Z275170236	CN1N=C(C)C(CN)=C1C	139,20	C7H13N3
Z228584858	NC(CN1CCCC1)C1=CC=CC=C1	190,29	C12H18N2
Z1258578427	CN1C=CC=C1C(O)CN	140,19	C7H12N2O
Z1276496834	CC1=C(C)C(C)=C(N1)C(O)=O	153,18	C8H11NO2
Z1259339728	CC1=CC2=C(NCCO2)C=C1	149,19	C9H11NO
Z1245634050	CN(C)CC1=CC(CO)=CC=C1	165,24	C10H15NO
Z1250132784	CCC(N)C1=NC=C(C)S1	156,25	C7H12N2S
Z1259273100	COC1=C(C=CC=C1)C(N)C(O)=O	181,19	C9H11NO3
Z1350606483	NC(=O)N1CCCCC1C(O)=O	172,18	C7H12N2O3
Z1350606423	COC1=CC(=CC=C1)C1=NON=C1N	191,19	C9H9N3O2
Z57820217	CNS(=O)(=O)C1=C(OC)C=CC(Cl)=C1	235,68	C8H10ClNO3S
Z1342208412	CC1=C(F)C=C(F)C(=C1)C(O)=O	172,13	C8H6F2O2
Z255306774	CC(=O)NCC(O)C1=CC=CC=C1	179,22	C10H13NO2
Z1333873233	OC1CCC2=C1C=C(F)C=C2	152,17	C9H9FO
Z54814038	CS(=O)(=O)NCC1=C(Cl)C=CC=C1	219,68	C8H10ClNO2S
Z228589664	CC1=CC(N)=C(C(O)=O)C(C)=C1	165,19	C9H11NO2
Z1324054906	COCC1(C)CNC(=O)C1	143,19	C7H13NO2
Z1324054904	CC1=C(C=CC=C1)C(N)CC(O)=O	179,22	C10H13NO2
Z1815157608	NC1=NN(C2CCCC2)C(=C1)C(O)=O	195,22	C9H13N3O2
Z1815156017	CN1C=CC2=C1C(N)=CC=C2	146,19	C9H10N2
Z1815155418	CC1=C(C)C(C(O)=O)=C(Cl)S1	190,64	C7H7ClO2S
Z1815155476	OC(=O)CS(=O)C1=CC=C(Cl)C=C1	218,65	C8H7ClO3S
Z1815155460	CC(=O)NC1=C(N=CC=C1)C(O)=O	180,16	C8H8N2O3
Z1814381814	COC1=CC(C)=C(C=C1)C(C)C(O)=O	194,23	C11H14O3
Z1665934308	CCC1=C(F)C(=NC=N1)N1CC(CO)C1	211,24	C10H14FN3O
Z1278605667	CC1CCC(C)N(C1)C(=O)NC1CC1	196,29	C11H20N2O
Z1791224447	O=C1NCCC1C1=CN=CC=C1	162,19	C9H10N2O
Z1791182212	NCC1=C(OC2=C1C=CC=C2)C(N)=O	190,20	C10H10N2O2
Z1787355592	CC(C)C1=CC(=O)N(C)N1	140,19	C7H12N2O
Z1787355945	OC(=O)C1(CC2=C(F)C=CC=C2)CC1	194,21	C11H11FO2
Z1787158625	CC(=O)NC1CCC(N)CC1	156,23	C8H16N2O
Z805551440	CN(C)S(=O)(=O)C1=CNN=C1	175,21	C5H9N3O2S
Z1778350445	CC1=C(C=NN1)C1=NN=C(N)S1	181,22	C6H7N5S
Z98996956	NC(=O)CN1C(=O)OC2=C1C=CC(Cl)=C2	226,62	C9H7ClN2O3
Z237599214	CCC(O)C1=NC2=C(C=CC=C2)N1CC	204,27	C12H16N2O
Z431947156	CC1=C(C)C=C(C#N)C(=O)N1	148,17	C8H8N2O
Z431947158	CC1=CC=C(C(O)=O)C(=O)N1	153,14	C7H7NO3
Z92433384	CNC(=O)C(C)OC1=C(F)C=C(F)C=C1	215,20	C10H11F2NO2
Z425449750	NC(=O)CN1C=C(N)C=N1	140,15	C5H8N4O
Z425389656	CC1=CC(C(O)=O)=C(C)N1C1=NC=CS1	222,26	C10H10N2O2S
Z65558737	OC(=O)COC1=CC(Cl)=C(Cl)C=C1	221,03	C8H6Cl2O3
Z406680118	CC1=NN(CC1)C1=CC=C(C=C1)C(O)=O	204,23	C11H12N2O2
Z234894117	CC1=CC=C(S1)C1=NN=C(N)O1	181,21	C7H7N3OS
Z398556904	CC1=CC=C(O1)C1=NC(=CS1)C(O)=O	209,22	C9H7NO3S
Z397585712	BrC1=CN=C(C=C1)N1CCNCC1	242,12	C9H12BrN3
Z384360874	BrC1=CNC(=C1)C(=O)NCC1CCOC1	273,13	C10H13BrN2O2
Z234894711	ClC1=CN=C(C=C1)N1CCNCC1	197,67	C9H12ClN3
Z18390836	CN(C)C(=O)COC1=C(Cl)C=C(F)C=C1	231,65	C10H11ClFNO2
Z381474098	O=C(NC1=NC=CC=C1)C1COCCO1	208,22	C10H12N2O3
Z409508902	CC1COCCN1C(=O)NC1=NN=CS1	228,27	C8H12N4O2S
Z992716962	OCC1=CN2C=C(Cl)C=C(Cl)C2=N1	217,05	C8H6Cl2N2O
Z275255230	OC(=O)C1=CC=C(CN2C=C(Br)C=N2)O1	271,07	C9H7BrN2O3
Z27633535	CC(NC(=O)C1CCC1)C1=NC=CC=C1	204,27	C12H16N2O
Z29672864	CC1=NN=C(NC(=O)C2=CC=CC=C2)S1	219,26	C10H9N3OS
